# A decoupling strategy toward spatiotemporal regulation and biomechanical transmission of sandwiched scaffold for osteochondral regeneration

**DOI:** 10.1038/s41467-026-71810-4

**Published:** 2026-04-17

**Authors:** Xuemiao Liu, Mingze Du, Weiguo Zhang, Kang Tian, Fuzhen Yuan, Xing Wang

**Affiliations:** 1 Beijing National Laboratory for Molecular Sciences, Institute of Chemistry, Chinese Academy of Sciences, Beijing, 100190 China; 2 Department of Bone & Joint, First Affiliated Hospital of Dalian Medical University, Dalian, 116000 China; 3 Department of Sports Medicine, Peking University Third Hospital, Beijing, 100191 China

**Keywords:** Translational research, Outcomes research, Biomedical engineering, Biomaterials

## Abstract

Osteochondral repair remains a great challenge due to the hierarchical complexity and functional heterogeneity. Despite significant efforts in gradient scaffolds, few strategies concern wavy interface and biomechanical functions of calcified cartilage layer (CCL) between the chondro-osseous junction. Here, a sandwich-layered hydrogel containing a biomimetic wavy CCL is developed with hypoxia-inducible capacity, spatiotemporal regulation and biomechanical transmission using a decoupling strategy. The scaffold presents intrinsic Fe^3+^-chelating with high activation and durable expression of HIF-1α signaling to facilitate cartilage development. Innovatively, synergistic effects of KGN and Mg^2+^ suppress chondrogenic hypertrophy and boost chondrogenesis. Instead, chondrocytes transition into hypertrophy associated with Cu^2+^-induced vascularization trigger endochondral ossification. Finite element analysis and transcriptome studies reveal the role of wavy CCL in alleviating stress concentration and protecting articular cartilage, thus presenting high efficacy in rabbits’ osteochondral repair. Collectively, this study emphasizes the indispensability of wavy CCL in multileveled scaffolds toward osteochondral regeneration.

## Abstract (web summary)

Osteochondral repair is difficult due to tissue complexity. Here, authors develop a sandwiched hydrogel scaffold with a wavy calcified layer as a strategy to alleviate stress concentration and protect articular cartilage for osteochondral regeneration.

## Introduction

Full-thickness osteochondral defects are generally caused by trauma or degenerative diseases, leading to joint pain, functional impairment, and progressive tissue degeneration^1^. The challenge of osteochondral repair stems from the reconstruction of various multileveled and continuous gradients in connective tissue, such as the gradients in biochemical compositions and biophysical conditions^2^. The clinical therapies mainly include microfracture surgery, autologous chondrocyte implantation (ACI) and osteochondral autograft transplantation (OAT). Whereas, the fibrocartilage induced by microfracture surgery has low mechanical strength, poor wear resistance and liable degenerative changes. ACI has unstable efficacy due to chondrocytes dedifferentiation after in vitro expansion and low integration rate of the grafts (<30%), and OAT is limited by insufficient donor sources, difficult size matching and the risk of secondary lesions in the donor area. Furthermore, these methods are not capable of providing mechanical support for deep defects, and the postoperative inflammatory microenvironment and scar formation may cause secondary damage to the repaired tissues^3–5^. So, the existing technologies cannot meet the requirements of heterogeneous regeneration and long-term functional recovery for complex osteochondral tissues, highlighting the urgent necessity for technological innovation and revolution in osteochondral repair.

The high incidence of trauma-related injuries and the limitations of current clinical therapy have motivated tremendous efforts to develop various gradient scaffolds that mimic the biocompatibility, morphology, and function of natural osteochondral tissues by precisely controlling the composition and structure of tissue engineering scaffold biomaterials. While previous studies have explored individual bioactive components (e.g., Mg^2+^ for cartilage-bone protection^6^, Cu^2+^ for vasculogenesis^7^, KGN for chondrogenesis^8^), these efforts primarily focus on the single-component or single-tissue applications. However, during the complex process of osteochondral repair, it is necessary to precisely regulate the behaviors of different cells at various stages and in different locations (such as inhibiting the hypertrophy of chondrocytes in the cartilage layer while promoting vascularization in the subchondral bone layer). So, simply regulating a certain process is far from sufficient to meet the complex spatiotemporal requirements involved in osteochondral regeneration. Additionally, despite recent advancement in hydrogel-based gradient scaffolds for osteochondral repair, the manufacturing process and preparation procedures are rather complicated and intractable. For example, the layer-by-layer assembly, three-dimensional (3D) printing and electrospinning methods generally require precision control, expensive equipment, and time-consuming process. Even so, the personalized heterogeneous architectures still have many gradient flaws and the interlayer interfaces are prone to peeling off. The discontinuous and abrupt connections between the chondro-osseous junction may lead to the delamination, making it difficult to reestablish the heterogeneous nature of osteochondral tissues^9,10^. In recent years, the emergence of continuous gradient scaffolds effectively avoids interlayer peeling, provides a smoother transition interface, and thereby improves the biomechanical properties and repair outcomes. For example, Zhang et al. proposed a hydrogel scaffold that achieved continuous biophysical and biochemical gradients between cartilage and subchondral bone, effectively simulating the natural mineral and mechanical gradients^11^. Xu et al. designed a mechanical-magnetic gradient hydrogel to achieve a continuous increase in the mechanical modulus from the cartilage layer to subchondral bone layer, thereby coordinating differentiation by regulating the gradient expression of YAP1^12^. However, these gradient scaffolds still have certain limitations because they lack a core structure of calcified cartilage layer (CCL) between articular cartilage and subchondral bone that takes on crucial biomechanical functions in joint development and ordinary activities^13^, thereby incapable of accurately simulating the gradient mechanical properties of natural osteochondral tissues. Structurally, the characteristic of CCL lies in the presence of a distinct wavy pattern on its upper boundary. Under an optical microscope, this pattern manifests as raised eosinophilic lines with a thickness of 10 μm, with a three-dimensional undulating shape composed of alternating peaks and valleys, forming a dynamic adaptive structure. It becomes more pronounced when subjected to mechanical stimuli such as long-term weight-bearing or movement^14–16^. However, up to now, the reported multilayered scaffolds have rarely mentioned the biomimetic design and elaborate construction of the wavy CCL interface within the chondro-osseous junction, which invariably results in the degenerative changes of the newborn tissues due to the negligence of its wavy tidemark and key biomechanical functions in stabilizing the integrity of osteochondral interface. In addition, the periodically undulating geometric features achieve functional optimization to minimize the stress damage via a multistage mechanical transmission mechanism. Under vertical loading, the wavy interface can transfer local compressive stress to the subchondral bone after three-dimensional stress dispersion, thereby significantly reducing stress concentration coefficient on the cartilage surface and effectively delaying the cartilage degeneration process^17^. Meanwhile, the transition of elastic modulus formed by the mineralization gradient can match the strain compatibility between the cartilage and the subchondral bone, which reduces the difference in interfacial shear strain. More importantly, the mechanical interlocking effect of this wavy layer can enhance the interfacial shear strength by anchoring the collagen fiber and mineral complex, thus resisting the periodic shear stress generated during the movement and preventing the detachment of cartilage layer^18^. Unfortunately, despite these insights, current research mainly focuses on the design of biphasic or gradient scaffolds, generally ignoring the biomimetic reconstruction of wavy CCL with the critical biomechanical transmission functions^16,19–21^. Even if a few studies attempted to construct CCL, they only replace with a flat mineralized layer, completely depriving the physiological and biomechanical performances of wavy CCL^22,23^. Consequently, the absence of CCL or simple adoption of flat interface can lead to a mismatch in biomechanical transmission, thereby impairing the high-quality osteochondral repair.

On the other hand, the weak biological capacity and insufficient regulation of cell behaviors make it extremely difficult to achieve the divisional reestablishment mirroring native osteochondral tissues. Especially, the intrinsic characteristics of cartilage without blood vessels, lymphatics, and nerve innervation result in its lower oxygen tension compared to the vascularized bone tissue^24^. This hypoxic environment activates the hypoxia-inducible factor-1α (HIF-1α) signaling pathway in chondrocytes, which regulates the metabolic behavior for chondrocytes renewal and intercellular matrix synthesis^25^. Therefore, using hypoxia-mimicking compounds to upregulate the expressions of HIF-1α and its related downstream genes is an efficient strategy to improve chondrogenic differentiation of bone marrow mesenchymal stem cells (BMSCs). Wherein, either a low oxygen level or the elimination of iron ions can effectively improve HIF-1α stability. Compared to the commonly used but highly toxic iron chelators of deferoxamine (DFO)^26^, a purely biomaterial-based method that can effectively chelate iron ions without incorporating hypoxia-mimetic agents is an attractive alternative. Moreover, a recent study found that prolonged expression of HIF-1α signaling in chondrocytes could cause pathological hypertrophy and regulate the transition into mature hypertrophic chondrocytes, ultimately activating the endochondral ossification (ECO) process^27^. It indicates the indispensable role of HIF-1α in early cartilage development and later bone formation. Under this circumstance, by controlling the window of HIF-1α expression and its spatiotemporal maintenance in situ at different layers, simultaneously achieving the inhibition of chondrocytes hypertrophy in the cartilage layer and facilitation of the ECO process in the subchondral bone layer is expected to pioneer an innovative strategy in boosting osteochondral repair. Apart from hypoxia, full-thickness osteochondral regeneration is a complex process that also requires the stimulation and intervention from multiple bioactive factors. Combinedly, the co-incorporation of HIF-1α regulation with other biological components (e.g., bioactive ions, therapeutic drugs) has become an effective attempts to facilitate intercellular matrix and neo-osteochondral formation.

**Fig. 1:**
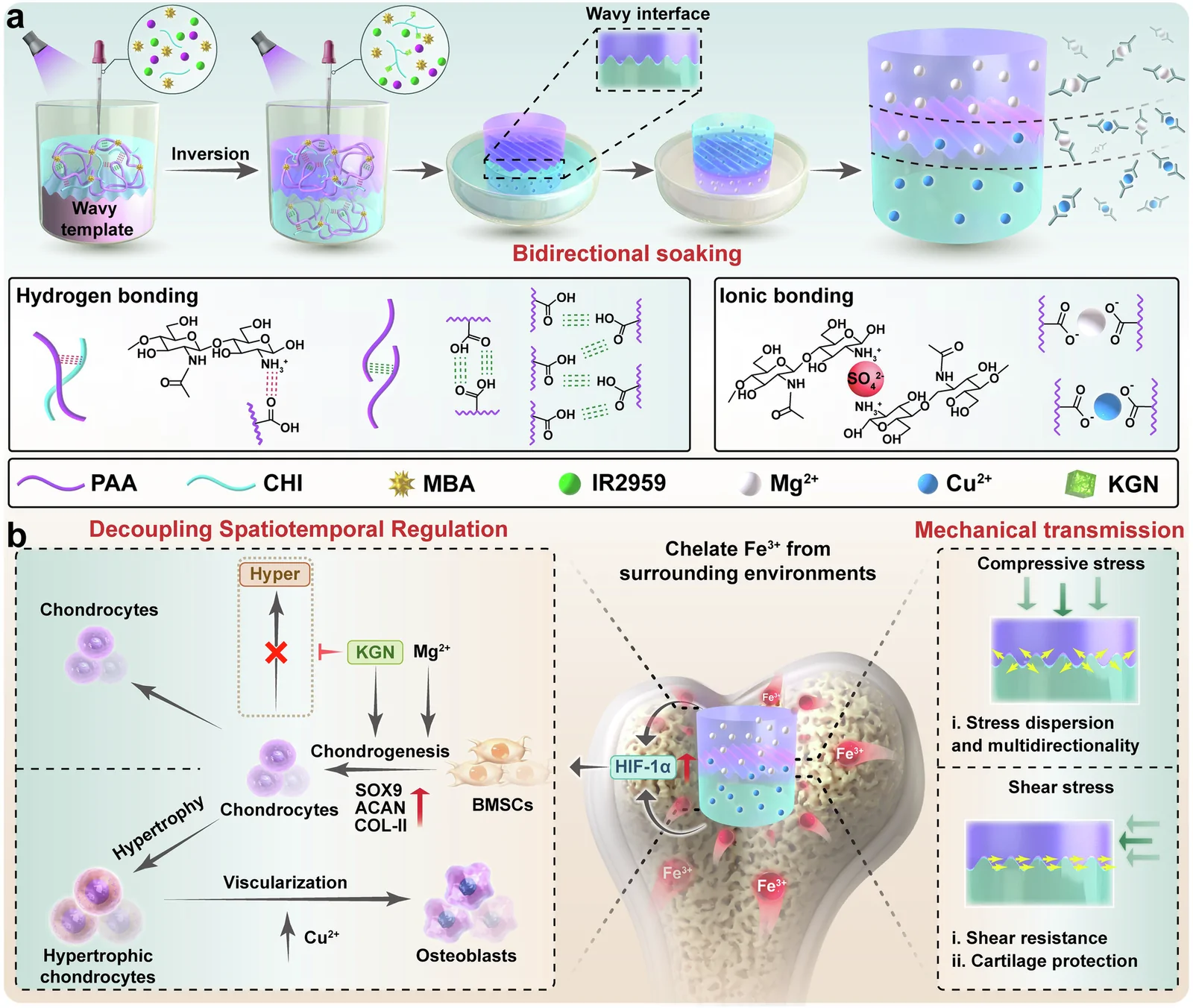
Schematic illustration of a multileveled bioactive WCCL-DG scaffold for full-thickness osteochondral regeneration. **a** The fabrication process of sandwiched WCCL-DG scaffold featuring a biomimetic wavy interface using a wavy-shaped template and stepwise bidirectional soaking strategy. The height ratio of three layers is 45:10:45. **b** The WCCL-DG scaffold containing bioactive Mg^2+^/Cu^2+^ and KGN agents persistently activates the HIF-1α signaling, heterogeneously regulates the intervention of hypertrophic chondrocytes and geometrically provides the biomechanical transmission function, achieving an ideal regeneration for osteochondral defects.

Based on the above rationale, here we endeavored to develop a sandwiched hydrogel scaffold containing a biomimetic wavy CCL interface via a wavy-shaped template and stepwise bidirectional soaking strategy for full-thickness osteochondral repair. The wavy CCL-embedded composite gel (PAA-CHI-KGN/CCL/PAA-CHI) was firstly prepared by ultraviolet (UV)- triggered polymerization of acrylic acid (AA) and short-chain chitosan (CHI) at the top of template for mimicking subchondral bone layer, after a simple inversion, the polymerization of AA monomer and kartogenin (KGN)-conjugated chitosan (CHI-KGN) was carried out at the wavy surface for mimicking cartilage layer. Then, this hydrogel was converted to wavy CCL-embedded double network gel (WCCL-DG, PAA-CHI-KGN-Mg^2+^/CCL/PAA-CHI-Cu^2+^) after stepwise bidirectional soaking in MgSO_4_/CuSO_4_ solutions via control of soaking depth and time, enabling spatiotemporal regulation of the architecture, function, and biochemical gradients for each layer to meet the personalized repair needs of different depth defects (Fig. 1a). Moreover, the carboxyl groups supplied on this sandwich-layered hydrogel could efficiently chelate iron ions to mimic a hypoxia microenvironment for improving the survival of chondrocytes and developing a cartilage matrix. To avoid the formation of hypertrophic chondrocytes in the cartilage layer, KGN was applied to inhibit the expression of hypertrophy marker and maintain the hyaline cartilage phenotype, and the incorporation of bioactive Mg^2+^ synergistically promoted the chondrogenic differentiation of BMSCs. Comparatively, the sustained expression of HIF-1α signaling induced the chondrocytes hypertrophy without the KGN intervention, associated with Cu^2+^-promoted vascularization, collectively promoting ECO process in the subchondral bone layer. This decoupling strategy effectively avoids signal interference and achieves the coordinated regulation of cartilage anti-hypertrophy and bone pro-vascularization process (Fig. 1b). More importantly, we have fabricated a biomimetic wavy CCL interface and reveal its biomechanical transmission function on alleviating stress concentration and protecting artificial cartilage through the finite element analysis (FEA) and transcriptome studies. We further validated the synergistical effect of sandwiched WCCL-DG scaffold in long-term expression of HIF-1α signaling in situ, controllable intervention of hypertrophic chondrocytes and biomechanical transmission through in vivo studies to elucidate the anisotropic arrangement and functional recapitulation for high-quality osteochondral regeneration. Collectively, through the scaffold with a wavy CCL-embedded structure, we have comprehensively addressed key issues such as the complex preparation process, out-of-control signal regulation, and neglected biomechanical transmission, consequently offering a clinically feasible and effective therapeutic approach for full-thickness osteochondral repair.

## Results

### Hydrogel fabrication and optimization formulation

**Fig. 2:**
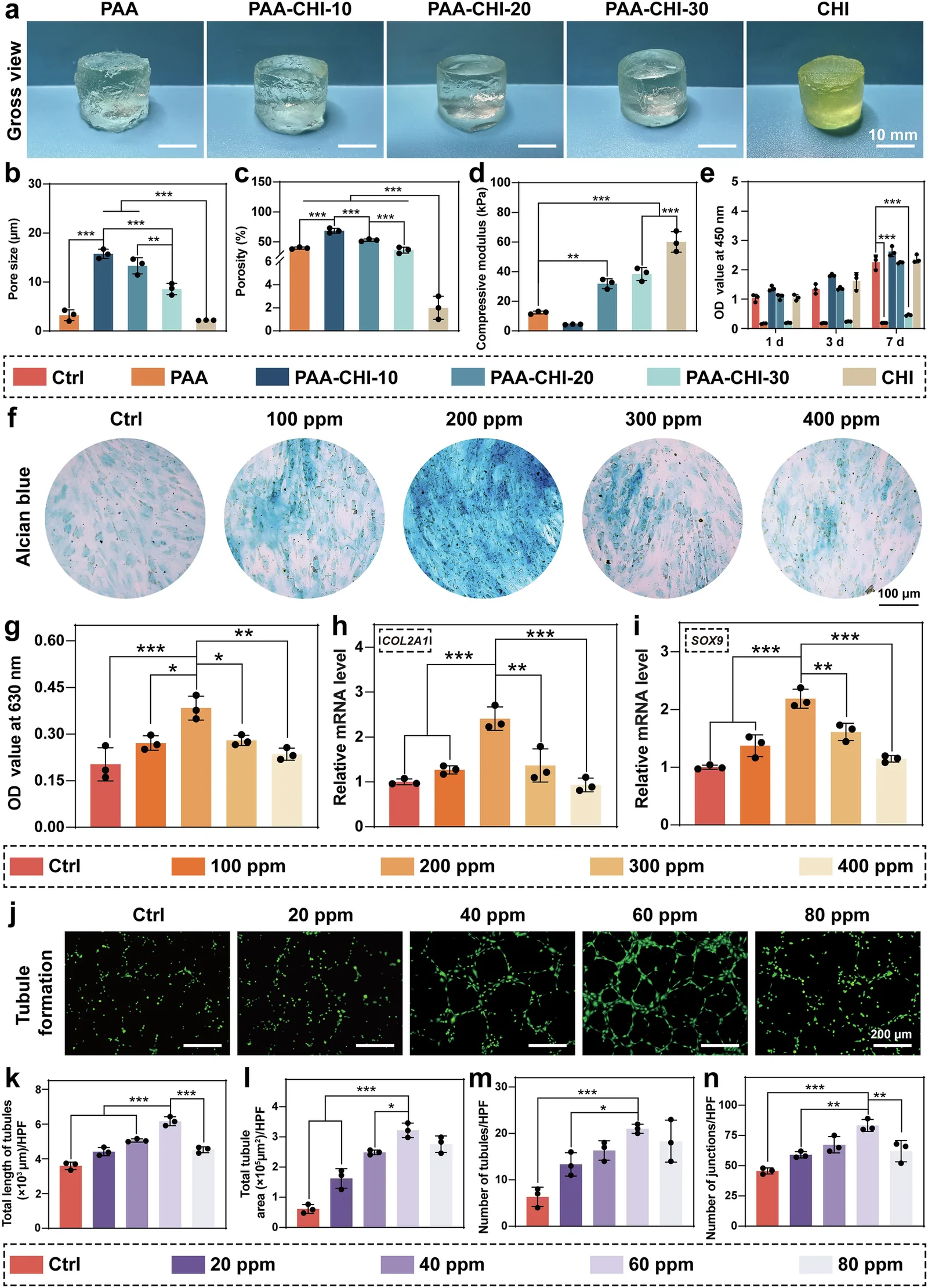
Optimization formulation of PAA-CHI hydrogel and ionic soaking concentration. **a–d** Gross view, pore size, porosity, and compressive behaviors of PAA, PAA-CHI-10, PAA-CHI-20, PAA-CHI-30, and CHI hydrogels. (for pore size: **p = 0.0029, ***p < 0.0001, ***p = 0.0001, ***p < 0.0001; for porosity: ***p < 0.0001, ***p = 0.0005, ***p = 0.0009, ***p < 0.0001; for com*p*ressive modulus: **p = 0.001, ***p = 0.0004, ***p < 0.0001, n = 3 independent samples). **e** CCK-8 assay of BMSCs after co-culture with various hydrogels at specific times. (***p < 0.0001, ***p < 0.0001, *n* = 3 independent samples). **f** Effect of Mg^2+^ concentration on the formation of GAG by Alcian blue staining in BMSCs cultured for 2 weeks. **g** Quantitative analysis of staining intensity in **f**. (*p = 0.0136, *p = 0.0218, ***p = 0.0004, **p = 0.002, n = 3 independent samples). **h**,** i** Relative mRNA expression of chondrogenic differentiation-related indicators after co-culture of BMSCs with different concentrations of Mg^2+^ for 2 weeks. (for *COL2A1*: **p = 0.0013, ***p = 0.0001, ***p = 0.0006, ***p < 0.0001; for *SOX9*: **p = 0.0025, ***p < 0.0001, ***p = 0.0002, ***p < 0.0001, n = 3 independent samples). **j** Live cell staining of HUVECs after co-culture with different concentrations of Cu^2+^ for 6 h. **k–n** Statistical analysis of the total length of tubules, total tubules area, number of tubules and junctions in high-magnification field at 6 h. (for total length of tubules: ***p < 0.0001, ***p = 0.0004, ***p < 0.0001; for total tubules area: *p = 0.0196, ***p < 0.0001; for number of tubules: ***p = 0.0004, *p = 0.0371; for number of junctions: **p = 0.0026, **p = 0.0066, ***p < 0.0001, n = 3 independent samples). Data in **b–e**,** g–i**,** k–n** were presented as means ± SD. Statistical significance was determined using the one-way ANOVA with Tukey’s post-hoc test. Source data are provided as a Source Data file.

PAA-CHI hydrogel was simply prepared via UV-initiated radical polymerization in which the PAA was crosslinked by covalent bonds and hydrogen interactions and CHI was well dispersed in the matrix (Supplementary Fig. 1). Since the PAA contents could directly affect the microstructure and property of the hydrogels, we prepared three composite hydrogels (PAA-CHI-10, PAA-CHI-20, and PAA-CHI-30) with different PAA contents, and used the chemically crosslinked PAA hydrogel and the physically crosslinked CHI hydrogel as control groups (Fig. 2a). Compared to the brittle CHI hydrogel, the ductile PAA chains enabled the favorable deformability and compressive property for the PAA-CHI hydrogel that could withstand a higher compressive strain (ε = 97%), significantly improving the toughness and pressure resistance to adapt to the repeated stress environment inside the joint (Supplementary Fig. 2). Along with the increase of PAA content, the pore size and porosity were gradually decreased and the mechanical strength was reasonably enhanced (Fig. 2b–d and Supplementary Fig. 3). However, CCK-8 assay and live/dead staining displayed that the excessive PAA proportion could restrict cell proliferation, which was due to the release of a large amount of H^+^ and the formation of harsh acidic environments despite the presence of alkaline polysaccharides in the PAA-CHI hydrogel (Fig. 2e and Supplementary Fig. 4)^28^. Taken together, comprehensive analysis of the pore size, mechanical strength, and cell biocompatibility of these PAA-CHI hydrogels, we selected the PAA-CHI-20 hydrogel for the further investigation.

Bioactive metal ions play an important role in maintaining biological functions, regulating metabolism, and promoting tissue repair^29^. For example, Mg^2+^ can mediate the synthesis of extracellular matrix by regulating HIF-1α to promote cartilage regeneration while Cu^2+^ can promote osteogenesis by promoting the vasculogenesis^30^. So, we designed concentration gradient experiments to determine the optimal soaking conditions for the cartilage layer and subchondral bone layer, respectively. For the cartilage layer, we set up a gradient of Mg^2+^ concentrations with 100, 200, 300, and 400 ppm. Alcian blue staining showed that in the 200-ppm group, the deposition of glycosaminoglycans was the most significant (Fig. 2f,g). The quantitative reverse transcription polymerase chain reaction (qRT-PCR) results also revealed that a Mg^2+^ concentration of 200 ppm was the most beneficial for the upregulation of *COL2A1* and *SOX9* (Fig. 2h,i), which was highly consistent with the previous study^31^. For the subchondral bone layer, considering the potential cytotoxicity of high Cu^2+^ concentration, we established a gradient of Cu^2+^ gradients with 20, 40, 60, and 80 ppm^32^. Tubule formation assays revealed that a Cu^2+^ concentration of 60 ppm exhibited the optimal vasculogenic effects, with the longest tubule length, largest tubule area, highest number of tubules and junctions (Fig. 2j-n). Notably, as both excessively high and low ion concentrations compromise biological efficacy, we sought to define the optimal soaking concentrations required to achieve target release levels of Mg^2+^ and Cu^2+^ for chondrogenic and osteogenic functions, respectively. To this end, we monitored the ion release profiles of hydrogels treated with varying concentrations of Mg^2+^ and Cu^2+^, establishing the correlation between initial soaking concentration and sustained release concentration. The results showed that the PAA-CHI-20 hydrogel soaked in a 0.5 M MgSO_4_ solution continuously released Mg^2+^ with a concentration reaching approximately 200 ppm, achieving best chondrogenic differentiation effect. When the PAA-CHI-20 hydrogel was soaked in a 0.1 M CuSO_4_ solution, it could release Cu^2+^ with a concentration of approximately 60 ppm, which matched the ideal vasculogenic concentration (Supplementary Fig. 5). The stable spatial distribution of Mg^2+^ and Cu^2+^ (with no significant cross-diffusion) was attributed to the high molar ratio of carboxyl groups to metal ions and the strong ion/chelation interaction, ensuring a continuous biochemical gradient over time. This rational matching of immersion concentration to the target release level enabled the precise temporal control of biological functions on the osteochondral scaffold.

KGN, as a non-protein small molecule chondrogenic inducer, plays a crucial role in the construction of functional hydrogels for cartilage regeneration, especially for effective exhibition of the hypertrophy of chondrocytes^33^. For this purpose, we synthesized the KGN-conjoined CHI with a grafting ratio of KGN at 4.9% through a simple amidation reaction via the verification of ^1^H NMR and FT-IR (Supplementary Fig. 6), and further developed the PAA-CHI-KGN-Mg^2+^ hydrogel and PAA-CHI-Cu^2+^ hydrogel through the categorized soaking strategy in MgSO_4_ and CuSO_4_ solutions with optimal soaking concentration, which were determined as the biomimetic cartilage and subchondral bone scaffolds, respectively. As shown in supplementary Fig. 7, the transparent PAA-CHI-20 hydrogel was changed into white and blue opacity for the PAA-CHI-KGN-Mg^2+^ and PAA-CHI-Cu^2+^ hydrogels. During this soaking process, divalent metal and sulfate ions were permeated into the hydrogel and served as crosslinkers to form amino-anion and carboxyl-cations domains: (i) the sulfate ions triggered the formation of ionic CHI networks, thereby resulting in recoverable energy dissipation mechanism^34^; and (ii) the strong carboxyl/Mg^2+^ or Cu^2+^ electrostatic interaction induced ionic crosslinks of PAA chains. Under this circumstance, various pore architectures and biochemical gradients can be simply customized via tailoring the salt concentration and immersion time, enabling the precise regulation of the structure and function for each layer to meet personalized repair needs of different depth defects. So, a high concentration of MgSO_4_ solution (0.5 M) was used for the fabrication of cartilage layer-mimicking PAA-CHI-KGN-Mg^2+^ hydrogel (pore size of 22.6 ± 1.1 μm) and a relatively low concentration of CuSO_4_ solution (0.1 M) was used for the construction of bone layer-mimicking PAA-CHI-Cu^2+^ hydrogel (pore size of 43.5 ± 0.9 μm). The pore size difference was mainly attributed to the higher Mg^2+^ concentration though its coordination effect with carboxyl groups was lower than that of the Cu^2+^, which also conformed to the shrinkage characteristics of the high cross-linking density system predicted by the Flory-Rehner theory and successfully simulated the gradient structure characteristics of the osteochondral interface^35,36^. EDS further verified the uniform distribution of O and C elements, as well as the spatial continuity of the characteristic signals of Mg^2+^ and Cu^2+^, indicating that the bioactive metal ions had been uniformly integrated into their respective polymer networks (Supplementary Fig. 7). Additionally, these PAA-CHI-KGN-Mg^2+^ and PAA-CHI-Cu^2+^ hydrogels displayed satisfactory cell biocompatibility and proliferation ability by verifications of live/dead staining and CCK-8 assay of both BMSCs and chondrocytes (Supplementary Fig. 8).

Compared to the PAA-CHI hydrogel, PAA-CHI-KGN-Mg^2+^ and PAA-CHI-Cu^2+^ hydrogels showed significant mechanical enhancement due to the transition from composite hydrogel to DN hydrogel by the ionic soaking strategy^37–39^. Both hydrogels could withstand stretching, torsion, crossing and knotting even under large deformation (Supplementary Fig. 9), demonstrating the favorable elasticity and toughness. Rheological measurement revealed their higher storage modulus (G’) than that of the PAA-CHI hydrogel, indicating that a more ordered microstructure and CHI ionic cross-linking network were formed by soaking in the divalent ion solutions. Additionally, on account of higher Cu^2+^/carboxyl coordination effect but lower salt concentration, the mechanical properties were slightly stronger than those of PAA-CHI-KGN-Mg^2+^ hydrogel, with clear verification of rheological and compressive properties (Supplementary Figs. 10,11). We further carried out a dynamic amplitude test to evaluate the self-healing property. As shown in Supplementary Fig. 12, a huge strain of 1000% was first applied to break its cross-linking network, and then a rapid recovery of G′ and G″ to their initial values were observed when the strain was restored to 1%. This self-healing property was mainly due to the large number of strong hydrogen bonds remaining among PAA chains even in the presence of a small metal/carboxyl complexation, which ensured the stable fusion of the cartilage layer and the subchondral bone layer as well as the long-term stability in vivo. Even so, the Mg^2+^/Cu^2+^ could disrupt certain hydrogen bonds between PAA chains to expose carboxyl groups, and these dynamic dissociation of metal-carboxyl complexes in aqueous environment increased charge repulsion and water molecule infiltration. The released carboxyl anions were driven to expand the conformation of the polymer chain segments by electrostatic repulsion, while the dissociated hydrated metal ions promoted the water molecule permeation through the osmotic pressure effect, jointly leading to an increase in the swelling ratio. It was particularly important to note that in this experiment, a low concentration of metal ion solution and a short-term soaking treatment process were adopted, resulting in a relatively low metal coordination conversion rate of carboxyl groups. In this case, the enhancing effect of metal coordination on network cross-linking failed to fully counteract its promoting effect on hydrophilicity and electrostatic repulsion. Reasonably, the PAA-CHI-KGN-Mg^2+^ and PAA-CHI-Cu^2+^ hydrogels exhibited higher swelling ratio than that of the PAA-CHI hydrogel (Supplementary Fig. 13). Furthermore, we evaluated the KGN release profile from the PAA-CHI-KGN-Mg^2+^ hydrogel in PBS solutions (Supplementary Fig. 14), which showed that the release rate of KGN increased from 4.4% to 68.1% from the 1st to the 7th week, corresponding to concentrations ranging from 5.1 μM to 73.9 μM, indicating that KGN could consistently meet the effective usage concentration requirement in vivo (100 nM–100 μM)^40^.

In addition to tailor the microstructure and property of DN hydrogels, these divalent metal ions also contributed to many biological functions, such as the antibacterial property, recruitment, and differentiation capacity of endogenous BMSCs. As shown in Supplementary Fig. 15, compared to the PAA-CHI hydrogel, PAA-CHI-KGN-Mg^2+^ and PAA-CHI-Cu^2+^ hydrogels displayed the superior antibacterial properties against *Escherichia coli* (*E. coli*) and *Staphylococcus aureus* (*S. aureus*) with the larger inhibition zone areas, which was of importance in preventing the failure of internal implants and poor wound healing after orthopedic surgery^41,42^. Additionally, instead of expensive chemokines to recruit BMSCs, these two metal ions and bioactive KGN participant could endow the PAA-CHI-KGN-Mg^2+^ and PAA-CHI-Cu^2+^ hydrogels with recruitment capacity by verification of cell scratch and transwell experiments (Supplementary Figs. 16,17). The scratch test showed that healing area of the PAA-CHI-KGN-Mg^2+^ and PAA-CHI-Cu^2+^ groups reached 90.7 ± 6.0% and 76.7 ± 5.5%, higher than that of the control group. Similarly, the transwell results revealed a greater number of cell migrations of these two hydrogel groups than that in the control group, convincingly confirming that the construction of bioactive metal ions and KGN on enhancing the migration ability of stem cells and facilitating the tissue regeneration.

### Design strategy of sandwiched WCCL-DG scaffold and its hypoxia-inducible capacity

**Fig. 3:**
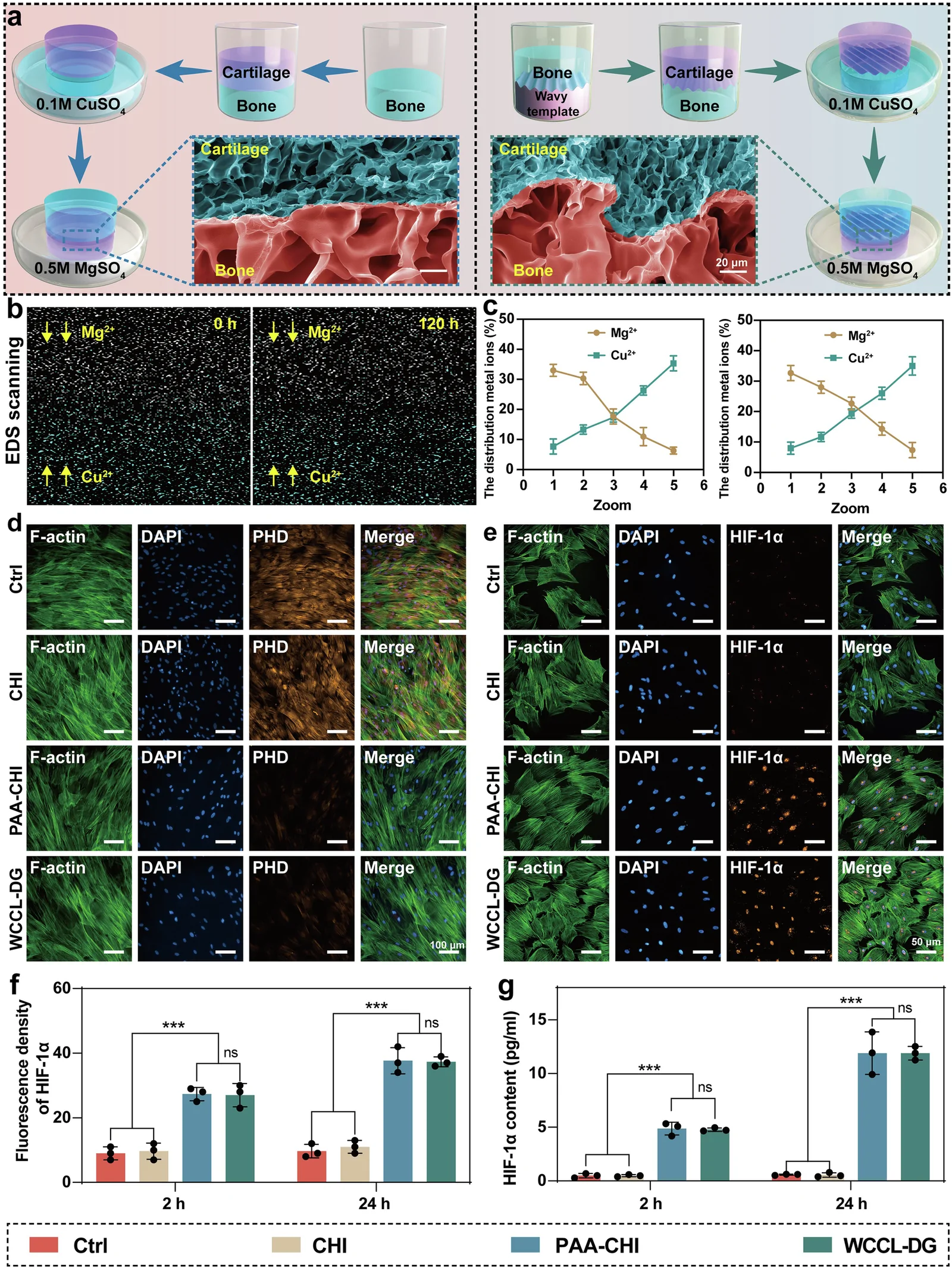
Design and hypoxia-inducible capacity of WCCL-DG scaffold. **a** Preparation process of FCCL-DG and WCCL-DG scaffolds with structural variance at the junctions. **b** EDS mapping of elemental distributions recorded from the cross section. **c** Quantification of Mg^2+^ and Cu^2+^ contents along the vertical direction, where the x axis indicated the distance from the upper to the bottom. **d**, **e** Immunofluorescence staining images of PHD and HIF-1α in BMSCs co-cultured with CHI, PAA-CHI, and WCCL-DG scaffolds at 24 h. **f** Quantitative analysis of intensity of HIF-1α at specific times. (***p = 0.0001, ***p = 0.0002, ***p < 0.0001, ***p < 0.0001, *n* = 3 independent samples). **g** ELISA analysis of the relative content of HIF-1α in BMSCs co-cultured with CHI, PAA-CHI, and WCCL-DG scaffolds. (***p < 0.0001, ***p < 0.0001, ***p < 0.0001, ***p < 0.0001, n = 3 independent samples). Data in **c**, **f**, **g** were presented as means ± SD. Statistical significance was determined using the one-way ANOVA with Tukey’s post-hoc test. Source data are provided as a Source Data file.

The wavy tidemark in natural osteochondral tissue serves as a transitional structure between the cartilage layer and the subchondral bone layer, playing a crucial role in the stress buffering, load transfer, and order arrangement of the extracellular matrix^43^. To systematically evaluate its biomechanical optimization for the stress transmission, a biomimetic wavy CCL interface similar to the tidemark structure between the cartilage layer (PAA-CHI-KGN-Mg^2+^ hydrogel) and subchondral bone layer (PAA-CHI-Cu^2+^ hydrogel) was feasibly designed and customized by integrating the innovative template design, aforementioned gelation process, and subsequent bidirectional soaking strategy. Using a wavy template strategy, the biomimetic subchondral bone layer of PAA-CHI hydrogel was pre-prepared by UV-triggered polymerization of AA with and CHI for a short time. After further simple inversion, another biomimetic cartilage components containing AA monomer and CHI-KGN were poured onto the wavy surface of PAA-CHI hydrogel followed by continued UV irradiation, thus simply yielding a heterogeneous hydrogel. Furthermore, the upper and lower layers of this hydrogel was successively soaked in 0.5 M MgSO_4_ and 0.1 M CuSO_4_ solutions by precise control of the immersion height and time to adjust their respective pore size gradient at the junction, ultimately yielding the targeted WCCL-DG scaffold. For a more effective comparison, we also prepared another kind of sandwiched DN hydrogel (FCCL-DG) with the same composition but a flat interface morphology as experimental controls (Fig. 3a). Due to the variety of hydrogel scaffolds, we have summarized their naming rules and characteristics Supplementary Table 1.

To determine the ion distribution inside the hydrogel, the longitudinal section of sandwiched hydrogel was scanned by EDS, which showed that Mg^2+^ and Cu^2+^ exhibited obvious regional differential distributions, with Mg^2+^ mainly distributed in the cartilage layer and Cu^2+^ mainly distributed in the subchondral bone layer (Fig. 3b). The quantitative ion trends further verified that content distributions of Mg^2+^ and Cu^2+^ did not change significantly over time, indicating that a stable biochemical gradient was established through stepwise bidirectional soaking strategy (Fig. 3c). The reason why these two metal ions were stably distributed within the WCCL-DG scaffold without significant free diffusion was mainly attributed to the higher excessive molar ratio of carboxyl groups/metal ions and formation of tightly ionic or chelation interactions. So, instead of dependence on those expensive or complex equipment to construct multilayered scaffolds, this entire fabrication process was convenient to operate and easy to implement, thereby making it high adaptable efficiency and low manufacture costs in the laboratory and broader application scenarios.

To verify the in vivo biocompatibility and degradability of different scaffolds, we conducted subcutaneous implantation tests in rats (Supplementary Fig. 18). Since WCCL-DG and FCCL-DG hydrogels only differed in structure, we selected WCCL-DG hydrogel for further investigation. After subcutaneous implantation for one month, we did not observe any obvious tissue damage or morphological changes, and the various indicators showed no significant differences compared to those of normal rats (Supplementary Fig. 19), indicating good biocompatibility of those scaffolds. For the in vivo degradation behavior of WCCL-DG and NCCL-CG, both hydrogel scaffolds exhibited a measurable weight gain attributed to their swelling response in the physiological microenvironment during the first week post-implantation. Subsequently, they exhibited a trend of continuous degradation, manifested as a gradual decrease in weight and a corresponding reduction in volume during the subsequent observation period (Supplementary Fig. 20). Interestingly, we found that the degradation rate of the hydrogel scaffold in the joint cavity was faster compared to the subcutaneous degradation. This was because the encapsulation effect of the fibrous capsule after subcutaneous implantation inevitably slowed down the degradation rate of the hydrogel scaffolds. Additionally, there were many degradable enzymes (such as collagenase and matrix metalloproteinase) within the joint, which further accelerated the decomposition of the hydrogel scaffolds. More importantly, in order to further demonstrate the biological function of the wavy CCL, we encouraged the rabbits to actively engage in running and jumping exercises after the surgery. This would exert pressure on the hydrogel scaffold, further accelerating its degradation process.

Microenvironmental factors, such as oxygen, are effective regulators of cell behavior, and HIF-1α is a key effector of low oxygen sensing during cell adaptation^44^. Fe^3+^, as an essential cofactor for maintaining the activity of prolyl hydroxylase (PHD), plays a crucial role in the regulation of HIF-1α degradation^45^. Since chelating Fe^3+^ can effectively inhibit the activity of PHD to stabilize the expression of HIF-1α^27^, the designed PAA-incorporated hydrogels were endowed with intrinsic ability to chelate iron ions by strong carboxyl-Fe^3+^ coordination interactions. In addition, taking consideration into the relatively low concentration of the divalent metal ion solution and the short soaking time, the iron ion chelating ability of WCCL-DG scaffold did not show significant changes (Supplementary Fig. 21). As expected, after co-culture with PAA-CHI and WCCL-DG hydrogel scaffolds, PHD expression in BMSCs was significantly reduced, with levels markedly lower than that of the Ctrl and CHI groups (Fig. 3d and Supplementary Fig. 22). This reduction led to a higher HIF-1α expression in the PAA-CHI and WCCL-DG groups (Fig. 3e–g and Supplementary Fig. 23). Concomitantly, downstream targets of HIF-1α, including stromal cell-derived factor 1α (*SDF1A*) and vascular endothelial growth factor (*VEGF*), were upregulated in these PAA-based hydrogels (Supplementary Fig. 24). These findings indicated that the WCCL-DG scaffold have hypoxia-inducible capacity to promote chondrogenesis.

### In vitro chondrogenesis of biomimetic cartilage-layered PAA-CHI-KGN-Mg^2+^ hydrogel

**Fig. 4:**
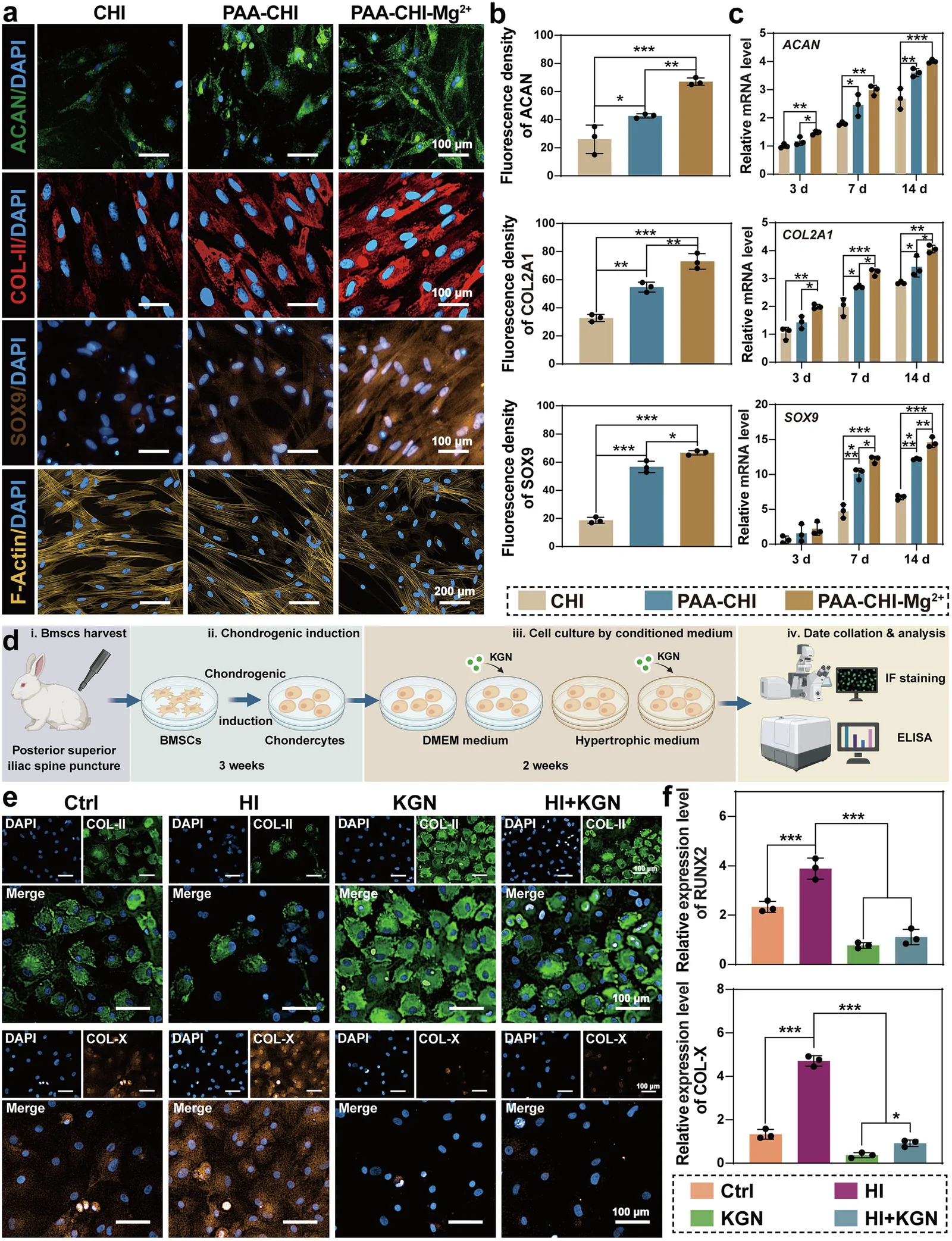
Synergistic effect of PAA-CHI-KGN-Mg^2+^ hydrogel for the chondrogenic differentiation and inhibition of chondrocytes hypertrophy. **a** IF staining of chondrogenesis markers (ACAN, COL2A1, SOX9) and F-Actin after co-culture of BMSCs with CHI, PAA-CHI, and PAA-CHI-Mg^2+^ hydrogels for 14 days. **b** Quantification of IF intensity in **a**. (for ACAN: *p = 0.0361, **p = 0.0067, ***p = 0.0004; for COL2A1: **p = 0.0014, **p = 0.0036, ***p < 0.0001; for SOX9: ***p < 0.0001, *p = 0.0106, ***p < 0.0001, *n* = 3 inde*p*endent samples). **c** Relative mRNA expression of chondrogenic differentiation-related indicators of BMSCs co-cultured with different hydrogels for 3, 7, and 14 days. (for *ACAN*: *p = 0.0240, **p = 0.0028, *p = 0.0381, **p = 0.0026, **p = 0.0062, **p = 0.0010; for *COL2A1*: *p = 0.0233, **p = 0.0018, *p = 0.0102, *p = 0.0424, ***p = 0.0006, *p = 0.0474, *p = 0.0303, **p = 0.0014; for *SOX9*: ***p = 0.0003, *p = 0.0411, ***p < 0.0001, ***p < 0.0001, **p = 0.0018, ***p < 0.0001, *n* = 3 independent samples). **d** Schematic re*p*resentation of conditional culture of BMSCs. Created in BioRender. Ke, L. (2026) https://BioRender.com/zeelqqr. **e** IF staining of COL2A1 and hypertrophy markers COL10A1 on cells after conditioned treatments. **f** ELISA analysis of the relative content of RUNX2 and COL10A1. (***p = 0.0009, ***p < 0.0001, ***p < 0.0001, ***p < 0.0001, ***p < 0.0001, ***p < 0.0001, *p = 0.0298, n = 3 independent samples). Data in **b**, **c**, **f** were presented as means ± SD. Statistical significance was determined using the one-way ANOVA with Tukey’s post-hoc test. Source data are provided as a Source Data file.

To evaluate the effect of WCCL-DG scaffold on the heterogeneous differentiation of BMSCs, we independently evaluated the in vitro differentiation ability of each layer. As shown in supplementary Fig. 25, the chondrogenesis of cartilage-layered hydrogel was firstly assessed using a co-culture system. Compared with the CHI group, the immunofluorescence (IF) staining intensity of key cartilage formation markers (ACAN, COL2A1 and SOX9) in the PAA-CHI group was significantly enhanced, along with the obvious morphology change from spindle-shaped stem cells to round chondrocytes by the cytoskeleton staining (Fig. 4a,b). It further verified its strong chelating ability and hypoxia-inducible capacity to promote the chondrogenic differentiation of BMSCs. When the chondro-proliferative Mg^2+^ was incorporated to the hydrogel scaffolds, a higher expression of chondrogenesis markers were observed, demonstrating the bioactive effect of Mg^2+^ on the chondrogenic differentiation^31^. qRT-PCR further confirmed that the chondrogenic markers in the CHI, PAA-CHI, and PAA-CHI-Mg^2+^ groups were upregulated successively (Fig. 4c). These above results revealed the synergistic effect of bioactive Mg^2+^ and HIF-1α on the promotion of chondrogenesis for cartilage-layered hydrogel.

It is well-known that HIF-1α can also promote the chondrocytes transition from the proliferation stage to the hypertrophy stage by promoting the secretion of VEGF^46^. Hypertrophic chondrocytes provide the basis for the mineralization of cartilage matrix and subsequent formation of bone tissue by increasing cell volume, secreting a mineralized matrix and synthesizing type X collagen (COL10A1)^47^. So, during the process of hyaline cartilage regeneration, it became particularly important to promptly suppress the excessive chondrocytes hypertrophy as needed. To control this process, we introduced a bioactive KGN drug and conducted verification in vitro (Fig. 4d). IF staining results showed that KGN significantly promoted the expression of COL2A1 and inhibited the expression of COL10A1. The fluorescence intensity of COL2A1 in the hypertrophy induction (HI) + KGN group was higher than that in the control group while the fluorescence intensity of COL10A1 was lower than that in the control group, indicating that the inhibitory effect of KGN on chondrocytes hypertrophy was stronger than that of the HI treatment (Fig. 4e and Supplementary Fig. 26). In addition, RUNX2, as a key gene that induces the transformation of hypertrophic chondrocytes into osteoblasts, plays an important role in ECO process^48^. Enzyme-linked immunosorbent assay (ELISA) analysis in Fig. 4f further validated that KGN could suppress the expression of RUNX2 and COL10A1 in chondrocytes, thereby stabilizing chondrocytes in the hyaline phenotype and avoiding chondrocytes hypertrophy.

### In vitro vasculogenesis of biomimetic subchondral bone-layered PAA-CHI-Cu^2+^ hydrogel

**Fig. 5:**
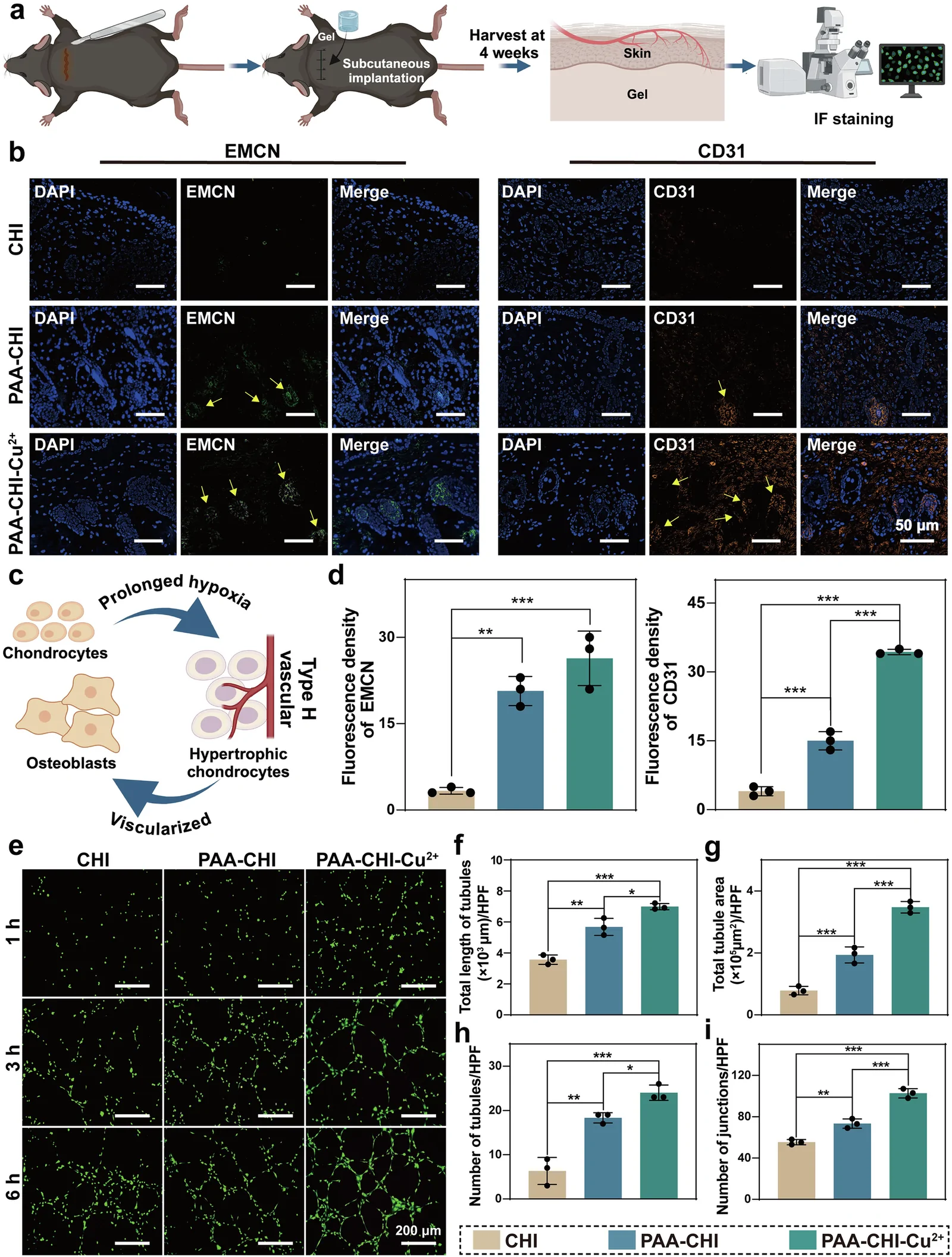
Synergistic effect of PAA-CHI-Cu^2+^ hydrogel for vascularization and following in situ endochondral ossification. **a** Schematic of subcutaneous implantation of CHI, PAA-CHI and PAA-CHI-Cu^2+^ hydrogels in mice. Created in BioRender. Ke, L. (2026) https://BioRender.com/7oc77n8. **b** IF staining of H-type vessel markers in various groups. **c** Schematic illustration of the ECO process. Created in BioRender. Ke, L. (2026) https://BioRender.com/7oc77n8. **d** Quantification of IF intensity in **b**. (**p = 0.0012, ***p = 0.0002, ***p = 0.0001, ***p < 0.0001, ***p < 0.0001, *n* = 3 independent samples). **e** Live cell staining of HUVECs after co-culture with various hydrogels for 1, 3, and 6 h. **f–i** Statistical analysis of the total length of tubules, total tubules area, number of tubules and junctions in high-magnification field at 6 h. (for total length of tubules: **p = 0.0012, ***p < 0.0001, *p = 0.0132; for total tubules area: ***p = 0.0010, ***p = 0.0002, ***p < 0.0001; for number of tubules: **p = 0.0011, *p = 0.0400, ***p = 0.0001; for number of junctions: **p = 0.0034, ***p = 0.0002, ***p < 0.0001, n = 3 independent samples). Data in **d**,** f–i** were presented as means ± SD. Statistical significance was determined using the one-way ANOVA with Tukey’s post-hoc test. Source data are provided as a Source Data file.

In the subchondral bone layer, differentiated chondrocytes undergo a hypertrophic transition due to the lack of KGN regulation, thereby accelerating the progress of ECO via sustainable and stable expression of HIF-1α (Fig. 5c). Meanwhile, the bioactive Cu^2+^ could also work together with HIF-1α to promote the formation of new blood vessels, and the transition process of hypertrophic cartilage into bone could be expediently promoted, collectively providing necessary nutritional support and crucial prerequisite conditions for the subsequent bone tissue reconstruction^49^. Given the fact that HIF-1α is a key promoting factor for the formation of H-type blood vessels, we investigated the vasculogenic ability of PAA-CHI-Cu^2+^ hydrogel through a mouse subcutaneous implantation (Fig. 5a). After 4 weeks, the IF intensity of H-type vessel markers (EMCN and CD31) in the PAA-CHI group was significantly higher than that in the CHI group but lower than that in the PAA-CHI-Cu^2+^ group (Fig. 5b,d). Moreover, after co-culture different hydrogels with HUVECs on Matrigel, the vasculogenesis of CHI, PAA-CHI and PAA-CHI-Cu^2+^ groups were increased successively (Fig. 5e). Furthermore, quantitative analysis of vasculogenesis-related indicators such as tubules and connections also verified that Cu^2+^ could enhance the vascularization (Fig. 5f–i), thus supporting the ECO process and accelerating the osteogenesis in the subchondral bone layer.

On the basis of persistent hypoxia-inducible capacity in situ by the PAA-incorporated WCCL-DG scaffolds and divisional regulation of chondrocytes destination using small molecule KGN, it was particularly simple to manipulate the heterogeneous differentiation and destination of BMSCs to promote osteochondral repair using such a decoupling strategy. More specifically, KGN inhibited the hypertrophy of chondrocytes and maintained the hyaline cartilage phenotype in the cartilage layer. In contrast, due to the absence of KGN intervene in the subchondral bone layer, chondrocytes were prone to hypertrophy and enter the ossification process. Consequently, it was reasonably predicted that the WCCL-DG scaffold could effectively regulate the differentiation direction of chondrocytes at each layer and achieve the heterogeneity regeneration with concurrent processes of cartilage anti-hypertrophy and bone pro-vascularization.

### Effect of various shaped CCL interfaces on biomechanical transmission

**Fig. 6:**
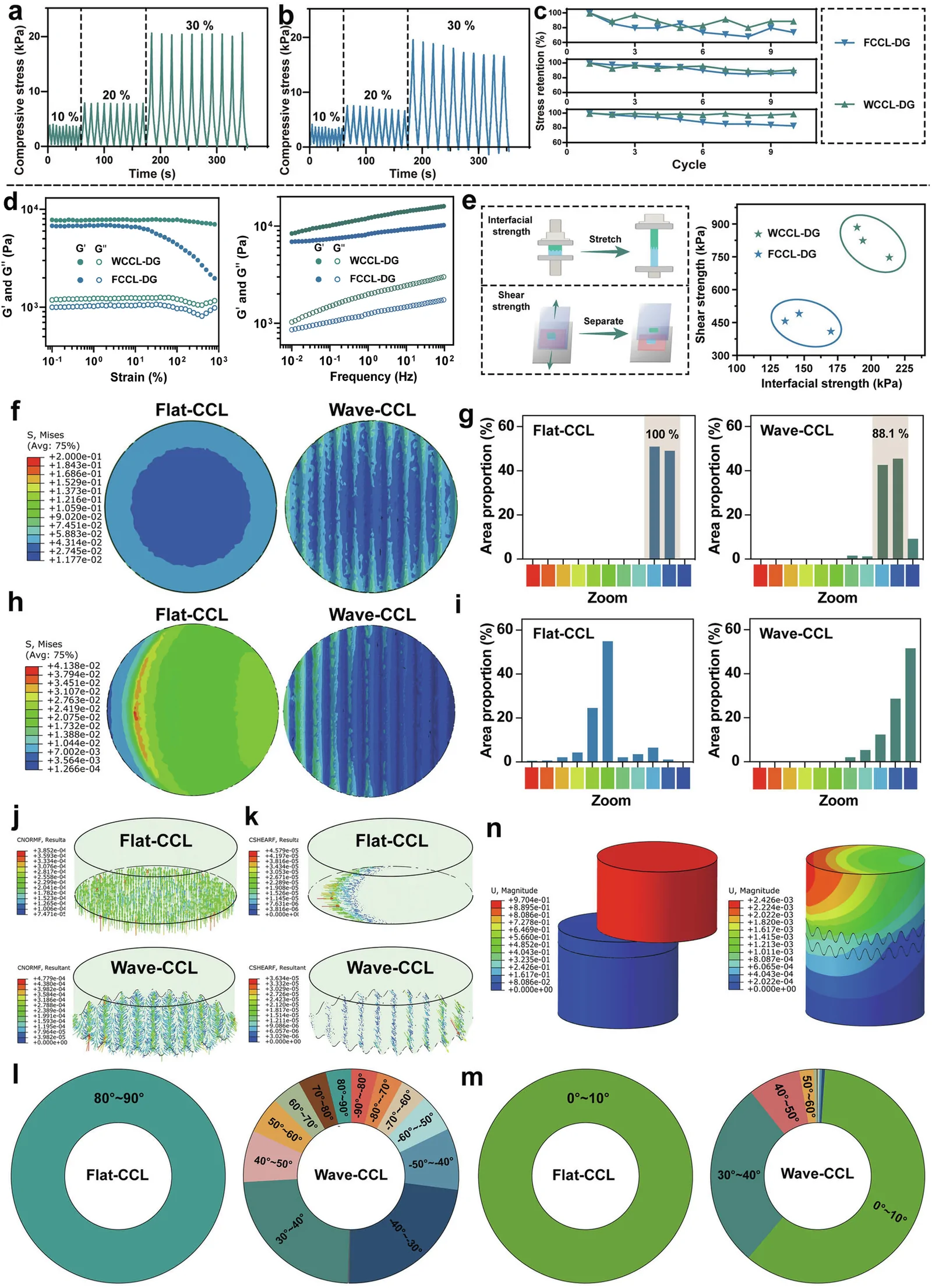
The mechanical differences between WCCL-DG and FCCL-DG hydrogels. **a** Cyclic compression curves of WCCL-DG hydrogel under different strains. **b** Cyclic compression curves of FCCL-DG hydrogel under different strains. **c** Stress retention conditions of WCCL-DG and FCCL-DG hydrogels during the cyclic compression process. **d** Rheological properties of WCCL-DG and FCCL-DG hydrogels. **e** Stretch and shear properties of WCCL-DG and FCCL-DG hydrogels. Created in BioRender. Ke, L. (2026) https://BioRender.com/95hywjt. **f** Thermograms of stress distribution on the lower surface of the cartilage layer when two models were subjected to a compressive stress of 0.1 MPs respectively. **g** Statistical analysis of the proportion of area occupied by different color blocks in **f**. **h** Thermograms of stress distribution on the lower surface of the cartilage layer when two models were subjected to a shear stress of 0.1 MPs respectively. **i** Statistical analysis of the proportion of area occupied by different color blocks in **h**. **j** Direction of stress in the lower surface of the cartilage layer when subjected to compressive stress in two models. **k** Direction of stress in the lower surface of the cartilage layer when subjected to shear stress in two models. **l** Statistical analysis of stress of different ranges in **j**. **m** Statistical analysis of stress of different ranges in **k**. **n** Thermal map of overall displacement under shear stress for two models. Source data are provided as a Source Data file.

The weak mechanical stability of the scaffold and the low efficiency of stress transmission are the main reasons for the failure of osteochondral regeneration^50,51^. Mechanical stability can maintain the structural integrity of the scaffold to resist deformation while the energy dissipation mechanism can buffer the stress to reduce the damage caused by stress concentration^52,53^. The CCL, as a natural transitional interface between cartilage and bone tissues, its geometric shape is crucial for regulating the mechanical properties of the scaffold. To better mimic the dynamic compressive environment within joints, we used multi-dimensional experiments to characterize the effect of CCL with distinct geometries on the mechanical properties of the scaffolds. In the cyclic compression test, WCCL-DG hydrogel maintained excellent repeatability during 30 test cycles under different strains (10%, 20%, and 30%), indicating its mechanical stability and fatigue resistance (Fig. 6a,c). This advantage stemmed from the wavy interface geometry of the WCCL-DG hydrogel, and its uneven peak-valley structures on its surface itself contributed to the elasticity of restoring to the original state after compression. In contrast, the peak stress of the FCCL-DG hydrogel gradually decreased because its flat interface lacked this geometric deformation recovery capability, resulting in cumulative structural deformation (Fig. 6b,c). This rebound characteristic mediated by the geometric structure simulated the dynamic mechanical response of the natural articular cartilage, supporting the continuous structural integrity of the regenerated tissues under physiological cyclic loads^54^. In the strain sweep experiment, the G’ and G” of the WCCL-DG were significantly higher than those of the FCCL-DG, and the linear viscoelastic region of the WCCL-DG was also wider (Fig. 6d). This was because the wavy interface expanded contact area and improved interlocking effect between interfaces through its geometric characteristics, thereby improving the mechanical property and integral stability. The frequency sweep results displayed that the modulus of the WCCL-DG gradually increased with the increasing frequency, especially in the high-frequency region. This was also attributed to its unique geometric shape that could effectively transfer and disperse the stress, thus displaying elastic recovery capacity under high-frequency vibration. Furthermore, wavy CCL could optimize dynamic response, reduce stress concentration, and enhance internal friction characteristics, so G” also showed a greater increase in the high-frequency range. It was worth noting that, due to its wavy structure, it was more prone to internal friction and intermolecular sliding under external stress, which led to the greater energy consumption and thereby increased the overall cohesive viscosity (Supplementary Fig. 27). For interface stability, we characterized the interfacial bonding strength in horizontal and vertical directions using shear and stretch tests (Fig. 6e). The Ashby plot clearly contrasted the shear and stretch resistance of the WCCL-DG and the FCCL-DG hydrogel scaffolds, demonstrating that WCCL-DG outperforms FCCL-DG in both stretch and shear resistance. In terms of stretch resistance, the wave interface increased the interlayer contact area and enhanced the interlayer bonding, thus improving the structural integrity under stretch loads. In terms of shear strength, the wave structure had created a mechanical interlocking effect, partially preventing the detachment of the cartilage layer induced by lateral shear stress, which was more significant in enhancing the overall shear resistance of the hydrogel scaffold compared to its stretch strength. Consequently, these above findings convincingly highlighted the role of wavy structure in effective energy dissipation and excellent mechanical properties, which could alleviate the stress concentration and enhance the stability, elastic recovery, and integrity of sandwich engineering scaffold, enabling it to better adapt to the mechanical environment inside the joint.

The natural thin CCL possesses significant biomechanical functions, but due to its close integration with the subchondral bone and the difficulty in separation, the in vitro biomechanical measurement becomes extremely challenging^55^. To evaluate the performance of wave-shaped CCL (Wave-CCL) in simulating the biomechanical functions of natural osteochondral structure, we used FEA to simulate the actual implantation of the scaffold at the defect site (Supplementary Fig. 28) and study its mechanical response under two representatively compressive and shear stresses (Supplementary Fig. 29). Additionally, to investigate the influence of different shapes of CCL on cartilage regeneration, we analyzed the lower surface of the cartilage layer including the magnitude and direction of the stress and the maximum principal stress distribution, and compared it with the flat-shaped CCL (Flat-CCL, Supplementary Fig. 30). As shown in Fig. 6f,g, when a compressive stress of 0.1 MPa was applied, the ratio of the stress on the lower surface of the cartilage layer in the Flat-CCL model reached 100% within the range of 2.745–5.883 × 10^−2 ^MPa. In contrast, in the Wave-CCL model, only 88.1% of the stress fell within this range, and even 9.1% of the stress was found in the range of 1.177–2.745 × 10^−2 ^MPa. This indicated that the wavy structure effectively reduced the stress on the cartilage layer and alleviated stress concentration. In addition, we analyzed the stress direction on the cartilage layer under compression for two models. As shown in Fig. 6j,l, the stress direction in the Flat-CCL model mainly fell within the range of 80-90°, presenting a single mechanical response. However, Wave-CCL model showed a multidirectional stress mode, in which the reaction stress perpendicular to the wavy structure effectively overcame the limitation of a single stress direction on the cells in the cartilage layer. This multidirectional stress mode not only reduced local stress concentration but also effectively promoted the active response and regeneration of chondrocytes^56^, ultimately optimizing the cartilage repair process.

When both models were subjected to a lateral shear stress of 0.1 MPa, the cartilage layer in the Wave-CCL model was firmly fixed on the subchondral bone layer due to the mechanical interlocking effect mediated by the wavy structure, while the cartilage layer in the Flat-CCL model showed obvious misalignment and probable detachment (Fig. 6n). Quantitatively, the shear stress on the cartilage layer in the Wave-CCL model was significantly lower than that in the Flat-CCL model (Fig. 6h,i), which ascribed to that shear stress in the Wave-CCL model was mainly borne by the subchondral bone layer, as shown in the diagram of the maximum principal stress distribution. In contrast, in the Flat-CCL model, the shear stress was mainly concentrated at the interface (Supplementary Fig. 31). So, its cartilage layer was prone to peeling off when exposed to intense shear stress or complex biomechanical environments in vivo. This was consistent with the aforementioned rheological behavior of the WCCL-DG scaffold that could endure the greater shear stress and maintain the structural stability in the high-frequency range, compared to that of the FCCL-DG scaffold. Meanwhile, in addition to enhancing the shear resistance of cartilage layer and maintaining the interfacial stability at the junction, the shear stress exerted by the subchondral layer further promoted the osteoblasts and subchondral bone regeneration, especially exposure onto the repetitious shear stimulation in vivo. Additionally, under the action of shear stress, Wave-CCL model also exhibited a multidirectional stress mode compared to the single mechanical response of Flat-CCL model, which could also promote the growth and development of chondrocytes (Fig. 6k,m).

To investigate the influence of geometric parameters on stress distribution and cellular behavior, four additional models (M2-M5) were also constructed based on the original model (M1) by independently modulating the wavelength and amplitude of the wavy interface (Supplementary Fig. 32a). After applying a compressive stress of 0.1 MPa, the mechanical response of the cartilage layer varies significantly with changes in geometric parameters of the interface. Specifically, as the wavelength shortens, the local stress on the cartilage layer increased, with the M2 showing the most obvious stress concentration phenomenon (Supplementary Fig. 32b,c). Meanwhile, the increase in wave amplitude also contributed to the enhancement of stress intensity to a certain extent. Additionally, quantitative analysis of the stress direction indicated that although the M2 and M4 still maintained a multi-directional stress distribution pattern, the proportion differences between different intervals were too large. This highly unbalanced mechanical stimulation to some extent restricted the coordinated development of cartilage tissue (Supplementary Fig. 32d,e). In contrast, the M3 and M5 exhibited a stress direction distribution highly similar to that of the original M1, providing a more optimized mechanical microenvironment.

In terms of biological evaluation, we fabricated five groups of osteochondral scaffolds using the corresponding biomimetic templates (Supplementary Fig. 33a) and investigated the biological responses of BMSCs under dynamic mechanical stimulation (Supplementary Fig. 33b). The qRT-PCR and IF staining results showed that compared to the flat scaffold, the wavy interface scaffolds significantly promoted the secretion of more *COL2A1* by BMSCs, once again confirming the superiority of the biomimetic wavy structure (Supplementary Fig. 33c,d). However, the chondrogenic differentiation levels of the M2 and M4 groups were overall lower than those of the M1, M3, and M5 groups. This result was highly consistent with the FEA prediction, indicating that the uneven distribution of stress directions weakened the chondrogenic efficacy of the scaffolds. These results indicate that when designing bionic osteochondral scaffolds, the optimization of the parameters of the wavey structure is of crucial importance. Excessively high amplitudes or overly short wavelengths will lead to stress concentration and imbalance in the distribution of stress, thereby hindering the regeneration of high-quality cartilage.

### In vivo therapeutic effects of osteochondral defects

**Fig. 7:**
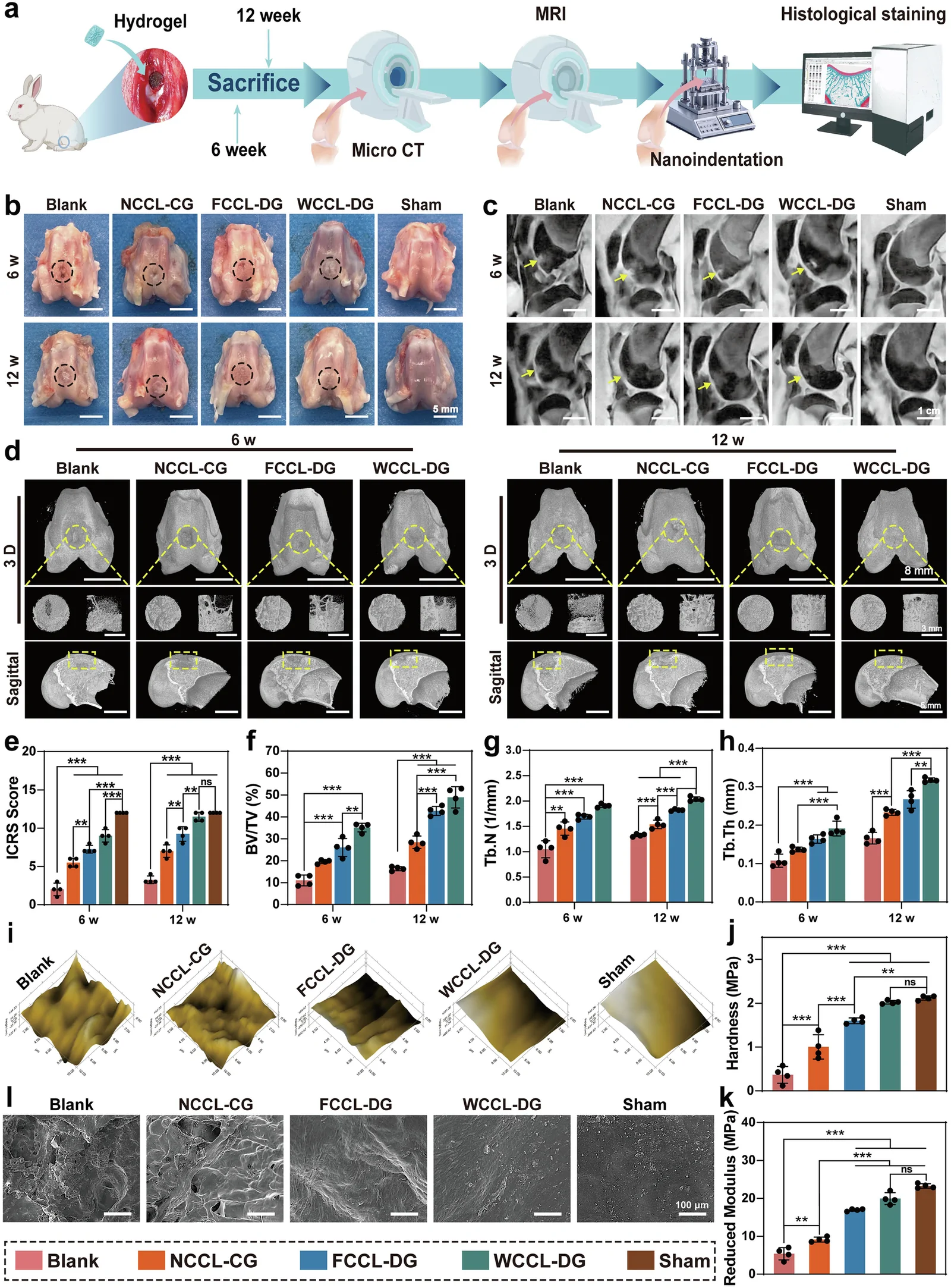
In vivo osteochondral repair effects. **a** The overall processes of animal experiments. **b–d** Representative images of macroscopic observations, MRI and micro-CT of repair effects in blank, NCCL-CG, FCCL-DG, WCCL-DG and Sham groups at 6 and 12 weeks. **e** ICRS histological scoring of the generated tissues in different groups. (**p = 0.0088, ***p < 0.0001, ***p < 0.0001, ***p < 0.0001, **p = 0.0017, **p = 0.0017, ^ns^*p* = 0.8167, ***p < 0.0001, n = 4 independent sam*p*les). **f–h** Quantified subchondral bone microstructural parameters. (for BV/TV: ***p < 0.0001, **p = 0.0018, ***p < 0.0001, ***p = 0.0001, ***p < 0.0001, ***p = 0.0006, ***p < 0.0001; for Tb. N: **p = 0.0010, ***p < 0.0001, ***p = 0.0003, ***p < 0.0001, ***p = 0.0002; for Tb. Th: ***p = 0.0008, ***p = 0.0006, ***p < 0.0001, ***p = 0.0001, **p = 0.0017, ***p < 0.0001, n = 4 independent samples). **i** Microsco*p*ic surface to*p*ography of regenerated cartilage tissues in different groups by nanoindentation. **j**, **k** Hardness and reduced modulus of regenerated cartilage in different groups. (for hardness: ***p = 0.0003, ***p = 0.0006, **p = 0.0022, ***p < 0.0001; for reduced modulus: **p = 0.0016, ***p < 0.0001, ***p < 0.0001, n = 4 inde*p*endent samples). **l** Surface morphology of regenerated cartilage tissues in different grou*p*s by SEM. Data in **e–h** and **j**, **k** were presented as means ± SD. Statistical significance was determined using the one-way ANOVA with Tukey’s post-hoc test. Source data are provided as a Source Data file.

Having demonstrated the durable hypoxia-inducible capacity in situ, heterogeneous spatiotemporal managements, and biomechanical transmission of this bioactive sandwiched WCCL-DG, we then investigated its in-vivo repair efficacy with using an osteochondral defect model on the rabbit femoral trochlea. The rabbits were sacrificed at 6 and 12 weeks after surgery for the examination of macroscopic observation, magnetic resonance imaging (MRI), micro computed tomography (Micro-CT), nanoindentation, and paraffin sections (Fig. 7a). As shown in Fig. 7b, the osteochondral defects were filled with the fibrous tissue in the blank group, and its surface displayed severe destruction at both 6 and 12 weeks. In contrast, the hydrogel groups exhibited the better healing efficiency owing to the bioactive components and mechanical supports for cell activity and growth. Wherein, the WCCL-DG group showed the best repair effect as early as the 6 weeks, and the defect was completely replaced by cartilage-like tissue at 12 weeks. The regenerated tissue was closely integrated with the surrounding tissues without obvious gaps. According to the macroscopic observation and the scoring system of the International Cartilage Repair Society (ICRS), the repair effect of WCCL-DG group was superior to that of the FCCL-DG and NCCL-CG (Fig. 7e), indicating the importance of wavy interface on the cartilage tissue regeneration.

MRI showed that the blank group had obvious defect areas at 6 weeks, and no significant improvement was observed by the 12 weeks. Although more newborn tissues were filled in the FCCL-DG group, their fusion with the surrounding tissues was relatively poor, and the abnormal high-signal shadows were clearly observed at the subchondral bone. On the contrary, the defect areas of the WCCL-DG group were filled with high-quality regenerated tissues, displaying the smooth surfaces and close integration with the surrounding tissues, analogous to the Sham group (Fig. 7c). Through Micro-CT analysis, newborn bone tissues in the hydrogel groups were arranged in an orderly manner, showing a clear trend of growth along the scaffold direction, while the appeared bone tissues of the blank group were disorderedly distributed and granular. As expected, the repair effect of subchondral bone in the WCCL-DG group was better than those of other hydrogel groups. The sagittal views at 6 and 12 weeks also confirmed that the thicker cancellous bone regeneration induced by the WCCL-DG was significantly better than any other groups (Fig. 7d). Moreover, the evaluation of bone trabecular volume fraction (BV/TV), trabecular number (Tb. N), and trabecular thickness (Tb. Th) also showed a significant improvement in subchondral bone formation in the WCCL-DG group (Fig. 7f–h). Additionally, we further conducted the morphological observations and mechanical assessments on the repaired defect areas by SEM observation and nanoindentation methods. As shown in Fig. 7i,l, the obvious cracks and uneven surfaces could be seen in the blank group and NCCL-CG group, and the FCCL-DG group showed a slightly uneven surface at 12 weeks, which indicated that the presence of the CCL was of great significance for osteochondral repair. In contrast, the WCCL-DG group exhibited a smooth and uniform surface, which was highly analogous to the microstructure of the Sham group. More importantly, nanoindentation results further revealed that the load-displacement curve of the regenerated cartilage in the WCCL-DG group showed the most similar periodic changes to that of the Sham group (Supplementary Fig. 34), and the hardness and modulus of the regenerated cartilage reached 2 ± 0.02 MPa and 20 ± 1.91 MPa, respectively, which were also closest to the normal tissues (Fig. 7j,k). Consequently, these therapeutic outcomes in the WCCL-DG group further validated the optimal integration including durably hypoxic environments via these PAA-based hydrogels, uniquely pathological hypertrophy regulation via KGN agents, feasibly spatiotemporal manipulation using bioactive ions, and attractively biomechanical transmission by biomimetic wavy interface.

**Fig. 8:**
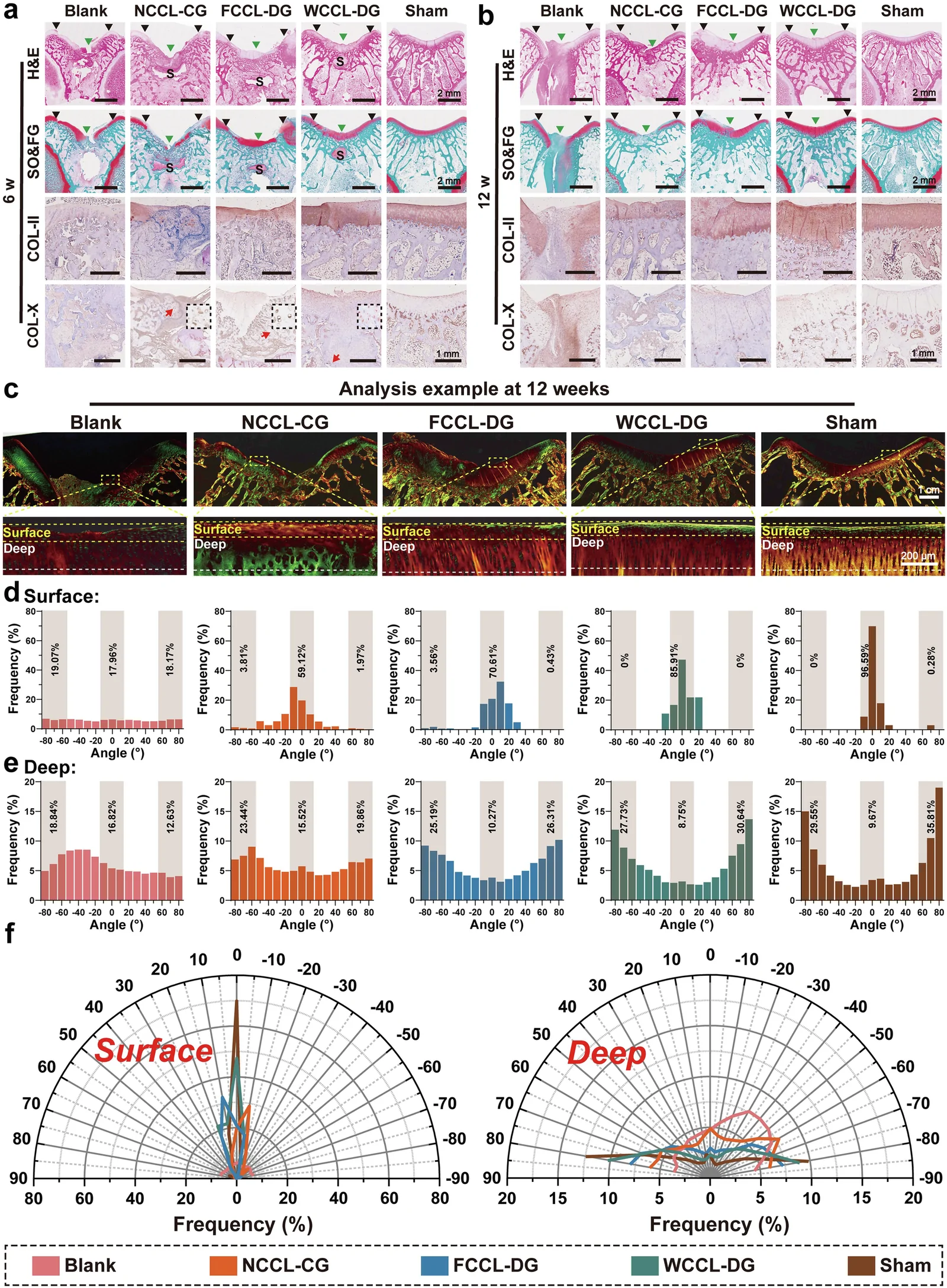
Histological staining and immunological assessment of osteochondral repair. **a**, **b** H&E staining, SO&FG staining, COL2A1, and COL10A1 immunohistochemical staining of regenerated osteochondral tissues in different groups at 6 and 12 weeks. (Black arrow: junction of old and new cartilage; green arrow: regenerative cartilage; red arrow: cells in the hypertrophic cartilage stage; black border: the enlarged areas indicated by the red arrow; S: residual hydrogels). **c** SR staining of regenerated osteochondral tissues in different groups at 12 weeks. **d**–**f** Comprehensive comparison of collagen fiber orientation within the surface and deep zones by polar coordinates.

The tissues at the modeling site were collected at the 6 and 12 weeks and subjected to histological analysis through hematoxylin-eosin (H&E), safranin O-fast green (SO&FG), sirius red (SR) staining, and immunohistochemistry (IHC) to evaluate the repair effect. H&E staining showed that the osteochondral defect persisted with a large cavity in the blank group within 6 weeks, and the repair tissues were fibrous with many disordered structures at 12 weeks, accompanied by inflammatory cell infiltration and poor repair of cartilage and subchondral bone (Fig. 8a,b). SO&FG staining was performed to detect the distribution of proteoglycans in the regenerated tissue. The positive staining in the WCCL-DG group was significantly stronger than that in the other hydrogel groups at both time points. At 12 weeks after surgery, the WCCL-DG group showed a smooth surface comparable to that in the Sham group, while the other groups showed unsatisfactory results, such as insufficient ECM and collagen regeneration (Fig. 8a,b). By comparing the FCCL-DG group with WCCL-DG group, although the volumes of regenerated tissues were similar, the distribution of collagen and matrix in the WCCL-DG group was significantly more uniform, and the cell activity of cartilage layer was also vigorous, which may be attributed to the efficient alleviation of stress concentration and preventing shear stress-induced tissue detachment for protecting cartilage layer.

Wakitani score also confirmed the best repair effect in the WCCL-DG group (Supplementary Fig. 35). The deposition of COL2A1 and COL10A1 in the regenerated tissue was assessed through IHC staining to analyze the tissue repair process. It could be seen that at both time points, the expression levels of COL2A1 in the blank group, NCCL-CG group, FCCL-DG group, and WCCL-DG group showed an increasing trend, and the WCCL-DG group was closest to the Sham group (Fig. 8a,b). IHC and IF staining results also confirmed that the expression of COL2A1 exhibited a distinct functional gradient characteristic. Compared with the blank group, with the introduction of hydrogel implantation, metal ion regulation, and wavy CCL, the expression level of COL2A1 gradually increased. Among them, the expression level of COL2A1 in the WCCL-DG group was close to the normal osteochondral tissue level at 12 weeks (Fig. 8a,b and Supplementary Fig. 36). Furthermore, it was worth noting that the changes in COL10A1 expression reflected the fundamental differences in the repair process. At 6 weeks, except for the sham group, the expression levels of COL10A1 were higher in all experimental groups, providing strong evidence to verify the transition of hypertrophic chondrocytes into mineralized bone tissue and the activity of ECO process for the subchondral bone. However, the high expression in the blank group was closely related to the degeneration of the damaged area, and we speculated that this might be an abnormal expression induced by aging, consistent with the poorer repair effect. In contrast, the hydrogel implantation group showed a normal physiological manifestation of chondrocytes hypertrophy during the process of ECO. At 12 weeks, the expression level of COL10A1 in the hydrogel implantation group gradually decreased as the repair effect improved, indicating that the ions and wavy CCL accelerated the process of ECO. Compared with the blank group, the new bone formation in the hydrogel groups was more extensive without observation of hypertrophic chondrocytes, further demonstrating the completion of the ECO process (Fig. 8a and Supplementary Fig. 36). Furthermore, using the IF co-localization technique, we also marked the tidemark zone in the regenerated tissue (Supplementary Fig. 37). The results showed that the tidemark in the WCCL-DG group were uniform and continuous, with clear and complete structures, which were highly consistent with the tidemark of normal osteochondral tissue. However, the tidemark in the other groups were all fractured or misaligned, and the interface integration was poor, which directly revealed the crucial role of the wavy CCL in promoting the long-term functional integration of osteochondral tissues.

In natural articular cartilage, the collagen fibers in the surface zone were horizontal, while those in the middle and deep cartilage zones were perpendicular to the surface. This heterogeneity and anisotropy pattern played an important role in stress distribution and the transition to subchondral bone^11,57^. We visualized the fiber orientation through SR staining and polarized light microscopy, and analyzed the orientation of the regenerated collagen fibers in two important regions (surface zone and deep zone) using the directionality plugin of Image J software. As shown in Fig. 8c, compared to the blank group, there was obvious collagen distribution in the regenerated cartilage areas of the hydrogel-implantation groups. In the surface zone, the proportion of fibers in the WCCL-DG group within -10-10° reached 85.9%, which was the closest to that of Sham group (96.6%). However, the blank group showed uniform fiber distribution within the range of -80 to 80° without any heterogeneity. This indicated that only fibrous connective tissue covered the defect area. Similarly, in the deep layer, the distribution of collagen fibers in the vertical direction in the WCCL-DG group reached 58.4%, which was also closest to the 65.4% of the Sham group (Fig. 8d–f). So, in views of a collaborative strategy of generalization construction, manipulation heterogeneity, wavy interface biomimetics, and hypoxia-inducible microenvironment regulation, this sandwiched WCCL-DG scaffold embodied full set of functionalities to synergistically achieve the full-thickness osteochondral regeneration.

### Biomechanical mechanisms of wavy interface on promoting cartilage regeneration

**Fig. 9:**
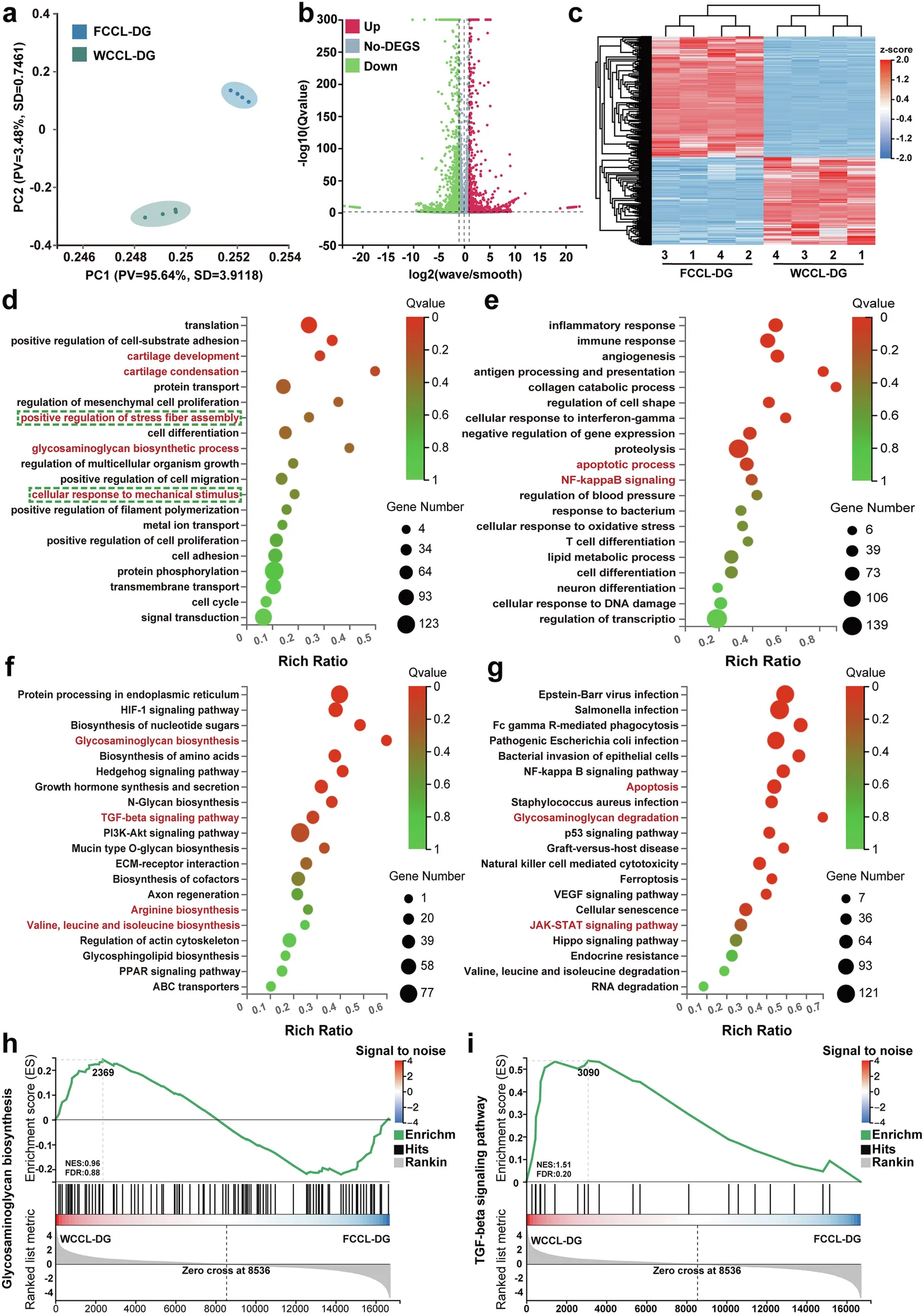
Biomechanical mechanism of wavy interface in promoting cartilage repair. **a**–**c** PCA, volcano map and heat map of differentially expressed genes between the FCCL-DG and the WCCL-DG groups. **d** Upregulated and **e** downregulated terms in enriched GO analysis. **f** Upregulated and **g** downregulated terms in enriched KEGG analysis. **h** Glycosaminoglycan biosynthesis GSEA enrichment analysis. **i)** TGF-β GSEA enrichment analysis.

To explore the molecular mechanism by which the wavy interface enhances chondrogenesis, we performed the RNA sequencing to analyze the differentially expressed genes (DEGs) in the regenerated cartilage samples of WCCL-DG and FCCL-DG groups. Principal component analysis (PCA) of the gene expression profiles showed a small difference within the two groups but a significant separation between the two groups of clusters, indicating significant differences in gene expression (Fig. 9a and Supplementary Fig. 38). Then, 710 DEGs were identified in the cartilage tissues of the two groups (p < 0.05, fold change > 2). As shown in Fig. 9b,c, compared with the FCCL-DG group, 287 genes were upregulated and 423 genes were downregulated in the WCCL-DG group, indicating the significant impact of wavy structure on the cartilage regeneration process.

We further explored the biological functions of DEGs through Gene ontology (GO) and Kyoto encyclopedia of genes and genomes (KEGG) pathway enrichment analyses. As shown in Fig. 9d, GO enrichment analysis revealed that the upregulated differentially expressed genes were mainly involved in the processes of cartilage development, cartilage organization, and glycosaminoglycan biosynthesis, which were closely related to cartilage regeneration. Surprisingly, the upregulation of these genes was also related to stress fiber assembly and the cell response to biomechanical stimulation, indicating that the multidirectional stress mode mediated by the wavy interface promoted cartilage regeneration (Supplementary Fig. 39). Conversely, the downregulated DEGs were related to apoptosis and the NF-kappaB signaling pathway, indicating that the wavy structure reduced apoptosis caused by stress concentration (Fig. 9e). On the other hand, KEGG pathway enrichment analysis showed that the TGF-β signaling pathway, glycosaminoglycan biosynthesis, and amino acid synthesis related to cartilage regeneration were all upregulated (Fig. 9f). In contrast, the apoptosis related to cartilage degradation, glycosaminoglycan degradation, and JAK-STAT signaling pathway were downregulated (Fig. 9g). Additionally, the gene set enrichment analysis (GSEA) further indicated that the glycosaminoglycan synthesis process and TGF-β signaling pathway were activated in the WCCL-DG group (Fig. 9h,i).

## Discussion

The full-thickness defect in osteochondral tissue, a typical interfacial tissue in joints, is hard to be restored after trauma-related injuries or osteoarthritis. The difficulty of osteochondral repair stems from the reconstruction of various multileveled and continuous gradients in connective tissue, which mainly include the gradients in physiochemical compositions (e.g., collagen, inorganic components, and cell types) and biomechanical conditions (e.g., strength, stiffness, and energy dissipation)^58^. So, the replication of complex hierarchical structures and functional integration during the osteochondral regeneration has long been a major challenge in the field of tissue engineering. Here, we introduced a sandwich-layered hydrogel scaffold featuring biomimetic wavy calcified cartilage layer by a synergistic decoupling strategy of bidirectional soaking, spatiotemporal differentiation regulation and interfacial biomimicry. Taking advantage of the full set of functionalities, it effectively solved the key problems regarding the complex preparation, out-of-control signal regulation and stress concentration-induced cartilage damage for most existing engineered scaffolds, thereby providing an innovative biomimetic solution for full-thickness osteochondral regeneration.

This study adopted a bidirectional ion soaking strategy to construct a sandwiched WCCL-DG scaffold with pore and biochemical gradients within a short period of time. Compared with the traditional layer-by-layer assembly technique, this simplified process significantly improved the preparation efficiency and reduced manufacturing costs of multilayered bioactive scaffolds, associated with its self-healing property to avoid the delamination risk at the interface, thereby providing more possibility for large-scale applications in clinic. Current 3D-printed gradient scaffolds always require complex fabrication processes, expensive equipment, and precise parameter control, leading to high manufacturing costs and potential interfacial delamination issues at the interlayer interfaces^59–61^. In contrast, our bidirectional ion soaking strategy simplifies the preparation of multilayered scaffolds and eliminates the risk of interfacial delamination, and stepwise ion soaking can easily achieve continuous gradient formation without the need for expensive instruments. Of note, without incorporating any toxic hypoxia-mimetic agents, PAA itself could inhibit the activity of PHD enzyme by chelating Fe^3+^ and stabilize HIF-1α from degradation, thus creating a long-lasting hypoxic environment in situ throughout the hydrogel scaffold that was critical to facilitate cartilage development. More importantly, by means of the elaborate intervention of chondrocytes metabolism using small molecule KGN, it was highly innovative to regulate the heterogeneous differentiation and destination of BMSCs to promote osteochondral repair using such a decoupling strategy. In the cartilage layer, HIF-1α, Mg^2+^ and KGN jointly promote the chondrogenic differentiation of BMSCs, and the local sustained release of KGN (Supplementary Fig. 14) effectively blocked the chondrocyte hypertrophy process under the long-term action of HIF-1α (Fig. 3e–g and Supplementary Fig. 23), stabilizing the hyaline cartilage phenotype and avoiding the ectopic calcification risk caused by a single hypoxic stimulus. In contrast, within the subchondral bone layer and independently of KGN regulation, the durable HIF-1α expression and Cu^2+^ release synergistically facilitated the vasculogenesis (Fig. 5), prompting hypertrophic chondrocytes into the typical ECO process and ultimately forming vascularized bone tissues (Fig. 8a,b). To our knowledge, this is the frontier report on achieving of homologous initiation-heterogeneous differentiation strategy to implement the heterotypic osteochondral regeneration using a wavy CCL-embedded WCCL-DG scaffold, thereby breaking through the limitation of traditional gradient scaffolds relying on static biochemical signals^62^. Moreover, the addition of Mg^2+^ and Cu^2+^ not only regulated the structural and biochemical gradients of the hydrogel scaffolds, but also endowed them with multiple biological functions beneficial to osteochondral repair (Fig. 3a-c and Supplementary Fig. 15-17).

The wavy tidemark at the natural osteochondral interface is a key biomechanical factor for stress transmission, but its biomimetic structural reconstruction has been merely mentioned in the field of tissue engineering scaffolds so far, let alone a deep investigation to recapitulate its biomechanical function for the cartilage protection and efficient osteochondral regeneration. In this study, a wavy interface was biomimetically introduced into the WCCL-DG scaffold through a simple template design, displaying significant advantages in effectively resisting fatigue, evenly distributing loads, and preventing stress concentration in the cartilage. The cyclic compression results indicated that the introduction of wave-CCL had enabled the scaffold to achieve an anti-fatigue capability of over 90% (Fig. 6a-c). The rheology showed the wider range of linear viscoelastic region with a more stable overall state, and the WCCL-DG could endure the violent shear stress and maintain the structural stability even in the high-frequency range (Fig. 6d), thus allowing for a better adaptation to compression environments within the joint. In addition, FEA revealed that the wavy interface could reduce the stress concentration in the cartilage layer (Fig. 6f-i) and enhance the shear resistance to avoid cartilage detachment (Fig. 6n), and moreover, when subjected to external stress, it could markedly cause the stress direction to shift from the unidirectional mode of the planar structure to a multidirectional mode (Fig. 6j–m), endowing the ability to promote the secretion of cell matrix and cartilage regeneration. Transcriptome analysis further confirmed the biological effects consistent with those of FEA. The results revealed that the multidirectional stress mode mediated by the WCCL-DG promoted the deposition of cartilage matrix by regulating the TGF-β/NF-kB signaling balance, and the stress dispersion alleviated the degradation of cartilage matrix (Fig. 9d–i), thereby restoring the true biological function of the CCL^63^. More importantly, in vivo results confirmed the best therapeutic effect for the WCCL-DG group. The wavy interface significantly enhanced the reduced modulus of the regenerated cartilage (20 ± 1.91 MPa vs 17 ± 0.32 MPa of the FCCL-DG group) by promoting the directional arrangement of collagen fibers, in which the proportion of shallow horizontal arrangement was 85.9% vs 70.6% of the FCCL-DG group, and the proportion of deep vertical arrangement was 58.4% vs 51.3% of the FCCL-DG group, closest to that in the Sham group.

Compared with current state-of-the-art technologies such as cartilage-like modulus degradable hydrogels or gradient scaffolds, our system’s core advantage lies in integrating rarely combined functional elements that address key gaps in these methods. As far as we know, most advanced methods are relatively simple in function and lack integrated regenerative capabilities. For example, gradient scaffolds always rely on the fixed biochemical cues but lack targeted control, and many modulus-matched hydrogels only meet mechanical requirements but neglect the reconstruction of wavy CCL with key mechanical properties, not to mention the regulation of differentiation signals. In contrast, our system unites three critical functions of decoupled spatiotemporal control, biomimetic biomechanical interface, and sequential biological processes, thereby surpassing state-of-the-art approaches. The bidirectional soaking strategy not only constructed a biochemical gradient of Mg^2+^ in the upper layer and Cu^2+^ in the lower layer but also generated the adjustable pore size and modulus gradients, which could simultaneously regulate cell differentiation and structural-mechanical properties of hydrogel scaffolds. Furthermore, the scaffolds possessed inherent iron chelation capabilities to trigger a hypoxic microenvironment by activating HIF-1α. In the cartilage layer, HIF-1α worked in synergy with Mg^2+^ to promote chondrogenic differentiation of BMSCs; while in the subchondral bone layer, HIF-1α worked in synergy with Cu^2+^ to promote vasculogenesis, thereby facilitating the process of ECO and achieving the effect of homologous initiation - heterochronic differentiation. Notably, KGN in the cartilage layer prevented chondrocytes hypertrophy caused by long-term HIF-1α activation, representing a repurposing of KGN beyond its conventional use. Under this circumstance, this decoupling strategy could control HIF-1α expression spatially and temporally, inhibiting hypertrophy in cartilage while promoting ECO in subchondral bone, beyond the existing literatures through integrated innovations that addressed unmet needs in osteochondral repair. Additionally, we innovatively developed a biomimetic wavey CCL, which effectively alleviated stress concentration and enhanced shear resistance, addressing a critical gap in existing scaffolds that overlooked the biomechanical functions of natural CCL. Collectively, we have fabricated a biomimetic wavy CCL interface and reveal its biomechanical transmission and cartilage protection function in addressing the challenges of stress concentration and interfacial detachment via the FEA and transcriptome studies, enabling the best therapeutic effect of full-thickness osteochondral defects.

Nevertheless, our study also revealed some limitations and areas for the future work. First, although the current CCL was fabricated based on a template with a regular wave-like structure, its structural fidelity to the native CCL—characterized by irregular and heterogeneous architecture—remained limited. It was difficult to precisely scan the microscopic structure of rabbit CCL using conventional methods, which limited the reproduction of the authenticity of the biomimetic structures. Further efforts are required to capture the complex, non-uniform morphology of natural CCL through advanced scanning technologies that can precisely map its three-dimensional shape, size, and thickness. Subsequent fabrication via the advanced imaging (e.g., micro-CT) and sophisticated manufacturing technologies (e.g., 3D bioprinting) would enable the development of more biomimetic osteochondral scaffolds with higher structural accuracy and create CCL models with various combinations of amplitude, wavelength, and thickness parameters, better recapitulating the native tissue’s structural complexity and functional properties. Through mechanical tests and FEA, the optimal parameters for stress transmission can be identified, thus providing crucial data support for the clinical translation of this biomimetic scaffold. Second, the present study was conducted using a rabbit femoral trochlea osteochondral defect model, which may not fully recapitulate the anatomical complexity, loading conditions, and pathological microenvironment of human osteochondral defects. Additionally, the current scaffold preparation was relied on a stepwise bidirectional soaking strategy. Since it was difficult to control the soaking depth within the limited height range of the osteochondral defects in the small rabbits, the accuracy was inevitably affected. So, the translation to clinical applications requires potent validation in larger animal models (such as goats and pigs) with more human-like joint structures and functional demands.

In summary, we proposed a multifunctional sandwiched hydrogel scaffold by integrating hypoxia signal regulation, spatiotemporal decoupling, and biomimetic interface design for full-thickness osteochondral regeneration. Its universal preparation method, long-lasting expression of HIF-1α signaling, chondrogenic differentiation intervention, and biomechanical transmission characteristics not only promoted the development of tissue engineering theory, but also laid a technical foundation for the clinical therapy of osteoarthritis and other complex osteochondral defects.

## Methods

### Ethical statement

All animal experiments were approved by the Ethical Committee of Laboratory Animals of Peking University Third Medical School (No. A2019029).

### Materials

Acrylic acid (AA) was purchased from Energy Chemical (Shanghai, China). Short-chain chitosan (CHI, degree of deacetylation> 90%, viscosity 45 mPas for 1% (w/v) solution) was purchased from Jinhu Company (Shanghai, China). Irgacure 2959 (IR2959) and kartogenin (KGN) were purchased from TCI Chemistry (Tokyo). N, N’-Methylenebisacrylamide (MBA) was purchased from J&K Scientific (Beijing, China). Anhydrous ethanol, 1-ethyl-3-(dimethylaminopropyl) carbodiimide hydrochloride (EDC) and N-hydroxysuccinimide (NHS) were purchased from Sinopharm Chemical Reagent Co., Ltd. (Shanghai, China). Ficoll lymphocyte separation medium was purchased from GE Healthcare (Sweden). Rhodamine labeled phalloidin were purchased from Cytoskeleton Inc., Denver (USA). Anti-prolyl hydroxylase (PHD) antibody was purchased from Novus Biologicals (USA). Anti-Aggrecan (ACAN) antibody, Anti-SOX9 antibody and Anti-Collagen II (COL2A1) antibody were purchased from Thermo Fisher Scientific (USA), Absin (Shanghai, China) and Arigo biolaboratories (Shanghai, China). Anti-CD31 antibody and Anti-Endomucin (EMCN) antibody were purchased from Arigo biolaboratories (Shanghai, China) and Bioss (Beijing, China). Goat Anti-Rabbit IgG H&L (Alexa Fluor® 555), Donkey Anti-Mouse IgG H&L (Alexa Fluor® 594) and Donkey Anti-Goat IgG H&L (Alexa Fluor® 594) were purchased from Abcam (UK). C57BL6 mice, New Zealand white rabbits, and Sprague-Dawley (SD) rats were purchased from SiPeiFu Biotechnology Co., Ltd. (SPF; Beijing, China). We used male animals for experiments in order to avoid the influences of sex on experimental results.

### Preparation of PAA, CHI and PAA-CHI hydrogels with different CHI contents

CHI (0.2 g), AA (1.8 g, 3.8 g, 5.8 g), 10 mg/mL MBA aqueous solution (86 μL) and photoinitiator IR2959 (41.6 mg) were added into 8 mL of H_2_O, and the solution was dissolved by shaking in a circular shaker for 10 min. The transparent solutions were UV irradiation for 2 h to obtain a series of PAA-CHI-X hydrogels (X represents the ratio of the mass of the total to that of CHI: PAA-CHI-10, PAA-CHI-20, PAA-CHI-30). The pure PAA hydrogel was obtained by weighing 0.2 g of AA into 8 mL of H_2_O and then subjected to UV irradiation for 2 h. CHI hydrogel was prepared by dissolving 0.2 g of CHI into 8 mL of H_2_O under horseradish peroxidase and hydrogen peroxide conditions. The formed hydrogels were washed to remove unreacted materials and residual byproducts.

### Determination of ion release concentration

The soaking concentration of PAA-CHI-20 hydrogel in MgSO_4_ and CuSO_4_ solutions was determined by the release concentrations of Mg^2+^ and Cu^2+^. After being immersed in solutions of different concentrations for 1 h, the prepared hydrogel was placed in 5 mL of deionized water and incubated in a 37 °C incubator. At the specified time points, the ion concentrations in PBS were measured using Inductively Coupled Plasma Optical Emission Spectrometry (ICP-OES, PlasmaQuant 9100, Germany).

### Synthesis of CHI-KGN conjugate

The small molecular drug of KGN was grafted on the molecular chain of CHI using an amide condensation reaction. Briefly, KGN, EDCI and NHS were added to deionized water in a molar ratio of 1:1.2:1.2 and stirred vigorously for 1 h. After the addition of CHI, the temperature was increased to 50 °C to improve the reactive efficacy for 48 h. Then, the reaction was dialyzed with deionized water for 72 h and further lyophilized to obtain the purified CHI-KGN conjugate.

### Characterization of CHI-KGN conjugate

Nuclear magnetic resonance hydrogen spectrum (^1^H NMR): The samples were performed on a Bruker Avance III 400 HD NMR spectrometer using deuterated water (D_2_O) as solvent. The final data were analyzed using MestReNova software.

Fourier infrared spectroscopy (FT-IR): The samples were lyophilized in a freeze dryer and subsequently ground and pressed with potassium bromide in a quartz mortar and tested using a TENSOR-27 spectrometer (Bruker, Germany). Scanning frequency: 4000–400 cm^-1^.

### Preparation of PAA-CHI-KGN hydrogel

CHI-KGN (0.2 g), PAA (3.8 g), 10 mg/mL MBA aqueous solution (86 μL) and photoinitiator Irgacure 2959 (41.6 mg) was added into 8 mL of H_2_O, and the solution was dissolved by shaking in a circular shaker for 10 min. After 2 h of UV irradiation, a PAA-CHI-KGN hydrogel was obtained.

### Cumulative release of KGN in vitro

To obtain the release behavior of KGN from the PAA-CHI-KGN-Mg^2+^ hydrogels, the samples were placed in 5 mL of phosphate buffer solution (PBS, pH 7.4,) in a 37 °C shaker with continuous agitation. After incubation for a certain time (1 d, 3 d, 5 d, 7 d, 10 d, 14 d, 21 d, 27 d, 35 d, 42 d and 49 d), we aspirated the PBS solution with released KGN, and then added the same volume of fresh PBS solution, keeping the total volume of 5 mL. The concentration of KGN in the PBS solution at different points in time was quantitatively analyzed by a UV-vis spectrophotometer with the absorption peak of KGN at 279 nm^64^. The cumulative release rate was calculated as follows:

1 $$	{{{\rm{Cumulative}}}}\; {{{\rm{release}}}}\; {{{\rm{rate}}}}\; {{{\rm{of}}}}\; {{{\rm{KGN}}}}(\%) \\ 	=({{{\rm{KGN}}}}\; {{{\rm{cumulative}}}}\; {{{\rm{release}}}}/{{{\rm{total}}}}\; {{{\rm{KGN}}}}\; {{{\rm{in}}}}\; {{{\rm{hydrogel}}}}) \times 100\%$$

### Process of template manufacture

The biomimetic wavy templates were fabricated using a three-component vacuum polyurethane resin (DPI 8400). The geometric profile of the interface was designed via Computer-Aided Design (CAD) to mimic the natural tidemark topography of osteochondral tissue. To prepare the templates, a master model was first created using high-precision 3D printing to produce a negative silicone rubber mold. The DPI 8400 resin components were mixed in a specific weight ratio and subjected to vacuum degassing to ensure the removal of all air bubbles. The mixture was then poured into the silicone mold, which had been pre-heated to 60–70 °C to ensure complete curing and optimal dimensional stability. Following polymerization and demolding, the high-precision polyurethane templates were utilized as the structural base for the subsequent layered polymerization of the hydrogel scaffold, ensuring the accurate replication of the biomimetic wavy interface.

### Preparation of sandwiched WCCL-DG scaffold (PAA-CHI-KGN-Mg^2+^/CCL/PAA-CHI-Cu^2+^)

The sandwiched WCCL-DG scaffold was prepared using a template and a soaking strategy. Firstly, the template with wavy upper surface was placed at the bottom of the container, the KGN-free hydrogel stock solution was dropped on the top, and after UV irradiation to form the gel, it was demolded and inverted, and the KGN-containing hydrogel stock solution was dropped and UV irradiated again, to obtain the hydrogel with the structure of the wavy tidemark. Subsequently, the KGN-containing cartilage layer was fully soaked in a 0.5 M MgSO_4_ solution for 1 h. Its height accounted for 45% of the total hydrogel height and the soaking depth was strictly controlled to cover the entire thickness of the cartilage layer. Following this, the subchondral bone layer was fully soaked in a 0.1 M CuSO_4_ solution for 1 h. Its height also accounted for 45% of the total hydrogel height and the soaking depth was ensured to cover the entire thickness of the subchondral bone layer. A sandwiched hydrogel was finally obtained with the height ratio of 45% for the cartilage layer, 10% for the intermediate layer and 45% for the subchondral bone layer. To ensure precise control over the soaking depth, we utilized an XYZ Linear Stage System (Zolix Instruments Co., Ltd.) integrated with a closed-loop control system. This setup, equipped with a high-resolution optical encoder and a servo motor, enables high-precision vertical displacement of the scaffold with a positioning accuracy of ±0.05 mm. By presetting the soaking depth with this mechanical positioning system, we ensured that the liquid-contact height remained strictly consistent across different batches. In addition, we also prepared NCCL-CG and FCCL-DG as controls. Due to the variety of hydrogel scaffolds, we have summarized their naming rules and characteristics in Supplementary Table 1.

### In vivo **hydrogel degradation and biocompatibility**

Eight-week-old SD rats were anesthetized by intraperitoneal injection of 3% pentobarbital sodium. After shaving the back skin and disinfecting it with iodophor, 1-centimeter incisions were made on both sides, and the skin was separated from the fascia. Sterilized hydrogel samples (WCCL-DG, NCCL-CG) were weighed (*W*_0_) and implanted subcutaneously on both sides. The incisions were sutured and then disinfected again. The control group did not undergo hydrogel implantation during the surgery.

After the surgery for one week, three weeks, five weeks, seven weeks and night weeks, hydrogel samples were taken from the back tissues, photographed and weighed (*W*_d_), and the in vivo degradation ratio was evaluated by the following formula:

2 $${{{\rm{Degradation}}}}{{{\rm{ratio}}}}(\%)={W}_{{{{\rm{d}}}}}/{W}_{0}\times 100\%$$

### Porous structure and elemental distribution

The hydrogel samples were freeze-quenched in liquid nitrogen and lyophilized, and the samples were adhered to the sample stage using conductive adhesive. Before testing, the samples were directly sputter-coated with a thin layer of Pt for 120 s to allow the dry sample conductive. Then, the interfacial micromorphology and pore size of the hydrogel were observed using a scanning electron microscope (SEM, JSM-7900F, Hitachi, Japan). The pore size was measured using the software tool in the machine, and the porosity was measured by Image J software.

Sandwiched hydrogel was prepared and placed on the sample stage with the longitudinal section facing upwards, after determining the scanning site of the sample, the elemental distribution was scanned using an energy dispersive spectrometer (EDS, AMETEK, USA).

### Compressive testing

The mechanical properties of the hydrogels were evaluated using an Instron 3365 universal testing machine (Instron Co, USA). Samples with a diameter of 15 mm and height of 10 mm were prepared for compressive testing at a rate of 5 mm/min. The size of the sensor is 8000 N, 3 parallel samples were tested in each group and the average value was taken after testing. The compressive stress and modulus of elasticity were calculated from the stress-strain curves.

The strain recovery ability of WCCL-DG and FCCL-DG scaffolds under the same loading rate was revealed through cyclic compression experiments. The hydrogel was compressed to 10%, 20%, and 30% of its original height, then restored at the same rate, and this process was repeated 10 times for each strain to obtain the stress-strain curve.

### Swelling property

To characterize the swelling ratio, the initial wet weight of dry hydrogel sample was first measured (*W*_d_). Then, the hydrogels were incubated in 20 mL of PBS (pH 7.4) at 37 °C, respectively. After incubation for a predetermined time (0 h, 0.5 h, 1 h, 3 h, 5 h, 7 h, 9 h, 18 h, 36 h, 72 h, 144 h and 216 h), the weight of the swollen samples (*W*_s_) was weighed after the surface water was gently removed with filter paper. The swelling rate was calculated by the following formula:

3 $${{{\rm{Swelling\; ratio}}}}(\%)=({W}_{{{{\rm{s}}}}}-{W}_{{{{\rm{d}}}}})/{W}_{{{{\rm{d}}}}}\times 100\%$$

### Antibacterial activity

The susceptibility of *E. coli* and *S. aureus* to PAA-CHI, PAA-CHI-KGN-Mg^2+^, and PAA-CHI-Cu^2+^ hydrogels were determined by methods similar to the Kirby-Bauer disk diffusion test (Oxoid, United Kingdom)^65^. *E. coli* and *S. aureus* were planted on the Mueller-Hinton agar plate (MH plate) using a cotton swab, respectively, and different kinds of hydrogels were planted on the center of the MH plate. All the groups were incubated at 37 °C with 5% CO_2_ for 48 h. After the culture, the results were calculated and photographed, the approximate diameter of the antibacterial cycle was measured, and the statistical analysis was conducted according to the measured results.

### Cell isolation

The bone marrow mesenchymal stem cells (BMSCs) were isolated from New Zealand white rabbits via posterior superior iliac spine puncture. Bone marrow blood was first extracted from the posterior superior iliac spine of rabbits using a bone marrow aspiration needle, and then diluted 1:1 with PBS (Zhongshan, Beijing, China), and then Ficoll-Paque Lymphocyte Separation Solution (GE Healthcare, USA) was slowly added dropwise to the diluted bone marrow blood. After centrifugation for 30 min at room temperature, the intermediate cell layer was collected, washed with PBS and centrifuged for 15 min. The precipitate was then resuspended and the cells were harvested for further use.

The chondrocytes were harvested from articular cartilage of New Zealand white rabbits via sequential enzymatic digestion. Cartilage slices were first dissected from the femoral condyles and tibial plateaus under sterile conditions, rinsed twice with PBS (Zhongshan, Beijing, China), and then minced into 1 mm^3^ fragments. The fragments were incubated in 0.25% (v/v) trypsin (Invitrogen, USA) at 37 °C for 30 min with gentle agitation, followed by two washes with PBS. Subsequently, the tissue was digested in 0.2 % type II collagenase (Sigma, USA) at 37 °C for 4 h with intermittent shaking. After filtration through a 70 µm cell strainer (BD Falcon, USA), the cell suspension was centrifuged for 10 min. The precipitate was then resuspended and the cells were harvested for further use.

### Cell culture

Cells were inoculated into appropriate petri dishes (Corning, USA) using medium (αMEM or DMEM, Gibco, USA) supplemented with 10% (v/v) fetal bovine serum (FBS, HyClone, USA) and 1% (v/v) penicillin/streptomycin (Gibco, USA). Then cells were cultured in an incubator at 37 °C with 5% CO_2_, and the medium was changed regularly. When cells reached 80-90% confluence, they were passaged using 0.25% (v/v) trypsin (Invitrogen, USA) for amplification. Aseptic techniques were maintained throughout the process to prevent contamination.

### Cytotoxicity

The effect of hydrogels on the viability of BMSCs and chondrocytes were evaluated by quantitative Cell Counting Kit-8 assay (CCK-8, Dojindo Laboratories, Japan) and live/dead staining. Cells were first inoculated in 96-well plates with a cell density of 5 × 10^3^ cells/100 μL per well, and cultured in a 5% CO_2_ and 37 °C incubator for 24 h to allow the cells to spread completely. Subsequently, the original medium was replaced with an extracting liquid obtained by soaking the hydrogels in complete medium for 24 h (0.1 g/mL), and fresh medium was used as a control. The cells continued to be cultured for 1, 3 and 7 days after which the medium was removed from the 96-well plates, 100 μL of fresh medium and 10 μL of CCK-8 reagent were added to each well, and incubation was continued in the incubator for 1 h. Finally, the absorbance at 450 nm was detected by enzyme marker, and 3 replicate wells were set up for each group. It was considered cytotoxic if cell viability was <80% after incubation with extracting liquid of hydrogels.

Live/dead kit (KeyGEN BioTECH, China) was used to observe the survival cells. Cells were cultured using extracting liquid of different hydrogels, and after 7 days, the extracts were aspirated and washed with PBS solutions, and then immersed in calcein-AM (2 μM) and propidium iodide (8 μM) reagents for 30 min at 37 °C. Live (green) and dead (red) cells were detected using confocal microscopy with excitation wavelengths of 488 nm and 545 nm.

### Cell migration

Cell scratch assay and transwell assay were used to evaluate the migration behavior of metal ions and KGN on BMSCs. For the cell scratch, BMSCs were inoculated at a density of 5 × 10^5^ per well and cultured until 100% fusion. The wells were rinsed after scratching and extracting liquid were added to the culture wells. The degree of wound closure was quantified by inverted microscope observation at 6-h intervals and measurement of the slit area using Image J software. The rate of migration was calculated as follows:

4 $${{{\rm{Migration\; rate}}}}(\%)=({S}_{{{{\rm{i}}}}}-{S}_{{{{\rm{r}}}}})/{S}_{{{{\rm{i}}}}}\times 100\%$$

the initial area was marked as *S*_i_ and the residual area was marked as *S*_r_.

For the transwell, 5 × 10^4^ BMSCs were inoculated in the upper chamber, and extracting liquid were added to the lower chamber. After incubation for 48 h, the migrated cells were stained with 1% crystal violet for observation and quantitative analysis.

### Alcian blue staining

Firstly, BMSCs were seeded at a density of 1 × 10^5^ cells per well in a 24-well plate. Then, they were cultured in chondrogenic differentiation medium (GUXMX-90041, Oricell, China) with Mg^2+^ concentrations of 100 ppm, 200 ppm, 300 ppm, and 400 ppm, respectively. The medium was changed every 2 days. After 14 days of induction, the cartilage differentiation was evaluated by Alcian blue staining and subsequent microscopic examination.

### Rheological measurements

The hydrogels were tested using a rotational rheometer ARES-G2 (TA, USA), where the hydrogels were prepared in the form of discs (diameter of 2.5 mm and thickness of 2 mm). The testing temperature was 25 °C. Dynamic strain scan: fixed frequency at 6.28 rad s^-1^ and strain at 0.1–1000% to determine the linear viscoelastic region. Dynamic frequency scan: fixed strain at 1%, frequency at 0.01–100%.

### Iron ion chelating capacity

To verify the adsorption capacity of CHI, PAA-CHI, and WCCL-DG groups for Fe^3+^, we immersed them in 1 M FeCl_3_ solutions, respectively. To ensure the adequate adsorption of metal ions on the hydrogels, stirring was performed for 6 h. Then, the hydrogels and solutions were separated by centrifugation, and the supernatant was collected. The change in the concentration of Fe^3+^ in the supernatant was analyzed by ICP-OES to calculate the amount of Fe^3+^ adsorbed by the hydrogels. The formula is as follows:

5 $$q=({c}_{0}-{c}_{{{{\rm{f}}}}})V/m$$

where *q* was the biosorption capacity (mmol g^−1^); *c*_0_ and *c*_f_ were the concentration of Fe^3+^ solution (mmol mL^−1^) before and after biosorption, respectively; *V* was the initial volume of Fe^3+^ solution (mL) and *m* was the weight of the hydrogels (g).

### Detection of PHD and HIF-1α

A hydrogel-cell co-culture system was established using a transwell experiment. Briefly, hydrogels were placed in the upper chamber and BMSCs were seeded in the lower chamber. After 2 h and 24 h of co-culture, the cells in the lower chamber were subjected to IF staining and ELISA of HIF-1α. For IF staining, samples were rinsed with PBS to remove the culture medium and then fixed with 4% paraformaldehyde for 30 min. Then, 0.1% Triton-X 100, 0.5% bovine serum albumin (BSA, ST2249, Beyotime, China), rabbit anti-HIF-1α primary antibody (1:100, PA1-16601, Thermo Fisher Scientific, USA), and Alexa Fluor® 555 goat anti-rabbit IgG antibody (ab150078, Abcam, UK) were used successively to incubate the samples. For PHD, rabbit anti-PHD primary antibody (1:100, NB100-137, Novus Biologicals, USA) and Alexa Fluor® 555 goat anti-rabbit IgG antibody (1:200, ab150078, Abcam, UK) were used successively to incubate the samples. The cytoskeleton was visualized by staining the cellular samples with Rhodamine-Phalloidine (1:200, C2201S, Beyotime, China). The cell nucleus was stained with DAPI (C1006, Beyotime, China). IF images were obtained using confocal microscopy. Additionally, culture medium was collected at specific time points and HIF-1α expression was assayed using the corresponding ELISA kits, following the manufacturer’s protocols.

### In vitro chondrogenic differentiation of BMSCs by the PAA-CHI-KGN-Mg^2+^ hydrogel

CHI, PAA-CHI, and PAA-CHI-KGN-Mg^2+^ hydrogels were placed in the upper chamber, while BMSCs were seeded in the lower chamber with chondrogenic differentiation medium (GUXMX-90041, Oricell, China). IF staining of COL2A1 (1:100, ARG20787, Arigo biolaboratories, China), Aggrecan (1:100, ACAN, MA3-16888, Thermo Fisher Scientific, USA), and SOX9 (1:100, abs154985, Absin, China) were carried out after 14 days. The genes were extracted on days 3, 7, and 14 for testing.

### Gene expression analysis

Real-time reverse transcription polymerase chain reaction (qRT-PCR) was used to assess gene expression. RNA was extracted using TRIzol (Thermo Corporation, USA) and reverse transcription was performed using a reverse transcription kit (Promega Corporation, USA). Commercial primers (Invitrogen, USA) were used to detect markers including *ACAN*, *COL2A1*, *SOX9*, *SDF1A*, *VEGF*, and glyceraldehyde-3-phosphate dehydrogenase (*GAPDH*). To standardize gene expression levels, *GAPDH* was used as an internal reference gene and corrected using the ^ΔΔ^Ct method. The primer sequences are listed in Supplementary Table 2.

### Inhibition of chondrocytes hypertrophy by KGN

BMSCs were cultured with chondrogenic differentiation medium for 3 weeks, after which the cells were divided into four portions and given conditioned environments for 2 weeks. To be specific, they were DMEM, DMEM + KGN (K0078, TCI, Japan, 100 nM), hypertrophy induction (HI) medium (DMEM containing 1% FBS (SV30208.02, HyClone), 1 nM of dexamethasone (D4902, Sigma-Aldrich), 0.1 mM of ascorbic acid (A92902, Sigma-Aldrich), 1× ITS premix (I3146, Sigma-Aldrich), 10 mM of β-glycerophosphate (ST637, Beyotime), and 1 nM of L-thyroxine (HY-18341, MCE)), HI + KGN (100 nM), respectively. The anti-hypertrophy effect of KGN was performed by adding of KGN to the HI medium. IF staining was used to determine collagen secretion.

### Vasculogenesis effect by the PAA-CHI-Cu^2+^ hydrogel

The in vivo vasculogenesis effect was verified by subcutaneous burial on the back of mice (male, 22–30 g, 8 weeks). A 2-cm incision was made on the back of mice, the subcutaneous tissue was freed, and a sterile hydrogel was inserted into the subcutaneous skin. The environment kept 18–22 °C, 40–70% relative humidity and 12/12 h light period. They were given with the sterile feed and water ad arbitrium. Two weeks later, the materials were removed, paraffin sectioned, and IF staining was performed for the CD31 (1:100, ARG52748, Arigo biolaboratories, China) and EMCN (1:100, bs-5884R, Bioss, China).

The in vitro vasculogenesis effect was verified by hydrogel-cell co-culture system established by transwell experiment. Briefly, hydrogels were placed in the upper layer, matrigel (354234, Corning, USA) was placed in the lower layer and seeded with HUVECs (iCell-h110, Cellverse, China). At specific time points, live cells were stained, photographed, and quantitatively analyzed for indicators of tubular cell formation using Image J software.

### Finite element analysis

Curve drawing and solid generation were performed in SOLIDWORKS software. Subsequently, the 3D model file was imported into ABAQUS software according to the model structure. The modulus of elasticity of cartilage, calcified cartilage, and subchondral bone was set at 5, 30, and 50 MPa, and Poisson’s ratio was set at 0.45, 0.38, and 0.3, respectively. Calcified cartilage was set in bound contact with subchondral bone, and cartilage was set in face-to-face contact with calcified cartilage.

To analyze the compressive stress, the lower surface of the subchondral bone was fixed, and a pressure load of 0.1 MPa was applied to the upper surface of the cartilage to analyze the magnitude and direction of the stress on the lower surface of the cartilage layer in different models. When analyzing the shear stress, the lower surface of the subchondral bone was still fixed, and a shear load of 0.1 MPa was applied to the left side surface of the cartilage layer to analyze the stress and displacement of the cartilage layer in different models.

In order to investigate the influence of the geometric parameters of waves on stress distribution and cell behavior, we prepared four additional molds with different parameters based on the original mold. The parameter information is listed in Supplementary Table 3.

### 3D culture of BMSCs

Hydrogel scaffolds with varied wavy interface geometries were fabricated using customized templates (M1-M5). Following the sequential crosslinking of the subchondral bone and cartilage layers, BMSCs were seeded into the cartilage layer and maintained in an unloaded static culture for 3 days to facilitate initial cell attachment and priming. Subsequently, the scaffolds were subjected to dynamic compression in a customized bioreactor, utilizing a sinusoidal loading pattern defined by amplitude (cos (2πt) - 1). This mechanical stimulation was applied at a frequency of 1 Hz for 1 h per day, 5 days per week, over a 14-day loading period^66^. This protocol was adopted based on established literature indicating its positive effects on cartilage tissue synthesis. Upon completion of the culture period, the constructs were harvested for comprehensive evaluation. Specifically, the expression of *COL2A1* was quantified via qRT-PCR and IF staining to assess the effects of interfacial topography.

### Surgical procedure

Thirty of New Zealand white rabbits (male, 2.5–3 kg, 3 months) were used in this study. After anesthesia, the rabbits were immobilized on an operating table, and the knee joints were sterilized. The medial knee ligaments and muscles were cut to dislocate the patella and expose the femoral trochanter. An osteochondral defect 5 mm in diameter and 3 mm in depth was created in the distal femoral trochanter using a coracoid perforator. Subsequently, the hydrogel scaffolds were implanted according to the groups. In order to study the impact of the mechanical environment on the regeneration effect, we encouraged the animals to engage in unrestricted activities within the cages. For further analysis, the rabbits were euthanized at 6 weeks and 12 weeks after the scaffold implantation.

### Evaluation of regenerative tissues

First, a general view was taken and scored according to the International Cartilage Repair Society (ICRS) criteria, as shown in Supplementary Table 4^67^. Then, MRI examination was performed on an MRI scanner (GE 3.0 T, USA). Subchondral bone was analyzed by microcomputed tomography (micro-CT, Inveon, Siemens, Germany). After evaluation of cartilage repair in different groups using histological methods, including H&E staining, Safranin O and Fast Green (SO&FG) staining, and Sirius red staining, the Wakitani score was quantitatively calculated in Supplementary Table 5^68^. IHC and IF staining were further performed to analyze collagen changes in the different groups, including COL2A1 (1:200, ARG20787, Arigo biolaboratories, China) and COL10A1 (1:200, GTX37732, GeneTex, USA for IHC and 1:100, bs-0554R, Bioss, China for IF).

### RNA-seq experiments

The regenerated cartilage layer was removed 4 weeks after scaffold implantation for RNA testing. An appropriate amount of tissue was ground into powder using liquid nitrogen and transferred into an EP tube containing TRIzol (15596018CN, Thermo Fisher Scientific, USA) lysate. After extraction, total RNA was characterized and quantified using an Agilent 2100 Bioanalyzer (Agilent, USA). Criteria for high quality RNA samples were as follows: OD260/280 = 1.8–2.0, RIN ≥ 6.5, 28S:18S ≥ 1.0, >1 μg. Transcriptome libraries were prepared with 1 μg of total RNA according to Illumina’s TruSeq™ RNA Sample Preparation Kit (USA). Next, messenger RNA was enriched by the magnetic bead method and libraries were constructed using the TruSeq RNA Sample Preparation Kit (Illumina). mRNA was extracted and purified using the poly A-tailed selection method and cDNA was synthesized and fragmented and ligated into junctions. The constructed library was subjected to high-throughput sequencing by Illumina NovaSeq 6000 platform, and raw data were generated by double-end sequencing strategy. After quality control and comparison with the reference genome, differential expression analysis and functional enrichment of genes from different groups were performed. Further in-depth exploration of genetic alterations associated with changes in the shape of the calcified cartilage layer was performed based on hypergeometric tests using Phyper for GO (http://www.geneontology.org/) and KEGG enrichment analysis of differential genes, with Qvalue ≤ 0.05 as the threshold, and those satisfying this condition were defined as significantly enriched in candidate genes.

### Statistical analysis

All data were expressed as means ± standard deviation of three representative experiments. Parametric statistics were conducted using GraphPad Prism (10.5.0). The sample size or number of biological replicates was shown in the figure legend. The stability of each result was verified by at least three independent experiments. Correlations between dependent variables were analyzed by two-tailed Student’s *t*-test for two groups or one-way ANOVA followed by Tukey’s post-hoc test for three or more groups. *p < 0.05, **p < 0.01, ***p < 0.001 were regarded as statistically significant. In addition, “ns” denoted no significant difference.

### Reporting summary

Further information on research design is available in the Nature Portfolio Reporting Summary linked to this article.

## Supplementary information

Supporting information

Reporting Summary

Transparent Peer Review file

## Source data

Source Data

## Data Availability

All data supporting the findings of this study are available within the article and its supplementary files. Raw sequencing data generated in this study have been deposited in the NCBI SRA database under accession number “PRJNA1305715” (https://www.ncbi.nlm.nih.gov/sra/PRJNA1305715). Source data are provided with this paper.
