# Author Correction: A decoupling strategy toward spatiotemporal regulation and biomechanical transmission of sandwiched scaffold for osteochondral regeneration

**DOI:** 10.1038/s41467-026-79199-w

**Published:** 2026-10-26

**Authors:** Xuemiao Liu, Mingze Du, Weiguo Zhang, Kang Tian, Fuzhen Yuan, Xing Wang

**Affiliations:** 1 Beijing National Laboratory for Molecular Sciences, Institute of Chemistry, Chinese Academy of Sciences, Beijing, 100190 China; 2 Department of Bone & Joint, First Affiliated Hospital of Dalian Medical University, Dalian, 116000 China; 3 Department of Sports Medicine, Peking University Third Hospital, Beijing, 100191 China

**Keywords:** Translational research, Outcomes research, Biomedical engineering, Biomaterials

Correction to: *Nature Communications* https://doi.org/10.1038/s41467-026-71810-4, published online 17 April 2026

In the version of this article initially published, in the Fig. 6d Source Data and associated figure plot, values presented were inadvertent duplicates of Supplementary Fig. 10 data. The Source Data and figure are now updated in the HTML and PDF versions of the article. For comparison, the original figure is available as Supplementary Information accompanying this amendment.

## Supplementary information

Original Fig. 6

