# Supplementary material for: A decoupling strategy toward spatiotemporal regulation and biomechanical transmission of sandwiched scaffold for osteochondral regeneration
Source: Nat Commun. 2026 Apr 17;17:5351. doi: 10.1038/s41467-026-71810-4 (PMC13272660; doi:10.1038/s41467-026-71810-4)
Supplement: Supplementary file 1 — Supporting information [file 41467_2026_71810_MOESM1_ESM.pdf]

Supplementary Information

A decoupling strategy toward spatiotemporal regulation and biomechanical transmission of sandwiched scaffold for osteochondral regeneration

Xuemiao Liu[1,2,†], Mingze Du[3,†], Weiguo Zhang[2], Kang Tian[2*], Fuzhen Yuan[3*] & Xing Wang[1*]

[1]Beijing National Laboratory for Molecular Sciences, Institute of Chemistry, Chinese Academy of Sciences, Beijing 100190, China.

[2]Department of Bone & Joint, First Affiliated Hospital of Dalian Medical University, Dalian 116000, China.

[3]Department of Sports Medicine, Peking University Third Hospital, Beijing 100191, China.

† These authors contribute equally to this work.

***Correspondence:**

E-mail: dmu-tiankang@outlook.com (K.T.); yuanfuzhen@pku.edu.cn (F.Z.Y.);

wangxing@iccas.ac.cn (X.W.)

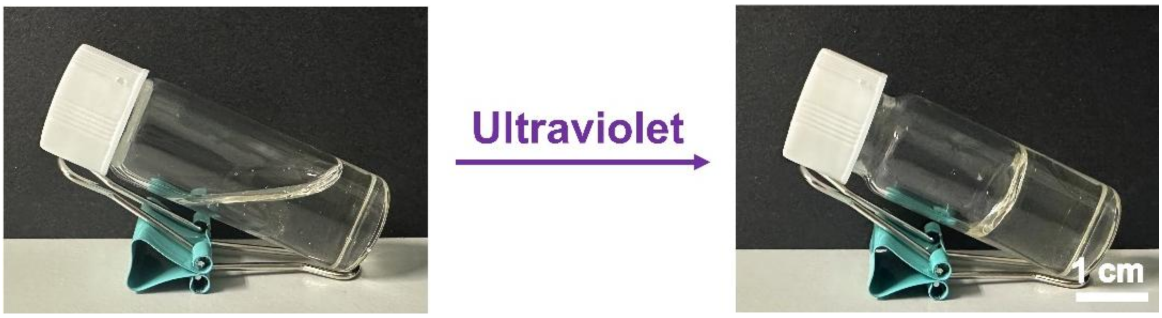

Supplementary Fig. 1 Gelation process of PAA-CHI-X hydrogel under UV irradiation.

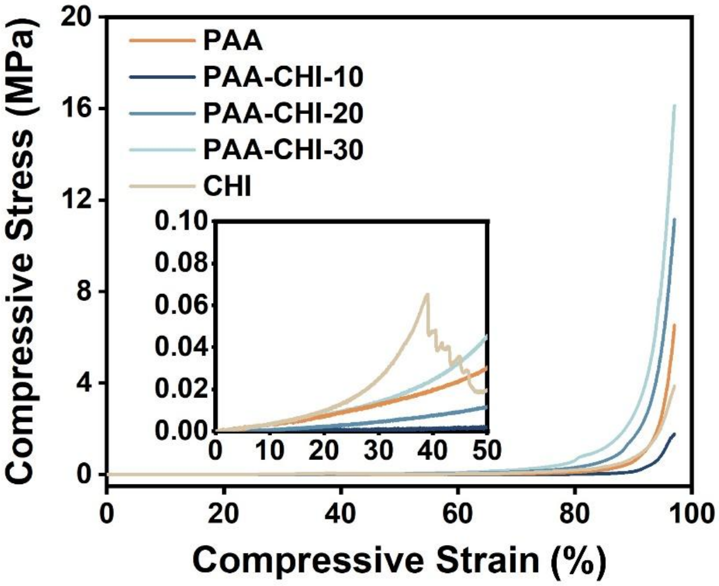

Supplementary Fig. 2 Stress-strain curves of PAA, PAA-CHI-10, PAA-CHI-20, PAA-CHI-30, and CHI hydrogels. Source data are provided as a Source Data file.

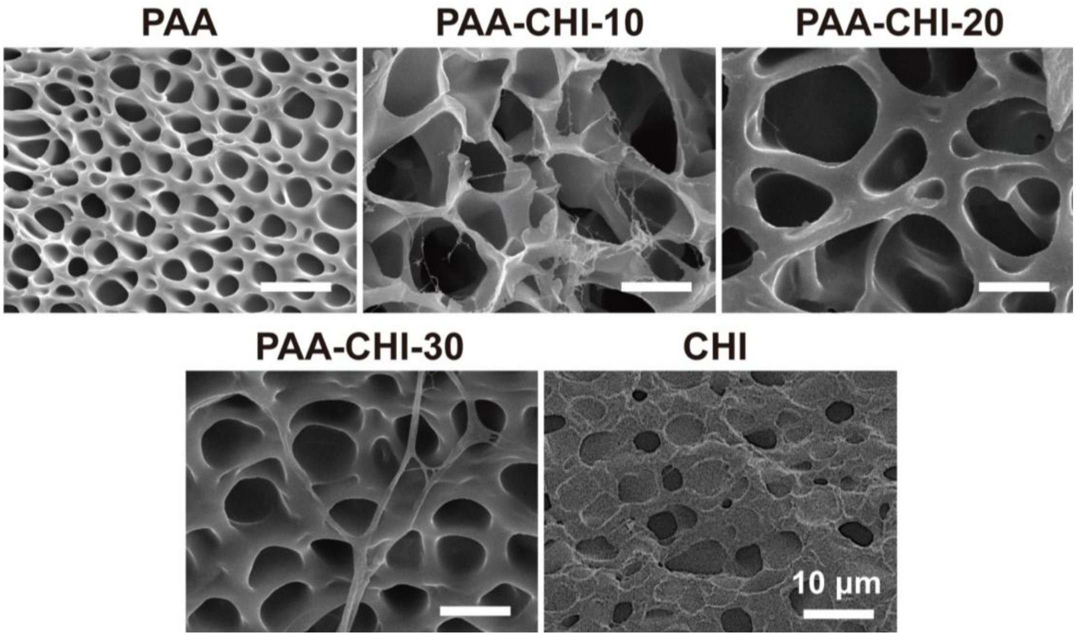

Supplementary Fig. 3 SEM images of PAA, PAA-CHI-10, PAA-CHI-20, PAA-CHI-30, and CHI hydrogels.

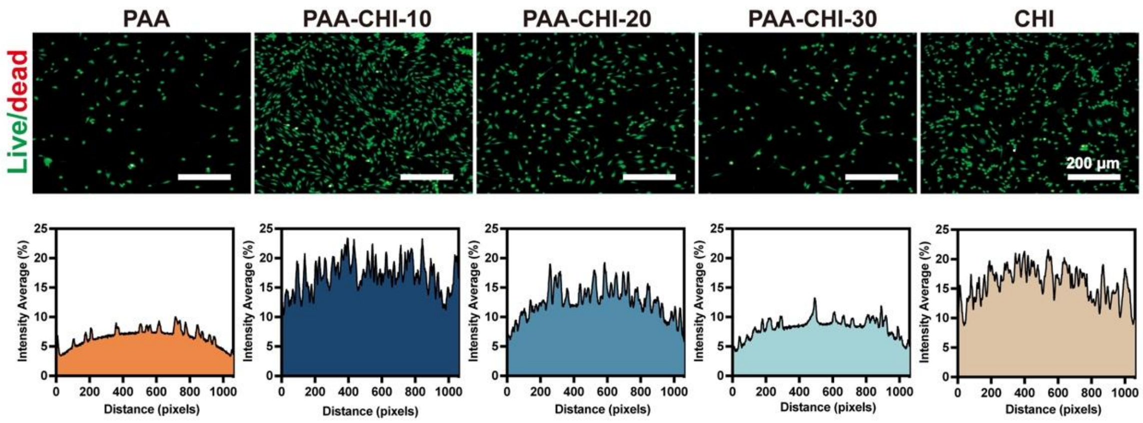

Supplementary Fig. 4 Live/dead staining of BMSCs under the influence of PAA, PAA-CHI-10, PAA-CHI-20, PAA-CHI-30, and CHI hydrogels. Source data are provided as a Source Data file.

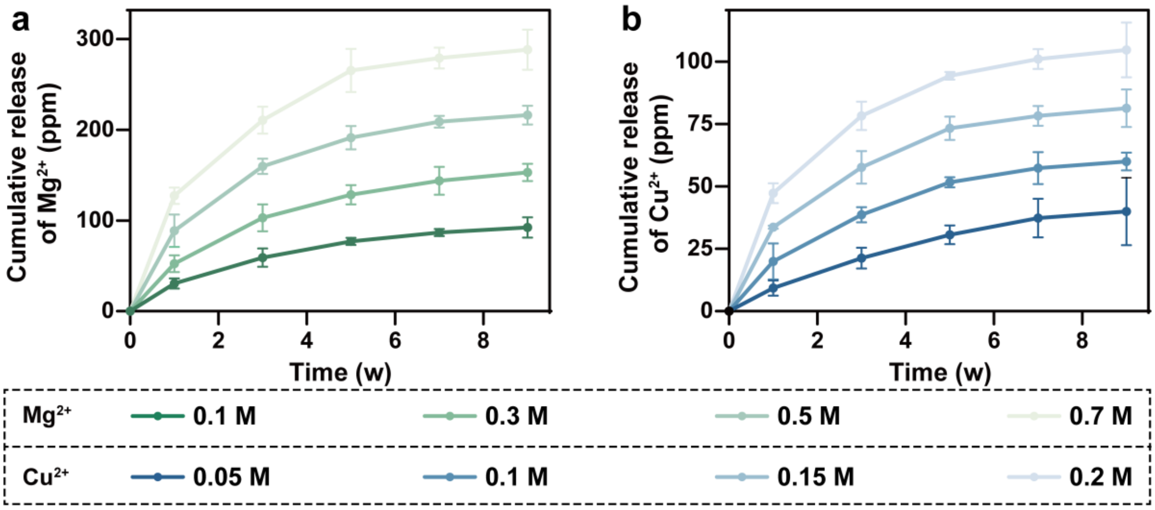

Supplementary Fig. 5 The ion release curves from PAA-CHI-20 hydrogels after various ionic soaking treatment, respectively. a) The release curves of Mg^{2+} from the PAA-CHI-20 hydrogels after soaking into $MgSO_4$ solution with different concentrations. **b)** The release curves of Cu^{2+} from the PAA-CHI-20 hydrogels after soaking into $CuSO_4$ solution with different concentrations. Data in **a,b)** were presented as means ± SD, n = 3. Source data are provided as a Source Data file.

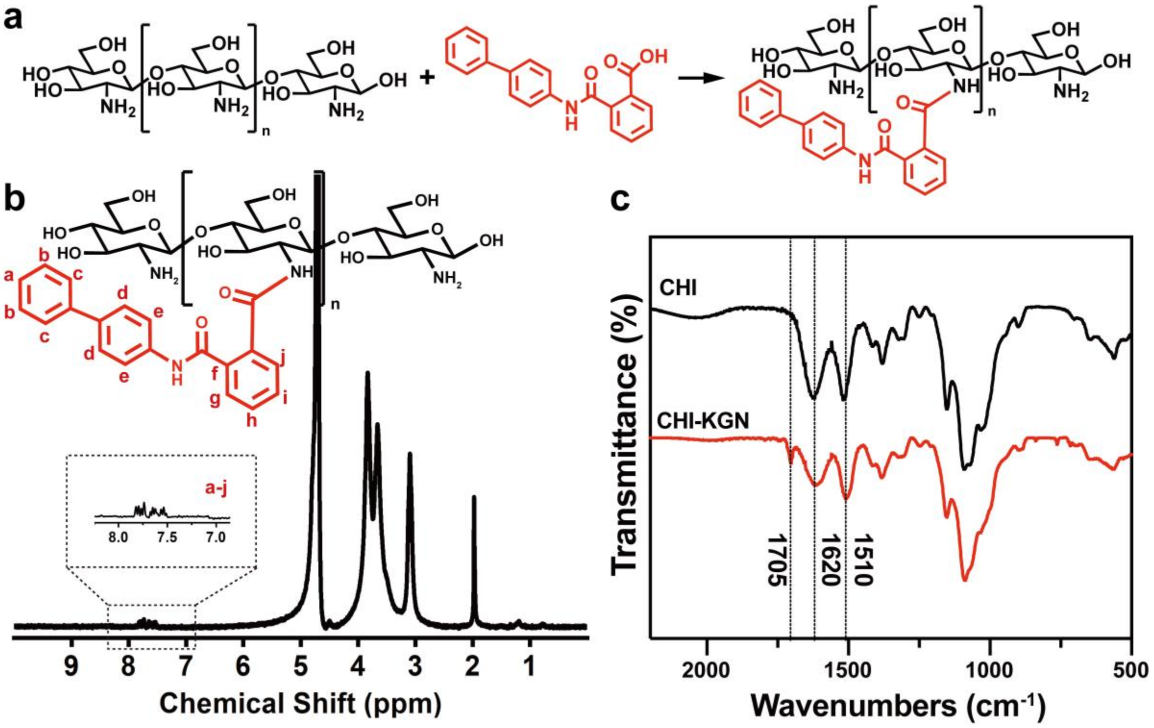

Supplementary Fig. 6 Synthesis and characterization of CHI-KGN conjugate. a) The synthetic route of CHI-KGN conjugate by amidation reaction. **b,c)** ^{1}H NMR and FT-IR spectra of CHI-KGN conjugate. The new resonant peaks at 7.3-7.9 ppm in ^{1}H-NMR spectrum and the appearance of a stretching vibrational absorption peak of C=O at 1705 cm^{-1} in FT-IR spectrum collectively confirmed the successful chemical modification of KGN onto the CHI. Source data are provided as a Source Data file.

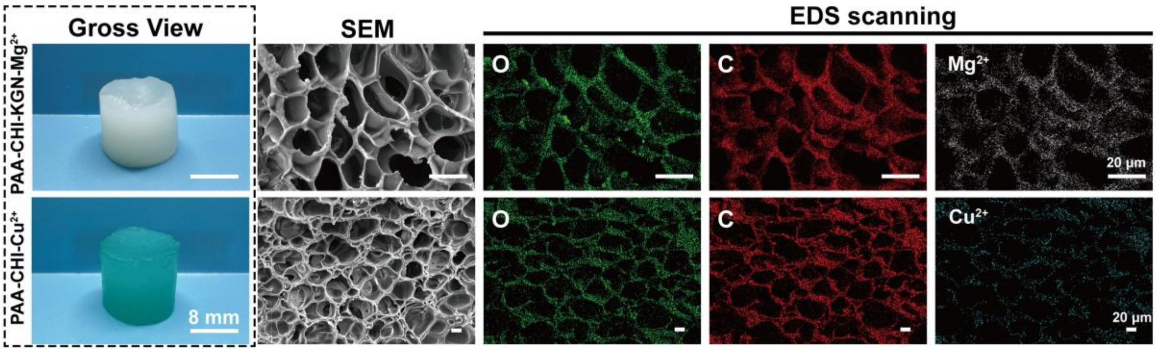

Supplementary Fig. 7 Gross view, SEM and EDS images of the PAA-CHI-KGN-Mg^{2+} and PAA-CHI-Cu^{2+} hydrogels.

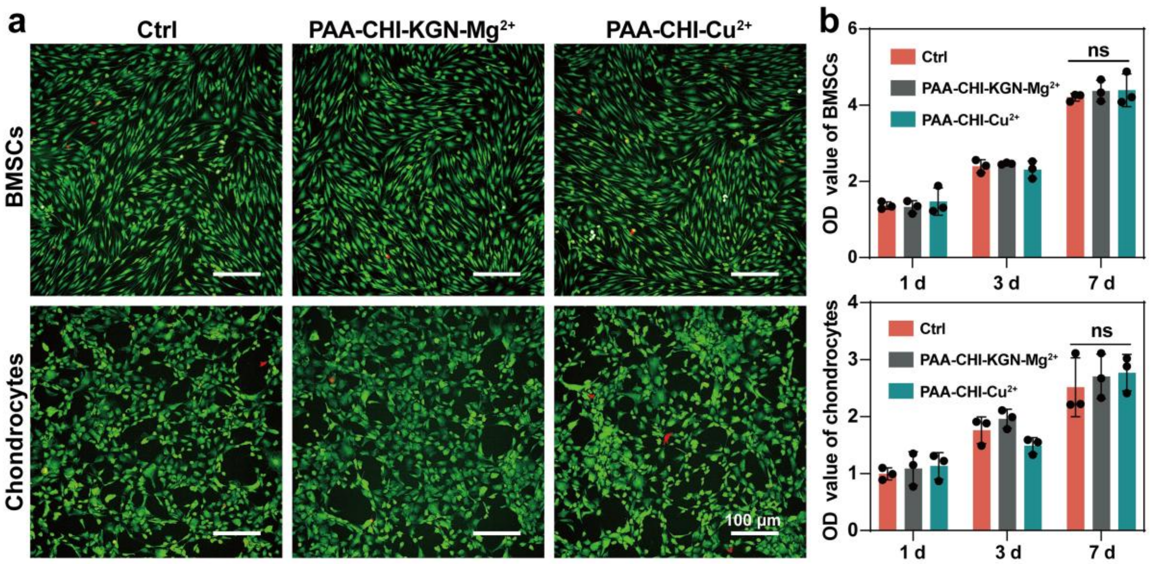

Supplementary Fig. 8 Cell compatibility of PAA-CHI-KGN-Mg^{2+} and PAA-CHI-Cu^{2+} hydrogels. **a)** Live/dead staining of BMSCs and chondrocytes at 7 days. **b)** CCK-8 assay of BMSCs and chondrocytes at specific times. Data in **b)** were presented as means ± SD, n = 3. Statistical significance was determined using the one-way ANOVA with Tukey's post-hoc test. Source data are provided as a Source Data file.

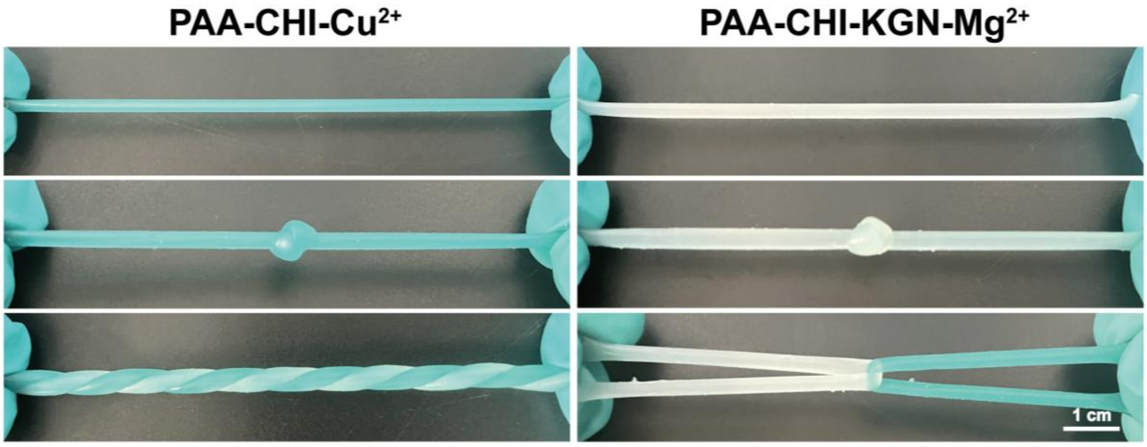

Supplementary Fig. 9 The extraordinary mechanical properties of the PAA-CHI-Cu^{2+} and PAA-CHI-KGN-Mg^{2+} hydrogels: stretching, knotting, twisting, and crossover stretching.

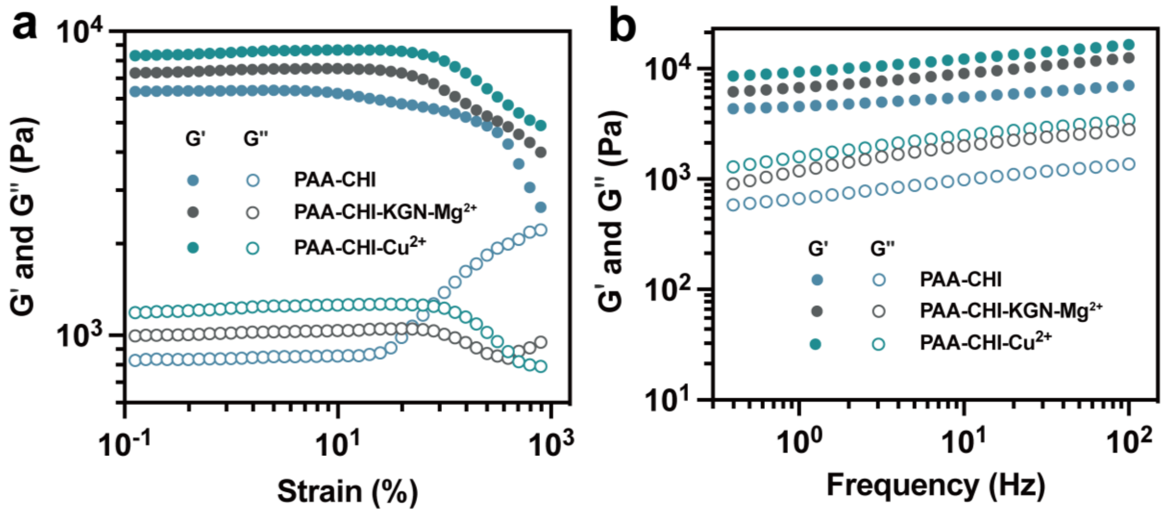

Supplementary Fig. 10 Rheological evaluation of PAA-CHI, PAA-CHI-KGN-Mg^{2+}, and PAA-CHI-Cu^{2+} hydrogels. a,b) Strain scanning and frequency scanning profiles of PAA-CHI, PAA-CHI-KGN-Mg^{2+}, and PAA-CHI-Cu^{2+} hydrogels. Source data are provided as a Source Data file.

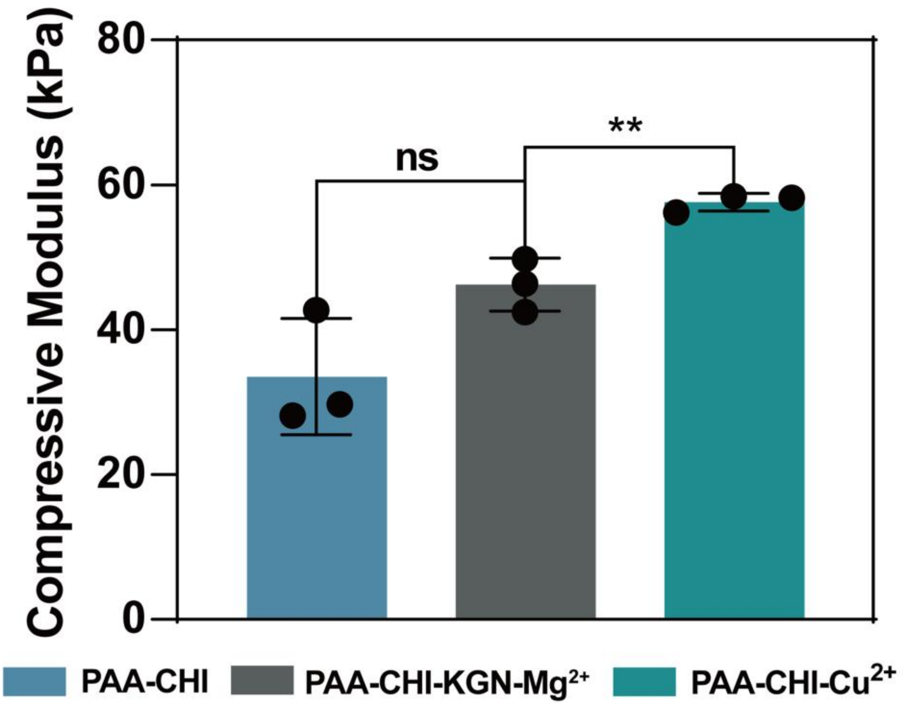

Supplementary Fig. 11 Compression modulus of PAA-CHI, PAA-CHI-KGN-Mg^{2+}, and PAA-CHI-Cu^{2+} hydrogels. (**p = 0.0029, n = 3 independent samples). Data were presented as means ± SD. Statistical significance was determined using the one-way ANOVA with Tukey's post-hoc test. Source data are provided as a Source Data file.

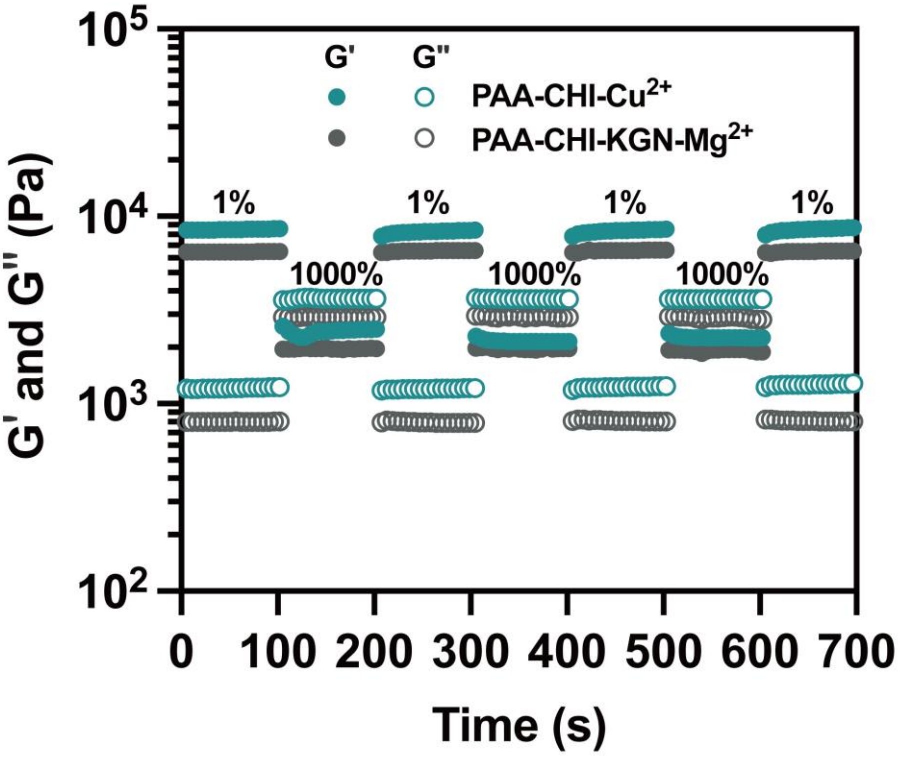

Supplementary Fig. 12 Self-healing behaviors of PAA-CHI-KGN-Mg^{2+} and PAA-CHI-Cu^{2+} hydrogels. Source data are provided as a Source Data file.

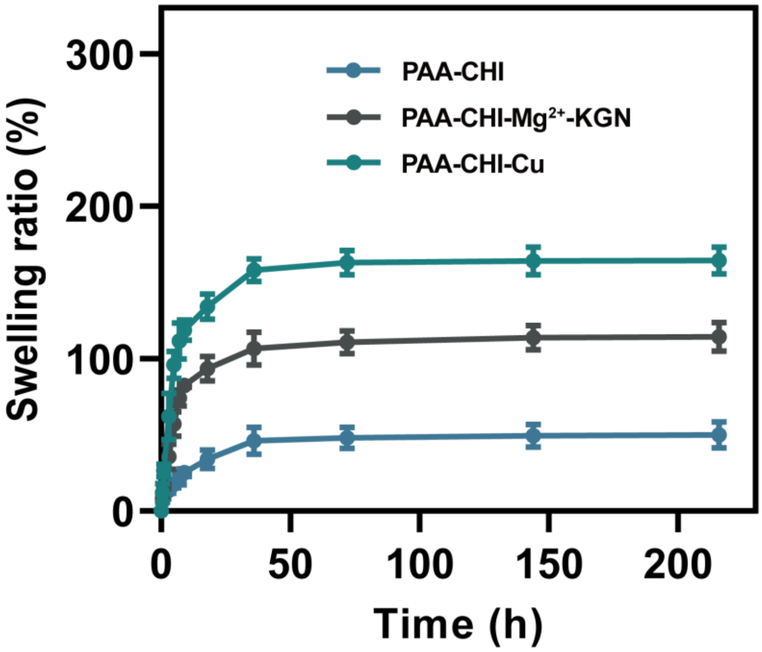

Supplementary Fig. 13 Swelling properties of PAA-CHI, PAA-CHI-KGN-Mg^{2+}, and PAA-CHI-Cu^{2+} hydrogels. Data are presented as means ± SD, n = 3. Source data are provided as a Source Data file.

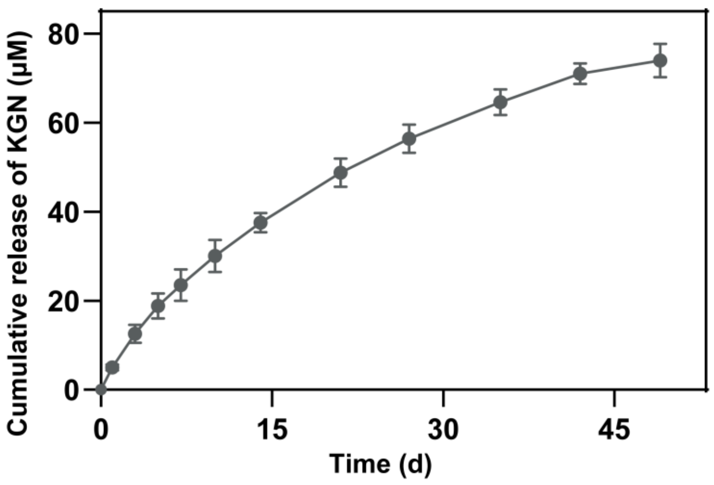

Supplementary Fig. 14 Cumulative release of KGN from the PAA-CHI-KGN-Mg^{2+} hydrogel. Data were presented as means ± SD, n = 3. Source data are provided as a Source Data file.

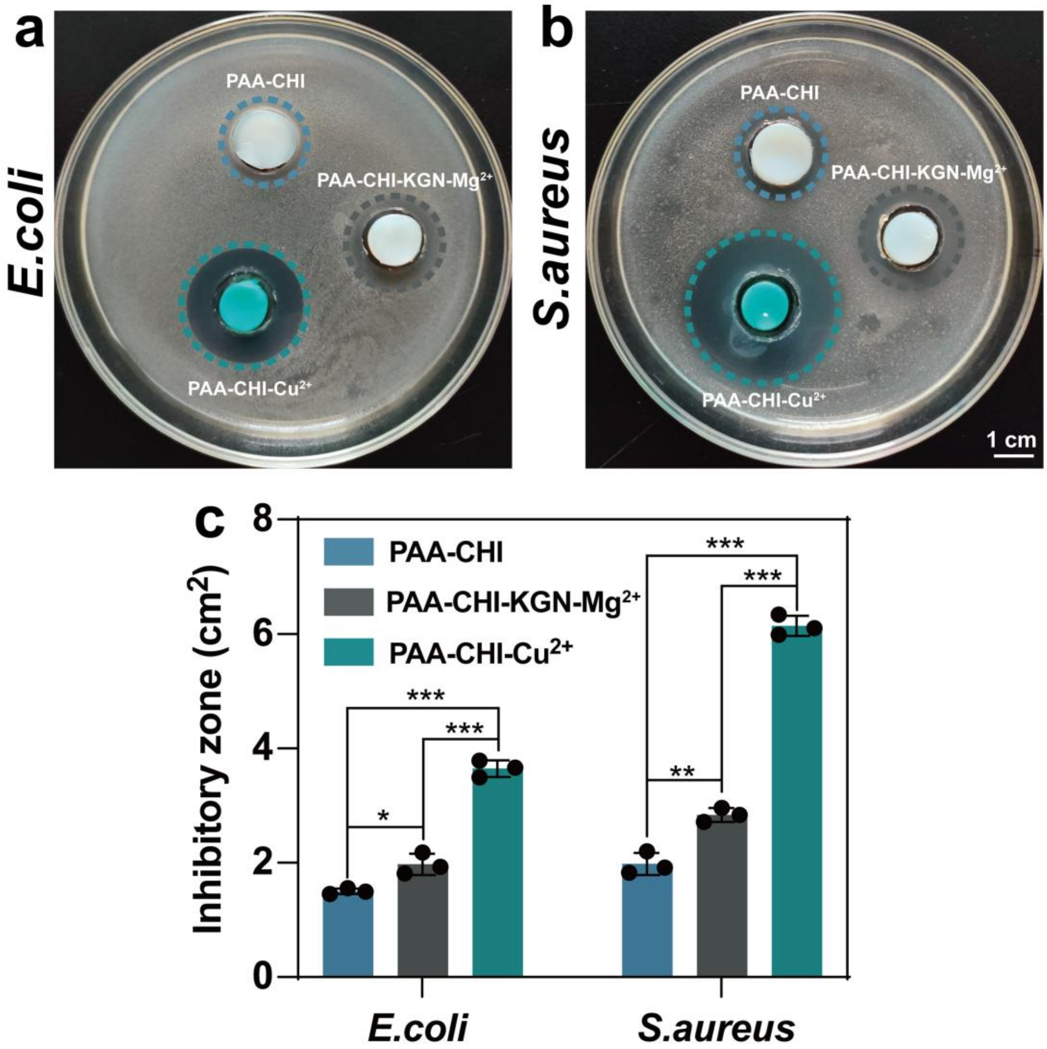

Supplementary Fig. 15 Metal ions endow hydrogels with antimicrobial properties. a,b) Evaluation of the antimicrobial activity of PAA-CHI, PAA-CHI-KGN-Mg^{2+}, and PAA-CHI-Cu^{2+} hydrogels against *E. coli* and *S. aureus* by Kirby-Bauer disks. **c)** Analysis of antimicrobial area. (for *E. coli*: **p* = 0.0143, ****p* < 0.0001; for *S. aureus*: ***p* = 0.0019, ****p* < 0.0001, n = 3 independent samples). Data in **c)** were presented as means ± SD. Statistical significance was determined using the one-way ANOVA with Tukey's post-hoc test. Source data are provided as a Source Data file.

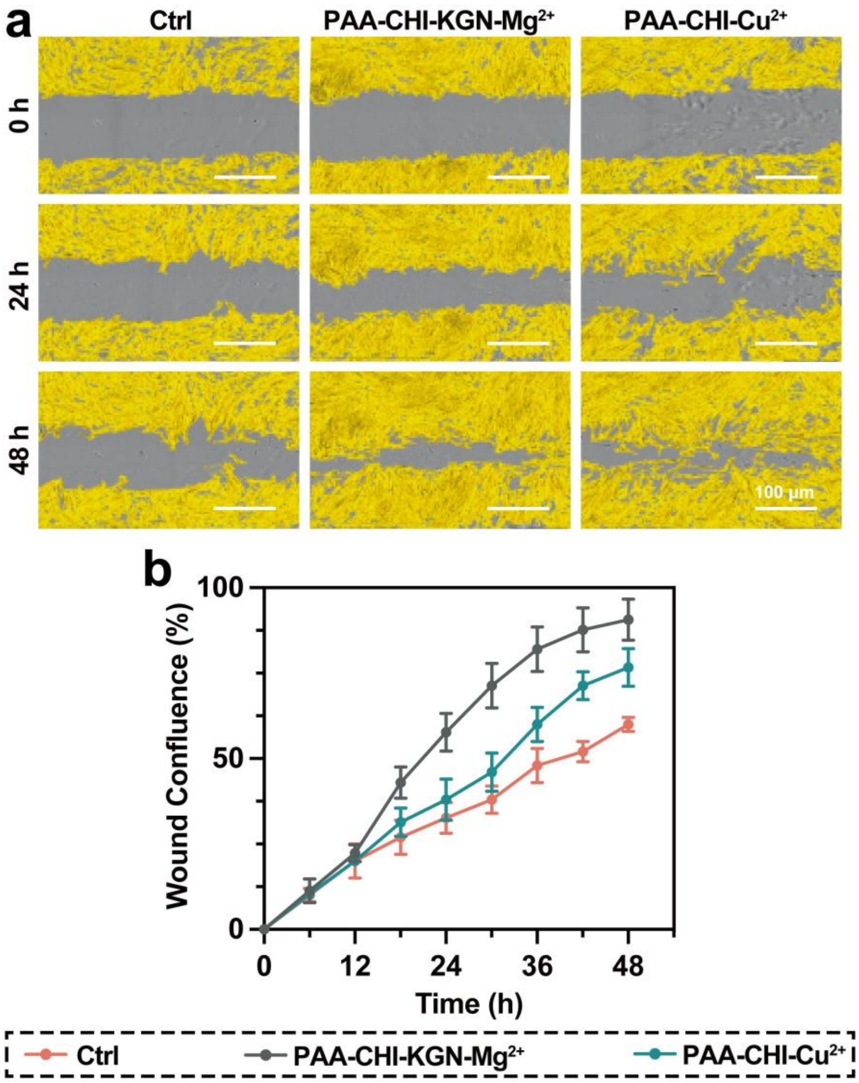

Supplementary Fig. 16 Metal ions and KGN promote horizontal migration of BMSCs. a) Cell migration maps of Ctrl, PAA-CHI-KGN-Mg^{2+}, and PAA-CHI-Cu^{2+} groups collected at specific time points. **b)** Quantification of the proportion of wounds healed at specific time points. Data in **b)** were presented as means ± SD, n = 3. Source data are provided as a Source Data file.

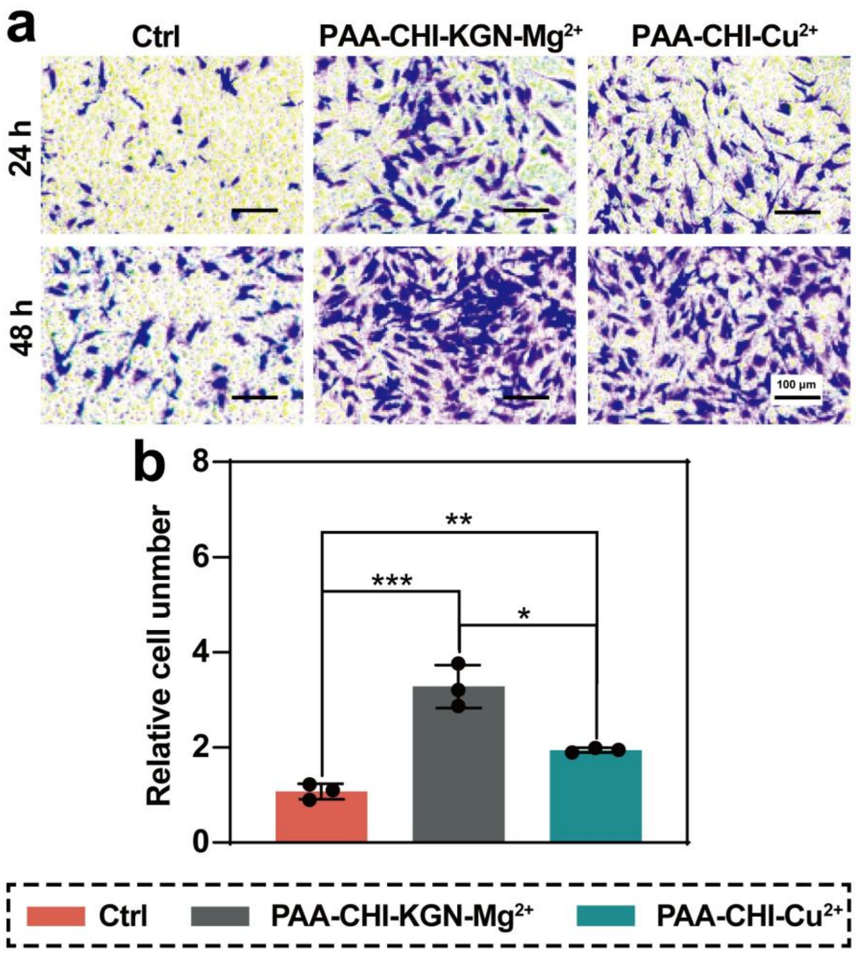

Supplementary Fig. 17 Metal ions and KGN promote vertical migration of BMSCs. **a)** Cell migration maps of different groups collected at specific time points. **b)** Relative cell numbers of different groups at 48 h. (**p* = 0.0199, ****p* = 0.0002, ***p* = 0.0025, n = 3 independent samples). Data in **b)** were presented as means ± SD. Statistical significance was determined using the one-way ANOVA with Tukey's post-hoc test. Source data are provided as a Source Data file.

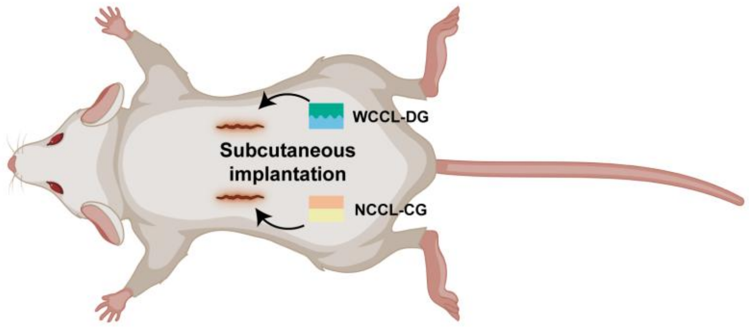

Supplementary Fig. 18 Schematic diagram of subcutaneous implantation of hydrogel scaffolds in rats. Created in BioRender. Ke, L. https://BioRender.com/7oc77n8.

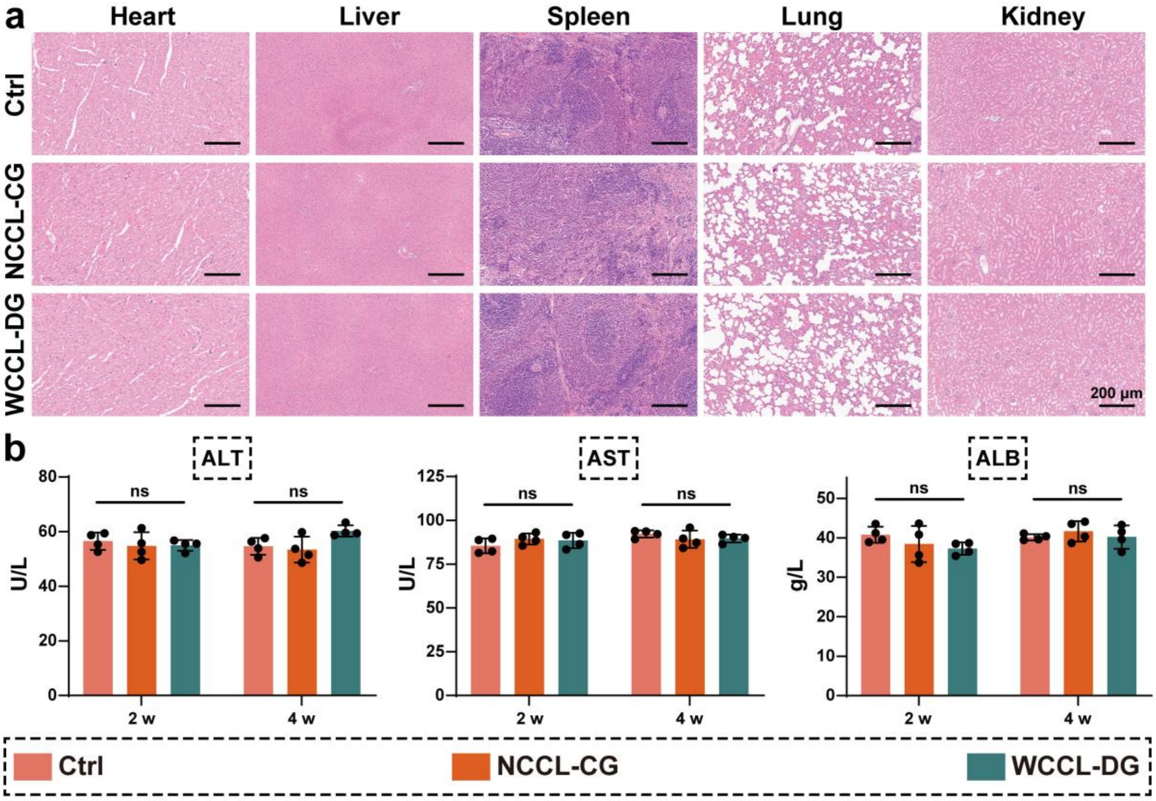

Supplementary Fig. 19 Biological safety verification of hydrogel scaffolds. a) Histological evaluation of rats' organs (Heart, Liver, Spleen, Lung, and Kidney). Representative H&E staining images of rat's organs following 1 month of *in vivo* hydrogel implantation. The experiment was repeated four times with similar results**. b)** Liver function test results at different time points, including alanine aminotransferase (ALT), aspartate aminotransferase (AST), and serum albumin (ALB). Data were presented as means ± SD, n = 4. Statistical significance was determined using the one-way ANOVA with Tukey's post-hoc test. Source data are provided as a Source Data file.

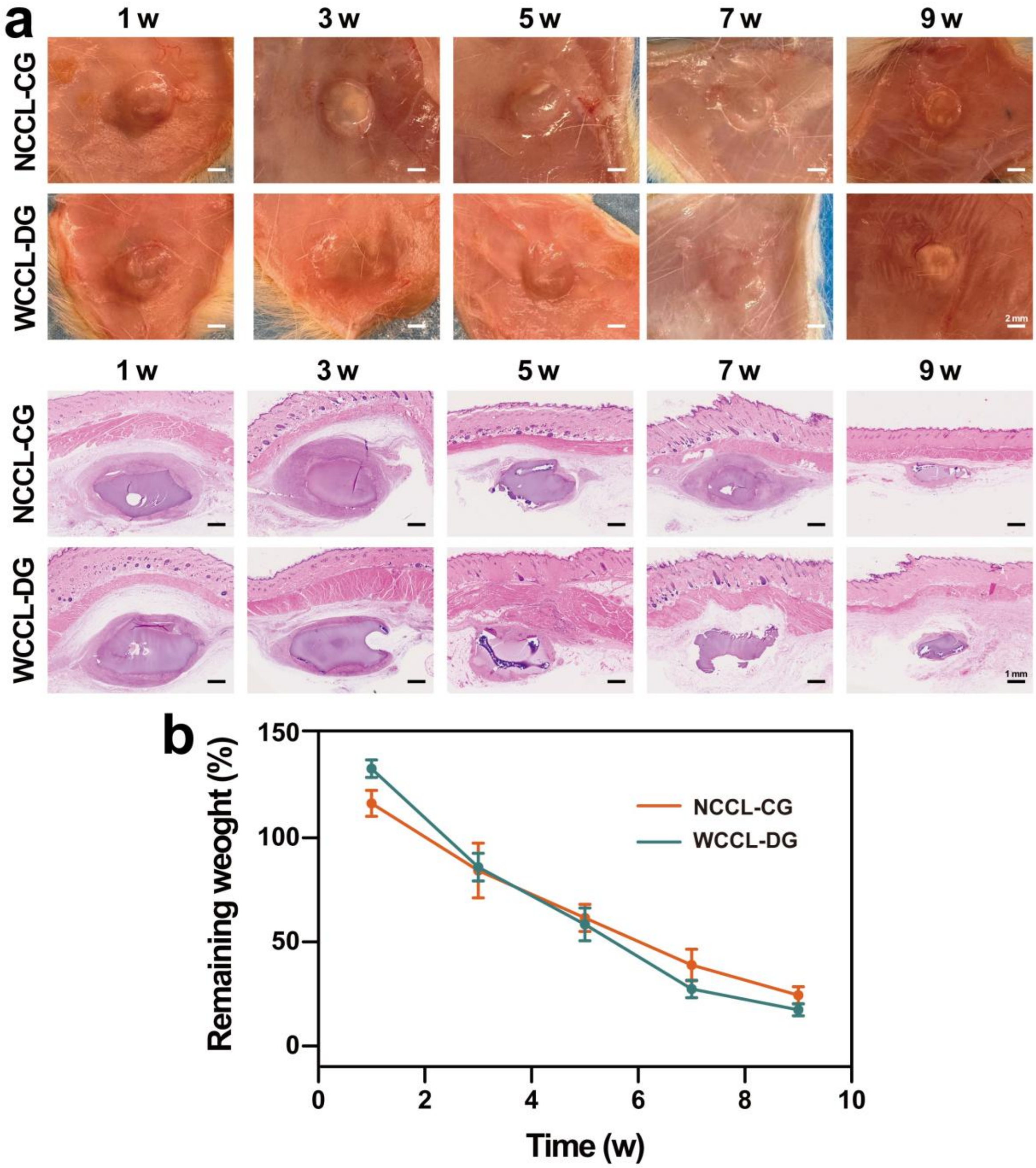

Supplementary Fig. 20 Subcutaneous degradation behaviors of WCCL-DG and NCCL-CG scaffolds. a) Macrograph and H&E staining images of WCCL-DG and NCCL-CG hydrogels with the surrounding skin at the different time point. **b)** The mass remained of WCCL-DG and NCCL-CG hydrogels at the different time point. Data in **b)** were presented as means ± SD, n = 3.

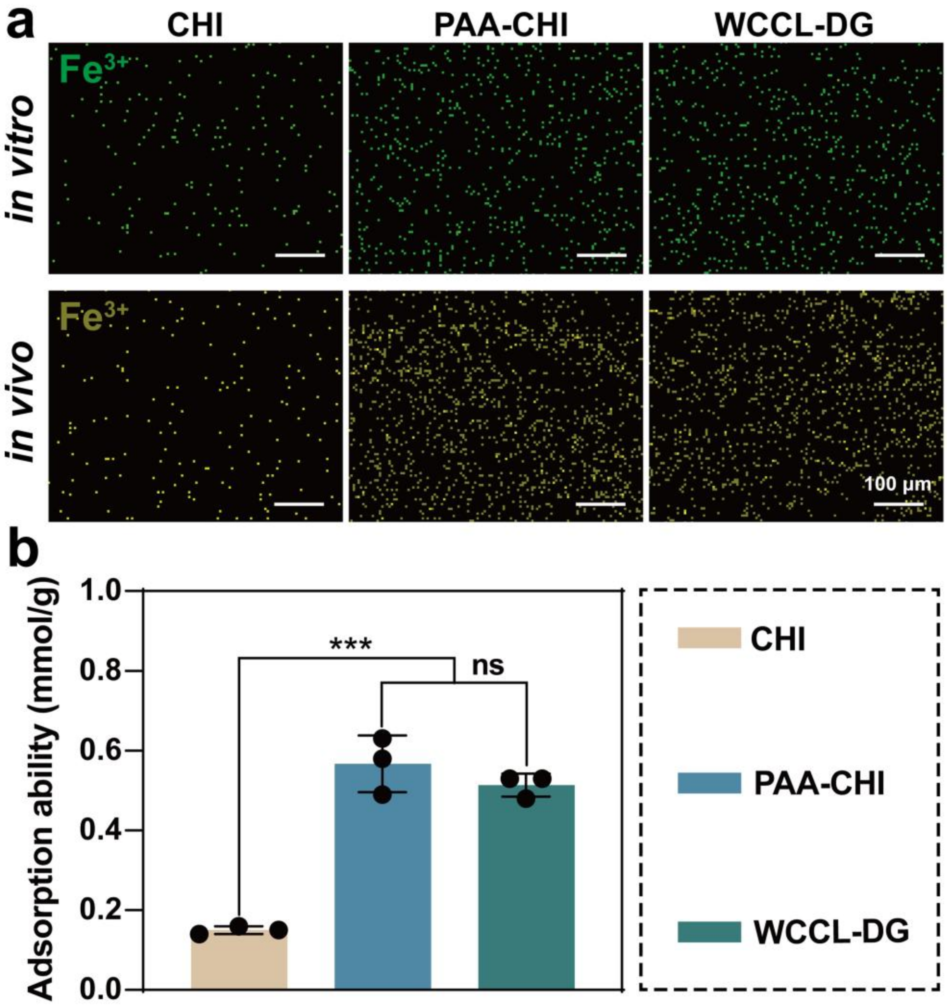

Supplementary Fig. 21 Verification of iron ion chelating ability of CHI, PAA-CHI and WCCL-DG groups. a) EDS scanning of hydrogels after *in vitro* soaking in a ferric ion solution or *in vivo* dorsal burial. **b)** Quantitative analysis of iron in hydrogels by ICP. (***$p < 0.0001$, ***$p = 0.0001$, n = 3 independent samples). Data in **b)** were presented as means ± SD. Statistical significance was determined using the one-way ANOVA with Tukey's post-hoc test. Source data are provided as a Source Data file.

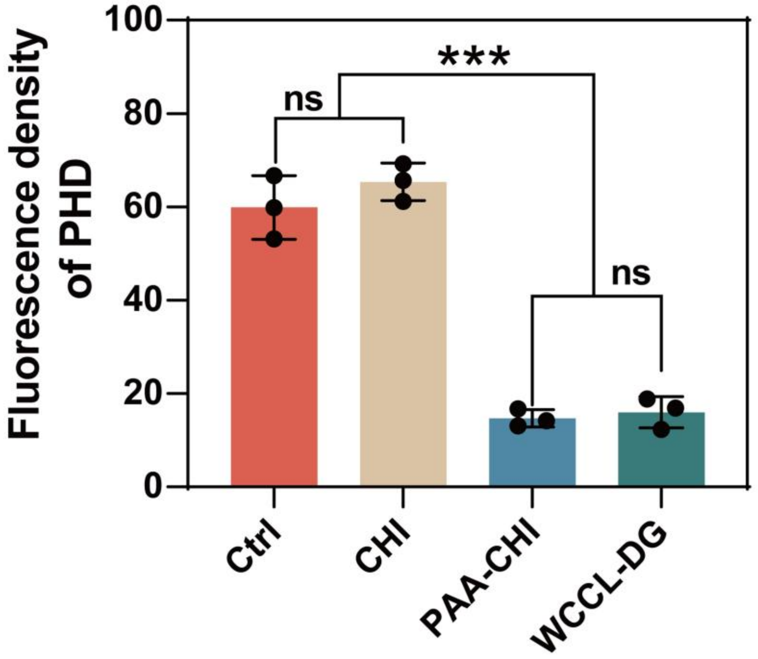

Supplementary Fig. 22 Quantitative analysis of intensity of PHD in BMSCs co-cultured with CHI, PAA-CHI and WCCL-DG scaffolds. (***p < 0.0001, n = 3 independent samples). Data were presented as means ± SD, n = 3. Statistical significance was determined using the one-way ANOVA with Tukey's post-hoc test. Source data are provided as a Source Data file.

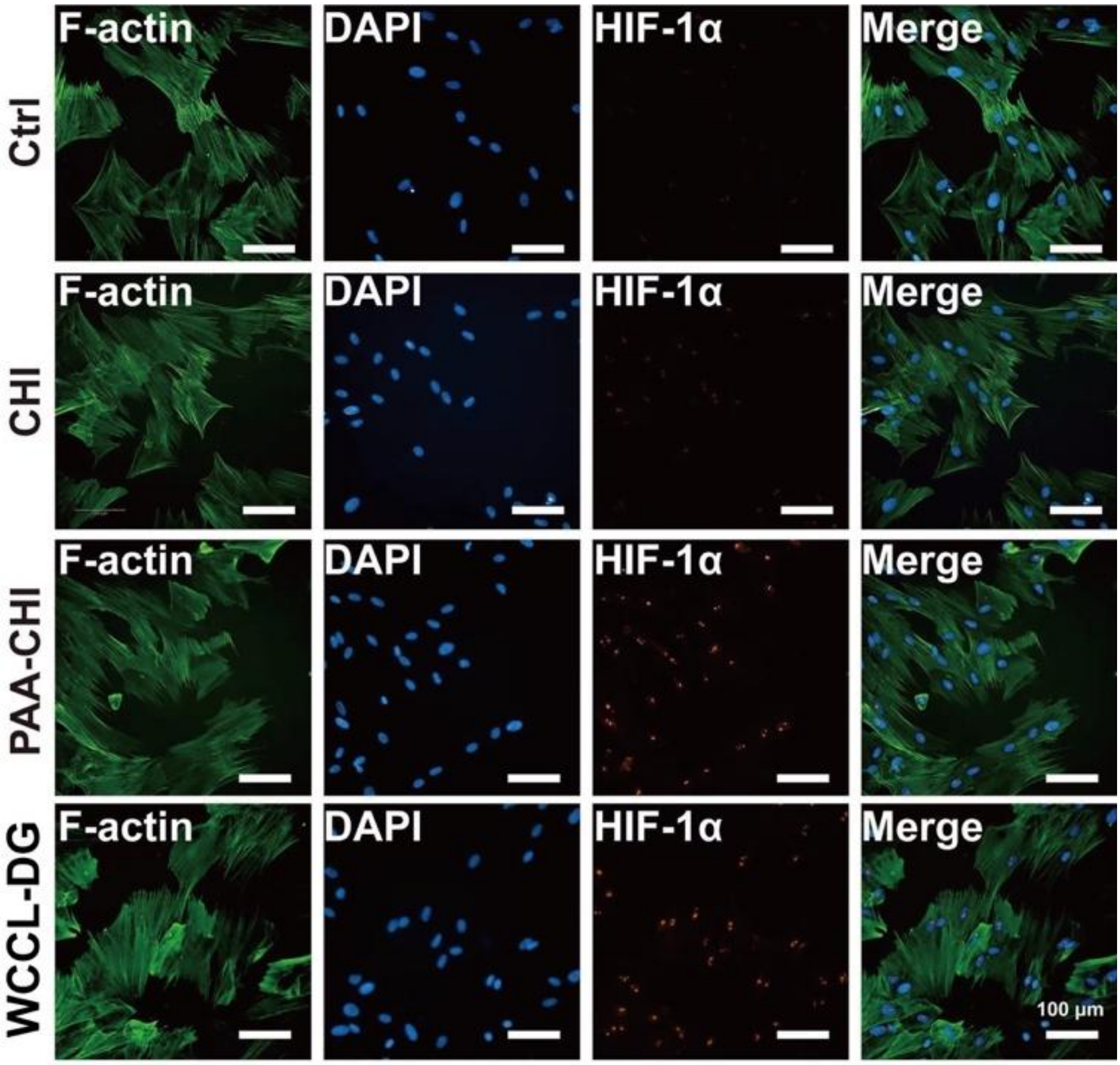

Supplementary Fig. 23 IF staining of HIF-1α in BMSCs co-cultured with CHI, PAA-CHI, and WCCL-DG scaffolds at 2 h.

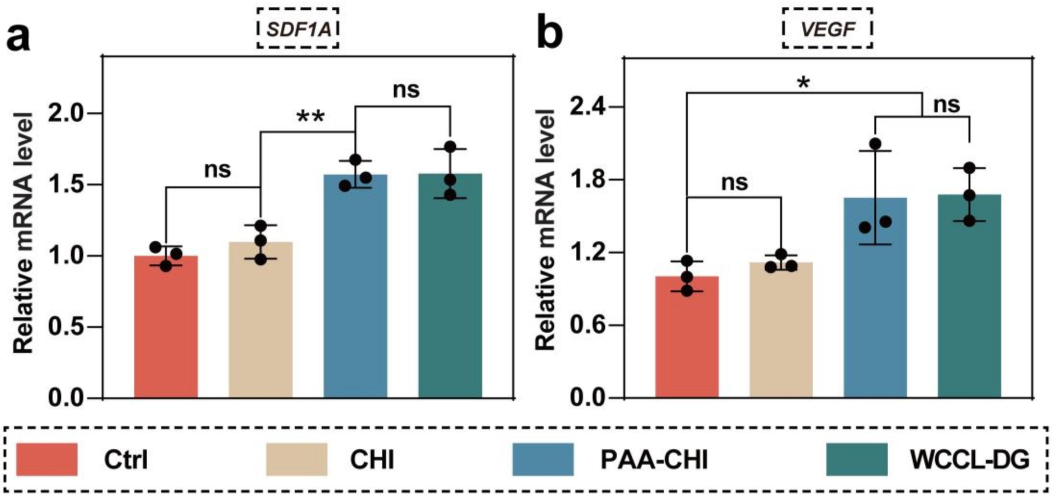

Supplementary Fig. 24 Expression of downstream factors of HIF-1α on various hydrogels. Relative mRNA expression of **a)** *SDF1A* and **b)** *VEGF* of BMSCs co-culture with various hydrogels. (for *SDF1A*: ***p* = 0.0056; for *VEGF*: **p* = 0.0367, **p* = 0.0306, n = 3 independent samples). Data in **a,b)** were presented as means ± SD. Statistical significance was determined using the one-way ANOVA with Tukey's post-hoc test. Source data are provided as a Source Data file.

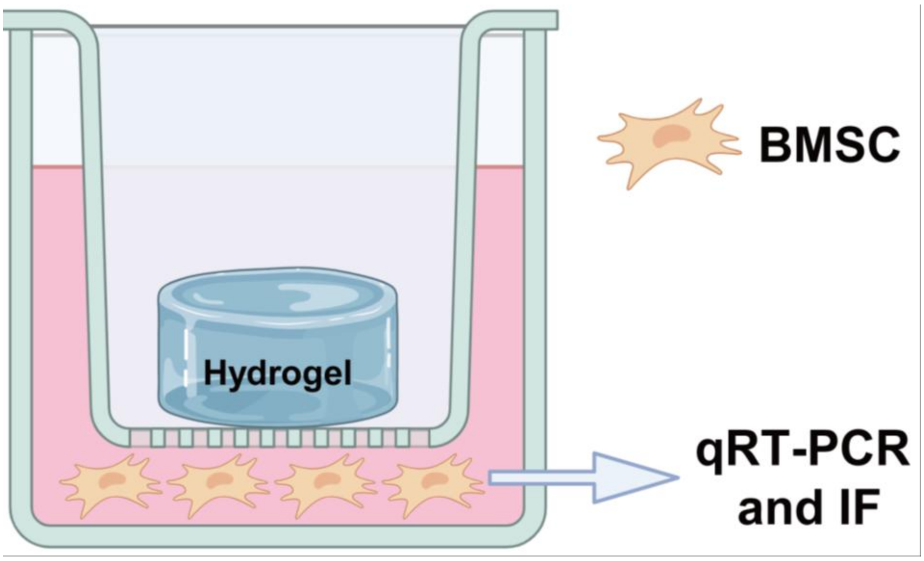

Supplementary Fig. 25 Schematic diagram of hydrogel-BMSCs co-culture system. Created in BioRender. Ke, L. https://BioRender.com/yefp7dx.

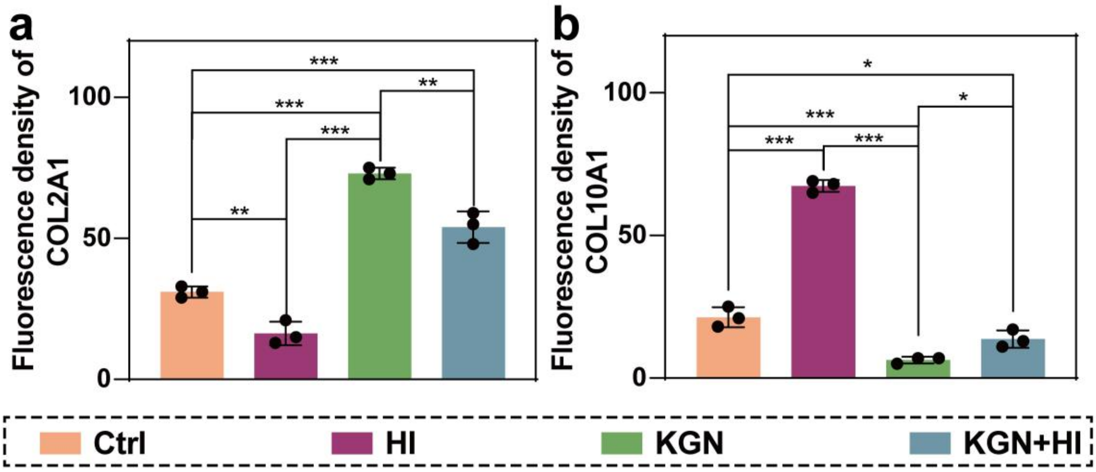

Supplementary Fig. 26 The characterization of KGN's inhibitory effect on chondrocytes hypertrophy. a,b) IF staining intensity of COL2A1 and COL10A1. (for COL2A1: **p = 0.0060, ***p < 0.0001, ***p < 0.0001, **p = 0.0012, ***p = 0.0003; for COL10A1: ***p < 0.0001, ***p = 0.0005, *p = 0.0361, *p = 0.0290, n = 3 independent samples). Data were presented as means ± SD. Statistical significance was determined using the one-way ANOVA with Tukey's post-hoc test. Source data are provided as a Source Data file.

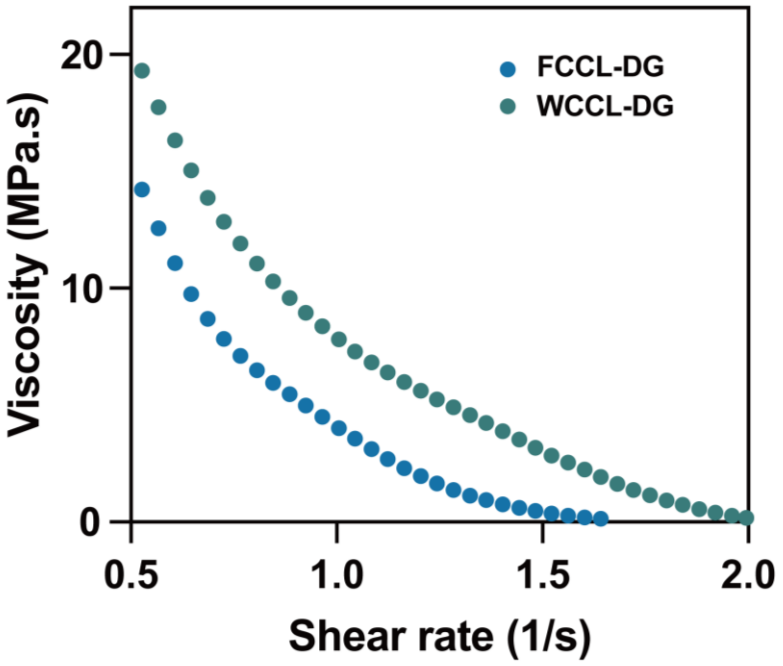

Supplementary Fig. 27 Comparison of viscosity of FCCL-DG and WCCL-DG scaffolds. Source data are provided as a Source Data file.

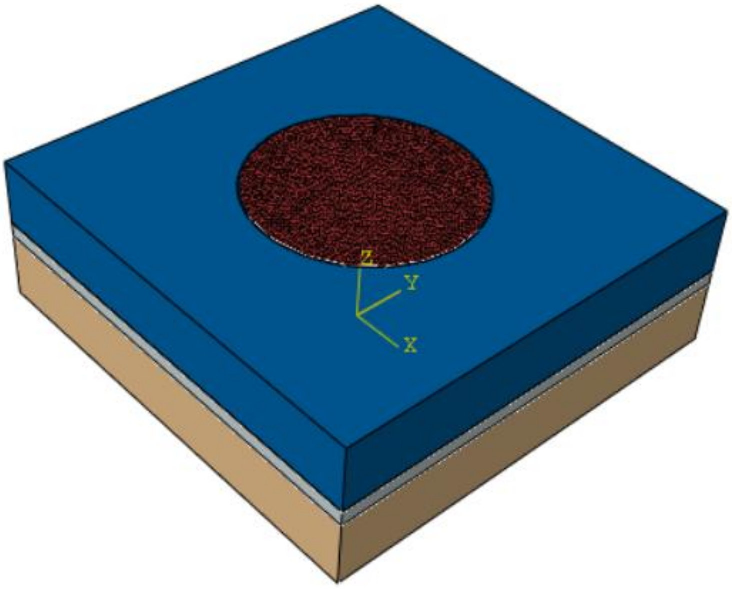

Supplementary Fig. 28 Presentation of the finite element model preparation and analysis sections. After applying different mechanical stimuli, a force analysis was conducted on the middle red cylinder.

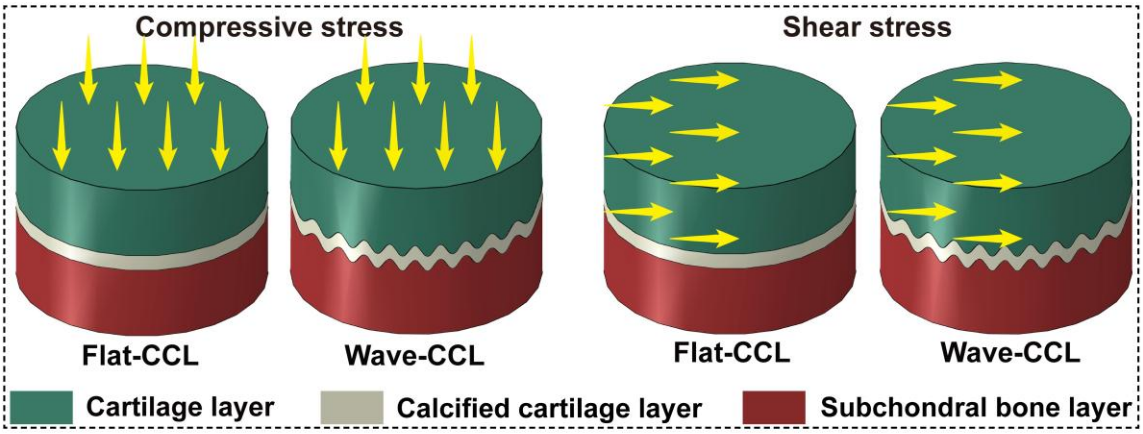

Supplementary Fig. 29 Schematic diagram of mechanical response under two representatively compressive and shear stresses by FEA.

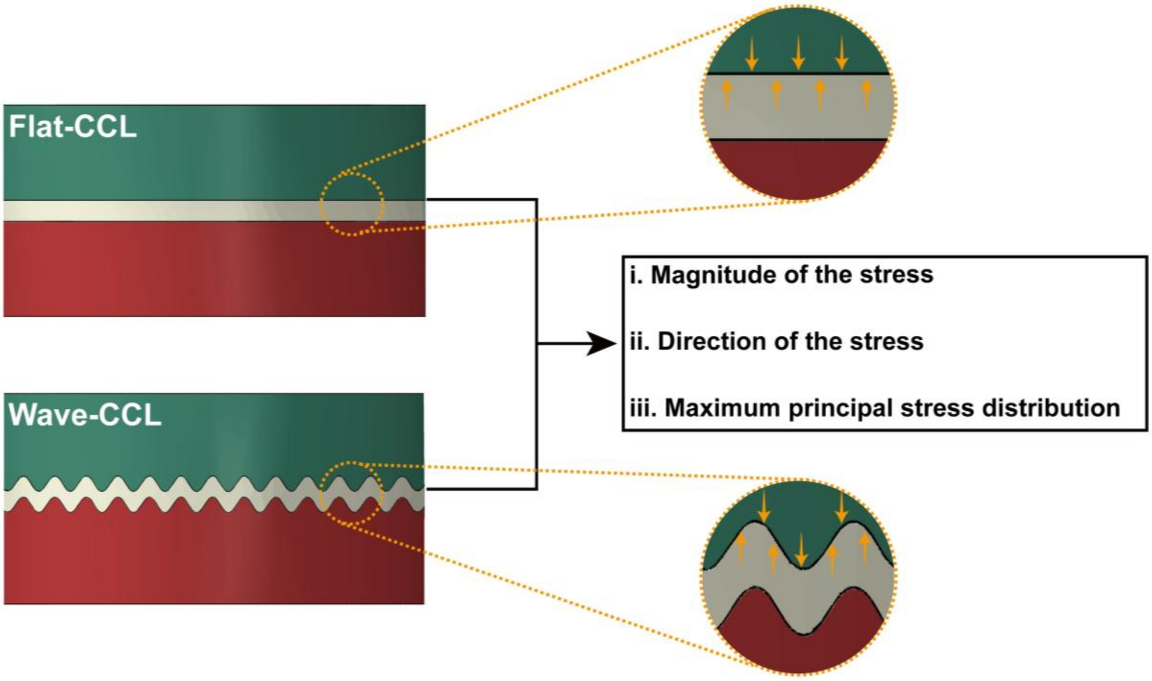

Supplementary Fig. 30 Schematic diagram of analysis area on the lower surface of the cartilage layer. i. Magnitude of the stress. ii. Direction of the stress. iii. Maximum principal stress distribution.

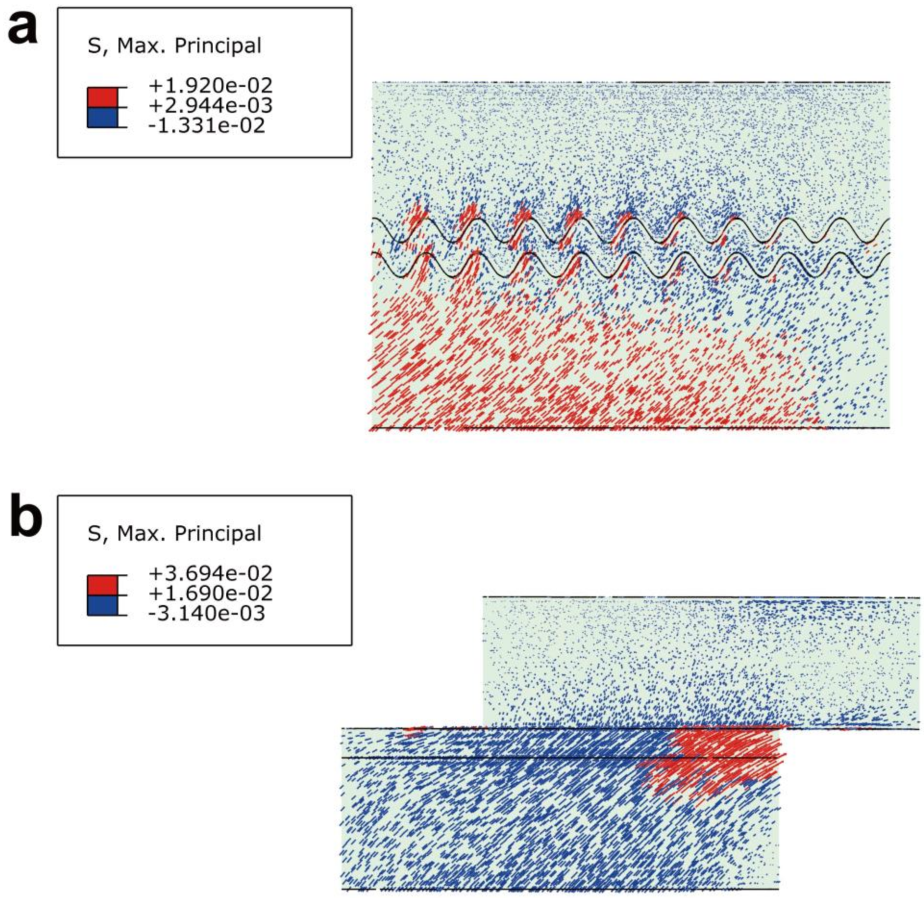

Supplementary Fig. 31 Maximum principal stress distribution of Flat-CCL and Wave-CCL models subjected to shear stress. a) Maximum principal stress in the Wave-CCL model were distributed in the subchondral bone layer. **b)** Maximum principal stress in the Flat-CCL model were distributed at the junction.

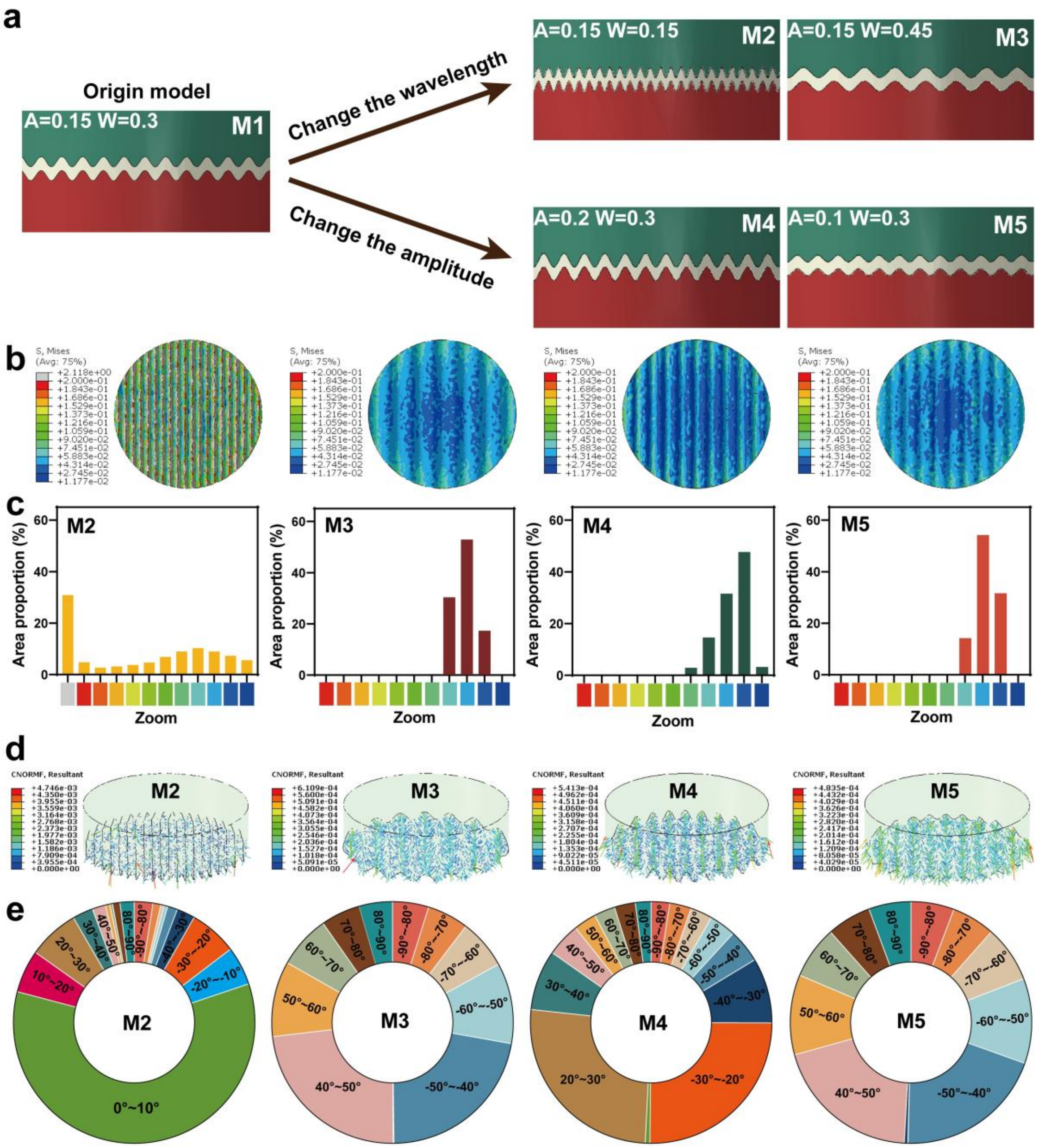

Supplementary Fig. 32 Stress responses of models with different parameters. a) Schematic diagram of models' construction with different parameters. W: Wavelength, A: Amplitude. M: Model. **b)** Thermograms of stress distribution on the lower surface of the cartilage layer when these models were subjected to a compressive stress of 0.1 MPs respectively. **c)** Statistical analysis of the proportion of area occupied by different color blocks in **b)**. **d)** Direction of stress in the lower surface of the cartilage layer when subjected to compressive stress in these models. **e)** Statistical analysis of stress of different ranges in **d)**. Source data are provided as a Source Data file.

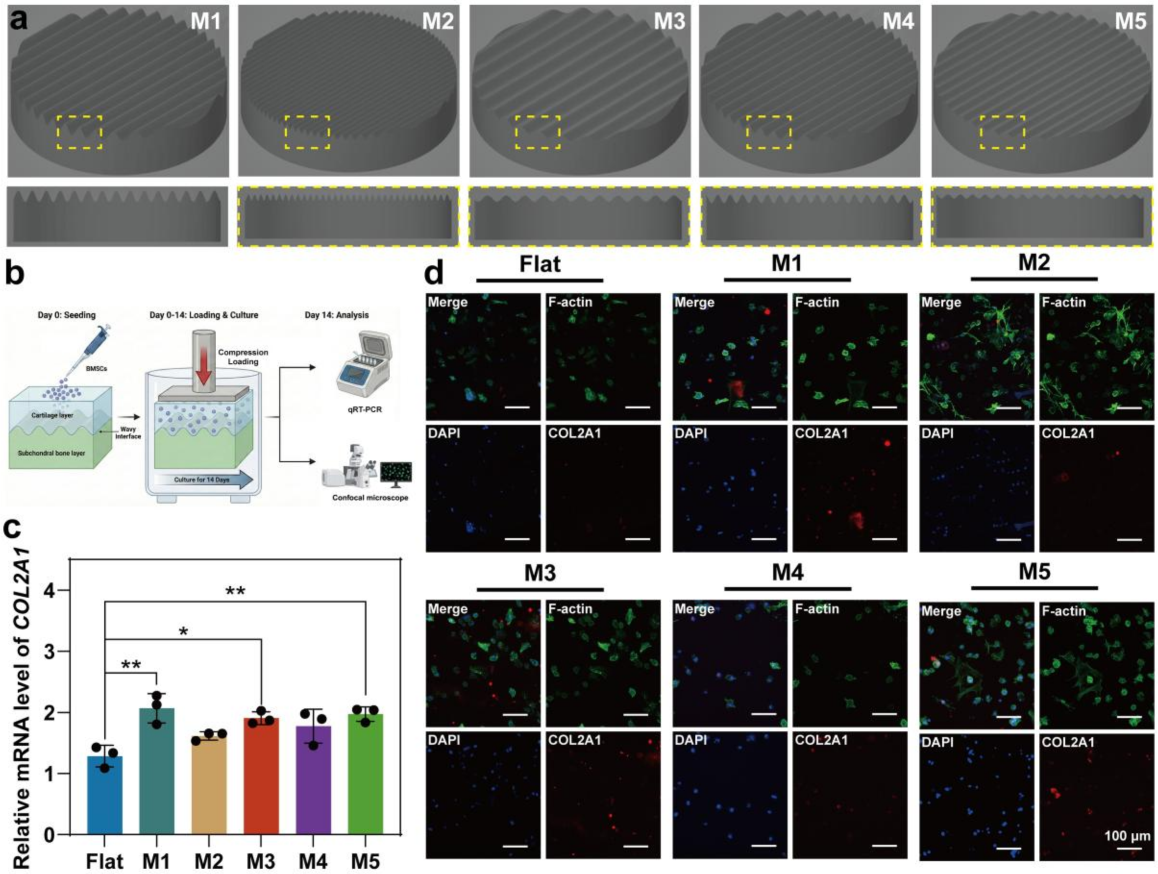

Supplementary Fig. 33 Chondrogenic differentiation of BMSCs cultivated in scaffolds with different parameters. a) Drawings for manufacturing wave-shaped templates with different parameters. **b)** Schematic diagram of the cultivation and analysis of BMSCs. **c)** Relative mRNA expression of *COL2A1* of BMSCs cultured in different scaffolds for 14 days. (**p = 0.0020, *p = 0.0118, **p = 0.0056, n = 3 independent samples). **d)** IF staining of COL2A1 and F-Actin of BMSCs cultured in different scaffolds for 14 days. Data were presented as means ± SD. Statistical significance was determined using the one-way ANOVA with Tukey's post-hoc test. Source data are provided as a Source Data file.

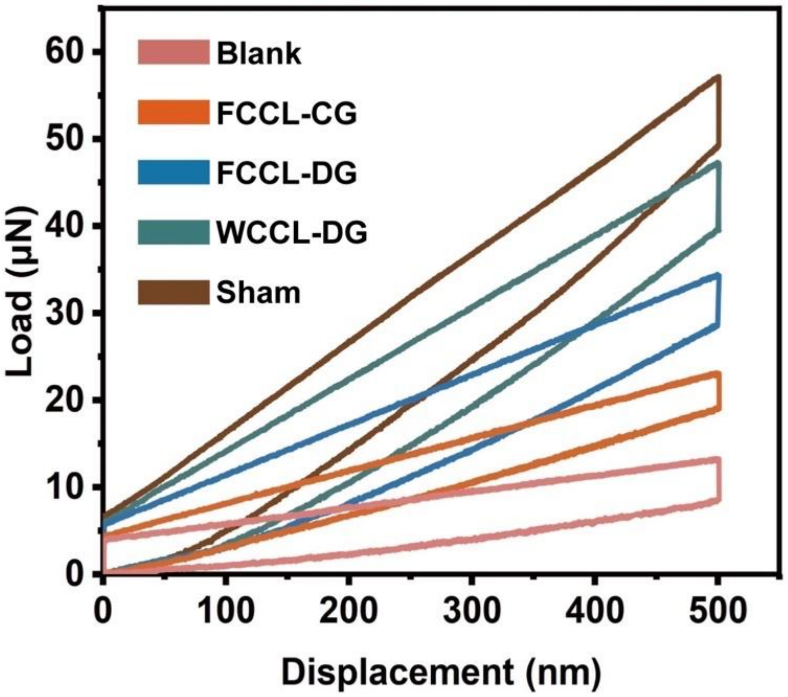

Supplementary Fig. 34 Load-displacement curves of regenerated cartilage by nanoindentation. Source data are provided as a Source Data file.

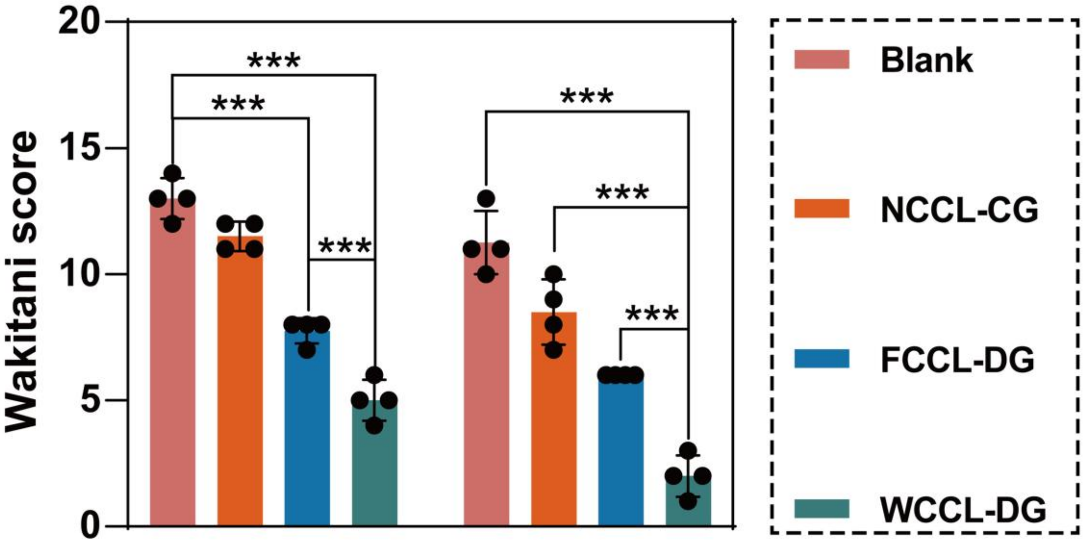

Supplementary Fig. 35 The modified Wakitani scores in terms of cartilage structure, cellular abnormalities, and matrix staining. (****p* = 0.0006, ****p* < 0.0001, ****p* = 0.0005, ****p* < 0.0001, n = 4 independent samples). Data were presented as means ± SD. Statistical significance was determined using the one-way ANOVA with Tukey's post-hoc test. Source data are provided as a Source Data file.

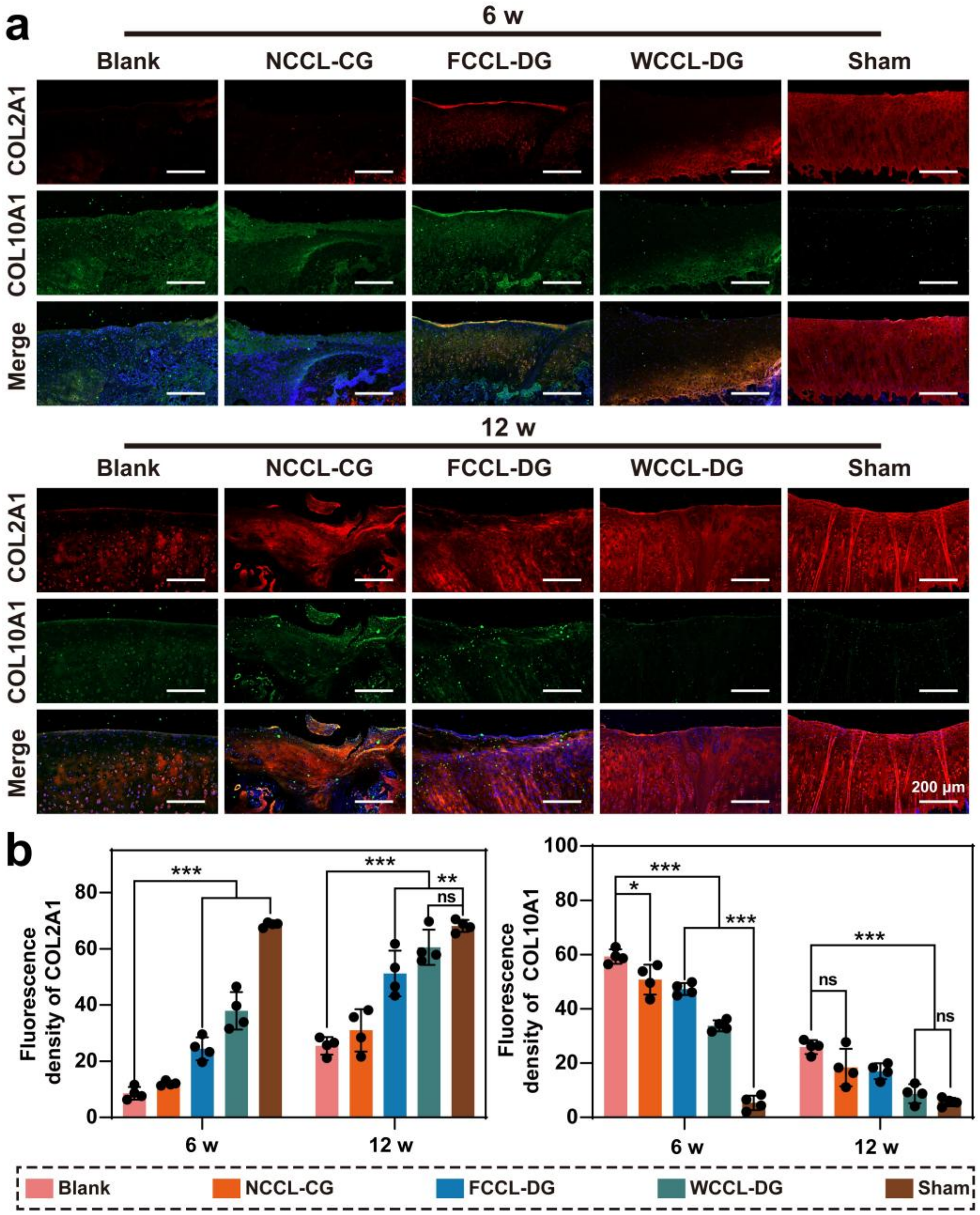

Supplementary Fig. 36 Expression levels of COL2A1 and COL10A1 at specific time points for each group. a) IF staining of COL2A1 and COL10A1. **b)** Quantitative analysis of the fluorescence intensity in **a)**. (for COL2A1: ***$p = 0.0002$, ***$p < 0.0001$, **$p = 0.0082$, ***$p = 0.0002$, ***$p < 0.0001$; for COL10A1: ***$p < 0.0001$, *$p = 0.0161$, ***$p = 0.0009$, ***$p < 0.0001$, ***$p = 0.0001$, ***$p < 0.0001$, n = 4 independent samples). Data were presented as means ± SD. Statistical significance was determined using the one-way ANOVA with Tukey's post-hoc test. Source data are provided as a Source Data file.

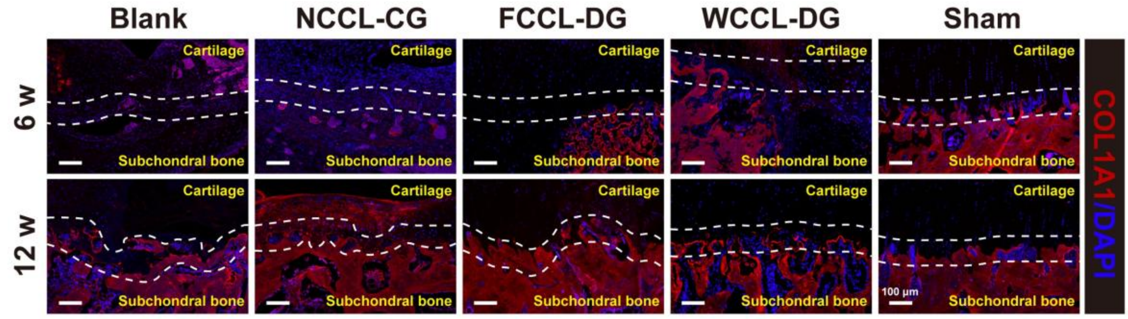

Supplementary Fig. 37 Comparison of the regeneration status of the tidemark at specific time points for each group through IF staining of COL1A1.

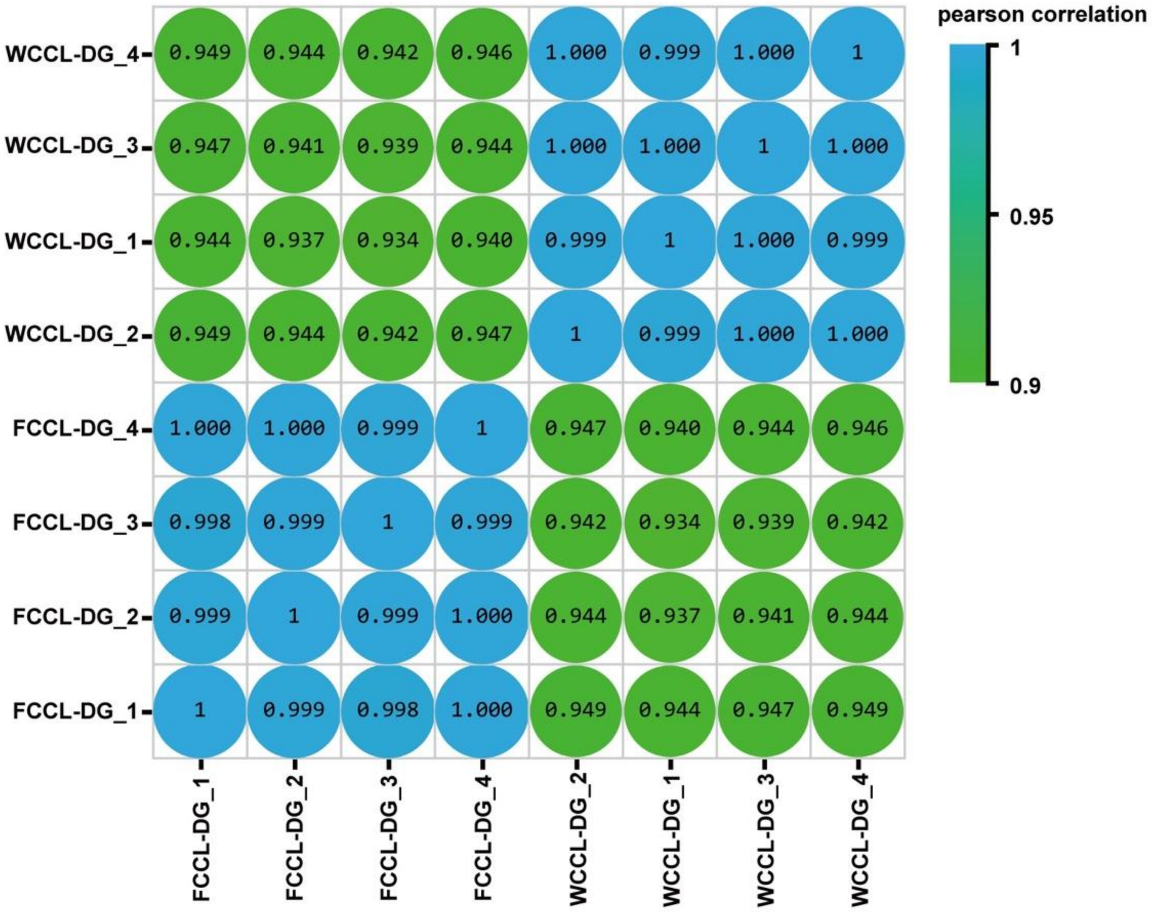

Supplementary Fig. 38 Correlation heat map enriched in DEGs.

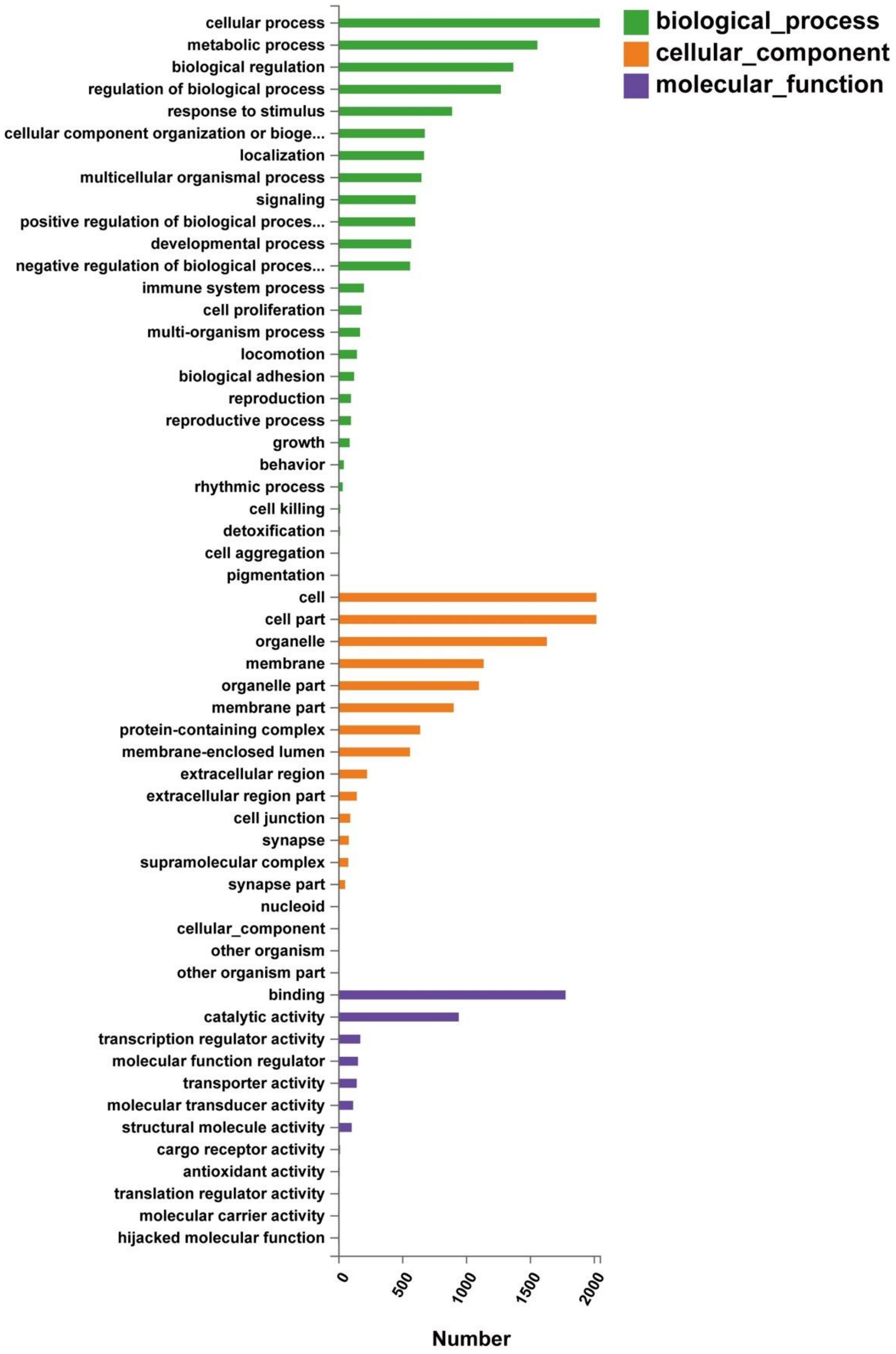

Supplementary Fig. 39 GO Classification of WCCL-DG vs FCCL-DG.

Supplementary Table 1. Naming conventions for hydrogel groups.

Abbreviation	Full name	Does it include the CCL layer and its form	Characteristics
NCCL-CG	None Calcified Cartilage Layer-Composite Gel	No	Double-layer hydrogel, not subjected to ion soaking
FCCL-DG	Flat Calcified Cartilage Layer-Double-network Gel	Yes，flat	Double-layer hydrogel subjected to bidirectional ion soaking
WCCL-DG	Wave Calcified Cartilage Layer-Double-network Gel	Yes，wave	Double-layer hydrogel prepared by a wave-shaped template subjected to bidirectional ion soaking

Supplementary Table 2. Primer sequences of target genes for qRT-PCR.

Genes	Forward (5'-3')	Reverse (3'-5')
ACAN	GGTGGTCTGGACAGGTGCTA	GGTTGGGGTAGAGGTAGACG
COL2A1	CCACGCTCAAGTCCCTCAAC	AGTCACCGCTCTTCCACTCG
SOX9	AGTACCCGCACCTGCACAAC	TACTTGTAGTCCGGGTGGTCTTTC
SDF1A	TGTCTCAGCGATGGGAAACC	TGAGATGCTTGACGTTGGCT
VEGF	ATGCGGATCAAACCTCACCA	GCCCACAGGGATTTTCTTGC
GAPDH	GTATGATTCCACCCACGGCA	CCAGCATCACCCCACTTGAT

Supplementary Table 3. Template parameters.

Name	Amplitude (mm)	Wavelength (mm)
M1 (Origin)	0.15	0.3
M2	0.15	0.15
M3	0.15	0.45
M4	0.2	0.3
M5	0.1	0.3

Supplementary Table 4. ICRS macroscopic evaluation of cartilage repair

Cartilage repair assessment	Item	Points
**Degree of defect repair**	In level with surrounding cartilage	4
	75% repair of defect depth	3
	50% repair of defect depth	2
	25% repair of defect depth	1
	0% repair of defect depth	0
**Integration to border zone**	Complete integration with surrounding cartilage	4
	Demarcating border < 1 mm	3
	3/4th of graft integrated, 1/4th with a notable border > 1 mm width	2
	1/2 of graft integrated with surrounding cartilage, 1/2 with a notable border > 1 mm	1
	From no contact to 1/4th of graft integrated with surrounding cartilage	0
**Macroscopic appearance**	Intact smooth surface	4
	Fibrillated surface	3
	Small, scattered fissures or cracks	2
	Several, small or few but large fissures	1
	Total degeneration of a grafted area	0
**Overall repair assessment**	Grade I: Normal	12
	Grade II: Nearly normal	11-8
	Grade III: Abnormal	7-4
	Grade IV: Severely abnormal	3-1

Supplementary Table 5. Elementary semiquantitative scoring system for grading of histological defect repair.

Wakitani Score

Category	Item	Points
**Cell morphology**	Hyaline cartilage	0
	Mostly hyaline cartilage	1
	Mostly fibrocartilage	2
	Mostly non-cartilage	3
	Non-cartilage only	4
**Matrix staining**	Normal	0
	Slightly reduced	1
	Markedly reduced	2
	No metachromatic stain	3
**Surface regularity (total smooth area compared with entire area of cartilage defect)**	Smooth (> 3/4)	0
	Moderate (> 1/2 - 3/4)	1
	Irregular (1/4 - 1/2)	2
	Severely irregular (< 1/4)	3
**Thickness of cartilage**	> 2/3	0
	1/3 - 2/3	1
	< 1/3	2
**Integration of donor with host adjacent cartilage**	Both edges integrated	0
	One end integrated	1
	Neither edge integrated	2
**Total points**		14
