# Supplementary material for: A decoupling strategy toward spatiotemporal regulation and biomechanical transmission of sandwiched scaffold for osteochondral regeneration
Source: Nat Commun. 2026 Apr 17;17:5351. doi: 10.1038/s41467-026-71810-4 (PMC13272660; doi:10.1038/s41467-026-71810-4)
Supplement: Supplementary file 3 — Transparent Peer Review file [file 41467_2026_71810_MOESM3_ESM.pdf]

nature portfolio

# Peer Review File

# A decoupling strategy toward spatiotemporal regulation and biomechanical transmission of sandwiched scaffold for osteochondral regeneration

Corresponding Author: Professor Xing Wang

**This file contains all reviewer reports in order by version, followed by all author rebuttals in order by version.**

Version 0:

Reviewer comments:

Reviewer #1

(Remarks to the Author)
For osteochondral regeneration, this study presented three points in their biphasic hydrogel design: 1) the wavy tidemark zone; 2) the PAA-CHI-KGN-Mg cartilage zone; 3) the PAA-CHI-Cu bone zone, to tell a regeneration story in terms of strengthened interface, the PAA chelating Fe ion to produce hypoxia for chondrogenesis, the kGN inhibiting hypertrophy, the Cu ion initiating angiogenesis to induce hypertrophy followed by ossification via ECO process. The authors proposed that they have developed an advanced scaffold for osteochondral regeneration, particularly, the introduction of the wavy CCL zone, with in vivo outcomes proving the repair efficiency of the WCCL-DG being better than the FCCL-DG. This study provides some interesting points, while apart from the outcomes, actually, the difference between the WCCL-DG and the FCCL-DG is only one with flat interface and another one with wavy interface. The mechanism why this difference can cause significant difference in cartilage regeneration in vivo, should be further clarified, particularly, the stress distribution on the interface upon their implantation in rabbit femoral trochlea should be analyzed. Before further consideration, the authors are required looking into the following issues.

Comments on the wavy CCL design
1)In Introduction, the authors emphasize the function of CCL in osteochondral structure using Ref. 9 and Ref.10 to support their statements on wavy CCL interfaces, which is not so proper. Both the references did not show a clear wavy morphology at the interface. As the wavy CCL is a key point of this study, the authors are suggested digging into more references on micromorphology related to the CCL.
2)The authors claimed the flaws of laminated osteochondral biphasic or triphasic scaffold being prone to peeling off, they did not cite those works on osteochondral scaffold with continuous gradient without concern on delamination.
3)In Ref.9, the osteochondral interface was identified as an ultrathin ~20-30 um graded calcified region with two layered micronano structures in human knee joint, featured with hydroxyapatite heterogeneity coupled with enrichment of elastic-responsive protein. In this study, a sandwiched scaffold with the total height of 3 mm was prepared for rabbit implantation, and the cartilage-CCL-bone height ratio is 45:10:45, which means the thickness of the CCL interface being 300 um, much deviating from the real case. Why did not the authors consider altering the thickness of the CCL zone for more characterizations on its effect on energy dissipation?
4)The WCCL-DG shows better energy dissipation and mechanical transmission than the FCCL-DG, due to expanded contact area. In this study, the bonding between the upper and lower layer is mainly via hydrogen bond and ionic interaction, without covalent bonding. If a stronger bonding can be achieved between the two layers, is the wavy design necessary?

Comments on the cartilage and bone zone design
5)In Fig. 2f and its corresponding text, the average pore sizes of the PAA-CHI-KGN-Mg and the PAA-CHI-Cu are described as 22.6 and 43.5 um, due to the higher $Mg^{2+}$ concentration. However, from Figure 3d, the SEM images indicated the cartilage layer having larger pore size than the bone layer. Which is correct?
6)The PAA-CHI-KGN-Mg and PAA-CHI-Cu have higher crosslinking density due to the extra metal/carboxyl complexation, leading to higher mechanical strength than the PAA-CHI, then why did they also show higher swelling ratio than the PAA-CHI?
7)According to Fig. S5, please provide the grafting ratio of KGN. In Fig. S11, the KGN release was provided in cumulative percentage, while the released concentration is more meaningful in judging its biofunction.

8)The release of KGN has been monitored for 50 days, does it mean the hydrogel remaining its morphology until this time point? From Figure 8, the residual material is almost not able to be seen at 6 weeks post implantation. It is important to know the degradation behaviors of the WCCL-DG scaffold.
9)The cell cultures were conducted using hydrogel extracts, as well as, the antibacterial test, it is important to know the Mg2+ and Cu2+ release from the hydrogels to understand these outcomes more clearly. In addition, the authors claimed the subchondral bone regeneration via the ECO procedure, therefore, it follows the events of chondrogenic differentiation, angiogenesis, hypertrophy and osteogenesis. If the Cu2+ was released rapidly within short time, it may not match the rhythm. Moreover, the release rates of Mg and Cu ions, also determined the hydrogel integrity.
10)The authors provided antibacterial data in Figure S12, while they did not provide the details of this test in experimental part. In the inhibition zone assay, the hydrogels displaying bacterial-killing ability should be due to the components released from the hydrogels. It is known Cu2+ able to kill bacterial at low concentration, which also brings cytotoxicity at a relatively higher concentration. MgO was reported having the ability to kill bacteria due to it causing peralkaline, however, in the case of PAA-CHI-KGN-Mg, only Mg2+ is released from the hydrogel, why did it achieve such obvious bacterial-killing ability?
11)In Ref.12, it is reported that a proper Mg gradient can induce the dual lineage regeneration of osteochondral defect. On the one hand, can the PAA-CHI-KGN-Mg hydrogel release the Mg2+ at an optimized concentration for chondrogenesis? On the other hand, Mg2+ also has the potential to promote angiogenesis as proved by many published works. Unlike Cu2+， Mg2+ shows significant cytotoxicity only at quite high concentrations. Why did not the authors consider still using Mg2+ in the bone zone?
12)The PAA-CHI-Cu showed slightly higher compression modulus than the PAA-CHI-KGN-Mg, however, they both are quite low (20-30 kPa) at strain less than 30%. The ability to deform at 80-90% strain is not so meaningful for its implantation in the osteochondral defect, where the deformation is only about 10-30%. Can the authors further improve the compression strength of the hydrogels? On the other hand, it is confused to find that the compression stress of the FCCL-DG and WCCL-DG reached 0.16-0.24 MPa at about 60% strain (Fig.3e), while the individual PAA-CHI-KGN-Mg and PAA-CHI-Cu only show less than 0.1 MPa at this strain (Fig. S8). Please explain the reason why their combination able to obtain a significant increase in compression property.
13)To form the Mg and Cu gradient in the hydrogel by soaking, please add some words in the experimental part to clarify the depth of the hydrogel soaking in the magnesium sulfate or copper sulfate solution.

Comments on in vitro cell culture
14)The authors collected BMSCs and chondrocytes for cytotoxicity assay, while in chondrogenic differentiation, they culture BMSCs with chondrogenic inductive medium for 7 days, and added hypertrophy induction medium and KGN to confirm the KGN effect on inhibiting hypertrophy. Though this test has no problem, the question is, why did not the authors directly apply chondrocytes in this test, which may reflect the situation more close to in-situ cases? On the other hand, in the chondrogenic differentiation assay, as shown in Fig. 4a-4c, why was the PAA-CHI-KGN-Mg group not involved?
15)For the PAA-CHI-Cu hydrogel, the authors focused on its vasculogenesis potential, without conducting chondrogenic, hypertrophic and osteogenic differentiation. The authors continuously mentioned ECO in their study, providing only an immunohistological staining on Col-X for regenerated tissues collected from rabbit to show the occurrence of the ECO process, while this is not sufficient. For in vitro evaluations, the authors are suggested to prove the chondrogenic - hypertrophic - osteogenic transition using the PAA-CHI-Cu hydrogel.

Comments on in vivo evaluations
16)The authors applied the rabbit femoral trochlea to perform in vivo evaluations, as well known, this is a defect model without significant loading. The authors found that the WCCL-DG is more sufficient in tissue regeneration than the FCCL-DG, as the only difference between them being the flat interface and wavy interface. The authors are required to clarify the difference in stress distribution on the implanted WCCL-DG and FCCL-DG, particularly at the interface, which is necessary to understand the in vivo outcomes.
17)For the Col-X staining shown in Figure 8a, they are actually hard to be distinguished, which are insufficient to support the ECO process. Immunofluorescent staining on Col-X is suggested. The staining on Col-I is also required. The interface of the regenerated tissue should be enlarged to mark out the tidemark zone for clear comparison.

Comments on mechanism
It is a good point that the authors collected the regenerated tissue at 4 weeks post-surgery for RNA sequencing analysis. However, due to the fact that the difference between the in-situ loading status on the WCCL-DG and FCCL-DG is unclear, the conclusion on the biomechanical mechanisms of wavy interface on promoting cartilage regeneration is not so convincing.

Other comments
Please check the 'Materials' section, it is claimed where the mice and SD rats were bought, but the SD rats should be New Zealand white rabbits.
Please check Fig. 6a, the texts and the images do not match each other.
In Fig.S17a, please replace the 'Ctrl' with 'CHI' for clarity. Scale bars are missing.
Throughout the text, the meaning of the 'FCCL-CG' was not explained.
In Fig.S25, there are two rows of images at the same time point for each group. Please clarify the differences between them in the legend.
In Figure 8a, what are those red arrows pointing to?
In paper writing, it is suggested to put the FEA data together with the rheological outcomes, that they can support each other more clearly without putting the differentiation sections between the two parts.

Reviewer #2

(Remarks to the Author)
The authors developed a sandwich-structured hydrogel scaffold featuring a biomimetic wavy calcified cartilage layer (CCL) via a decoupling strategy for full-thickness osteochondral defect repair. While the structural design of the scaffold shows some novelty and may offer a potential candidate solution for osteochondral regeneration, the manuscript exhibits significant limitations that currently preclude its acceptance for publication. Below are detailed comments for the authors' consideration:

1. The biological strategies involving $Mg2+$, $Cu2+$, and KGN to promote chondrogenesis, regulate vascularization, and inhibit chondrocyte hypertrophy are not entirely novel. The Introduction section fails to adequately highlight how this study advances beyond existing literature. For example, the use of these bioactive components has been previously reported in studies such as:
[1] Zheng L, Zhao S, Li Y, et al. Engineered MgO nanoparticles for cartilage-bone synergistic therapy. Sci Adv. 2024;10(10):eadk6084. doi:10.1126/sciadv.adk6084
[2] Yan J, Xia D, Zhou W, et al. pH-responsive silk fibroin-based CuO/Ag micro/nano coating endows polyetheretherketone with synergistic antibacterial ability, osteogenesis, and angiogenesis. Acta Biomater. 2020;115:220-234. doi:10.1016/j.actbio.2020.07.062
[3] Chen YR, Yan X, Yuan FZ, et al. Kartogenin-Conjugated Double-Network Hydrogel Combined with Stem Cell Transplantation and Tracing for Cartilage Repair. Adv Sci (Weinh). 2022;9(35):e2105571. doi:10.1002/advs. 202105571

2. Although the wavy CCL design is inspired by natural anatomy, the regular waveform template used in this study does not fully recapitulate the irregular and heterogeneous architecture of native CCL. Quantitative morphological parameters (e.g., wavelength, amplitude) of native CCL should be provided and compared with the engineered structure to assess biomimetic fidelity. Future work should incorporate advanced imaging (e.g., micro-CT) and manufacturing techniques (e.g., 3D printing) to achieve higher structural accuracy.

3. The claim that $Fe^{3+}$ chelation by PAA sustainably activates HIF-1α signaling lacks direct molecular evidence. Experiments such as prolyl hydroxylase (PHD) activity assays and quantification of downstream HIF-1α targets (e.g., VEGF, COL-II) are necessary to validate the proposed mechanism.

4. The use of a rabbit femoral trochlear defect model alone is insufficient to demonstrate clinical translatability. Large animal models (e.g., sheep or minipigs) should be employed to evaluate the scaffold's efficacy under physiologically relevant loading conditions. Additionally, the 12-week evaluation period is too short to assess long-term outcomes. Extended studies (6–12 months) are needed to monitor tissue maturation, scaffold degradation, and potential complications (e.g., ectopic calcification, inflammatory responses).

5.The compressive and shear tests provided are inadequate to simulate the complex joint environment. Fatigue testing and tribological assessments should be conducted to evaluate the scaffold's durability and friction properties under cyclic loading.

6. Comparisons with only flat-interface scaffolds (FCCL-DG) are insufficient. A no-CCL control group should be included to directly demonstrate the functional necessity of the wavy CCL.

7. The naming system for hydrogel groups (e.g., WCCL-DG, FCCL-DG, PAA-CHI-KGN-$Mg2+$) is confusing. A table or detailed glossary should be provided to clarify the naming conventions.

8. Some figures do not specify whether multiple comparison corrections (e.g., Tukey's or Bonferroni's tests) were applied. This should be clearly stated in the figure legends and methods section.

9. The current manufacturing process, relying on wavy templates and stepwise soaking, is complex and may lack reproducibility. Process optimization is needed to ensure scalability and standardization for potential clinical translatio.

10. The immunogenic potential of the scaffold and the safety of its degradation products were not evaluated. Comprehensive toxicological studies are essential before clinical application.

11. The Discussion section should be strengthened to directly compare this study's strategies with existing technologies (e.g., 3D-printed gradient scaffolds). The authors should emphasize how their integrated system—combining decoupled spatiotemporal regulation, biomechanical interfacing, and sequential biological processes—surpasses current state-of-the-art approaches.

Reviewer #3

(Remarks to the Author)
I co-reviewed this manuscript with one of the reviewers who provided the listed reports. This is part of the Nature Communications initiative to facilitate training in peer review and to provide appropriate recognition for Early Career Researchers who co-review manuscripts.

Reviewer #4

(Remarks to the Author)
This study presents a bilayer hydrogel scaffoldwith a wavy calcified cartilage layer to mimic the osteochondral interface. Using spatial ion gradients and KGN delivery, the authors direct chondrogenic versus hypertrophic differentiation. Although the work is creative and supported by extensive in vitro and in vivo data, the following key issues require clarification to meet the journal's standards:
1. Please clarify the subcutaneous degradation profile of the hydrogel material. Animal experimental results appear to indicate rapid degradation within 6 weeks, but this does not seem to be the case for PAA and chitosan.
2. For subcutaneous implantation, please provide H&E staining images, preferably with 3-month results to substantiate material degradation and whether cells infiltrate the material.
3. For SEM image (Figure 3a), it is recommended to add false coloring to better illustrate the interface and multilayer structure.
4. The metal ion release experiment in vitro should be supplemented, because the co culture of cells and hydrogels also has good biological effects.
5. Figure 3e could use an Ashby plot to demonstrate performance comparison rather than stress-strain curves from other studies.
6. Finite element analysis should simulate practical application scenarios, such as implantation in osteochondral defects subject to confinement by surrounding tissues.
7. Regarding the advantages of this study compared to others: degradable hydrogels with modulus closer to cartilage have already been reported. The advantages of this work should be elaborated in detail in the Discussion section.
8. The strength and toughness of the hydrogel can be counted, because it seems that strength is a significant advantage, and the modulus is far less than the natural cartilage tissue.

Version 1:

Reviewer comments:

Reviewer #1

(Remarks to the Author)
The authors have addressed most of the concerns and comments in an acceptale way, though some questions are not supplemented with extra data in this revision, the authors pointed out the limitations in the manuscript. Therefore, due to their attempts on waved interfaces and tissue-specific hydrogel compositions for osteochondral repair, it shows the value for publication.

Reviewer #2

(Remarks to the Author)
The revisions provided by the authors have improved the manuscript, and we appreciate their response to the reviewers' comments.
However, the manuscript still falls short of the level of completeness and clarity required for publication in this journal. Several important aspects, such as experimental design, data interpretation, and writing presentation, need further attention. We therefore recommend another round of major revisions, with a focus on the points detailed below. We are confident that these changes will enhance the manuscript’s quality and impact.

1. The article does not provide precise geometric parameters of the template (such as wavelength, amplitude), materials, or preparation methods, which may affect the reproducibility of the experiments.
2. Although "control of the soaking depth" is mentioned, no specific control methods (e.g., visual systems, mechanical positioning) are described, nor is the uniformity of ion diffusion during soaking or batch-to-batch variability evaluated.
3. The influence of wave geometric parameters (e.g., curvature, thickness) on stress distribution and cellular behavior is not explored.
4. The scale bars in the upper and lower groups of supplementary Fig. 7 are inconsistent.
5. How were the hydrogels sterilized?
6. The Methods section lacks the experimental procedure for measuring ion release from PAA-CHI-20 hydrogels after different ionic soaking treatments.
7. Bacterial specific terms and abbreviations should be italicized (line 292).
8. Some figure and caption annotations are unclear (e.g., certain explanations in Fig. 1a are missing and are placed below Fig. 1b, affecting readability).
9. The manuscript would benefit from grammatical polishing.

Reviewer #3

(Remarks to the Author)
I co-reviewed this manuscript with one of the reviewers who provided the listed reports. This is part of the Nature Communications initiative to facilitate training in peer review and to provide appropriate recognition for Early Career Researchers who co-review manuscripts.

Reviewer #4

(Remarks to the Author)
Thank you for submitting your revised manuscript and for your thorough responses to the reviewers' comments. I have carefully reviewed your detailed point-by-point responses and the corresponding revisions made to the manuscript. Therefore, based on the substantial improvements made to the manuscript, I recommend Acceptance of this manuscript for publication in its current form.

Version 2:

Reviewer comments:

Reviewer #2

(Remarks to the Author)
Thank you for submitting the revised manuscript and providing a detailed response to the reviewers' comments. I have carefully reviewed the corresponding revisions. Given the improvements made, I now recommend accepting the manuscript for publication.

Reviewer #3

(Remarks to the Author)
I co-reviewed this manuscript with one of the reviewers who provided the listed reports. This is part of the Nature Communications initiative to facilitate training in peer review and to provide appropriate recognition for Early Career Researchers who co-review manuscripts.

**Open Access** This Peer Review File is licensed under a Creative Commons Attribution 4.0 International License, which permits use, sharing, adaptation, distribution and reproduction in any medium or format, as long as you give appropriate credit to the original author(s) and the source, provide a link to the Creative Commons license, and indicate if changes were made.
In cases where reviewers are anonymous, credit should be given to 'Anonymous Referee' and the source.
The images or other third party material in this Peer Review File are included in the article's Creative Commons license, unless indicated otherwise in a credit line to the material. If material is not included in the article's Creative Commons license and your intended use is not permitted by statutory regulation or exceeds the permitted use, you will need to obtain permission directly from the copyright holder.
To view a copy of this license, visit https://creativecommons.org/licenses/by/4.0/

# A point-by-point response to the reviewers

Dear reviewers:

Thank you very much for your kind advice and valuable comments in helping us improve our m anuscript (Title: “A decoupling strategy toward spatiotemporal regulation and biomechanica l transmission of sandwiched scaffold for full-thickness osteochondral regeneration”). We have s ubstantially modified the manuscript, according to the questions raised by the reviewers. All the modified words, sentences and paragraphs were labeled with red fonts. A point-to-point response t o highlight how we have addressed each of the comments is listed below.

**Reviewer #1 (Remarks to the Author):**

For osteochondral regeneration, this study presented three points in their biphasic hydrogel design: 1) the wavy tidemark zone; 2) the PAA-CHI-KGN-Mg cartilage zone; 3) the PAA-CHI-Cu bone zone, to tell a regeneration story in terms of strengthened interface, the PAA chelating Fe ion to produce hypoxia for chondrogenesis, the KGN inhibiting hypertrophy, the Cu ion initiating vasculogenesis to induce hypertrophy followed by ossification via ECO process. The authors proposed that they have developed an advanced scaffold for osteochondral regeneration, particularly, the introduction of the wavy CCL zone, with *in vivo* outcomes proving the repair efficiency of the WCCL-DG being better than the FCCL-DG. This study provides some interesting points, while apart from the outcomes, actually, the difference between the WCCL-DG and the FCCL-DG is only one with flat interface and another one with wavy interface. The mechanism why this difference can cause significant difference in cartilage regeneration *in vivo*, should be further clarified, particularly, the stress distribution on the interface upon their implantation in rabbit femoral trochlea should be analyzed. Before further consideration, the authors are required looking into the following issues.

Comments on the wavy CCL design

1) In Introduction, the authors emphasize the function of CCL in osteochondral structure using Ref. 9 and Ref.10 to support their statements on wavy CCL interfaces, which is not so proper. Both the references did not show a clear wavy morphology at the interface. As the wavy CCL is a key point of this study, the authors are suggested digging into more references on micromorphology related to the CCL.

**Reply 1:** Thanks for your kind suggestions. We have re-examined the relevant literatures related to the microscopic morphology of CCL in a systematic manner, particularly focusing on the research on its interface structural characteristics. We have also supplemented the latest research that can clearly support the wave-shaped structure of CCL in the revised introduction section, in order to comprehensively and accurately explain the physiological significance of the wave-shaped morphology of CCL at the natural chondro-osseous junction, thereby providing a more solid theoretical basis for the rationality of the WCCL design in our research. Once again, we thank you for your valuable suggestions. We firmly believe this modification will significantly enhance the

accuracy and persuasiveness of the introduction section of this study.

Revision: "Structurally, the characteristic of CCL lies in the presence of a distinct wavy pattern on its upper boundary. This pattern appears as raised eosinophilic lines with a thickness of 10 μm under an optical microscope. Moreover, this pattern has a 3D undulating shape consisting of alternating peaks and valleys, forming a dynamic adaptive structure that becomes more pronounced when subjected to mechanical stimuli such as long-term weight-bearing or movement[14, 15]."

2) The authors claimed the flaws of laminated osteochondral biphasic or triphasic scaffold being prone to peeling off, they did not cite those works on osteochondral scaffold with continuous gradient without concern on delamination.

**Reply 2:** Thanks for your kind suggestions. Regarding this issue, we have added a targeted elaboration on the recent continuous gradient osteochondral scaffold research in the introduction, and detailedly introduced the technical advantages of such scaffolds through continuous transition design of components, structures, or performances in reducing the risk of inter-layer peeling. This was done to present more comprehensively the significant progress in the field of osteochondral scaffolds, which aimed to address the shortcomings of traditional layered scaffolds. Additionally, we also further clearly pointed out in the introduction that although continuous gradient scaffolds effectively improved the inter-layer bonding problem, most current studies still failed to construct a CCL with key mechanical conductivity and tissue isolation functions in the scaffold structure. Due to the absence of this core structure, these scaffolds still had certain limitations compared to the scaffolds proposed in our study that incorporated a biomimetic wave-shaped CCL. This was because the lack of this core structure made these scaffolds less capable of precisely simulating the gradient mechanical properties of natural osteochondral tissue, guiding the specific differentiation of cartilage and bone tissues, and maintaining the stability of the interface over the long term.

Revision: "In recent years, the emergence of continuous gradient scaffolds effectively avoids interlayer peeling, provides a smoother transition interface, and thereby improves the biomechanical properties and repair outcomes. For example, Zhang et al. proposed a hydrogel scaffold that can achieve continuous biophysical and biochemical gradients between cartilage and subchondral bone, thereby effectively simulating the natural mineral and mechanical gradients[11]. Xu et al. designed a mechanical-magnetic gradient hydrogel to achieve a continuous increase in the mechanical modulus from the cartilage layer to the subchondral bone layer, thereby coordinating differentiation by regulating the gradient expression of YAP1[12]."

3) In Ref.9, the osteochondral interface was identified as an ultrathin ~20-30 um graded calcified region with two layered micronano structures in human knee joint, featured with hydroxyapatite heterogeneity coupled with enrichment of elastic-responsive protein. In this study, a sandwiched scaffold with the total height of 3 mm was prepared for rabbit implantation, and the cartilage-CCL-bone height ratio is 45:10:45, which means the thickness of the CCL interface being 300 um, much deviating from the real case. Why did not the authors consider altering the thickness of the CCL zone for more characterizations on its effect on energy dissipation?

**Reply 3:** Thanks for your kind suggestions. Regarding the related questions concerning the design of the CCL interface, based on the characteristics and technical feasibility of the experimental model, we have provided the following supplementary explanations for the core issues. The physiological thickness of the CCL in rabbit knee joints is only in the single-digit micrometer range, significantly smaller than the 20-30 μm in humans (Nano Lett. 2022, 22, 2309). Actually, this extremely small scale is incompatible with the technical contradiction of the bidirectional soaking strategy. Preliminary experimental results show that even if the target thickness is increased to 100 μm, the interface thickness error still reaches ± 30 μm, and the thickness consistency among different batches of scaffolds is extremely poor. When the target thickness drops to <10 μm, our bidirectional soaking strategy cannot overcome the surface tension of the solution and the anisotropy of diffusion, and theoretically cannot achieve the required micrometer-level accuracy for the experiment. Therefore, the selection of CCL thickness in this study is a necessary compromise under the constraints of technical feasibility and model size. Furthermore, ref.9 also mentioned that the natural CCL was not a uniform thin layer, but rather presented a 3D irregular "mineralized nodules - collagen fibers interwoven" network structure, such as irregular topological interfaces and vertically penetrating fiber bundles. The current template method can only achieve macroscopic layering but not reproduce above microscopic morphology, thus resulting in the limited morphological biomimicry. This simplification affects the precise simulation of the interface mechanical microenvironment, but in the rabbit model, it is a phased choice prioritizing functional verification over detailed biomimicry, and has taken an important step in the preparation of a wavy interface based on existing traditional research.

However, your suggestion regarding the relationship between thickness and energy dissipation has provided us with very valuable insights. We are also actively considering solutions through collaborative optimization of model upgrades and technological innovations, hoping to make some breakthroughs in our future research. For example, in terms of animal models, we intend to use large animals such as goats or pigs to construct knee joint osteochondral defect models, as their CCL thickness is closer to the physiological range of humans. Compared to rabbit models, this can significantly reduce the influence of size differences on experimental results, and their anatomical structure and load-bearing pattern are also more analogous to the physiological characteristics of human joints. In addition, the femoral trochlea of large animals can accommodate an implanted scaffold with a diameter of 8-10 mm, which can simulate the common scale of osteochondral injuries in clinical practice and is more suitable for evaluating the integration ability and long-term mechanical stability of scaffolds with CCL interfaces during defect repair. In terms of technological innovation, we will rely on high-resolution 3D imaging technology to analyze the microscopic topological structure and mineralization gradient characteristics of natural CCL. Combined with high-precision manufacturing technologies such as 3D bioprinting, we will achieve the biomimetic construction of natural CCL. By adjusting the composition and microstructure to replicate the structural features and interface mechanical properties of the original CCL, we will ultimately

establish a preparation system that combines structural biomimicry and functional stability, providing more valuable experimental basis for clinical translation.

4) The WCCL-DG shows better energy dissipation and mechanical transmission than the FCCL-DG, due to expanded contact area. In this study, the bonding between the upper and lower layer is mainly via hydrogen bond and ionic interaction, without covalent bonding. If a stronger bonding can be achieved between the two layers, is the wavy design necessary?

**Reply 4:** Thanks for your kind suggestions. The question you raised regarding "whether the wave design is still necessary if a stronger inter-layer bonding is achieved" is precisely the core issue that we focused on during our research. In fact, the key significance of the WCCL design goes far beyond merely enhancing the bonding effect by increasing the contact area. More importantly, through the unique geometric features of the wavy interface, it fundamentally changes the mechanical transmission mode and achieves a biomimetic simulation of the biomechanical function of the natural chondro-osseous junction. Therefore, even if in the future the inter-layer chemical bonding force is further strengthened through strategies such as covalent bonds, its unique advantages remain irreplaceable.

The wavy tideline structure at the junction of natural bone-cartilage interface is a mechanically optimized design through long-term evolution. Its core function is not merely to enhance physical adhesion, but to maintain the interface stability during joint movement through multi-directional stress dispersion and gradient energy dissipation. Finite element analysis shows that under compressive load, the stress direction distribution of the cartilage layer in WCCL-DG is significantly wider than that in FCCL-DG, which is more conducive to promoting the vitality of chondrocytes. This characteristic cannot be achieved merely by enhancing the binding force through covalent bonds. Meanwhile, during joint movement, in addition to the static binding strength, the influence of cyclic shear stress and impact loads on interface stability is also more critical. WCCL disperses shear stress to the subchondral bone layer through geometric interlocking effect, while FCCL, even though the enhancement of binding force through covalent bonds, still increases the risk of cartilage layer peeling due to the interface stress concentration. Moreover, the larger hysteresis loop area and wider range of linear viscoelastic region of WCCL enable it more adaptable to dynamic loads in physiological activities.

Consequently, the core value of this wave-shaped design lies in achieving precise replication of biomechanical functions through structural mimicry, rather than relying solely on chemical binding forces. Even if the interlayer bonding is further enhanced through strategies such as covalent bonds in the future, the unique advantages of WCCL in stress dispersion, dynamic load adaptation, and tissue regeneration regulation remain irreplaceable and are key design elements for achieving long-term functional repair of osteochondral defects.

Comments on the cartilage and bone zone design

5) In Fig. 2f and its corresponding text, the average pore sizes of the PAA-CHI-KGN-Mg and the PAA-CHI-Cu are described as 22.6 and 43.5 um, due to the higher $Mg^{2+}$ concentration. However,

from Figure 3d, the SEM images indicated the cartilage layer having larger pore size than the bone layer. Which is correct?

**Reply 5:** Thanks for your kind suggestions. We are deeply sorry for our oversight regarding this detail. Your meticulous observation helped us identify the key issue with the chart annotations. We attach great importance to this and have conducted a thorough verification and correction. Regarding the correct situation of the pore size, the following clarification is provided below.

As shown in Fig. 3a, the upper layer is the cartilage layer and the lower layer is the bone layer. According to the actual data, the pore size of the bone layer is indeed larger than that of the cartilage layer, which is consistent with the description of the average pore size of PAA-CHI-KGN-$Mg^{2+}$ (22.6 μm) and PAA-CHI-$Cu^{2+}$ (43.5 μm) in the manuscript. Due to the failure to accurately mark the corresponding positions of cartilage layer and bone layer in the figure, we have clearly added the labels of "Cartilage" layer and "Bone" layer to ensure a clear correspondence with the data to prevent any further misunderstandings. Furthermore, we have also conducted a systematic review of the manuscript that involve the description of chart data to eliminate such mistakes. In the future, we will strengthen the multi-round cross-review to ensure the accuracy of our research results.

Revision:

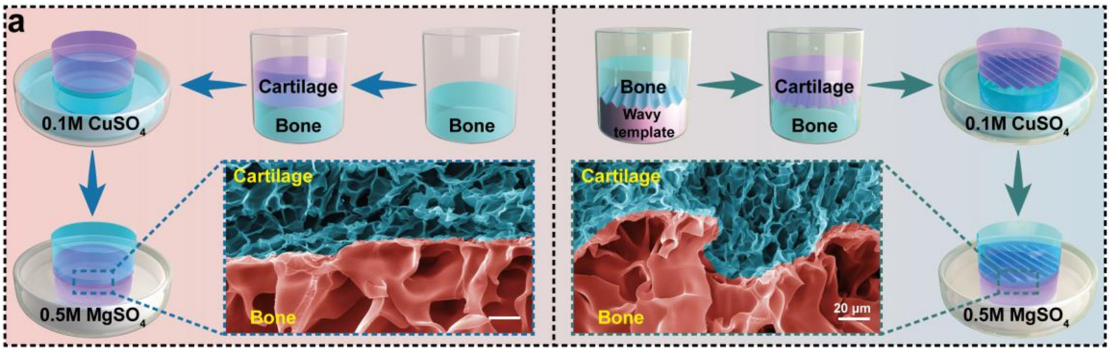


**Fig. 3 a)** Preparation process of FCCL-DG and WCCL-DG scaffolds with structural variance at the junctions.

6) The PAA-CHI-KGN-Mg and PAA-CHI-Cu have higher crosslinking density due to the extra metal/carboxyl complexation, leading to higher mechanical strength than the PAA-CHI, then why did they also show higher swelling ratio than the PAA-CHI?

**Reply 6:** Thanks for your kind suggestions. The swelling behavior of metal-coordinated PAA-based hydrogels is regulated by multiple intermolecular interactions. On the one hand, the coordination bonds formed between $Mg^{2+}$/$Cu^{2+}$ and the carboxyl groups of the PAA side chains constitute a secondary crosslinking network. Through the metal-ligand coordination, the crosslinking density of the network is enhanced, which is consistent with the experimental results showing an increase in mechanical strength. On the other hand, the introduction of metal cations significantly disrupts the original hydrogen bond association between PAA molecular chains. By competing for binding sites, the carboxyl groups are released from the intramolecular and intermolecular hydrogen bond interactions. When the hydrogel is placed in an aqueous environment, the metal-carboxyl coordination bonds undergo dynamic dissociation under the solvation effect. The released carboxyl

anions are driven to expand the conformation of the polymer chain segments by electrostatic repulsion, while the dissociated hydrated metal ions promote water molecule permeation through the osmotic pressure effect, jointly leading to an increase in the swelling ratio.

It is particularly important to note that in this experiment, a low concentration of metal ion solution and a short-term soaking treatment process were adopted, resulting in a relatively low metal coordination conversion rate of carboxyl groups. Under these conditions, the enhancing effect of metal coordination on network cross-linking failed to fully counteract its promoting effect on hydrophilicity and electrostatic repulsion. Therefore, a slight increase in swelling ratio was observed. Theoretically, if the carboxyl groups were fully coordinated, a significant increase in cross-linking density would dominate the swelling behavior, potentially leading to a decrease in the swelling ratio. Within the low coordination degree range, the contributions of electrostatic repulsion and solvation effects are dominant, resulting in an increase in swelling ratio with the increase in the amount of metal ion addition.

Once again, we would like to thanks for your professional questioning. This issue has enabled us to deepen our understanding of the structure-activity relationship of metal coordination hydrogels. The revised version has supplemented the explanation of the above molecular mechanism, ensuring that the experimental results are logically consistent with the mechanism explanation. We have deeply realized that the mechanism analysis of abnormal experimental phenomena is the key to enhancing the depth of the research. This modification will significantly enhance the academic rigor of the manuscript.

Revision: "The $Mg^{2+}$/$Cu^{2+}$ could disrupt certain hydrogen bonds between PAA chains to expose carboxyl groups, and these dynamic dissociation of metal-carboxyl complexes in aqueous environment increased charge repulsion and water molecule infiltration The released carboxyl anions were driven to expand the conformation of the polymer chain segments by electrostatic repulsion, while the dissociated hydrated metal ions promoted the water molecule permeation through the osmotic pressure effect, jointly leading to an increase in the swelling ratio. It was particularly important to note that in this experiment, a low concentration of metal ion solution and a short-term soaking treatment process were adopted, resulting in a relatively low metal coordination conversion rate of carboxyl groups. In this case, the enhancing effect of metal coordination on network cross-linking failed to fully counteract its promoting effect on hydrophilicity and electrostatic repulsion. Reasonably, PAA-CHI-KGN-$Mg^{2+}$ and PAA-CHI-$Cu^{2+}$ hydrogels exhibited higher swelling ratio than that of the PAA-CHI hydrogel."

7) According to Fig. S5, please provide the grafting ratio of KGN. In Fig. S11, the KGN release was provided in cumulative percentage, while the released concentration is more meaningful in judging its biofunction.

**Reply 7:** Thanks for your valuable suggestion. The grafting ratio of KGN on the PAA-CHI-KGN-$Mg^{2+}$ hydrogel is ca. 4.9% by verification of $^{1}H$ NMR analysis (Supplementary Fig. 6). In addition, we have converted the cumulative release percentage to actual concentration accordingly in the

revised Supplementary Fig. 14. It was found that the release concentration of KGN throughout the entire release period remained within the effective *in vivo* concentration range of 100 nM to 100 μM. The concentration corresponding to the cumulative release of 68.1% at 7 weeks also satisfied this effective range, indicating that the released KGN drug was able to exert the expected biological effects *in vivo*.

Revision: "Furthermore, we evaluated the KGN release profile from the PAA-CHI-KGN-$Mg^{2+}$ hydrogel in PBS solutions (Supplementary Fig. 14), which showed that the release rate of KGN increased from 4.4% to 68.1% from the 1st to the 7th week, corresponding to concentrations ranging from 5.1 μM to 73.9 μM, indicating that KGN could consistently meet the effective usage concentration requirement *in vivo* (100 nM–100 μM)[40]."

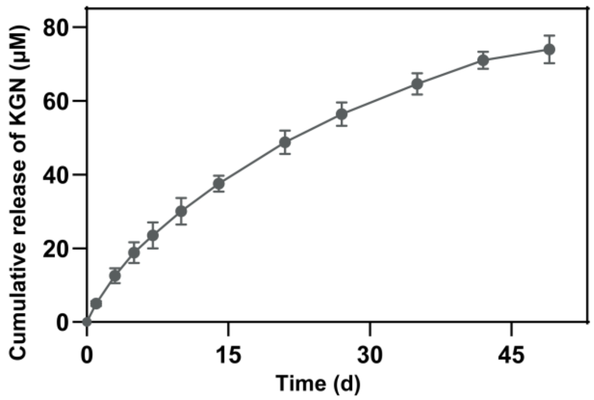


**Supplementary Fig. 14** Cumulative release of KGN from the PAA-CHI-KGN-$Mg^{2+}$ hydrogel. Data were presented as means ± SD, n = 3.

8) The release of KGN has been monitored for 50 days, does it mean the hydrogel remaining its morphology until this time point? From Figure 8, the residual material is almost not able to be seen at 6 weeks post implantation. It is important to know the degradation behaviors of the WCCL-DG scaffold.

**Reply 8:** We sincerely appreciate your valuable suggestions. We have conducted additional *in vivo* degradation experiments to confirm that the remaining mass of both hydrogels was 40%-50% after 6 weeks of subcutaneous implantation (Supplementary Fig. 20). Compared to *in vitro* degradation and *in vivo* subcutaneous degradation, the faster degradation rate in joint cavity was mainly due to the following reasons. Firstly, the encapsulation by the fibrous capsule after subcutaneous implantation inevitably slowed down the degradation rate of the hydrogel scaffold. Additionally, there were many degradable enzymes (such as collagenase and matrix metalloproteinase) within the joint, which further accelerated the decomposition of hydrogel scaffold. More importantly, in order to further demonstrate the biological function of the wavy CCL, we encouraged the rabbits to actively engage in running and jumping exercises after the surgery. This would exert pressure on the hydrogel scaffold, further accelerating its degradation process. We have included these detailed degradation data accordingly in the revised manuscript.

Revision: "To verify the *in vivo* biocompatibility and degradability of different scaffolds, we conducted subcutaneous implantation tests in rats (Supplementary Fig. 18). Since WCCL-DG and

FCCL-DG only differed in structure, we selected WCCL-DG for further investigation. After subcutaneous implantation for one month, we did not observe any obvious tissue damage or morphological changes, and the various indicators showed no significant differences compared to those of normal rats (Supplementary Fig. 19), indicating good biocompatibility of those scaffolds. For the *in vivo* degradation behavior of WCCL-DG and NCCL-CG, both scaffolds exhibited a measurable weight gain attributed to their swelling response in the physiological microenvironment during the first week post-implantation. Subsequently, they exhibited a trend of continuous degradation, manifested as a gradual decrease in weight and a corresponding reduction in volume during the subsequent observation period (Supplementary Fig. 20). Interestingly, we found that the degradation rate of the hydrogel scaffold in the joint cavity was faster compared to the subcutaneous degradation. This was because the encapsulation effect of the fibrous capsule after subcutaneous implantation inevitably slowed down the degradation rate of the hydrogel scaffolds. Additionally, there were many degradable enzymes (such as collagenase and matrix metalloproteinase) within the joint, which further accelerated the decomposition of the hydrogel scaffolds. More importantly, in order to further demonstrate the biological function of the wavy CCL, we encouraged the rabbits to actively engage in running and jumping exercises after the surgery. This would exert pressure on the hydrogel scaffold, further accelerating its degradation process."

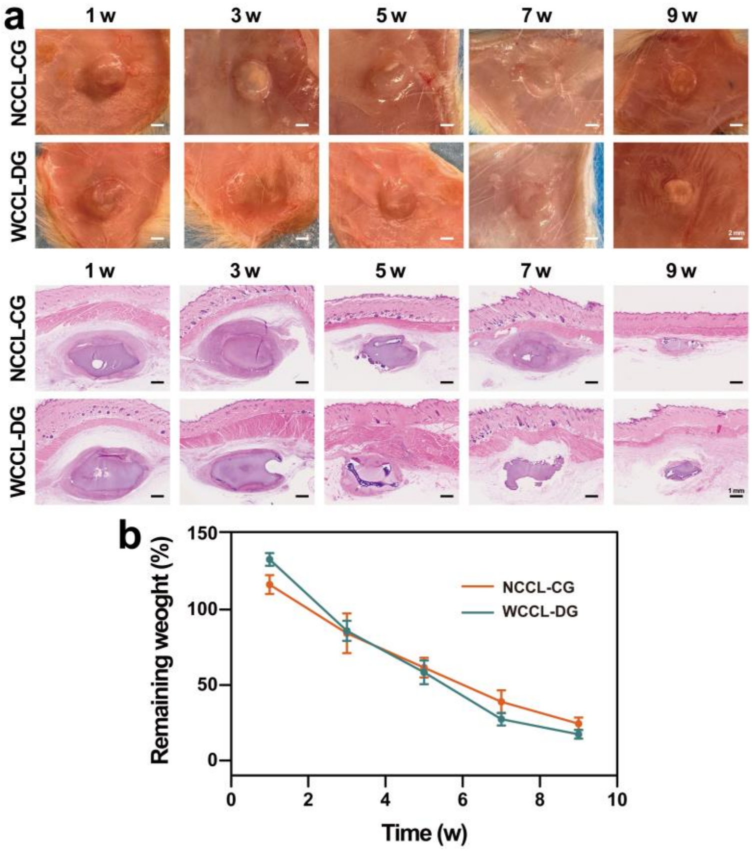


**Supplementary Fig. 20 Subcutaneous degradation behaviors of WCCL-DG and NCCL-CG scaffolds. a)** Macrograph and H&E staining images of WCCL-DG and NCCL-CG scaffolds with the surrounding skin at the different time point. **b)** The mass remained of WCCL-DG and NCCL-CG scaffolds at the different time point. Data in **b)** were presented as means ± SD, n = 3.

9) The cell cultures were conducted using hydrogel extracts, as well as, the antibacterial test, it is important to know the $Mg^{2+}$ and $Cu^{2+}$ release from the hydrogels to understand these outcomes more clearly. In addition, the authors claimed the subchondral bone regeneration via the ECO procedure, therefore, it follows the events of chondrogenic differentiation, angiogenesis, hypertrophy and osteogenesis. If the $Cu^{2+}$ was released rapidly within short time, it may not match the rhythm. Moreover, the release rates of Mg and Cu ions, also determined the hydrogel integrity.

**Reply 9:** Thanks for your valuable insights regarding the release of $Mg^{2+}$ and $Cu^{2+}$ from the hydrogels. This information is crucial for elucidating the biological effects and the structural integrity of the hydrogel. Firstly, we conducted *in vitro* release experiments to study the release behaviors of $Mg^{2+}$ and $Cu^{2+}$ from the hydrogel. As described in Supplementary Fig. 5, the carboxyl groups of PAA form strong ionic interactions with $Mg^{2+}$ and $Cu^{2+}$, allowing these ions to continuously and controllably release over a long period of time rather than releasing in large quantities all at once. This is crucial for matching the biological rhythm of osteochondral regeneration and maintaining the stability of hydrogel scaffolds.

For $Mg^{2+}$, the cartilage layer hydrogel can release $Mg^{2+}$ in a sustainable manner. By screening the soaking concentration, we achieve an optimal $Mg^{2+}$ release level to promote chondrogenic differentiation of BMSCs (200 ppm), this sustained stimulation has been verified via *in vitro* experiments. For $Cu^{2+}$, due to the stronger coordination ability of $Cu^{2+}$ with PAA carboxyl groups compared to $Mg^{2+}$ and the lower soaking concentration of the subchondral bone layer, the release rate of $Cu^{2+}$ is significantly slower than that of $Mg^{2+}$. The *in vitro* results indicate that $Cu^{2+}$ also possesses the ability for sustainable release. Furthermore, this sustained release aligns with the rhythm of the ECO process in the subchondral bone layer as confirmed by our tissue section staining results. At 6 weeks post-operation, chondrocytes enter the hypertrophic cartilage stage (Fig. 8a and Supplementary Fig. 34), where the $Cu^{2+}$ release concentration exactly reaches the optimal level for vasculogenesis (Supplementary Fig. 5), thus promoting the transformation of hypertrophic chondrocytes to osteoblasts. It supports this process well rather than disrupting it through rapid and sudden release. The detailed ion release curves and corresponding discussions have been added accordingly in the revised manuscript.

Revision:

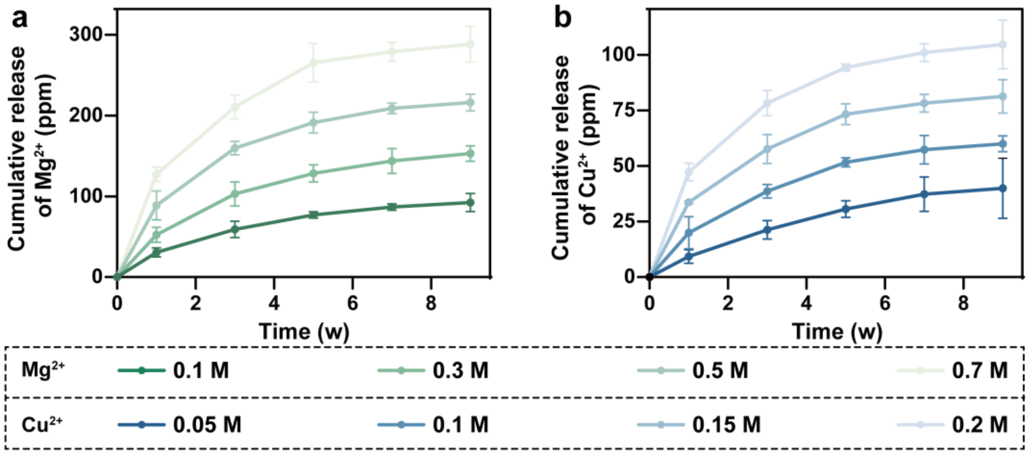

**Supplementary Fig. 5 The ion release curves from PAA-CHI-20 hydrogels after various ionic soaking treatment, respectively.** a) The release curves of $Mg^{2+}$ from the PAA-CHI-20 hydrogels after soaking into $MgSO_4$ solution with different concentrations. b) The release curves of $Cu^{2+}$ from the PAA-CHI-20 hydrogels after soaking into $CuSO_4$ solution with different concentrations. Data in a,b) were presented as means ± SD, n = 3.

10) The authors provided antibacterial data in Figure S12, while they did not provide the details of this test in experimental part. In the inhibition zone assay, the hydrogels displaying bacterial-killing ability should be due to the components released from the hydrogels. It is known $Cu^{2+}$ able to kill bacterial at low concentration, which also brings cytotoxicity at a relatively higher concentration. MgO was reported having the ability to kill bacteria due to it causing peralkaline, however, in the case of PAA-CHI-KGN-Mg, only $Mg^{2+}$ is released from the hydrogel, why did it achieve such obvious bacterial-killing ability?

**Reply 10:** Thanks for your kind suggestions. We sincerely apologize for the issue you pointed out regarding the experimental method of the antibacterial data in Supplementary Fig. 12, which is not elaborated in detail. This is indeed due to our oversight during the manuscript preparation process. Your rigorous attitude not only helps us promptly identify the shortcomings in our work, but also provides important guidance for improving the scientificity and completeness of the manuscript. In response to this oversight, we have supplemented the detailed method for the antibacterial performance test in the Methods section for your review.

Regarding the phenomenon that the PAA-CHI-KGN-$Mg^{2+}$ hydrogel releases only $Mg^{2+}$ but exhibits a significant antibacterial effect, we have provided the supplementary explanations and mechanism clarification based on the previous literatures and experimental observations. As a key effector ion, $Mg^{2+}$ can exert its effects through multiple mechanisms. Firstly, $Mg^{2+}$ can bind to the active enzymes within bacteria, interfering with the catalytic activity of these enzymes to disrupt vital biological processes such as DNA replication and protein synthesis. Meanwhile, it triggers oxidative stress responses, leading to the death of the bacteria. Secondly, $Mg^{2+}$ can also directly act on the bacterial membrane, by altering the permeability and stability of the membrane and disrupting the membrane potential balance, causing the leakage of internal substances and thereby inhibiting the growth and reproduction of bacteria. Thirdly, $Mg^{2+}$ may cause the pH value of the local culture medium to increase, thereby creating an alkaline microenvironment. This ability can damage the cell membrane structure and enzyme system function of pathogenic bacteria, and synergistically enhance the antibacterial effect together with the direct interaction of $Mg^{2+}$ (RSC Adv. 2024, 14, 10874; Front. Bioeng. Biotechnol. 2024, 12, 1417742; Chem Eng J. 2023, 475, 146092). The above is our supplementary explanation and mechanism clarification regarding the relevant issues. We sincerely request your review. If there are still any questions or if further clarification is needed, we are always ready to provide more detailed experimental data and literature support, and we look forward to your valuable feedback to improve the research.

Revision: "The susceptibility of *E. coli* and *S. aureus* to PAA-CHI, PAA-CHI-KGN-$Mg^{2+}$, and PAA-

CHI-$Cu^{2+}$ hydrogels were determined by methods similar to the Kirby-Bauer disk diffusion test (Oxoid, United Kingdom)[64]. *E. coli* and *S. aureus* were planted on the Mueller-Hinton agar plate (MH plate) using a cotton swab respectively, and different kinds of hydrogels were planted on the center of the MH plate. All the groups were incubated at 37 °C with 5% $CO_2$ for 48 h. After the culture, the results were calculated and photographed, the approximate diameter of the antibacterial cycle was measured, and the statistical analysis was conducted according to the measured results."

11) In Ref.12, it is reported that a proper Mg gradient can induce the dual lineage regeneration of osteochondral defect. On the one hand, can the PAA-CHI-KGN-Mg hydrogel release the Mg2+ at an optimized concentration for chondrogenesis? On the other hand, $Mg^{2+}$ also has the potential to promote angiogenesis as proved by many published works. Unlike $Cu^{2+}$, $Mg^{2+}$ shows significant cytotoxicity only at quite high concentrations. Why did not the authors consider still using Mg2+ in the bone zone?

**Reply 11:** We sincerely appreciate your valuable suggestions. Firstly, we have conducted a screening of $Mg^{2+}$ concentration and verified that a $Mg^{2+}$ concentration of 200 ppm can promote the chondrogenic differentiation of BMSCs to the greatest extent (Fig. 2f-i), which is consistent with the results presented in the Ref.32. Subsequently, by changing the concentration of the soaking solution, we confirm that the hydrogel soaked in 0.5 M $MgSO_4$ solution can release $Mg^{2+}$ at the optimal concentration (Supplementary Fig. 5). To further strengthen the rigor of this work, we have also supplemented relevant experiments to determine the optimal $Cu^{2+}$ concentration for promoting vasculogenesis. Our data indicate that $Cu^{2+}$ with concentration of 60 ppm can promote the vasculogenic effect of HUVECs to the greatest extent, and soaking the hydrogel into 0.1 M $CuSO_4$ solution can enable $Cu^{2+}$ to be released at the optimal concentration (Fig. 2j-n and Supplementary Fig. 5). More importantly, the sustained-release effect of $Cu^{2+}$ enables it to meet the vascularization requirements of the bone region and effectively promotes the ECO process. Under this circumstance, both $Mg^{2+}$ and $Cu^{2+}$ loaded hydrogels exhibit good biocompatibility. Live/dead staining and CCK-8 assays confirm that BMSCs and chondrocytes can proliferate well on these hydrogels without obvious cytotoxicity (Supplementary Fig. 8).

Regarding the situation where $Cu^{2+}$ is used instead of $Mg^{2+}$ in the subchondral bone zone, there are three factors as follows. Firstly, the hydrogel loaded with $Mg^{2+}$ appears white, while the one loaded with $Cu^{2+}$ shows a blue color (Supplementary Fig. 7). This color difference is helpful for precisely controlling the soaking depth and clearly displaying the ion distribution within the cartilage and subchondral bone layers. Secondly, compared with $Mg^{2+}$, $Cu^{2+}$ also has a stronger ability to promote vasculogenesis at low concentrations, which makes it more suitable for meeting the vascularization requirements of subchondral bone regions. Thirdly, the subchondral bone tissue area requires higher mechanical strength. The modification with $Cu^{2+}$ can enhance the mechanical properties of the hydrogel, enabling it to meet the requirements of the bone tissue (Supplementary Fig. 11). The stronger interaction between $Cu^{2+}$ and carboxyl groups in the hydrogel matrix increases the cross-linking density, thereby making its mechanical properties superior to those of the hydrogel

modified with $Mg^{2+}$. On the contrary, if the subchondral bone layered hydrogel is exposed to a low concentration of $Mg^{2+}$, its mechanical strength is bound to be weaker than that of the cartilage layer.

These results collectively confirm the rationality of our ion-selective strategy and provide strong evidence for the temporal control of sandwiched hydrogel scaffold on osteochondral regeneration. We will incorporate these detailed research results into the revised manuscript to further elucidate the mechanism and enhance the rigor of the study.

Revision:

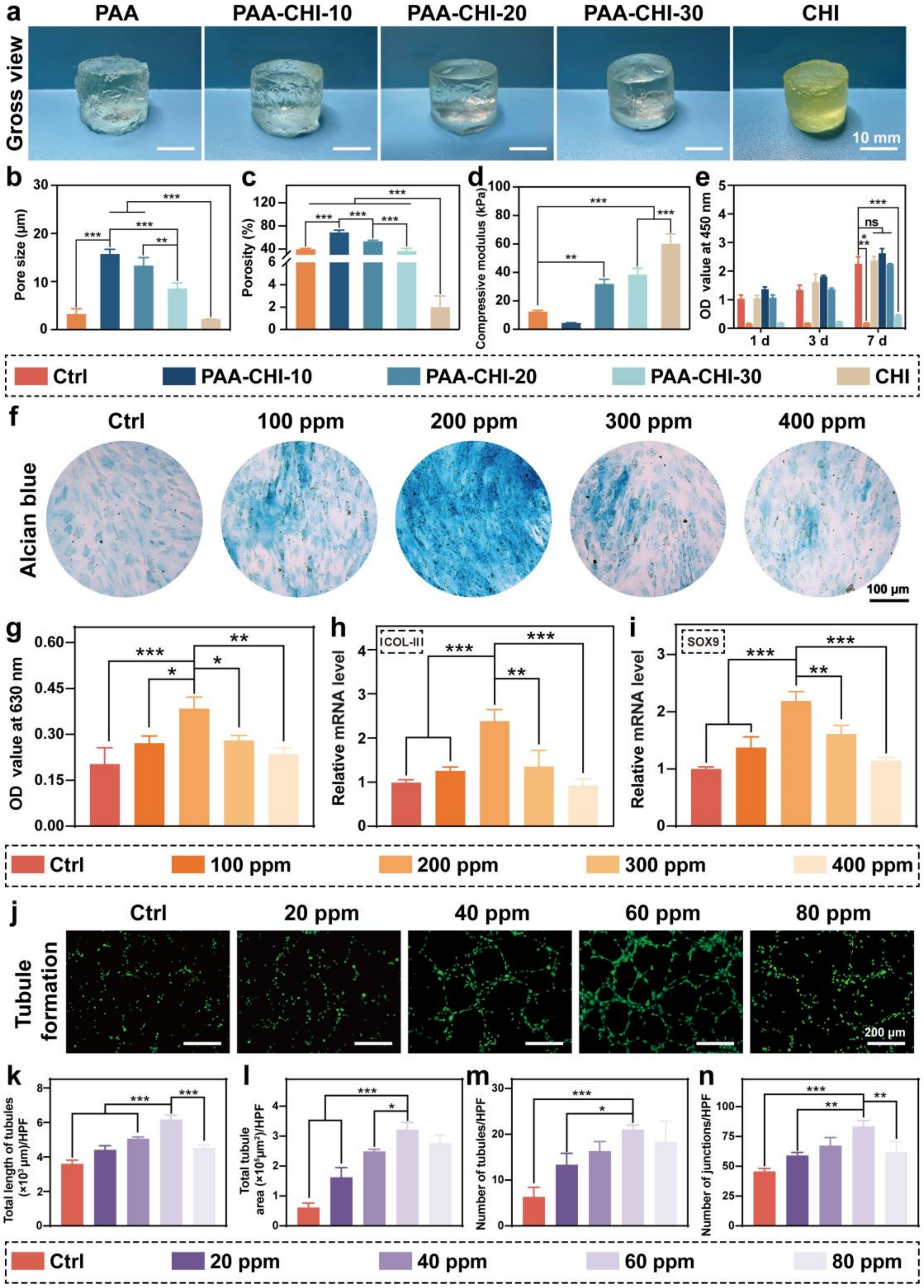

**Fig. 2 Optimization formulation of PAA-CHI hydrogels and ionic soaking concentration. a-d)** Gross view, pore size, porosity, and compressive behaviors of PAA, PAA-CHI-10, PAA-CHI-20, PAA-CHI-30, and CHI hydrogels. **e)** CCK-8 assay of BMSCs after co-culture with various hydrogels at specific times. **f)** Effect of $Mg^{2+}$ concentration on the formation of GAG by Alcian blue staining in BMSCs cultured for 2 weeks. **g)** Quantitative analysis of staining intensity in **f)**. **h, i)** Relative mRNA expression of chondrogenic differentiation-related indicators after co-culture of BMSCs with different concentrations of $Mg^{2+}$ for 2 weeks. **j)** Live cell staining of HUVECs after co-culture with different concentrations of $Cu^{2+}$ for 6 h. **k-n)** Statistical analysis of the total length of tubules, total tubules area, number of tubules and junctions in high-magnification field at 6 h. Data in **b-e, g-i, k-n)** were presented as means ± SD, n = 3. Statistical significance was determined using the one-way ANOVA with Tukey's post-hoc test. *p < 0.05, **p < 0.01, ***p < 0.001.

12) The PAA-CHI-Cu showed slightly higher compression modulus than the PAA-CHI-KGN-Mg, however, they both are quite low (20-30 kPa) at strain less than 30%. The ability to deform at 80-90% strain is not so meaningful for its implantation in the osteochondral defect, where the deformation is only about 10-30%. Can the authors further improve the compression strength of the hydrogels? On the other hand, it is confused to find that the compression stress of the FCCL-DG and WCCL-DG reached 0.16-0.24 MPa at about 60% strain (Fig.3e), while the individual PAA-CHI-KGN-Mg and PAA-CHI-Cu only show less than 0.1 MPa at this strain (Fig. S8). Please explain the reason why their combination able to obtain a significant increase in compression property.

**Reply 12:** Thanks for your valuable suggestions regarding the improvement of the compressive strength of the hydrogel. In fact, we have made some attempts to enhance the compressive strength of the hydrogel, such as increasing the solid content of PAA and ionic soaking concentration (Adv. Sci. 2023, 10, 2206242). However, high PAA concentration can cause a decrease in the environmental pH value while high ionic soaking concentration also brings about a certain biological toxicity, which can inhibit cell proliferation and damage biocompatibility (Supplementary Fig. 4). Taking into account the biocompatibility, mechanical performance, biological characteristics and feasible accessibility for the hydrogel scaffold and avoidance of the unnecessary excessive variables introduced by other additives (such as inorganic nanoparticles), here in this work, we have decided not to consider significantly increasing compressive modulus. Instead of overemphasizing complete modulus matching with natural tissues, we concentrated on the regulatory effects of the cartilage hydrogel and the subchondral bone hydrogel on cell fate, including the maintenance of hyaline cartilage phenotype in the cartilage layer and the promotion of vascularization in the subchondral bone layer, supported by detailed cellular experimental data and molecular mechanism analysis (Fig. 4d-f, Fig. 5 and Supplementary Fig. 26). This discovery highlights the necessity of balancing mechanical performance and biological functionality when designing scaffolds, which is also an important consideration in our design process. In addition, we would like to briefly mention that the compressive strength of the hydrogel is not significantly different from the previous literatures (Adv. Mater. 2024, 36, 2409400; Bioact. Mater. 2023, 19, 678). This indicates that our

current design meets the basic mechanical requirements for osteochondral repair, but we also fully aware that there are still many areas that need improvement to reach the mechanical performance level of natural tissues, as you have keenly pointed out.

In terms of the significant difference in compression performance, we have carefully examined the original data and found that the stress of the individual PAA-CHI-KGN-$Mg^{2+}$ and PAA-CHI-$Cu^{2+}$ hydrogels was approximately 0.1 MPa at a strain of 60%. The observed discrepancies can be attributed to three overlapping systematic errors that impacted measurement reliability. Firstly, during the preloading process, the control of initial applied force was not precise, thereby altering the baseline and causing the reported stress values under the target strain to include unexpected initial stress components. Secondly, the unevenness of samples clamping in different repeated experiments resulted in the non-uniformity of strain distribution, inconsistent contact between the hydrogel and the test fixture, resulting in higher strains being exerted in some areas compared to others, which led to the variability of stress readings and the reduction in the repeatability of the measurement results. Thirdly, the long-term operation fatigue of the force sensor led to a gradual drift in sensitivity, disrupting the linearity of the sensor's response during the experiment, and this drift caused systematic deviations in stress measurement, especially at higher strain levels, further causing data to be biased. We sincerely apologize for any confusion caused by these data. To address these issues, we will take targeted corrective measures. On the one hand, we will adopt a preloading scheme based on a dynamic mechanical analyzer to eliminate the baseline deviation of prestress, and deploy standard fixtures with micron-adjustable guides to ensure uniform clamping. On the other hand, we will calibrate the force sensors monthly using NIST-standardized weights to reduce sensitivity drift, and use standardized molds to ensure the consistency of the sample geometry.

We sincerely appreciate your valuable suggestions and insightful observations. We have identified the root cause of the data deviation and will immediately implement calibration and standardization measures to improve the situation. We are committed to using more accurate data to further enhance and develop the osteochondral repair research.

13) To form the Mg and Cu gradient in the hydrogel by soaking, please add some words in the experimental part to clarify the depth of the hydrogel soaking in the magnesium sulfate or copper sulfate solution.

**Reply 13:** Thanks for your kind suggestions. We have carefully revised the experimental section to clearly describe the soaking depth of the hydrogel in the $MgSO_4$ and $CuSO_4$ solutions. Specifically, the cartilage layer containing KGN (accounting for 45% of the entire height) is completely immersed in the 0.5 M $MgSO_4$ solution, while the subchondral bone layer (accounting for 45% of the entire height) is completely immersed in the 0.1 M $CuSO_4$ solution, ensuring that the entire thickness of each layer is thoroughly soaked.

Revision: "Subsequently, the KGN-containing cartilage layer was fully soaked in a 0.5 M $MgSO_4$ solution for 1 h. Its height accounted for 45% of the total hydrogel height and the soaking depth was strictly controlled to cover the entire thickness of the cartilage layer. Following this, the subchondral

bone layer was fully soaked in a 0.1 M $CuSO_4$ solution for 1 h. Its height also accounted for 45% of the total hydrogel height and the soaking depth was ensured to cover the entire thickness of the subchondral bone layer. A sandwiched hydrogel was finally obtained with the height ratio of 45% for the cartilage layer, 10% for the intermediate layer and 45% for the subchondral bone layer. In addition, we also prepared NCCL-CG and FCCL-DG as controls. Due to the variety of hydrogel scaffolds, we have summarized their naming rules and characteristics in Supplementary Table 1."

Comments on in vitro cell culture

14) The authors collected BMSCs and chondrocytes for cytotoxicity assay, while in chondrogenic differentiation, they culture BMSCs with chondrogenic inductive medium for 7 days, and added hypertrophy induction medium and KGN to confirm the KGN effect on inhibiting hypertrophy. Though this test has no problem, the question is, why did not the authors directly apply chondrocytes in this test, which may reflect the situation more close to in-situ cases? On the other hand, in the chondrogenic differentiation assay, as shown in Fig. 4a-4c, why was the PAA-CHI-KGN-Mg group not involved?

**Reply 14:** Thanks for your kind suggestions. Your professional doubts have provided us with an important direction for optimizing the research explanation. Firstly, regarding the rationality of choosing BMSCs over directly using chondrocytes in the *in vitro* experiments, the design of the *in vivo* scaffolds in this study follows the "cell-free loading" strategy. The chondrogenesis process relies on the recruitment and differentiation of endogenous stem cells from the host. Based on our research foundation, the scaffolds have certain functions in promoting the migration and recruitment of BMSCs (Supplementary Fig. 16, 17). These stem cells, in the hypoxic microenvironment induced by the scaffolds, differentiate directionally into chondrocytes through the HIF-1α signaling pathway (Fig. 4a-c). However, long-term hypoxic stimulation may induce chondrocytes hypertrophy by activating the RUNX2/COL-X signaling axis, which is a key pathological process leading to ectopic ossification of tissue-engineered cartilage. Therefore, in the *in vitro* experiments, we adopted a staged model of "BMSCs chondrogenesis induction → hypertrophy induction" to simulate the complete cell fate regulation process *in vivo* of "stem cell recruitment - chondrogenesis - hypertrophy transformation". The phenotypic stability of mature chondrocytes differs from that of newly generated chondrocytes *in vivo*. Therefore, this design is more in line with the cell source and differentiation sequence of the scaffolds *in vivo*, and is expected to reflect the intervention effect of KGN more realistically on the hypertrophy of newly generated chondrocytes.

Regarding the reason why the PAA-CHI-KGN-$Mg^{2+}$ group was not included in the cartilage differentiation experiment, thank you for pointing out this detail. The role of KGN in promoting the chondrogenic differentiation of BMSCs has been widely confirmed in the past decade (Bioact. Mater. 2025, 49, 255; Adv. Mater. 2024, 36, 2401094). The innovation focus of this study lies in exploring the inhibitory mechanism of KGN on chondrocytes hypertrophy, rather than repeating the verification of its chondrogenic differentiation efficacy. Therefore, in the chondrogenic differentiation experiment, the PAA-CHI-KGN-$Mg^{2+}$ group was not set up, but the targeted

regulatory effect of KGN was directly verified through the hypertrophy model (Fig. 4d-f and Supplementary Fig. 26). This design conforms to the research logic of “avoiding redundant verification and focusing on core innovation”, and reserves more sufficient experimental space for mechanism exploration.

15) For the PAA-CHI-Cu hydrogel, the authors focused on its vasculogenesis potential, without conducting chondrogenic, hypertrophic and osteogenic differentiation. The authors continuously mentioned ECO in their study, providing only an immunohistological staining on Col-X for regenerated tissues collected from rabbit to show the occurrence of the ECO process, while this is not sufficient. For in vitro evaluations, the authors are suggested to prove the chondrogenic - hypertrophic - osteogenic transition using the PAA-CHI-Cu hydrogel.

**Reply 15:** Thanks for your kind suggestions. Your suggestion regarding the supplementation of PAA-CHI-$Cu^{2+}$ hydrogel-mediated ECO for *in vitro* verification has prompted us to systematically explore the complexity of the ECO process and the technical limitations of *in vitro* simulation. ECO, as the core pathological physiological process of bone regeneration, involves a cascade of reactions including proliferation and differentiation of chondrocytes, hypertrophy, vascular invasion, and mineralization of bone matrix. Among these, the dynamic interaction of blood, cartilage and bone is the key factor driving bone formation. In the *in vitro* culture system, although certain factors can induce chondrocytes to transform into hypertrophic phenotype, characterized by increased COL-X expression and enlarged cell volume, the transformation of hypertrophic chondrocytes into osteoblasts phenotype is highly dependent on the multiple microenvironmental signals brought about by vascular invasion. On the one hand, BMP2/4 secreted by vascular endothelial cells can activate the RUNX2 pathway, promoting the expression of ALP and mineralization-related genes in hypertrophic chondrocytes. On the other hand, the nutritional gradient and oxygen pressure changes formed by vascular invasion, that is, from a hypoxic environment to a normoxic state, can regulate the metabolic reprogramming of cells through the HIF-1α/VEGF axis. This process cannot be replicated in static culture dishes or Transwell systems. The extracellular environment lacks the 3D spatial structure necessary for vascular growth, and cannot simulate the vascular niche *in vivo* composed of vascular endothelial cells, pericytes, and MSCs. This results in hypertrophic chondrocytes being unable to receive paracrine signals and thus remaining in an intermediate state of differentiation, making it difficult for them to complete the final transformation into osteoblasts.

Inspired by your suggestion, we have added IF staining for COL-X to support the ECO process (Supplementary Fig. 34). Moreover, we intend to adopt *in vivo* models such as subcutaneous implantation in our subsequent research to verify the regulatory effect of PAA-CHI-$Cu^{2+}$ hydrogel on the ECO process. The core design concept is to reveal how the hydrogel promotes bone regeneration by regulating the “vascular-cartilage-bone” cascade reaction through dynamic monitoring of the vascularization process, the phenotypic transformation from cartilage hypertrophy to osteogenesis, and the spatiotemporal expression of key markers. The experiment will focus on the dynamic interaction between vascular invasion and cartilage hypertrophy, and verify the

hydrogel-mediated ECO process by detecting the expression changes of vasculogenic markers, cartilage hypertrophy markers, and osteogenic markers. Technically, we intend to combine *in vivo* imaging to observe the cell interaction trajectories and analyze the cell factor network to clarify the regulatory mechanism of the hydrogel on microenvironment signals. This research will provide key experimental evidence for the clinical translation of the hydrogel scaffolds in osteochondral regeneration, and once again, thanks for your professional suggestions that have pointed out the important direction for the deepening of our research.

Revision:

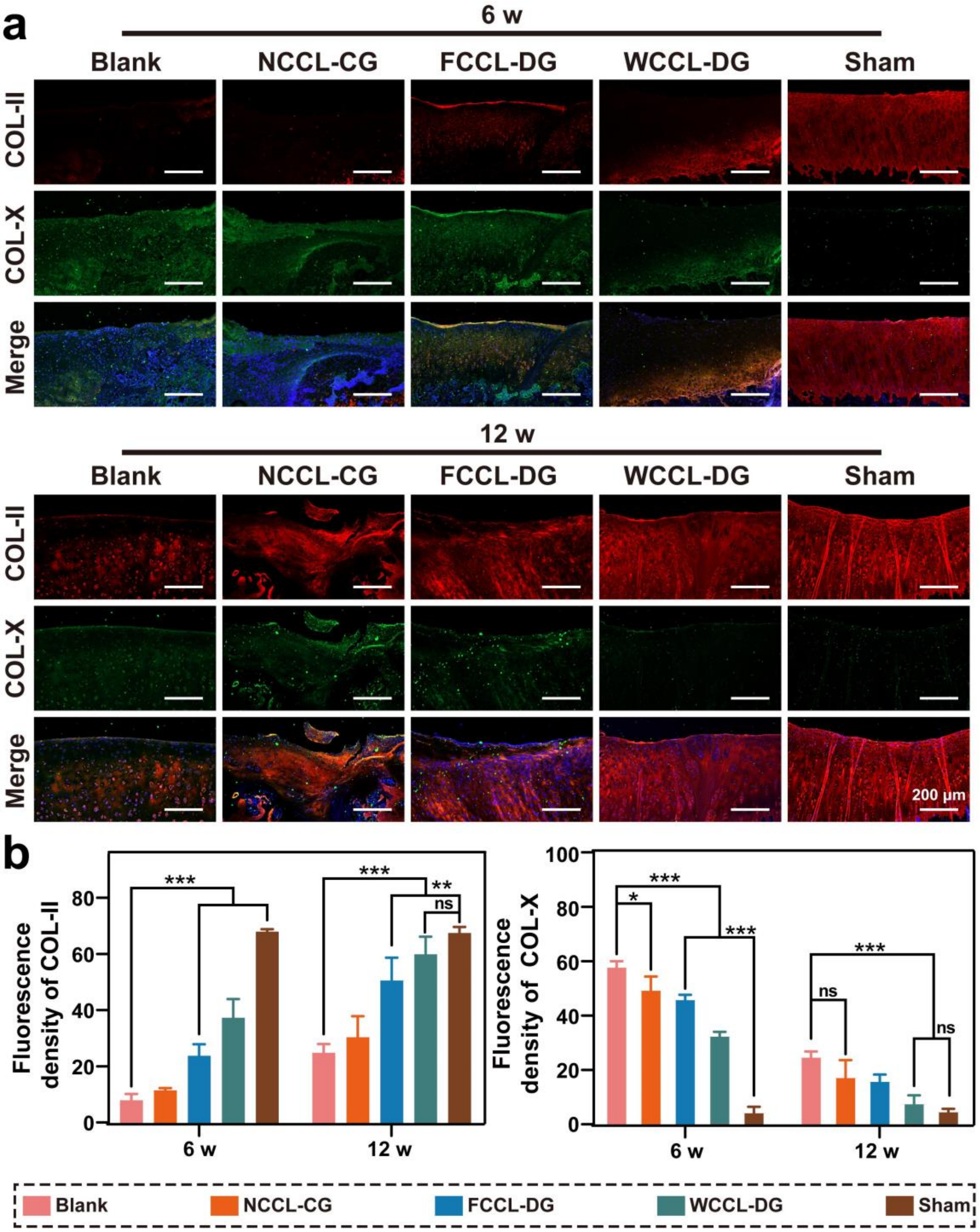


**Supplementary Fig. 34 Expression levels of COL-II and COL-X at specific time points for**

**each group. a)** IF staining of COL-II and COL-X. **b)** Quantitative analysis of the fluorescence intensity in **a)**. Data were presented as means ± SD, n = 4. Statistical significance was determined using the one-way ANOVA with Tukey's post-hoc test. *p < 0.05, **p < 0.01, ***p < 0.001.

Comments on in vivo evaluations

16) The authors applied the rabbit femoral trochlea to perform in vivo evaluations, as well known, this is a defect model without significant loading. The authors found that the WCCL-DG is more sufficient in tissue regeneration than the FCCL-DG, as the only difference between them being the flat interface and wavy interface. The authors are required to clarify the difference in stress distribution on the implanted WCCL-DG and FCCL-DG, particularly at the interface, which is necessary to understand the in vivo outcomes.

**Reply 16:** Thanks for your kind suggestions. Regarding the question of "the rabbit femoral trochlea being a model without significant load", we have provided an explanation based on anatomical features and relevant research progress. The femoral trochlea, as a key component of the patellofemoral joint, has been extensively studied in clinical research and its stress characteristics have been well established (Ann. Rheum. Dis. 1979, 38, 307; Ann. Rheum. Dis. 2014, 73, 1222). During the flexion and extension of the joint, the dynamic contact between the patella and the trochlear groove generates significant compressive stress. When the knee joint is from the extended position to 90° of flexion, the contact pressure of the patellofemoral joint can increase from 0 MPa to 7-10 MPa, and under extreme postures such as deep squats, the pressure peak can reach above 20 MPa. This physiological load is not only a necessary condition for maintaining the balance of cartilage metabolism but also an important trigger for pathological changes. Clinical data show that approximately 85% of patients with patellofemoral joint diseases have trochlear dysplasia or imbalance in stress distribution. This further confirms that the mechanical environment of this area has a direct impact on joint function. Furthermore, a recent study also provides direct evidence for the validity of this model (Adv. Mater. 2024, 36, 2409400). This study used a femoral trochlea osteochondral defect model in Bama pigs. By implanting a piezoelectric conductive hydrogel scaffold, mechanical compression forces during joint movement were utilized to induce a potential difference in the scaffold, ultimately achieving the directed differentiation of BMSCs into chondrocytes and osteoblasts. The core mechanism lies in the fact that the dynamic loads generated by joint movement can directly regulate cell behavior. This discovery is highly consistent with the research strategy of choosing the femoral trochlea model. Additionally, as a commonly used experimental animal, the anatomical structure and kinetic characteristics of the patellofemoral joint of rabbits have been comprehensively studied. During natural activities such as jogging and jumping, the periodic loads in the cartilaginous area are sufficient to simulate the physiological mechanical environment *in vivo*, providing reliable biomechanical stimulation conditions for evaluating the osteochondral regeneration performance. It should be emphasized that the femoral trochlea model has been widely applied in osteochondral repair research. Its advantage is that it can simultaneously evaluate the repair effects of the hydrogel scaffolds on cartilage, subchondral bone, and interface

integration, and has a high similarity to the pathological physiological process of human patellofemoral joint diseases. The obtained results will be closer to clinical application scenarios.

In response to the requirement of "clarify the difference in stress distribution on the implanted WCCL-DG and FCCL-DG", we have provided the following explanation by combining finite element analysis (FEA) and transcriptomics data. The wave-shaped interface of WCCL-DG and the planar interface of FCCL-DG exhibit fundamental differences in terms of stress conduction and cell mechanical perception. The FEA results indicate that under the simulated physiological load conditions, the planar interface of FCCL-DG can cause stress concentration on the surface of the cartilage layer, which may lead to the degradation of the cartilage matrix (Fig. 6f-i). In contrast, the wave-shaped interface of WCCL-DG effectively reduces the peak stress of the cartilage layer through gradient buffering, and through the mechanical interlocking effect of the concave-convex structure, it evenly distributes the stress to the subchondral bone (Supplementary Fig. 31). More importantly, the reactive force generated by the wave-shaped structure under pressure causes the cartilage layer to be subjected to multi-directional stress, enhancing the vitality of chondrocytes and promoting their secretion of specific matrix (Fig. 6j-m).

To better mimic the dynamic compressive environment within the joints, we further supplemented the cyclic compression tests for both WCCL-DG and FCCL-DG hydrogels at strain levels of 10%, 20%, and 30% to assess their fatigue resistance (Fig. 6a-c). The results demonstrated that WCCL-DG maintained excellent mechanical stability across all strain conditions. Its peak stress remained consistently between 90% and 100% of the initial value throughout the cyclic loading process. In contrast, FCCL-DG exhibited a continuous decline in peak stress with increasing cycles, indicating that the wavy interface design of WCCL-DG significantly improved its fatigue resistance compared to the flat interface of FCCL-DG. Furthermore, we also evaluated the interface stability of both hydrogels through stretch and shear tests (Fig. 6e). Ashby's analysis indicated that the fracture resistance of WCCL-DG was significantly superior to that of FCCL-DG. This enhancement could be attributed to its wavy interface, which increased the contact area between the upper and lower layers, thereby effectively enhancing the fracture strength of WCCL-DG. Additionally, the mechanical interlocking effect caused by the wavy interface also significantly improved the friction resistance of WCCL-DG. This effect enabled the scaffold to maintain structural stability even under higher shear forces, which was crucial for withstanding the complex biomechanical stresses in natural joints.

In addition, transcriptomics sequencing further revealed the regulatory mechanism of the stress microenvironment changes mediated by structural differences on cell functions (Fig. 9). Compared with the FCCL-DG group, the chondrocytes in the WCCL-DG group showed significant differential expression of stress-sensitive gene pathways under multi-directional stress stimulation. The expression levels of extracellular matrix synthesis-related genes were significantly upregulated, the secretion of glycosaminoglycan increased significantly, and the fiber arrangement presented an anisotropic distribution, which was closer to the natural cartilage matrix structure. In addition, the

expression levels of stress injury-related pathways and apoptosis genes were significantly reduced, indicating that the wavy structure alleviated stress concentration and reduced cartilage degradation. These results confirmed that the stress distribution differences mediated by interface structure were the key factors regulating cell behavior. The wavy design of WCCL-DG achieved a dual effect of reducing damage and promoting synthesis by optimizing the mechanical microenvironment, thereby significantly improving the effect of osteochondral regeneration.

These supplementary mechanical evaluations confirm that the wavy interface design of WCCL-DG not only enhances fatigue resistance but also improves interfacial stability and friction properties, making it a more robust scaffold for osteochondral regeneration in dynamic joint environments. These detailed results have been added in the revised manuscript to provide a comprehensive assessment of the scaffold's mechanical performance.

17) For the Col-X staining shown in Figure 8a, they are actually hard to be distinguished, which are insufficient to support the ECO process. Immunofluorescent staining on Col-X is suggested. The staining on Col-I is also required. The interface of the regenerated tissue should be enlarged to mark out the tidemark zone for clear comparison.

**Reply 17:** Thanks for your kind suggestions. Regarding the issue of unclear results from the Col-X staining, we have re-performed the IF staining for COL-X and COL-II for all groups, and conducted a systematic analysis of the repaired tissues from each group at 6 weeks and 12 weeks (Supplementary Fig. 34). The results showed a distinct functional gradient for the expression of COL-II. Compared with the blank group, with the introduction of hydrogel implantation, metal ion regulation, and wavy CCL, the expression level of COL-II gradually increased. Among them, the COL-II expression level in the WCCL-DG group was close to the normal osteochondral tissue level at 12 weeks. Furthermore, it was worth noting that the changes in COL-X expression reflected the fundamental differences in the repair process. At 6 weeks, except for the sham group, the expression levels of COL-X were higher in all experimental groups. However, the high expression in the blank group was closely related to the degeneration of damaged area, and we speculated that this might be an abnormal expression induced by aging, consistent with the poorer repair effect. In contrast, the hydrogel implantation groups showed a normal physiological manifestation of chondrocytes hypertrophy during the process of ECO. At 12 weeks, the expression level of COL-X in the hydrogel implantation groups gradually decreased as the repair result improved, indicating that ions and the wavy CCL accelerated the ECO process, which effectively support the rationality of ECO process.

Furthermore, we supplemented the COL-I IF staining according to your constructive advices (Supplementary Fig. 35). Using the IF co-localization technique, we marked the tidemark zone in the regenerated tissue. The results showed that the tidemark in the WCCL-DG group were uniform and continuous, with clear and complete structures, which was highly consistent with the tidemark of normal osteochondral tissue. However, the tidemark in the other groups were all fractured or misaligned, and the interface integration was poor. This result directly revealed the crucial role of the wavy CCL in promoting the long-term functional integration of osteochondral.

Revision: “IF staining results also confirmed that the expression of COL-II exhibited a distinct functional gradient characteristic. Compared with the blank group, with the introduction of hydrogel implantation, metal ion regulation, and wavy CCL, the expression level of COL-II gradually increased. Among them, the expression level of COL-II in the WCCL-DG group was close to the normal osteochondral tissue level at 12 weeks (Fig. 8a,b and Supplementary Fig. 34). Furthermore, it was worth noting that the changes in COL-X expression reflected the fundamental differences in the repair process. At 6 weeks, except for the sham group, the expression levels of COL-X were higher in all experimental groups, providing strong evidence to verify the transition of hypertrophic chondrocytes into mineralized bone tissue and the activity of ECO process for the subchondral bone. However, the high expression in the blank group was closely related to the degeneration of the damaged area, and we speculated that this might be an abnormal expression induced by aging, consistent with the poorer repair effect. In contrast, the hydrogel implantation group showed a normal physiological manifestation of chondrocytes hypertrophy during the process of ECO. At 12 weeks, the expression level of COL-X in the hydrogel implantation group gradually decreased as the repair effect improved, indicating that the ions and wavy CCL accelerated the process of ECO. Compared with the blank group, the new bone formation in the hydrogel groups was more extensive without observation of hypertrophic chondrocytes, further demonstrating the completion of the ECO process (Fig. 8a and Supplementary Fig. 34).

Furthermore, using the IF co-localization technique, we also marked the tidemark zone in the regenerated tissue (Supplementary Fig. 35). The results showed that the tidemark in the WCCL-DG group were uniform and continuous, with clear and complete structures, which were highly consistent with the tidemark of normal osteochondral tissue. However, the tidemark in the other groups were all fractured or misaligned, and the interface integration was poor, which directly revealed the crucial role of the wavy CCL in promoting the long-term functional integration of osteochondral tissues.”

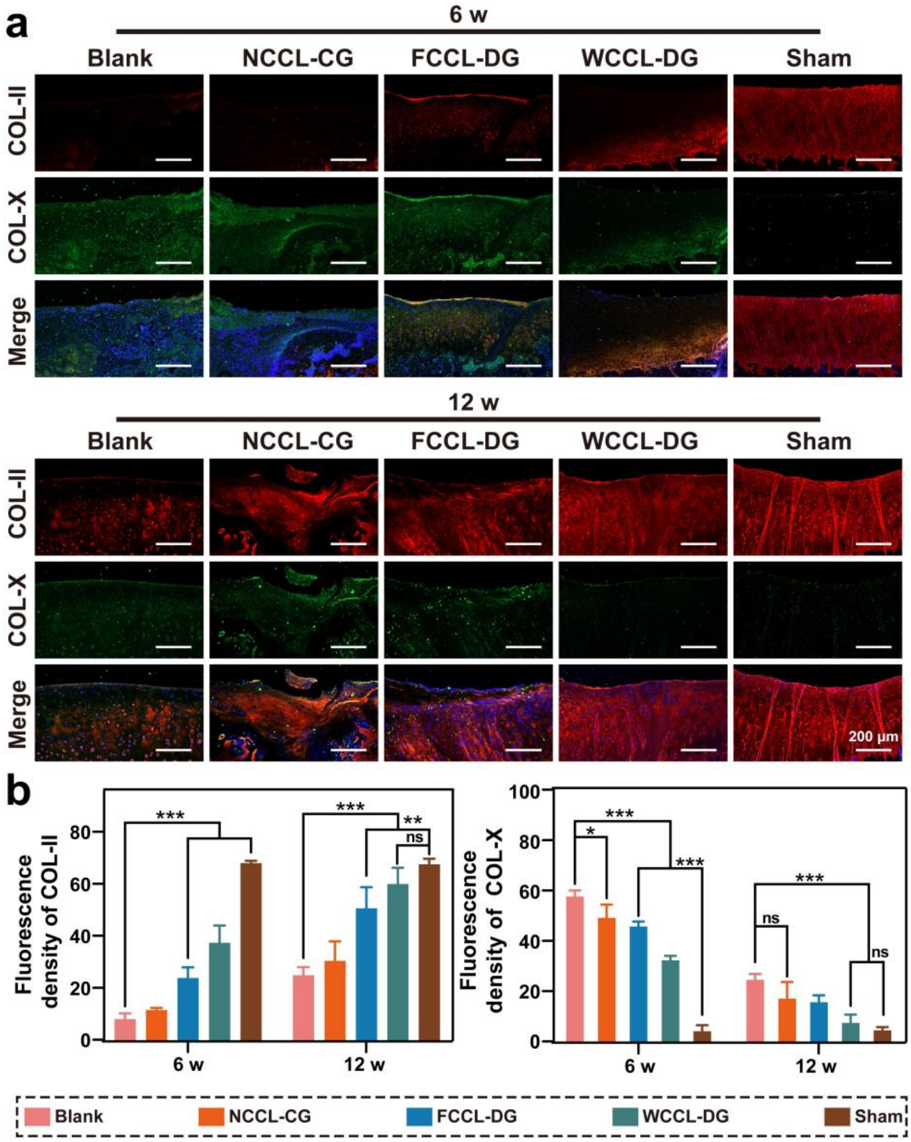


**Supplementary Fig. 34 Expression levels of COL-II and COL-X at specific time points for each group. a)** IF staining of COL-II and COL-X. **b)** Quantitative analysis of the fluorescence intensity in **a)**. Data were presented as means ± SD, n = 4. Statistical significance was determined using the one-way ANOVA with Tukey's post-hoc test. *$p < 0.05$, **$p < 0.01$, ***$p < 0.001$.

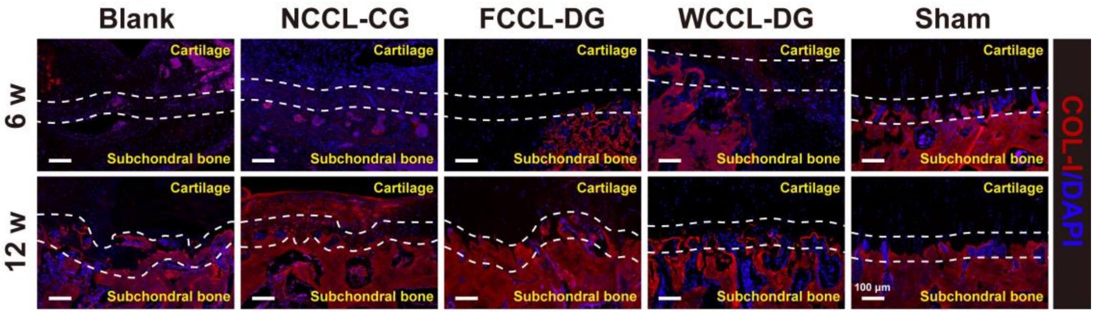


**Supplementary Fig. 35** Comparison of the regeneration status of the tidemark at specific time points for each group through IF staining of COL-I.

Comments on mechanism

18) It is a good point that the authors collected the regenerated tissue at 4 weeks post-surgery for

RNA sequencing analysis. However, due to the fact that the difference between the in-situ loading status on the WCCL-DG and FCCL-DG is unclear, the conclusion on the biomechanical mechanisms of wavy interface on promoting cartilage regeneration is not so convincing.

**Reply 18:** Thanks for your professional and constructive suggestions. The femoral trochlea is a core weight-bearing component of the patellofemoral joint and not a pressure-free area. It bears significant dynamic loads during joint movement: during knee flexion from full extension to 90 degrees, contact pressure increases from near zero to 7-10 MPa, and deep squatting can push peaks above 20 MPa. This physiological load maintains cartilage metabolism and activates intracellular signals, while dysplasia leads to uneven stress distribution and chondromalacia. In addition, a recent study using Bama pig models also validated that dynamic pressure induced piezoelectric effects in hydrogels to promote stem cell differentiation (Adv. Mater. 2024, 36, 2409400), strongly supporting the rationality of our model selection. The relevant discussion has been elaborated in detail in question 16.

We deeply regret that we cannot supplement *in vivo* mechanical testing at this stage due to two insurmountable constraints. Original animal models have completed all preset endpoints and were euthanized per ethical protocols; reconstructing the model would require rearing rabbits, performing surgeries, and monitoring for at least three months, which far exceeds the current review cycle. Additionally, modifying the experiment plan to implant sensors involves additional ethical approval, including biocompatibility reports, surgical safety protocols, and post-operative pain management plans, whose review typically takes 2-3 weeks and is infeasible within the tight timeline. In future studies, we will implement targeted experiments to address the identified gaps. We will use fiber-optic micro-pressure sensors to monitor dynamic pressure distribution at the scaffold-host tissue interface at multiple post-operative time points to clarify how the wavy interface modulates mechanical load transfer compared to the flat interface. Combining existing RNA-seq data, we can analyze mechanical signal pathways such as YAP/TAZ and MAPK, validate key genes, and localize nuclear translocation to reveal molecular mechanisms underlying cartilage regeneration regulation.

The current FEA results indicate that the wave-shaped CCL has significant stress dispersion and cartilage protection effects (Fig. 6f-i). The core mechanism lies in the wave-like structure, which effectively reduces the stress peak on the cartilage surface, thereby reducing the risk of cartilage cell apoptosis and extracellular matrix degradation. This theoretical discovery provides an important foundation for subsequent research but still needs to be verified by *in vivo* experiments for its actual effect. In future studies, we will conduct large-animal experiments to evaluate the function of the wave-like structure. The model selection will prioritize species such as goats or pigs that have similar joint anatomical structure, load-bearing characteristics, and cartilage metabolism patterns to humans. The experimental design may cover multi-dimensional assessment indicators under dynamic load conditions, including real-time monitoring of contact pressure distribution during the postoperative period using joint pressure sensors, combined with histological staining to observe cartilage cell morphology and matrix secretion, and using high quality precision equipment to

analyze the structural integrity and morphological changes of the CCL. The experimental period will cover 6 to 12 months after surgery to observe the stability and repair effect under long-term load conditions. Meanwhile, we will focus on exploring the influence of the shape and thickness of the CCL on the relationship between structure and stress distribution. By constructing CCL models with different wave amplitudes, wavelengths, and thicknesses, we can systematically analyze their regulatory effects on stress dispersion efficiency and energy absorption capacity, and identify the more optimal structural parameter combinations to achieve uniform stress distribution and effective energy buffering. These results can provide key support for clinical translation. On the one hand, the optimized parameters obtained from large-animal experiments can guide the design of wave-shaped CCL scaffolds, making them more in line with the physiological load requirements of the human body. On the other hand, by revealing the structure-activity relationship of the CCL, we can develop more biocompatible and mechanically adaptable scaffolds, improving their stability and repair effect *in vivo*. Moreover, these findings can also be used to formulate standardized clinical application guidelines, helping doctors select the optimal scaffold design parameters based on the specific injury condition of patients, further improving the individualization and effectiveness of treatment, and ultimately promoting the transformation of this technology from laboratory research to clinical application, thus providing more precise and effective treatment solutions for osteochondral repair.

We are deeply grateful for your meticulous attention to details and valuable insights during this review process. Although we are aware of the limitations of the current work, we remain committed to further improving our research in the future to fill the remaining gaps and provide more powerful and influential research results in the field of osteochondral regeneration.

19) Other comments

Please check the "Materials" section, it is claimed where the mice and SD rats were bought, but the SD rats should be New Zealand white rabbits.

Please check Fig. 6a, the texts and the images do not match each other.

In Fig.S17a, please replace the "Ctrl" with "CHI" for clarity. Scale bars are missing.

Throughout the text, the meaning of the "FCCL-CG" was not explained.

In Fig.S25, there are two rows of images at the same time point for each group. Please clarify the differences between them in the legend.

In Figure 8a, what are those red arrows pointing to?

In paper writing, it is suggested to put the FEA data together with the rheological outcomes, that they can support each other more clearly without putting the differentiation sections between the two parts.

**Reply 19:** We sincerely appreciate your meticulous and valuable comments. We deeply apologize for the oversights and inaccuracies in the initial submission, which may cause inconvenience during your review process. Below, we have provided a detailed response to each comment and outline the corresponding revisions.

1. Animal model correction: In the "Materials" section, we have changed the "SD rats" to "New Zealand white rabbits", and verified the accuracy of the source information for their procurement to ensure complete compliance with the requirements of the research method.

Revision: "C57BL6 mice, female New Zealand white rabbits, and Sprague-Dawley (SD) rats were purchased from SiPeiFu Biotechnology Co., Ltd. (SPF; Beijing, China)."

2. We have updated the figures to ensure precise matching between the images and their descriptive texts, eliminating any discrepancies between visual content and its explanation.

Revision:

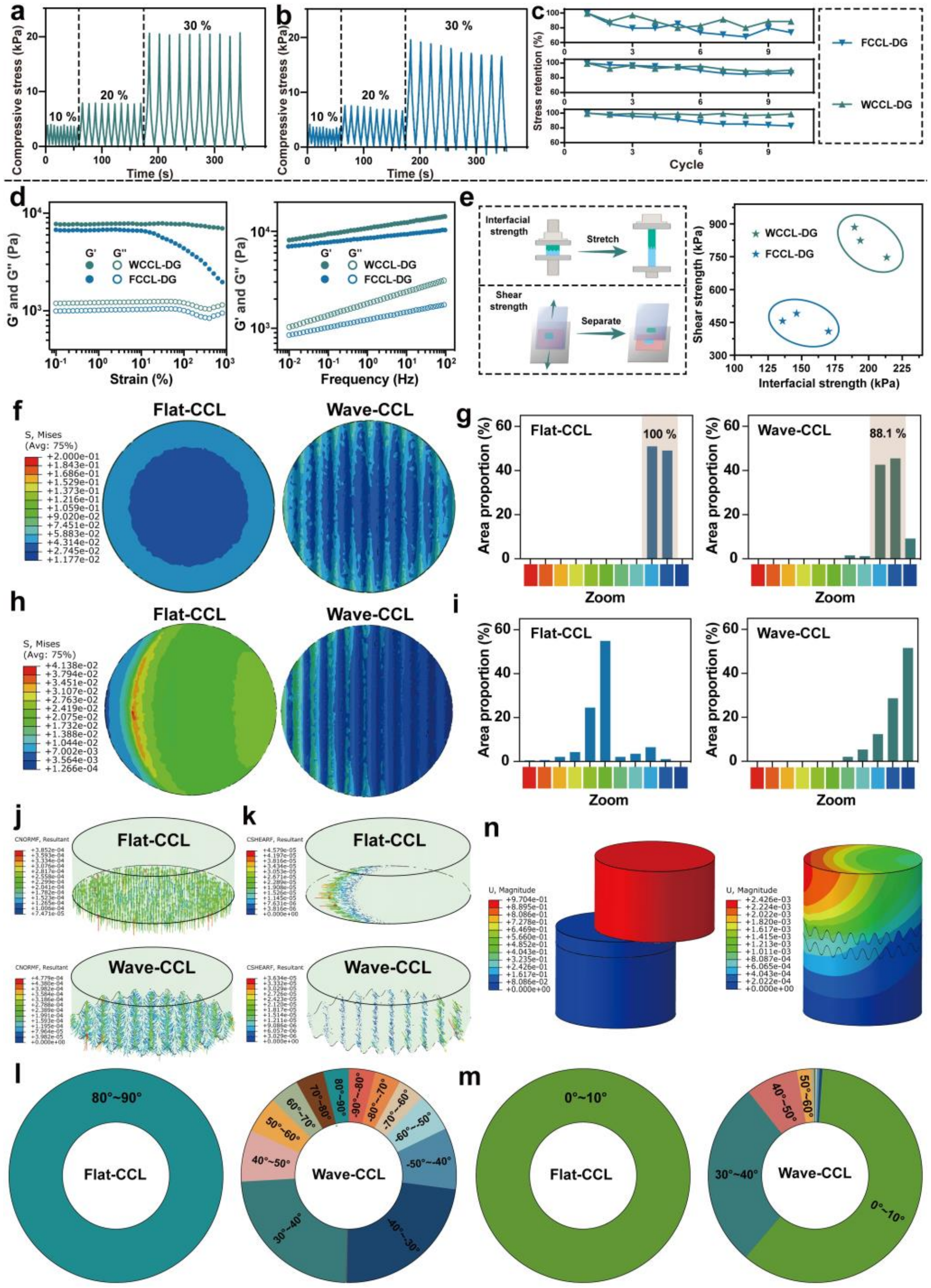


**Fig. 6 The mechanical differences between WCCL-DG and FCCL-DG hydrogels. a)** Cyclic compression curves of WCCL-DG hydrogel under different strains. **b)** Cyclic compression curves

of FCCL-DG hydrogel under different strains. **c)** Stress retention conditions of WCCL-DG and FCCL-DG hydrogels during the cyclic compression process. **d)** Rheological properties of FCCL-DG and WCCL-DG hydrogels. **e)** Stretch and shear properties of WCCL-DG and FCCL-DG hydrogels. **f)** Thermograms of stress distribution on the lower surface of the cartilage layer when two models were subjected to a compressive stress of 0.1 MPs respectively. **g)** Statistical analysis of the proportion of area occupied by different color blocks in **f)**. **h)** Thermograms of stress distribution on the lower surface of the cartilage layer when two models were subjected to a shear stress of 0.1 MPs respectively. **i)** Statistical analysis of the proportion of area occupied by different color blocks in **h)**. **j)** Direction of stress in the lower surface of the cartilage layer when subjected to compressive stress in two models. **k)** Direction of stress in the lower surface of the cartilage layer when subjected to shear stress in two models. **l)** Statistical analysis of stress of different ranges in **j)**. **m)** Statistical analysis of stress of different ranges in **k)**. **n)** Thermal map of overall displacement under shear stress for two models.

3. We have replaced the label "Ctrl" with "CHI" in Supplementary Fig. 21 for clarity and added the missing scale bars to all figures, providing essential spatial context for the results.

Revision:

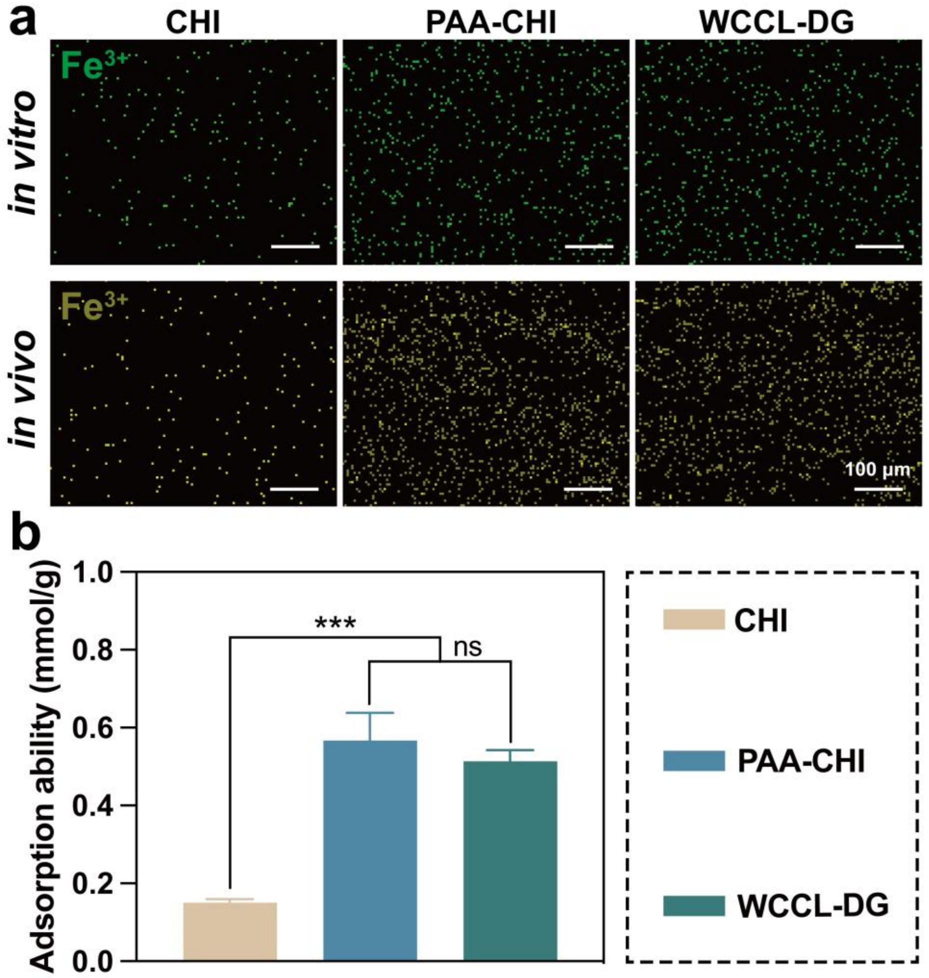


**Supplementary Fig. 21 Verification of iron ion chelating ability of CHI, PAA-CHI and WCCL-**

**DG groups. a)** EDS scanning of hydrogels after *in vitro* soaking in a ferric ion solution or *in vivo* dorsal burial. **b)** Quantitative analysis of iron in hydrogels by ICP. Data in **b)** were presented as means ± SD, n = 3. Statistical significance was determined using the one-way ANOVA with Tukey's post-hoc test. *$p < 0.05$, **$p < 0.01$, ***$p < 0.001$.

4. To resolve the ambiguity of "FCCL-CG", we have added Supplementary Table 1 in the revised manuscript, which systematically describes the naming conventions and key characteristics of all hydrogel formulation for reader comprehension.

Revision:

**Supplementary Table 1.** Naming conventions for hydrogel groups.

| **Abbreviation** | **Full name** | **Does it include the CCL layer and its form** | **Characteristics** |
|---|---|---|---|
| NCCL-CG | None Calcified Cartilage Layer-Composite Gel | No | Double-layer hydrogel, not subjected to ion soaking |
| FCCL-DG | Flat Calcified Cartilage Layer-Double-network Gel | Yes，flat | Double-layer hydrogel subjected to bidirectional ion soaking |
| WCCL-DG | Wave Calcified Cartilage Layer-Double-network Gel | Yes，wave | Double-layer hydrogel prepared by a wave-shaped template subjected to bidirectional ion soaking |

5. Due to the initial errors in image arrangement and layout, we have re-stained the tissue sections and reorganized Supplementary Fig. 34 to clearly present IF images of different groups at two distinct time points.

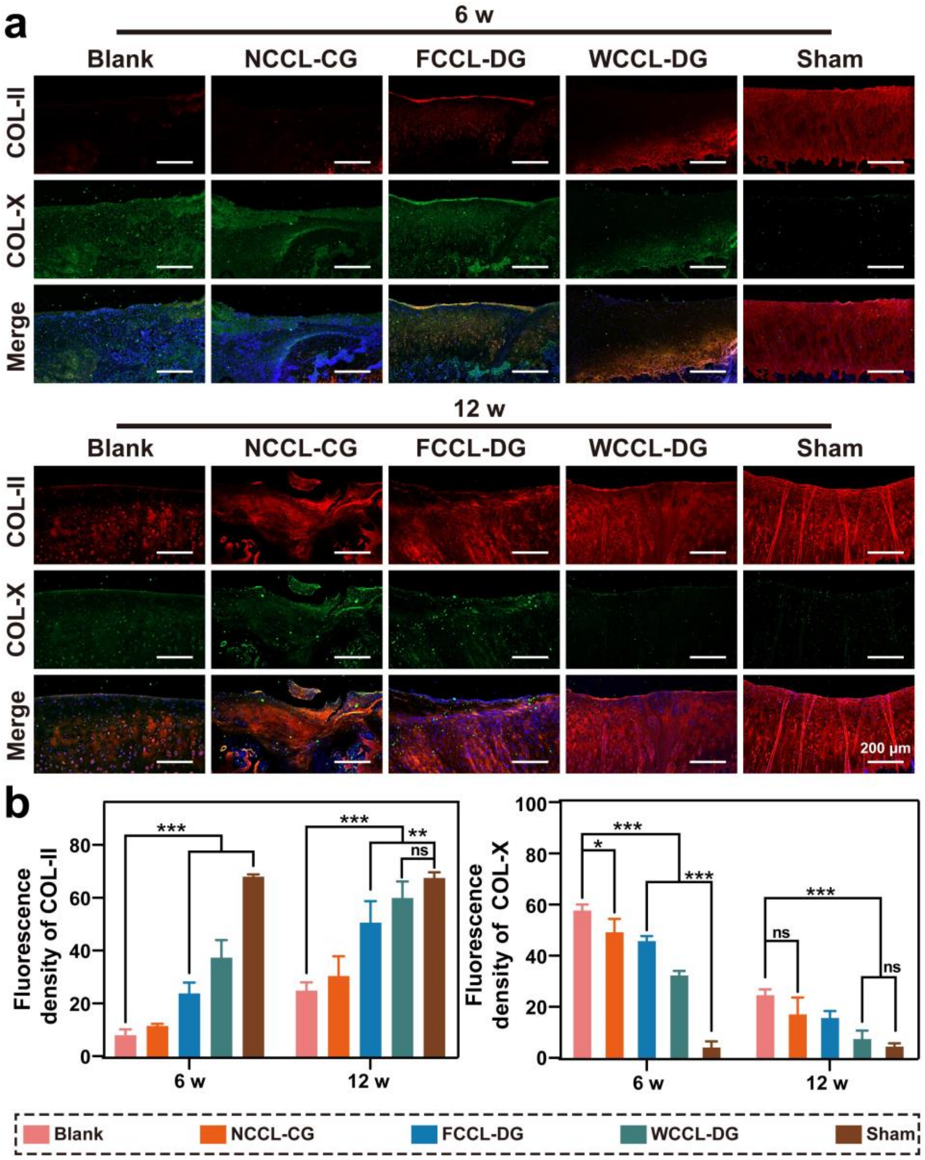


**Supplementary Fig. 34 Expression levels of COL-II and COL-X at specific time points for each group. a)** IF staining of COL-II and COL-X. **b)** Quantitative analysis of the fluorescence intensity in **a)**. Data were presented as means ± SD, n = 4. Statistical significance was determined using the one-way ANOVA with Tukey's post-hoc test. *$p < 0.05$, **$p < 0.01$, ***$p < 0.001$.

6. We have provided detailed explanations of the structure in the legend of Fig. 8a (as indicated by the red arrows) to ensure the understanding of the key observations.

Revision: "Black arrow: junction of old and new cartilage; green arrow: regenerative cartilage; red arrow: cells in the hypertrophic cartilage stage; black border: the enlarged areas indicated by the red arrow; S: residual hydrogels."

7. According to your suggestions, we have reorganized the manuscript and placed the rheological data and FEA results together in the revised manuscript. Additionally, to further validate WCCL-DG advantages over FCCL-DG, we have also added cyclic compression tests (fatigue resistance), stretch tests, and shear resistance tests in the updated Fig. 6 for a comprehensive analysis of hydrogel properties.

**Reviewer #2 (Remarks to the Author):**

The authors developed a sandwich-structured hydrogel scaffold featuring a biomimetic wavy calcified cartilage layer (CCL) via a decoupling strategy for full-thickness osteochondral defect repair. While the structural design of the scaffold shows some novelty and may offer a potential candidate solution for osteochondral regeneration, the manuscript exhibits significant limitations that currently preclude its acceptance for publication. Below are detailed comments for the authors' consideration:

1. The biological strategies involving $Mg^{2+}$, $Cu^{2+}$, and KGN to promote chondrogenesis, regulate vascularization, and inhibit chondrocyte hypertrophy are not entirely novel. The Introduction section fails to adequately highlight how this study advances beyond existing literature. For example, the use of these bioactive components has been previously reported in studies such as:

[1] Zheng L, Zhao S, Li Y, et al. Engineered MgO nanoparticles for cartilage-bone synergistic therapy. Sci Adv. 2024;10(10):eadk6084. doi:10.1126/sciadv.adk6084

[2] Yan J, Xia D, Zhou W, et al. pH-responsive silk fibroin-based CuO/Ag micro/nano coating endows polyetheretherketone with synergistic antibacterial ability, osteogenesis, and angiogenesis. Acta Biomater. 2020;115:220-234. doi:10.1016/j.actbio.2020.07.062

[3] Chen YR, Yan X, Yuan FZ, et al. Kartogenin-Conjugated Double-Network Hydrogel Combined with Stem Cell Transplantation and Tracing for Cartilage Repair. Adv Sci (Weinh). 2022;9(35):e2105571. doi:10.1002/advs. 202105571

**Reply 1:** We sincerely thanks for your insightful and constructive comments regarding the novelty of our study. We acknowledge that $Mg^{2+}$, $Cu^{2+}$, and KGN have been individually explored in cartilage and bone regeneration (Sci. Adv. 2024, 10, eadk6084; Acta Biomater. 2020, 115, 220; Adv. Sci 2022, 9, e2105571). However, our work distinctively integrates hierarchical structural design, mechanical optimization, and multi-pathway biological regulation into a single scaffold, rather than mere component stacking or single-function modification.

Our work highlights the comprehensive system combining decoupled spatiotemporal regulation, biomimetic interfacing, and sequential biological processes, thereby surpassing state-of-the-art approaches. The bidirectional soaking strategy not only constructed a biochemical gradient of $Mg^{2+}$ in the upper layer and $Cu^{2+}$ in the lower layer but also generated the adjustable pore size and modulus gradients, which could simultaneously regulate cell differentiation and structural-mechanical properties of hydrogel scaffolds. Furthermore, the scaffolds possessed inherent iron chelation capabilities to trigger a hypoxic microenvironment by activating HIF-1α. In the cartilage layer, HIF-1α worked in synergy with $Mg^{2+}$ to promote chondrogenic differentiation of BMSCs; while in the subchondral bone layer, HIF-1α worked in synergy with $Cu^{2+}$ to promote vasculogenesis, thereby facilitating the process of ECO and achieving the effect of homologous initiation - heterochronic differentiation. Notably, KGN in the cartilage layer prevents chondrocytes hypertrophy caused by long-term HIF-1α activation, representing a repurposing of KGN beyond its conventional use. Under this circumstance, this decoupling strategy can control HIF-1α expression spatially and

temporally, inhibiting hypertrophy in cartilage while promoting ECO in subchondral bone, beyond the existing literatures through integrated innovations that address unmet needs in osteochondral repair. Additionally, we innovatively developed a biomimetic wavey CCL, which effectively alleviates stress concentration and enhances shear resistance, addressing a critical gap in existing scaffolds that always overlook the biomechanical functions of natural CCL. It is the first time to fabricate a biomimetic wavy CCL interface and reveal its biomechanical transmission and cartilage protection function in addressing the challenges of stress concentration and interfacial detachment via the FEA and transcriptome studies, enabling the best therapeutic effect of full-thickness osteochondral defects.

Therefore, our design is not merely a simple stacking of individual active ingredients; instead, it organically integrates and synergizes the functions of each component within a single scaffold, which could effectively avoid the signal interference and achieve the coordinated regulation of "cartilage anti-hypertrophy and bone pro-vascularization" process. In the revised Introduction and Discussion sections, we have further elaborated the ingenious design by integrating these innovative elements into a single biomimetic scaffold for osteochondral repair.

Once again, we thank you for your valuable suggestions. We firmly believe this modification will significantly enhance the accuracy and persuasiveness of the introduction section of this study.

Revision: "While previous studies have explored individual bioactive components (e.g., $Mg^{2+}$ for cartilage-bone protection[6], $Cu^{2+}$ for vasculogenesis[7], KGN for chondrogenesis[8]), these efforts primarily focus on the single-component or single-tissue applications. However, during the complex process of osteochondral repair, it is necessary to precisely regulate the behaviors of different cells at various stages and in different locations (such as inhibiting the hypertrophy of chondrocytes in the cartilage layer while promoting vascularization in the subchondral bone layer). So, simply regulating a certain process is far from sufficient to meet the complex spatiotemporal requirements involved in osteochondral regeneration."

2. Although the wavy CCL design is inspired by natural anatomy, the regular waveform template used in this study does not fully recapitulate the irregular and heterogeneous architecture of native CCL. Quantitative morphological parameters (e.g., wavelength, amplitude) of native CCL should be provided and compared with the engineered structure to assess biomimetic fidelity. Future work should incorporate advanced imaging (e.g., micro-CT) and manufacturing techniques (e.g., 3D printing) to achieve higher structural accuracy.

**Reply 2:** We appreciate your insightful comments on the bionic fidelity of the wavy CCL design. Although we acknowledge that the conventional wave template cannot fully replicate the irregular structure of the natural CCL, the current design strategy was mainly focused on considering the balance between anatomical inspiration and experimental feasibility in small-animal models. The natural CCL of large mammals has spatial features that facilitate precise morphological quantification and template manufacturing, but applying it to small-animal models introduces inevitable limitations. The physiological thickness of the CCL in rabbit knee joints is only in the

single-digit micrometer range, significantly smaller than the 20-30 μm in humans (Nano Lett. 2022, 22, 2309). The alignment and size control of the irregular wave-shaped template in the restricted defect space present significant challenges, as maintaining accuracy at the microscale is difficult. Actually, this extremely small scale is incompatible with the technical contradiction of the bidirectional soaking strategy. These technical limitations affect the reproducibility of the experiments, which is a fundamental requirement for preclinical research. Therefore, we adopted the conventional waveform as a practical compromise solution to ensure consistent morphological structure and mechanical stability in the small-scale model, prioritizing the methodological reliability in the early research. Even so, this work is the first time to fabricate a biomimetic wavy CCL interface and reveal its biomechanical transmission and cartilage protection function in addressing the challenges of stress concentration and interfacial detachment via the finite element analysis and transcriptome studies, enabling the best therapeutic effect of full-thickness osteochondral defects.

In addition, the acquisition of such parameters (e.g., wavelength, amplitude) from native rabbit CCL is hindered by significant technical limitations. The primary challenge in obtaining quantitative morphological parameters of native rabbit CCL lies in its extremely small anatomical scale, which exceeds the resolution and precision limits of current preclinical research technologies. Physical isolation of intact CCL specimens is precluded by the tissue's delicate nature and intimate integration with adjacent structures, as even microsurgical dissection techniques risk structural disruption at this scale. This fragility, combined with the minute volume and small size of tissues, makes it impossible to obtain undamaged samples for subsequent quantitative analysis without introducing artifacts that compromise morphological fidelity. These inherent technical constraints currently prevent the reliable extraction of quantitative morphological data for direct comparison with engineered structures.

However, inspired by your valuable and insightful suggestions, we intend to use large-animal models such as goats and pigs and the most advanced high-resolution instruments (such as super-resolution microscopes, atomic force microscopes) to obtain the precise structural parameters of natural CCL in our future research. Then, we can systematically compare these data with engineered structures to rigorously evaluate the accuracy of bionics. Additionally, we also fully agree that technological advancements can play a crucial role in addressing the current limitations in small-animal CCL research and facilitating the clinical translation of osteochondral engineering. High-precision micro-CT scanners, with enhanced resolution and optimized contrast agents, will be capable of achieving non-invasive 3D reconstruction of natural structures, thereby quantifying previously difficult-to-obtain morphological parameters. The next generation of super-resolution microscopes will reveal microscopic structural details, including fiber directions and matrix gradients, which can guide the design of biomimetic scaffolds. Synchronous progress in the field of 3D bioprinting can also synergize with these imaging breakthroughs. The combination of high-resolution imaging with advanced printing modes such as multi-material extrusion and laser-assisted

printing will enable the fabrication of scaffolds with controllable porosity, stiffness, and bioactivity gradients, which can simulate the natural heterogeneity. This precision will facilitate cell infiltration, nutrient diffusion, and mechanical integration, thereby improving the *in vivo* repair effect. Through comparisons with natural morphology, the accuracy of the scaffolds will be verified, and a solid clinical translation foundation will be established. Consequently, the combination of advanced imaging technologies and 3D bioprinting techniques will ensure the construction of structurally accurate and functionally feasible scaffolds, thereby accelerating the transition from laboratory models to treatment solutions for osteochondral defects.

We sincerely appreciate your suggestions, which have pointed out the direction for our future research. These limitations regarding the absence of higher structural accuracy have been explicitly stated in the Discussion section of the revised manuscript.

Revision: "Nevertheless, our study also revealed some limitations and areas for the future work. First, although the current CCL was fabricated based on a template with a regular wave-like structure, its structural fidelity to the native CCL—characterized by irregular and heterogeneous architecture—remained limited. It was difficult to precisely scan the microscopic structure of rabbit CCL using conventional methods, which limited the reproduction of the authenticity of the biomimetic structures. Further efforts are required to capture the complex, non-uniform morphology of natural CCL through advanced scanning technologies that can precisely map its three-dimensional shape, size, and thickness. Subsequent fabrication via the advanced imaging (e.g., micro-CT) and sophisticated manufacturing technologies (e.g., 3D bioprinting) would enable the development of more biomimetic osteochondral scaffolds with higher structural accuracy and create CCL models with various combinations of amplitude, wavelength, and thickness parameters, better recapitulating the native tissue's structural complexity and functional properties. Through mechanical tests and FEA, the optimal parameters for stress transmission can be identified, thus providing crucial data support for the clinical translation of this biomimetic scaffold. Second, the present study was conducted using a rabbit femoral trochlea osteochondral defect model, which may not fully recapitulate the anatomical complexity, loading conditions, and pathological microenvironment of human osteochondral defects. Additionally, the current scaffold preparation was relied on a stepwise bidirectional soaking strategy. Since it was difficult to control the soaking depth within the limited height range of the osteochondral defects in the small rabbits, the accuracy was inevitably affected. So, the translation to clinical applications requires potent validation in larger animal models (such as goats and pigs) with more human-like joint structures and functional demands. Third, although the 12-week *in vivo* evaluation demonstrated promising regenerative outcomes, long-term assessments (e.g., 6–12 months) are also necessary to monitor tissue maturation, verify the stability and durability of the repair tissues, as well as the great potential for late-stage complications (e.g., ectopic calcification, inflammatory responses) under physiological loading conditions. Moving forward, we intend to build upon this work by employing more comprehensive detection methods to conduct thorough large-animal verification in subsequent

studies."

3. The claim that $Fe^{3+}$ chelation by PAA sustainably activates HIF-1α signaling lacks direct molecular evidence. Experiments such as prolyl hydroxylase (PHD) activity assays and quantification of downstream HIF-1α targets (e.g., VEGF, COL-II) are necessary to validate the proposed mechanism.

**Reply 3:** Thanks for your kind suggestions. We have noted that the experiments related to COL-II, such as IF staining and qRT-PCR determination of COL-II, have been included in Fig. 4. These results indicated that the activation of HIF-1α promoted the expression of COL-II, as the PAA-CHI hydrogel outperformed the CHI hydrogel in the chondrogenic differentiation of BMSCs. To further strengthen the evidence of HIF-1α activation mechanism, we have also studied the expression of stromal cell-derived factor 1α (SDF-1α), which is another key downstream target of HIF-1α and involved in tissue repair and cell recruitment. The results showed that PAA could efficiently chelate $Fe^{3+}$, thereby reducing the expression of PHD (Fig. 3d and Supplementary Fig. 22). The reduction in PHD activity alleviated its role in HIF-1α degradation, leading to an increase in HIF-1α protein levels. Therefore, the expression of downstream factors of HIF-1α, including SDF-1α and VEGF, was significantly upregulated (Supplementary Fig. 24). These results collectively indicated that PAA could efficiently activate the HIF-1α signaling pathway and induce a persistent hypoxic microenvironment *in situ*.

We have incorporated the detailed quantitative data on the expression of PHD, SDF-1α and VEGF into the revised manuscript to provide comprehensive evidence for the proposed mechanism, thereby enhancing the reliability of our research.

Revision: "As expected, after co-culture with PAA-CHI and WCCL-DG hydrogel scaffolds, PHD expression in BMSCs was significantly reduced, with levels markedly lower than that of the Ctrl and CHI groups (Fig. 3d and Supplementary Fig. 22). This reduction led to a higher HIF-1α expression in the PAA-CHI and WCCL-DG groups (Fig. 3e-g and Supplementary Fig. 23). Concomitantly, downstream targets of HIF-1α, including stromal cell-derived factor 1α (SDF-1α) and vascular endothelial growth factor (VEGF), were upregulated in these PAA-based hydrogels (Supplementary Fig. 24). These findings indicated that WCCL-DG hydrogel scaffolds could effectively mimic hypoxic microenvironment with remarkable hypoxia-inducible capacity to promote chondrogenesis."

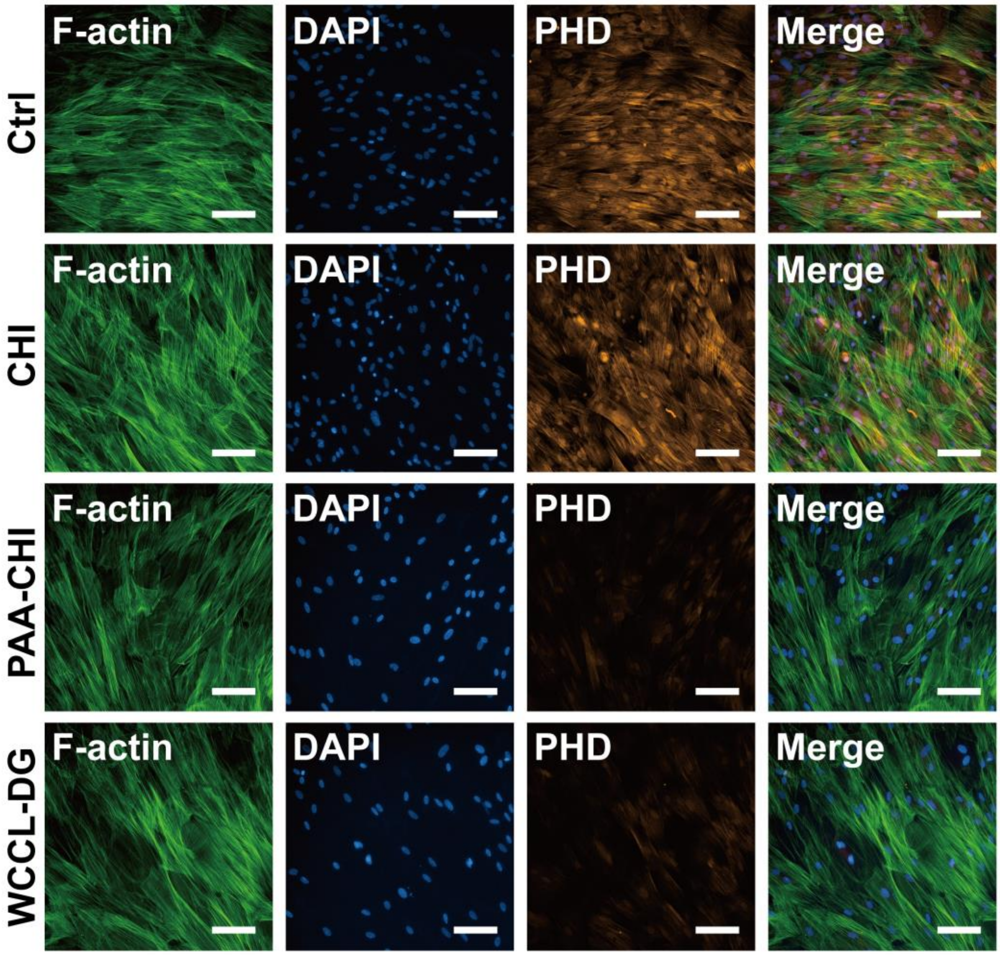


**Fig. 3 d)** Immunofluorescence staining images of PHD in BMSCs co-cultured with CHI, PAA-CHI, and WCCL-DG scaffolds at 24 h.

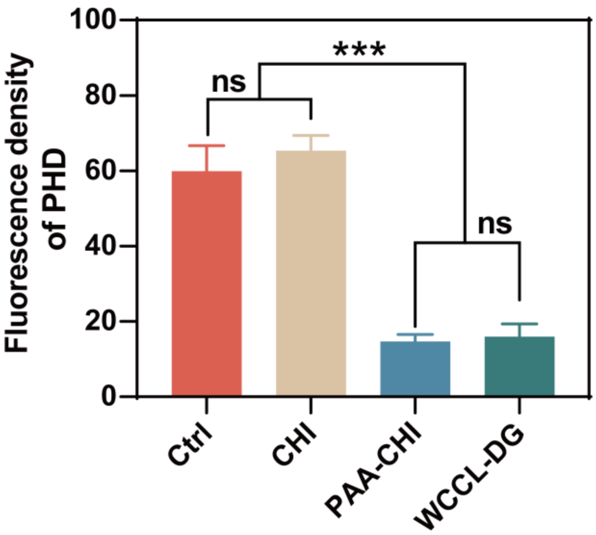


**Supplementary Fig. 22** Quantitative analysis of intensity of PHD in BMSCs co-cultured with CHI, PAA-CHI and WCCL-DG scaffolds. Data were presented as means ± SD, n = 3. Statistical significance was determined using the one-way ANOVA with Tukey's post-hoc test. $*p < 0.05$, $**p < 0.01$, $***p < 0.001$.

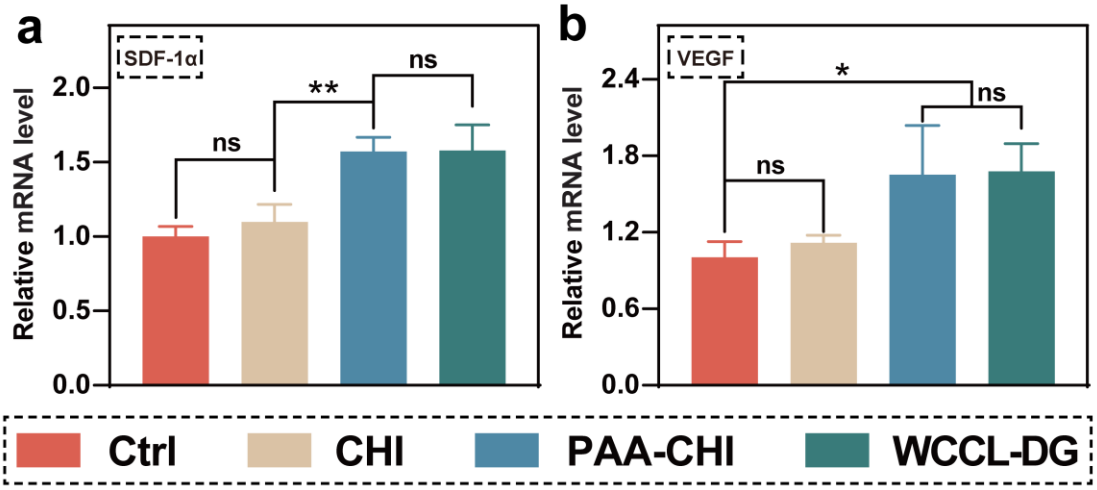


**Supplementary Fig. 24 Expression of downstream factors of HIF-1α on various hydrogels.** Relative mRNA expression of a) SDF-1α and b) VEGF of BMSCs co-culture with different hydrogels. Data in **a,b)** were presented as means ± SD, n = 3. Statistical significance was determined using the one-way ANOVA with Tukey's post-hoc test. *$p < 0.05$, **$p < 0.01$, ***$p < 0.001$.

4. The use of a rabbit femoral trochlear defect model alone is insufficient to demonstrate clinical translatability. Large animal models (e.g., sheep or minipigs) should be employed to evaluate the scaffold's efficacy under physiologically relevant loading conditions. Additionally, the 12-week evaluation period is too short to assess long-term outcomes. Extended studies (6–12 months) are needed to monitor tissue maturation, scaffold degradation, and potential complications (e.g., ectopic calcification, inflammatory responses).

**Reply 4:** We deeply appreciate your careful consideration of our work and fully recognize the critical importance of evaluating clinical translatability through large-animal models and extended observation periods. We would like to sincerely apologize to you for the limitations present in our current research. Specifically, there is a lack of large-animal experiments conducted under physiological-related load conditions, and the evaluation period is relatively short (only 12 weeks). These are indeed essential aspects for validating long-term efficacy and safety of hydrogel scaffolds, and we regret that we cannot address them comprehensively in the revised manuscript. On the one hand, a 3-month revision cycle is fundamentally inadequate for large-animal studies, which typically require 8–10 months due to sequential stages including ethical approval (4-6 weeks), animal acquisition and acclimatization (2-3 weeks), technically demanding surgeries, and extended postoperative monitoring period covering the initial recovery stage, a 6-month observation period and long-term evaluation of tissue regeneration, which collectively extend far beyond the constraints of a three-month window. On the other hand, the substantial cost of large-animal studies exceeds our current funding. Expenses include high animal procurement, specialized housing, surgical instrumentation, and long-term analytical follow-up. Our existing budget is committed to the small-scale research, and securing additional grants requires a months-long application process incompatible with the revision timeline.

We deeply regret that these actual constraints prevent us from incorporating your valuable

suggestions. However, we would like to assure you that large-animal models and long observation periods are the core components of our long-term research plan. This current study primarily aims to propose novel concepts regarding an innovative decoupling strategy to achieve the biological process of spatiotemporal regulation and a first demonstration of the wavy CCL with biomechanical interfacing to expand the contact area, distribute the load and alleviate stress concentration. The large-animal studies with long-term observation are indeed essential for future translational work, but the current manuscript mainly focuses on establishing the foundational principle and verifying its biomechanical effect, rather than achieving full preclinical validation. These limitations regarding the absence of *in-vivo* large-animal data have been explicitly stated in the Discussion section of the revised manuscript.

Revision: "Nevertheless, our study also revealed some limitations and areas for the future work. First, although the current CCL was fabricated based on a template with a regular wave-like structure, its structural fidelity to the native CCL—characterized by irregular and heterogeneous architecture—remained limited. It was difficult to precisely scan the microscopic structure of rabbit CCL using conventional methods, which limited the reproduction of the authenticity of the biomimetic structures. Further efforts are required to capture the complex, non-uniform morphology of natural CCL through advanced scanning technologies that can precisely map its three-dimensional shape, size, and thickness. Subsequent fabrication via the advanced imaging (e.g., micro-CT) and sophisticated manufacturing technologies (e.g., 3D bioprinting) would enable the development of more biomimetic osteochondral scaffolds with higher structural accuracy and create CCL models with various combinations of amplitude, wavelength, and thickness parameters, better recapitulating the native tissue's structural complexity and functional properties. Through mechanical tests and FEA, the optimal parameters for stress transmission can be identified, thus providing crucial data support for the clinical translation of this biomimetic scaffold. Second, the present study was conducted using a rabbit femoral trochlea osteochondral defect model, which may not fully recapitulate the anatomical complexity, loading conditions, and pathological microenvironment of human osteochondral defects. Additionally, the current scaffold preparation was relied on a stepwise bidirectional soaking strategy. Since it was difficult to control the soaking depth within the limited height range of the osteochondral defects in the small rabbits, the accuracy was inevitably affected. So, the translation to clinical applications requires potent validation in larger animal models (such as goats and pigs) with more human-like joint structures and functional demands. Third, although the 12-week *in vivo* evaluation demonstrated promising regenerative outcomes, long-term assessments (e.g., 6–12 months) are also necessary to monitor tissue maturation, verify the stability and durability of the repair tissues, as well as the great potential for late-stage complications (e.g., ectopic calcification, inflammatory responses) under physiological loading conditions. Moving forward, we intend to build upon this work by employing more comprehensive detection methods to conduct thorough large-animal verification in subsequent studies."

5. The compressive and shear tests provided are inadequate to simulate the complex joint environment. Fatigue testing and tribological assessments should be conducted to evaluate the scaffold's durability and friction properties under cyclic loading.

**Reply 5:** Thanks for your valuable suggestion regarding the need for comprehensive mechanical evaluations to simulate the complex joint environment. To better mimic the dynamic compressive environment within joints, we supplemented cyclic compression tests for both WCCL-DG and FCCL-DG hydrogels at strain levels of 10%, 20%, and 30% to assess their fatigue resistance. The results demonstrated that WCCL-DG maintained excellent mechanical stability across all strain conditions. Its peak stress remained consistently between 90% and 100% of the initial value throughout the cyclic loading process. In contrast, FCCL-DG exhibited a continuous decline in peak stress with increasing cycles, indicating that the wavy interface design of WCCL-DG significantly improved its fatigue resistance compared to the flat interface of FCCL-DG (Fig. 6a-c).

Furthermore, we also evaluated the interface stability of both hydrogels through stretch and shear tests. Ashby's analysis indicated that the fracture resistance of WCCL-DG was significantly superior to that of FCCL-DG (Fig. 6e). This enhancement could be attributed to its wavy interface, which increased the contact area between the upper and lower layers, thereby effectively enhancing the fracture strength of WCCL-DG. Additionally, the mechanical interlocking effect caused by the wavy interface also significantly improved the friction resistance of WCCL-DG. This effect enabled the scaffold to maintain structural stability even under higher shear forces, which was crucial for withstanding the complex biomechanical stresses in natural joints.

These supplementary mechanical evaluations confirm that the wavy interface design of WCCL-DG not only enhances fatigue resistance but also improves interfacial stability and friction properties, making it a more robust scaffold for osteochondral regeneration in dynamic joint environments. These detailed results have been added in the revised manuscript to provide a comprehensive assessment of the scaffold's mechanical performance.

Revision: "To better mimic the dynamic compressive environment within joints, we used multi-dimensional experiments to characterize the effect of CCL with distinct geometries on the mechanical properties of the scaffolds. In the cyclic compression test, WCCL-DG hydrogel maintained excellent repeatability during 30 test cycles under different strains (10%, 20%, and 30%), indicating its outstanding mechanical stability and fatigue resistance (Fig. 6a,c). This advantage stemmed from the wavy interface geometry of the WCCL-DG hydrogel, and its uneven peak-valley structures on its surface itself contributed to the elasticity of restoring to the original state after compression. In contrast, the peak stress of the FCCL-DG hydrogel gradually decreased because its flat interface lacked this geometric deformation recovery capability, resulting in cumulative structural deformation (Fig. 6b,c). This rebound characteristic mediated by the geometric structure simulated the dynamic mechanical response of the natural articular cartilage, supporting the continuous structural integrity of the regenerated tissues under physiological cyclic loads[53]."

"For interface stability, we characterized the interfacial bonding strength in horizontal and vertical

directions using shear and stretch tests (Fig. 6e). The Ashby plot clearly contrasted the shear and stretch resistance of the WCCL-DG and the FCCL-DG hydrogel scaffolds, demonstrating that WCCL-DG outperforms FCCL-DG in both stretch and shear resistance. In terms of stretch resistance, the wave interface increased the interlayer contact area and enhanced the interlayer bonding, thus improving the structural integrity under stretch loads. In terms of shear strength, the wave structure had created a mechanical interlocking effect, partially preventing the detachment of the cartilage layer induced by lateral shear stress, which was more significant in enhancing the overall shear resistance of the hydrogel scaffold compared to its stretch strength."

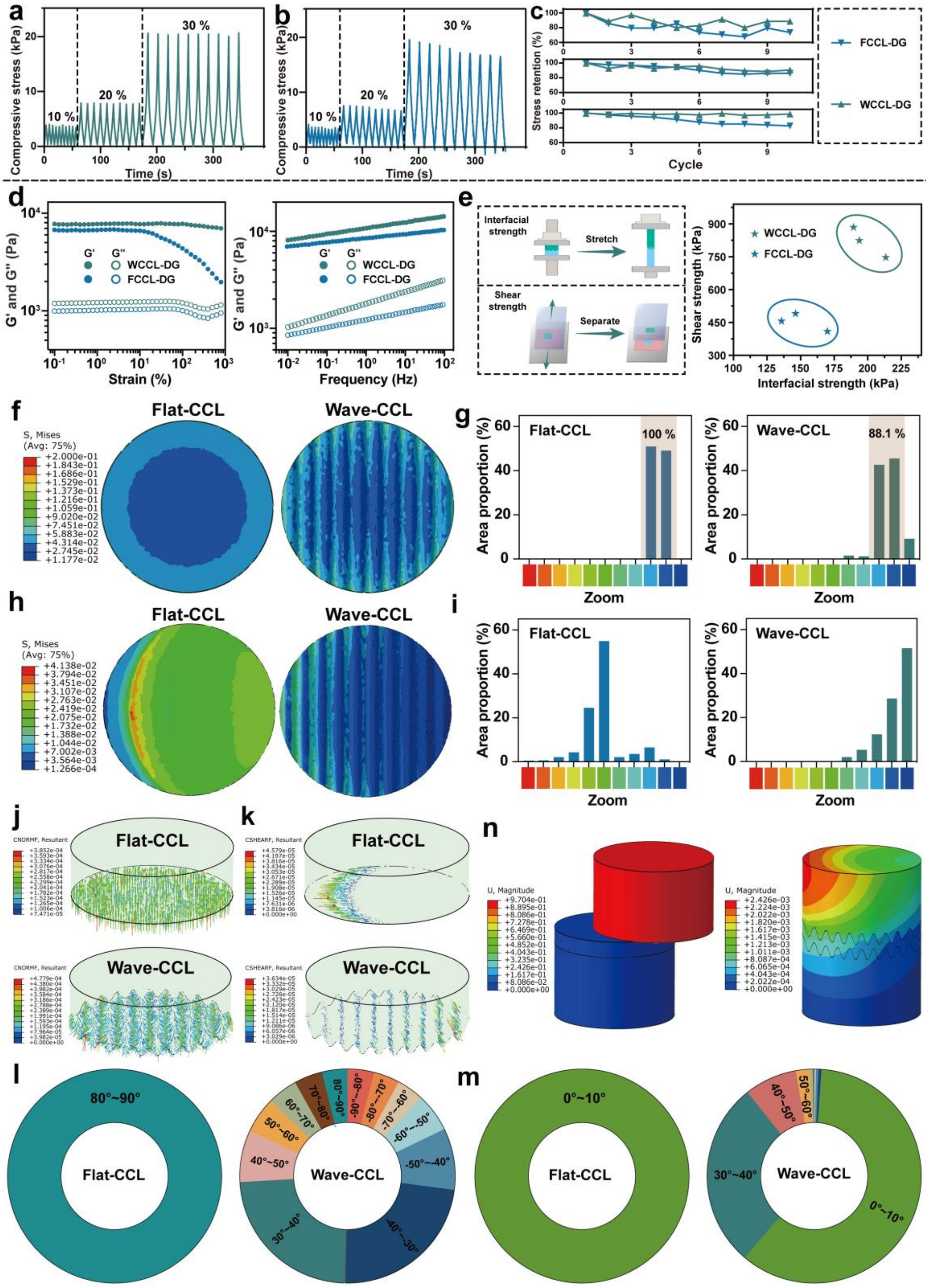


**Fig. 6 The mechanical differences between WCCL-DG and FCCL-DG hydrogels. a)** Cyclic

compression curves of WCCL-DG hydrogel under different strains. **b)** Cyclic compression curves of FCCL-DG hydrogel under different strains. **c)** Stress retention conditions of WCCL-DG and FCCL-DG hydrogels during the cyclic compression process. **d)** Rheological properties of WCCL-DG and FCCL-DG hydrogels. **e)** Stretch and shear properties of WCCL-DG and FCCL-DG hydrogels. **f)** Thermograms of stress distribution on the lower surface of the cartilage layer when two models were subjected to a compressive stress of 0.1 MPs respectively. **g)** Statistical analysis of the proportion of area occupied by different color blocks in **f)**. **h)** Thermograms of stress distribution on the lower surface of the cartilage layer when two models were subjected to a shear stress of 0.1 MPs respectively. **i)** Statistical analysis of the proportion of area occupied by different color blocks in **h)**. **j)** Direction of stress in the lower surface of the cartilage layer when subjected to compressive stress in two models. **k)** Direction of stress in the lower surface of the cartilage layer when subjected to shear stress in two models. **l)** Statistical analysis of stress of different ranges in **j)**. **m)** Statistical analysis of stress of different ranges in **k)**. **n)** Thermal map of overall displacement under shear stress for two models.

6. Comparisons with only flat-interface scaffolds (FCCL-DG) are insufficient. A no-CCL control group should be included to directly demonstrate the functional necessity of the wavy CCL.

**Reply 6:** We appreciate your attention to this critical control design, and we would like to clarify that a control group without CCL was indeed included in our study. We deeply regret that the original naming convention caused misunderstanding and appreciate your vigilance in identifying this issue. In fact, the group initially labeled as FCCL-CG was designed to serve as the no-CCL control, which was a simple composite hydrogel without any ionic soaking treatments. As a result, FCCL-CG is purely a bilayer hydrogel structure and does not contain a CCL. To avoid further confusion, no-CCL group has been updated as NCCL-CG group in the revised manuscript. Also, we have systematically described the naming conventions and key characteristics of all hydrogel formulation for reader comprehension in the revised Supplementary Table 1.

Revision:

**Supplementary Table 1.** Naming conventions for hydrogel groups.

| Abbreviation | Full name | Does it include the CCL layer and its form | Characteristics |
|---|---|---|---|
| NCCL-CG | None Calcified Cartilage Layer-Composite Gel | No | Double-layer hydrogel, not subjected to ion soaking |
| FCCL-DG | Flat Calcified Cartilage Layer-Double-network Gel | Yes，flat | Double-layer hydrogel subjected to bidirectional ion soaking |
| WCCL-DG | Wave Calcified Cartilage Layer-Double-network Gel | Yes，wave | Double-layer hydrogel prepared by a wave-shaped template subjected to bidirectional ion soaking |

7. The naming system for hydrogel groups (e.g., WCCL-DG, FCCL-DG, PAA-CHI-KGN-$Mg^{2+}$) is confusing. A table or detailed glossary should be provided to clarify the naming conventions.

**Reply 7:** Thanks for your kind suggestions. We have provided it accordingly in the revised Supplementary Table 1, which includes key characteristics of each hydrogel such as their structural components and functional features as well as an overview of their preparation processes. Specifically, for those abbreviated as CCL-DG, they all referred to the sandwich-layered double-network gel scaffold after being soaked in salt solutions, but it could be either a wave (WCCL) or a flat structure (FCCL). For the spelling descriptions such as PAA-CHI-KGN and PAA-CHI-KGN-$Mg^{2+}$, it refers to a single hydrogel, not a multi-layer scaffold. We hope this addition resolves the confusion regarding the hydrogel group nomenclature and improves the overall clarity of the manuscript. Thanks again for your constructive feedback.

8. Some figures do not specify whether multiple comparison corrections (e.g., Tukey's or Bonferroni's tests) were applied. This should be clearly stated in the figure legends and methods section.

**Reply 8:** Thanks for your kind suggestions. We sincerely apologize for the oversight in the legend and methods section where the use of multiple comparison correction methods (such as Tukey's test or Bonferroni's test) was not clearly indicated. To address this issue, we have added clear explanations in the legend of the main text and supplementary figures for multiple comparisons to ensure transparency. Additionally, we have updated the statistical analysis section in the Methods section accordingly in the revised manuscript.

Revision: Statistical significance was determined using the one-way ANOVA with Tukey's post-hoc test. $*p < 0.05$, $**p < 0.01$, $***p < 0.001$.

**Statistical analysis**

All data were expressed as means ± standard deviation of three representative experiments. The sample size or number of biological replicates was shown in the figure legend. The stability of each result was verified by at least three independent experiments. Correlations between dependent variables were analyzed by two-tailed Student's t-test for two groups or one-way ANOVA followed by Tukey's post-hoc test for three or more groups, $p < 0.05$ was considered statistically significant.

9. The current manufacturing process, relying on wavy templates and stepwise soaking, is complex and may lack reproducibility. Process optimization is needed to ensure scalability and standardization for potential clinical translation.

**Reply 9:** Thanks for your kind suggestions. As the first study to introduce a wavy interface for calcified cartilage layer reconstruction in osteochondral scaffolds, our primary goal was to propose a biomimetic concept addressing the critical gap in existing literatures, where most studies either omit the CCL or use planar substitutes, neglecting the biomechanical significance of the native wavy interface. Even so, we acknowledge that the current process based on wave templates and stepwise immersion is still in its infancy and has inherent limitations. Regarding the variability between scaffolds, this issue is partially attributed to the constraints of using small-animal models, where

minor dimensional differences in scaffolds may arise. This issue has been explicitly discussed in the manuscript, and we anticipate that scaling to large-animal models will mitigate such variability, as the increased scaffold size for larger implants will enhance manufacturing consistency. Furthermore, we are also highly interested in advanced manufacturing technologies such as 3D bioprinting. However, we have encountered numerous difficulties in recreating micro-scale wavy structures using this method. Especially, the acquisition of such parameters (e.g., wavelength, amplitude) from native rabbit CCL is hindered by significant technical limitations. The primary challenge in obtaining quantitative morphological parameters of native rabbit CCL lies in its extremely small anatomical scale, which exceeds the resolution and precision limits of current preclinical research technologies. As a microscale tissue structure, the native rabbit CCL exhibits minimal spatial dimensions that render conventional sample preparation and imaging methodologies inherently inadequate. So, it requires a dedicated multi-material printer with an independent deposition head, customized CAD models, and extensive parameter optimization for each tissue type and ion combination. To adapt to new applications or ion systems, the printing process needs to be completely reprogrammed, and iterative tests lasting several weeks are required to recalibrate the material deposition rate and cross-linking parameters. Therefore, although we have repeatedly attempted to write 3D bioprinting parameters for the wavy CCL interface, but the geometric complexity of tiny CCL makes 3D bioprinting inefficient in developing CCL-containing gradient scaffolds across multiple scenarios at present. Instead, our bidirectional soaking strategy and wave template as a practical alternative has wide applicability and minimal resource consumption.

Of note, for the manufacturing of $Mg^{2+}$/$Cu^{2+}$ gradient scaffolds, the bidirectional soaking strategy boasts unparalleled operational simplicity and flexibility, transcending the limitations of a single application. Moreover, in addition to being used for osteochondral repair, this method can precisely adapt to various clinical needs through a dual regulation mechanism. By adjusting the vertical soaking height of the scaffold in the ionic solutions, the steepness of the gradient can be precisely controlled. Furthermore, simply by changing the type of ions in the soaking solutions, such as replacing $Mg^{2+}$/$Cu^{2+}$ with $Ca^{2+}$/$Sr^{2+}$ for bone regeneration or replacing them with $Zn^{2+}$/$Si^{4+}$ for tendon-bone interface repair, it further expands its application scope. This dual flexibility ensures that this soaking strategy can meet the different ionic requirements in various tissue repair scenarios, whether it is using $Mg^{2+}$ to promote chondrocytes differentiation or $Cu^{2+}$ to enhance osteogenic activity, no specialized equipment or complex procedural operations are required. Basic laboratory tools are sufficient to adjust the gradient parameters and ion composition, ensuring a rapid response to the requirements of various tissue microenvironments.

We fully recognize that optimizing the manufacturing process for clinical translation is of great necessity. Next, we intend to formulate a biomimetic template based on the natural or large animal CCL form to reduce the differences. We will utilize a programmable system to automate the soaking process to standardize the formation of gradients, and explore a hybrid method that combines precision and structural complexity. Thanks again for your feedback, which is invaluable for

refining our approach and accelerating the translation of this biomimetic concept.

10. The immunogenic potential of the scaffold and the safety of its degradation products were not evaluated. Comprehensive toxicological studies are essential before clinical application.

**Reply 10:** Thanks for your kind suggestions. To assess the immunogenicity of the scaffold and the safety of its degradation products, we conducted a subcutaneous implantation experiment in rats. We performed HE staining on important organs such as the heart, liver, spleen, lungs, and kidneys, and conducted biochemical index tests at 2 weeks and 4 weeks after the surgery (Supplementary Fig. 19). The results showed that there were no obvious pathological abnormalities in each individual, preliminarily confirming the biological safety of those scaffolds.

Revision: "To verify the *in vivo* biocompatibility and degradability of different scaffolds, we conducted subcutaneous implantation tests in rats (Supplementary Fig. 18). Since WCCL-DG and FCCL-DG only differed in structure, we selected WCCL-DG for further investigation. After subcutaneous implantation for one month, we did not observe any obvious tissue damage or morphological changes, and the various indicators showed no significant differences compared to those of normal rats (Supplementary Fig. 19), indicating good biocompatibility of those scaffolds."

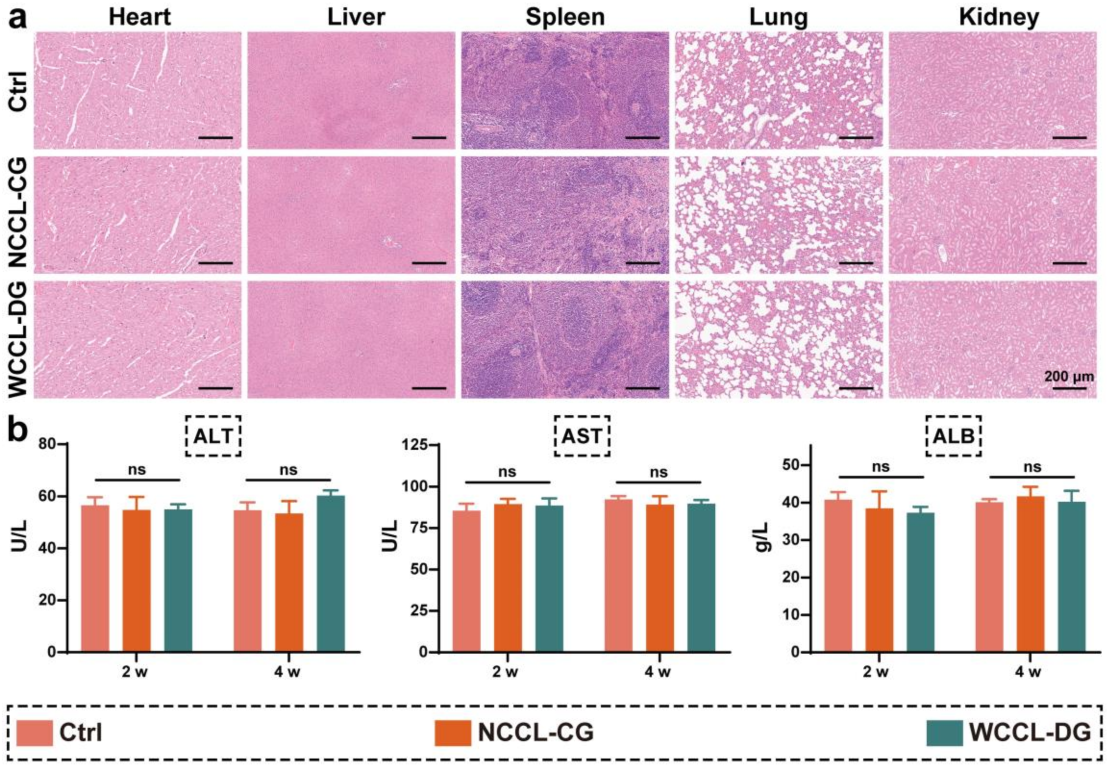


**Supplementary Fig. 19 Biological safety verification of hydrogel scaffolds. a)** Histological evaluation of rats' organs (Heart, Liver, Spleen, Lung, and Kidney). Representative H&E staining images of rat's organs following 1 month of *in vivo* hydrogel implantation. The experiment was repeated four times with similar results. **b)** The liver function test results at different time points, including alanine aminotransferase (ALT), aspartate aminotransferase (AST), and serum albumin (ALB). Data were presented as means ± SD, n = 4. Statistical significance was determined using the one-way ANOVA with Tukey's post-hoc test. *$p < 0.05$, **$p < 0.01$, ***$p < 0.001$.

11. The Discussion section should be strengthened to directly compare this study's strategies with existing technologies (e.g., 3D-printed gradient scaffolds). The authors should emphasize how their integrated system—combining decoupled spatiotemporal regulation, biomechanical interfacing, and sequential biological processes—surpasses current state-of-the-art approaches.

**Reply 11:** We sincerely appreciate your valuable suggestions. We have accordingly strengthened the direct comparison between our strategy and existing technologies, particularly 3D-printed gradient scaffolds. Current 3D-printed gradient scaffolds always require complex fabrication processes, expensive equipment, and precise parameter control, leading to high manufacturing costs and potential interfacial delamination issues at the layer interfaces. In contrast, our bidirectional ion soaking strategy simplifies the preparation of multilayered scaffolds and eliminates the risk of interfacial delamination, and stepwise ion soaking can easily achieve continuous gradient formation without the need for expensive instruments.

More importantly, we have emphasized our integrated system combining decoupled spatiotemporal regulation, biomimetic interfacing, and sequential biological processes to surpasses state-of-the-art approaches. Our bidirectional soaking strategy not only constructed a biochemical gradient of $Mg^{2+}$ in the upper layer and $Cu^{2+}$ in the lower layer but also generated the adjustable pore size and modulus gradients, which could simultaneously regulate cell differentiation and structural-mechanical properties of hydrogel scaffolds. Furthermore, the scaffolds possessed inherent iron chelation capabilities to trigger a hypoxic microenvironment by activating HIF-1α. In the cartilage layer, HIF-1α worked in synergy with $Mg^{2+}$ to promote chondrogenic differentiation of BMSCs; while in the subchondral bone layer, HIF-1α worked in synergy with $Cu^{2+}$ to promote vasculogenesis, thereby facilitating the process of ECO and achieving the effect of homologous initiation - heterochronic differentiation. Notably, KGN in the cartilage layer prevents chondrocytes hypertrophy caused by long-term HIF-1α activation, representing a repurposing of KGN beyond its conventional use. Under this circumstance, this decoupling strategy can control HIF-1α expression spatially and temporally, inhibiting hypertrophy in cartilage while promoting ECO in subchondral bone, beyond the existing literatures through integrated innovations that address unmet needs in osteochondral repair. Additionally, we innovatively developed a biomimetic wavey CCL, which effectively alleviates stress concentration and enhances shear resistance, addressing a critical gap in existing scaffolds that always overlook the biomechanical functions of natural CCL. It is the first time to fabricate a biomimetic wavy CCL interface and reveal its biomechanical transmission and cartilage protection function in addressing the challenges of stress concentration and interfacial detachment via the FEA and transcriptome studies, enabling the best therapeutic effect of full-thickness osteochondral defects.

These comparisons and elaborations have been added into the Discussion section according to your constructive comments, which will significantly improve the quality of our manuscript.

Revision: "Current 3D-printed gradient scaffolds always require complex fabrication processes, expensive equipment, and precise parameter control, leading to high manufacturing costs and

potential interfacial delamination issues at the layer interfaces[58, 59, 60]. In contrast, our bidirectional ion soaking strategy simplifies the preparation of multilayered scaffolds and eliminates the risk of interfacial delamination, and stepwise ion soaking can easily achieve continuous gradient formation without the need for expensive instruments."

"Compared with current state-of-the-art technologies such as cartilage-like modulus degradable hydrogels or gradient scaffolds, our system's core advantage lies in integrating rarely combined functional elements that address key gaps in these methods. As far as we know, most advanced methods are relatively simple in function and lack integrated regenerative capabilities. For example, gradient scaffolds always rely on the fixed biochemical cues but lack targeted control, and many modulus-matched hydrogels only meet mechanical requirements but neglect the reconstruction of wavy CCL with key mechanical properties, not to mention the regulation of differentiation signals. In contrast, our system unites three critical functions of decoupled spatiotemporal control, biomimetic biomechanical interface, and sequential biological processes, thereby surpassing state-of-the-art approaches. The bidirectional soaking strategy not only constructed a biochemical gradient of $Mg^{2+}$ in the upper layer and $Cu^{2+}$ in the lower layer but also generated the adjustable pore size and modulus gradients, which could simultaneously regulate cell differentiation and structural-mechanical properties of hydrogel scaffolds. Furthermore, the scaffolds possessed inherent iron chelation capabilities to trigger a hypoxic microenvironment by activating HIF-1α. In the cartilage layer, HIF-1α worked in synergy with $Mg^{2+}$ to promote chondrogenic differentiation of BMSCs; while in the subchondral bone layer, HIF-1α worked in synergy with $Cu^{2+}$ to promote vasculogenesis, thereby facilitating the process of ECO and achieving the effect of homologous initiation - heterochronic differentiation. Notably, KGN in the cartilage layer prevented chondrocytes hypertrophy caused by long-term HIF-1α activation, representing a repurposing of KGN beyond its conventional use. Under this circumstance, this decoupling strategy could control HIF-1α expression spatially and temporally, inhibiting hypertrophy in cartilage while promoting ECO in subchondral bone, beyond the existing literatures through integrated innovations that addressed unmet needs in osteochondral repair. Additionally, we innovatively developed a biomimetic wavey CCL, which effectively alleviated stress concentration and enhanced shear resistance, addressing a critical gap in existing scaffolds that overlooked the biomechanical functions of natural CCL. It was the first time to fabricate a biomimetic wavy CCL interface and reveal its biomechanical transmission and cartilage protection function in addressing the challenges of stress concentration and interfacial detachment via the FEA and transcriptome studies, enabling the best therapeutic effect of full-thickness osteochondral defects."

Finally, we sincerely thanks for your meticulous review of our manuscript and your constructive suggestions, and hope our explanation can get your understanding and recognition. We recognize that this feedback is of great importance in further refining and enhancing our research outcomes.

**Reviewer #3 (Remarks to the Author):**

I co-reviewed this manuscript with one of the reviewers who provided the listed reports. This is part of the Nature Communications initiative to facilitate training in peer review and to provide appropriate recognition for Early Career Researchers who co-review manuscripts.

**Reply:** We sincerely thanks for your meticulous review of our manuscript and your constructive suggestions, and hope our explanation can get your understanding and recognition. We recognize that this feedback is of great importance in further refining and enhancing our research outcomes.

**Reviewer #4 (Remarks to the Author):**

This study presents a bilayer hydrogel scaffold with a wavy calcified cartilage layer to mimic the osteochondral interface. Using spatial ion gradients and KGN delivery, the authors direct chondrogenic versus hypertrophic differentiation. Although the work is creative and supported by extensive *in vitro* and *in vivo* data, the following key issues require clarification to meet the journal's standards:

1. Please clarify the subcutaneous degradation profile of the hydrogel material. Animal experimental results appear to indicate rapid degradation within 6 weeks, but this does not seem to be the case for PAA and chitosan.

**Reply 1:** We sincerely appreciate your valuable suggestions regarding the subcutaneous degradation profile of the hydrogels. We have conducted additional *in vivo* degradation experiments to confirm that the remaining mass of both hydrogels was 40%-50% after 6 weeks of subcutaneous implantation (Supplementary Fig. 20). Compared to *in vitro* degradation and *in vivo* subcutaneous degradation, the faster degradation rate in joint cavity was mainly due to the following reasons. Firstly, the encapsulation by the fibrous capsule after subcutaneous implantation inevitably slowed down the degradation rate of the hydrogel scaffold. Additionally, there were many degradable enzymes (such as collagenase and matrix metalloproteinase) within the joint, which further accelerated the decomposition of hydrogel scaffold. More importantly, in order to further demonstrate the biological function of the wavy CCL, we encouraged the rabbits to actively engage in running and jumping exercises after the surgery. This would exert pressure on the hydrogel scaffolds, further accelerating its degradation process. In addition, the degradation of PAA- or chitosan-based hydrogels within 6 weeks have also been found in the previous literatures (Adv. Sci. 2022, 9, e2105571; Biomaterials. 2016, 108, 157; Adv. Funct. Mater. 2021, 31, 2008515; Carbohydr. Polym. 2025, 347, 122643; J. Mater. Chem. 2021, 9, 9370). We have included these detailed degradation data accordingly in the revised manuscript.

Revision: "To verify the *in vivo* biocompatibility and degradability of different scaffolds, we conducted subcutaneous implantation tests in rats (Supplementary Fig. 18). Since WCCL-DG and FCCL-DG only differed in structure, we selected WCCL-DG for further investigation. After subcutaneous implantation for one month, we did not observe any obvious tissue damage or morphological changes, and the various indicators showed no significant differences compared to

those of normal rats (Supplementary Fig. 19), indicating good biocompatibility of those scaffolds. For the *in vivo* degradation behavior of WCCL-DG and NCCL-CG, both scaffolds exhibited a measurable weight gain attributed to their swelling response in the physiological microenvironment during the first week post-implantation. Subsequently, they exhibited a trend of continuous degradation, manifested as a gradual decrease in weight and a corresponding reduction in volume during the subsequent observation period (Supplementary Fig. 20). Interestingly, we found that the degradation rate of the hydrogel scaffold in the joint cavity was faster compared to the subcutaneous degradation. This was because the encapsulation effect of the fibrous capsule after subcutaneous implantation inevitably slowed down the degradation rate of the hydrogel scaffolds. Additionally, there were many degradable enzymes (such as collagenase and matrix metalloproteinase) within the joint, which further accelerated the decomposition of the hydrogel scaffolds. More importantly, in order to further demonstrate the biological function of the wavy CCL, we encouraged the rabbits to actively engage in running and jumping exercises after the surgery. This would exert pressure on the hydrogel scaffold, further accelerating its degradation process."

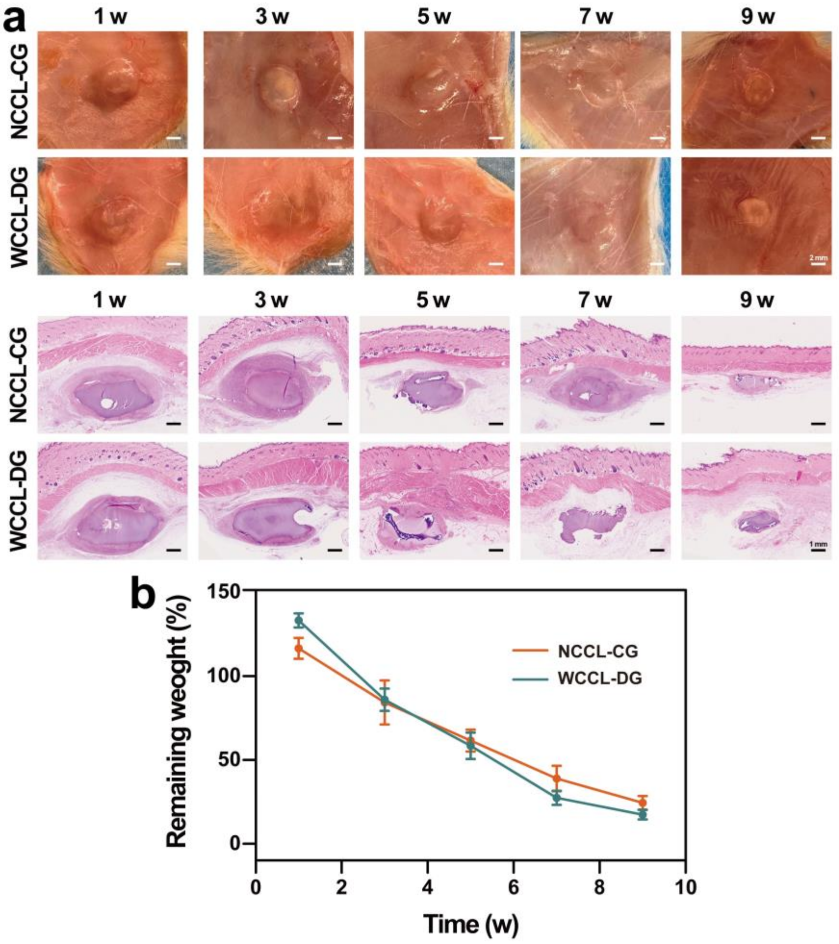


**Supplementary Fig. 20 Subcutaneous degradation behaviors of WCCL-DG and NCCL-CG scaffolds. a)** Macrograph and H&E staining images of WCCL-DG and NCCL-CG scaffolds with the surrounding skin at the different time point. **b)** The mass remained of WCCL-DG and NCCL-CG scaffolds at the different time point. Data in **b)** were presented as means ± SD, n = 3.

2. For subcutaneous implantation, please provide H&E staining images, preferably with 3-month

results to substantiate material degradation and whether cells infiltrate the material.

**Reply 2:** We sincerely thanks for your valuable comment. Regretfully, due to the 3-month revision cycle constraint, we are unable to add the subcutaneous implantation data for the next 3 months. However, we have included H&E staining images at 1 week, 3 weeks, 5 weeks, 7 weeks, and 9 weeks post-implantation. At 5-week, the hydrogel exhibited fragmentation and disintegration, with cells proliferating in the gaps, indicating the ideal biocompatibility and repair potentials of WCCL-DG. These sequential images clearly illustrate the continuous degradation process of implanted scaffolds (Supplementary Fig. 20).

3. For SEM image (Figure 3a), it is recommended to add false coloring to better illustrate the interface and multilayer structure.

**Reply 3:** Thanks for your kind suggestions. Your opinion is of great value in optimizing the presentation of our structural feature analysis results and ensuring the clear expression of key details. We have applied false-color processing to the SEM images to better display the interfaces and multi-layer structures in the revised manuscript (Fig. 3a). This modification significantly enhances the visibility of interface morphology and layered arrangement, making structural details more intuitive.

Revision:

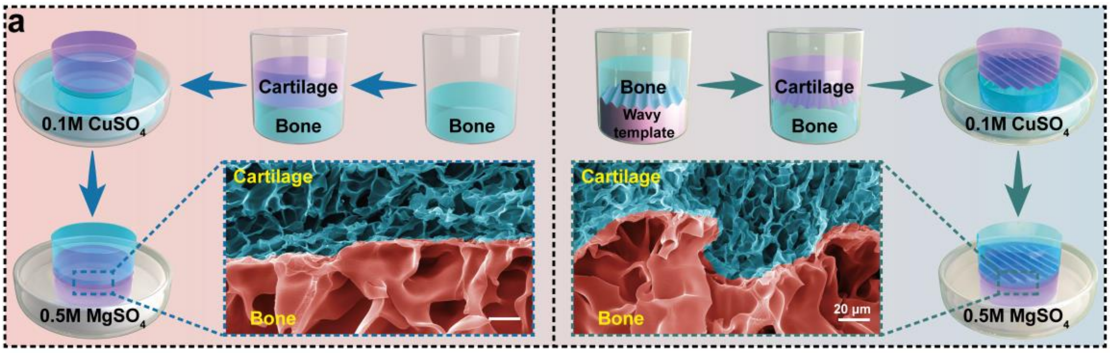


**Fig. 3 a)** Preparation process of FCCL-DG and WCCL-DG scaffolds with structural variance at the junctions.

4. The metal ion release experiment in vitro should be supplemented, because the co culture of cells and hydrogels also has good biological effects.

**Reply 4:** We sincerely appreciate your valuable comments. We have supplemented the *in vitro* metal ion release experiment accordingly in the revised manuscript (Supplementary Fig. 5). Both $Mg^{2+}$ and $Cu^{2+}$ from the WCCL-DG hydrogel scaffold exhibit the sustained release profiles over time. This sustained release is critical for maintaining long-term biological activity and supporting the dynamic needs of osteochondral regeneration, as it ensures continuous provision of bioactive cues for cell differentiation and tissue formation.

For $Mg^{2+}$, the cartilage layer hydrogel can release $Mg^{2+}$ in a sustainable manner. By screening the soaking concentration, we achieve an optimal $Mg^{2+}$ release level to promote chondrogenic differentiation of BMSCs (200 ppm), this sustained stimulation has been verified via *in vitro* experiments (Fig. 2). For $Cu^{2+}$, due to the stronger coordination ability of $Cu^{2+}$ with PAA carboxyl groups compared to $Mg^{2+}$ and the lower soaking concentration of the subchondral bone layer, the

release rate of $Cu^{2+}$ is significantly slower than that of $Mg^{2+}$. The *in vitro* results indicate that $Cu^{2+}$ also possesses the ability for sustainable release. Furthermore, this sustained release aligns with the rhythm of the ECO process in the subchondral bone layer as confirmed by our tissue section staining results. At 6 weeks post-operation, chondrocytes enter the hypertrophic cartilage stage (Fig. 8a and Supplementary Fig. 34), where the $Cu^{2+}$ release concentration exactly reaches the optimal level for vasculogenesis (Supplementary Fig. 5), thus promoting the transformation of hypertrophic chondrocytes to osteoblasts. It supports this process well rather than disrupting it through rapid and sudden release.

These optimized ion release profiles ensure that each layer of the scaffold provides the appropriate biological cues for the corresponding tissue type, thereby supporting heterogeneous regeneration of the osteochondral tissue. The detailed ion release curves and corresponding discussions have been added accordingly in the revised manuscript.

Revision: "So, we designed concentration gradient experiments to determine the optimal soaking conditions for the cartilage layer and subchondral bone layer, respectively. For the cartilage layer, we set up a gradient of $Mg^{2+}$ concentrations with 100, 200, 300 and 400 ppm. Alcian blue staining showed that in the 200-ppm group, the deposition of glycosaminoglycans was the most significant (Fig. 2f,g). The quantitative reverse transcription polymerase chain reaction (qRT-PCR) results also revealed that a $Mg^{2+}$ concentration of 200 ppm was the most beneficial for the upregulation of COL-II and SOX9 (Fig. 2h,i), which was highly consistent with the previous study[31]. For the subchondral bone layer, considering the potential cytotoxicity of high $Cu^{2+}$ concentration, we established a gradient of $Cu^{2+}$ gradients with 20, 40, 60, and 80 ppm[32]. Tubule formation assays revealed that a $Cu^{2+}$ concentration of 60 ppm exhibited the optimal vasculogenic effects, with the longest tubule length, largest tubule area, highest number of tubules and junctions (Fig. 2j-n). Notably, as both excessively high and low ion concentrations compromise biological efficacy, we sought to define the optimal soaking concentrations required to achieve target release levels of $Mg^{2+}$ and $Cu^{2+}$ for chondrogenic and osteogenic functions, respectively. To this end, we monitored the ion release profiles of hydrogels treated with varying concentrations of $Mg^{2+}$ and $Cu^{2+}$, establishing the correlation between initial soaking concentration and sustained release concentration. The results showed that the PAA-CHI-20 hydrogel soaked in a 0.5 M $MgSO_4$ solution continuously released $Mg^{2+}$ with a concentration reaching approximately 200 ppm, achieving best chondrogenic differentiation effect. When the PAA-CHI-20 hydrogel was soaked in a 0.1 M $CuSO_4$ solution, it could release $Cu^{2+}$ with a concentration of approximately 60 ppm, which matched the ideal vasculogenic concentration (Supplementary Fig. 5). The stable spatial distribution of $Mg^{2+}$ and $Cu^{2+}$ (with no significant cross-diffusion) was attributed to the high molar ratio of carboxyl groups to metal ions and the strong ion/chelation interaction, ensuring a continuous biochemical gradient over time. This rational matching of immersion concentration to the target release level enabled the precise temporal control of biological functions on the osteochondral scaffold."

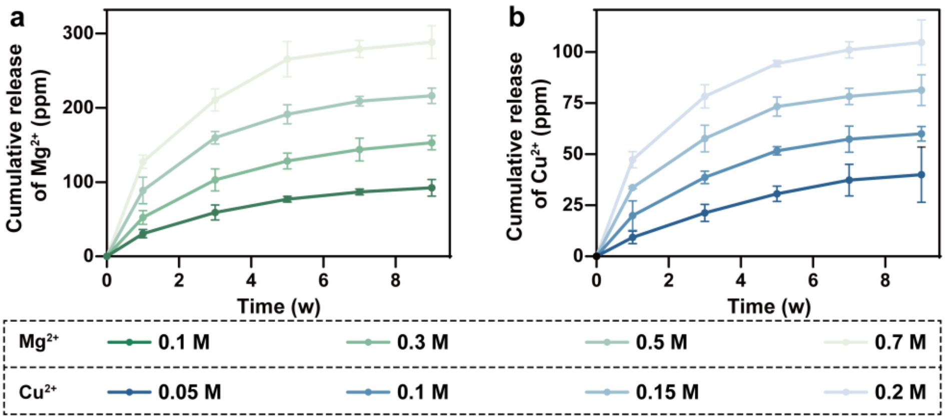


**Supplementary Fig. 5 The ion release curves from PAA-CHI-20 hydrogels after various ionic soaking treatment, respectively.** a) The release curves of $Mg^{2+}$ from the PAA-CHI-20 hydrogels after soaking into $MgSO_4$ solution with different concentrations. b) The release curves of $Cu^{2+}$ from the PAA-CHI-20 hydrogels after soaking into $CuSO_4$ solution with different concentrations. Data in a,b) were presented as means ± SD, n = 3.

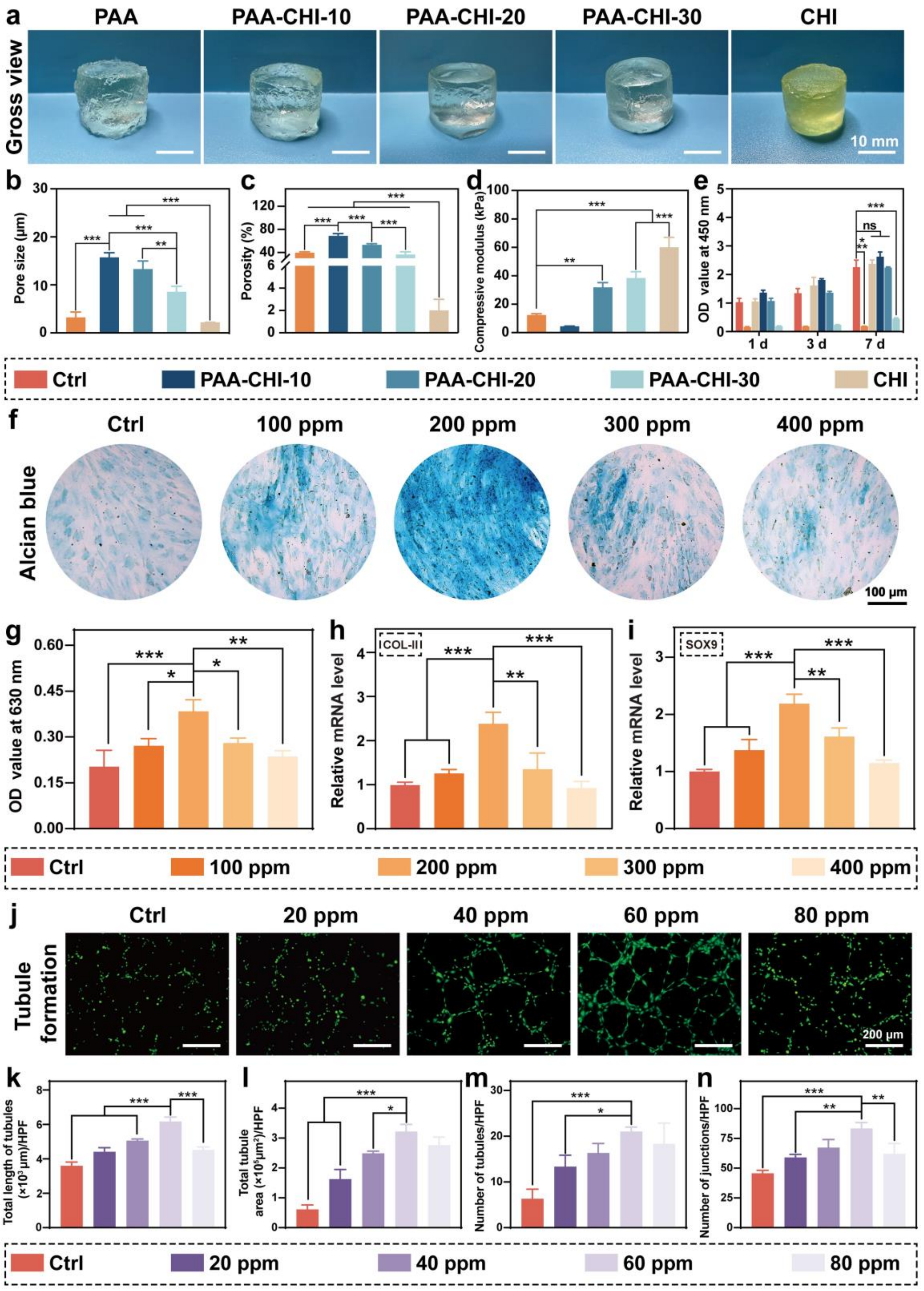


**Fig. 2 Optimization formulation of PAA-CHI hydrogels and ionic soaking concentration. a-d)** Gross view, pore size, porosity, and compressive behaviors of PAA, PAA-CHI-10, PAA-CHI-20, PAA-CHI-30, and CHI hydrogels. **e)** CCK-8 assay of BMSCs after co-culture with various hydrogels at specific times. **f)** Effect of $Mg^{2+}$ concentration on the formation of GAG by Alcian blue staining in BMSCs cultured for 2 weeks. **g)** Quantitative analysis of staining intensity in **f)**. **h, i)** Relative mRNA expression of chondrogenic differentiation-related indicators after co-culture of

BMSCs with different concentrations of $Mg^{2+}$ for 2 weeks. **j)** Live cell staining of HUVECs after co-culture with different concentrations of $Cu^{2+}$ for 6 h. **k-n)** Statistical analysis of the total length of tubules, total tubules area, number of tubules and junctions in high-magnification field at 6 h. Data in **b-e, g-i, k-n)** were presented as means ± SD, n = 3. Statistical significance was determined using the one-way ANOVA with Tukey's post-hoc test. *p < 0.05, **p < 0.01, ***p < 0.001.

5. Figure 3e could use an Ashby plot to demonstrate performance comparison rather than stress-strain curves from other studies.

**Reply 5:** Thanks for your kind suggestions. Your feedback is instrumental in refining the data presentation of our study and enhancing the clarity of key performance comparisons. We have replaced the stress-strain curves with Ashby plot to systematically compare the performance of our hydrogel system in the revised manuscript. The Ashby plot clearly contrasts the shear and stretch resistance of the WCCL-DG and the FCCL-DG hydrogel scaffolds, demonstrating that WCCL-DG outperforms FCCL-DG in both stretch and shear resistance (Fig. 6e). In terms of stretch resistance, the wave interface increases the interlayer contact area and enhances the interlayer bonding, thus improving the structural integrity under stretch loads. In terms of shear strength, the wave structure has created a mechanical interlocking effect, which is more significant in enhancing the overall shear resistance of the hydrogel compared to its stretch strength. Therefore, the Ashby plot presents the differences in performance between these two hydrogels in an intuitive and concise manner, enhancing the rigor of our data presentation and making our research results more systematic and persuasive.

Revision: "For interface stability, we characterized the interfacial bonding strength in horizontal and vertical directions using shear and stretch tests (Fig. 6e). The Ashby plot clearly contrasted the shear and stretch resistance of the WCCL-DG and the FCCL-DG hydrogel scaffolds, demonstrating that WCCL-DG outperforms FCCL-DG in both stretch and shear resistance. In terms of stretch resistance, the wave interface increased the interlayer contact area and enhanced the interlayer bonding, thus improving the structural integrity under stretch loads. In terms of shear strength, the wave structure had created a mechanical interlocking effect, partially preventing the detachment of the cartilage layer induced by lateral shear stress, which was more significant in enhancing the overall shear resistance of the hydrogel scaffold compared to its stretch strength."

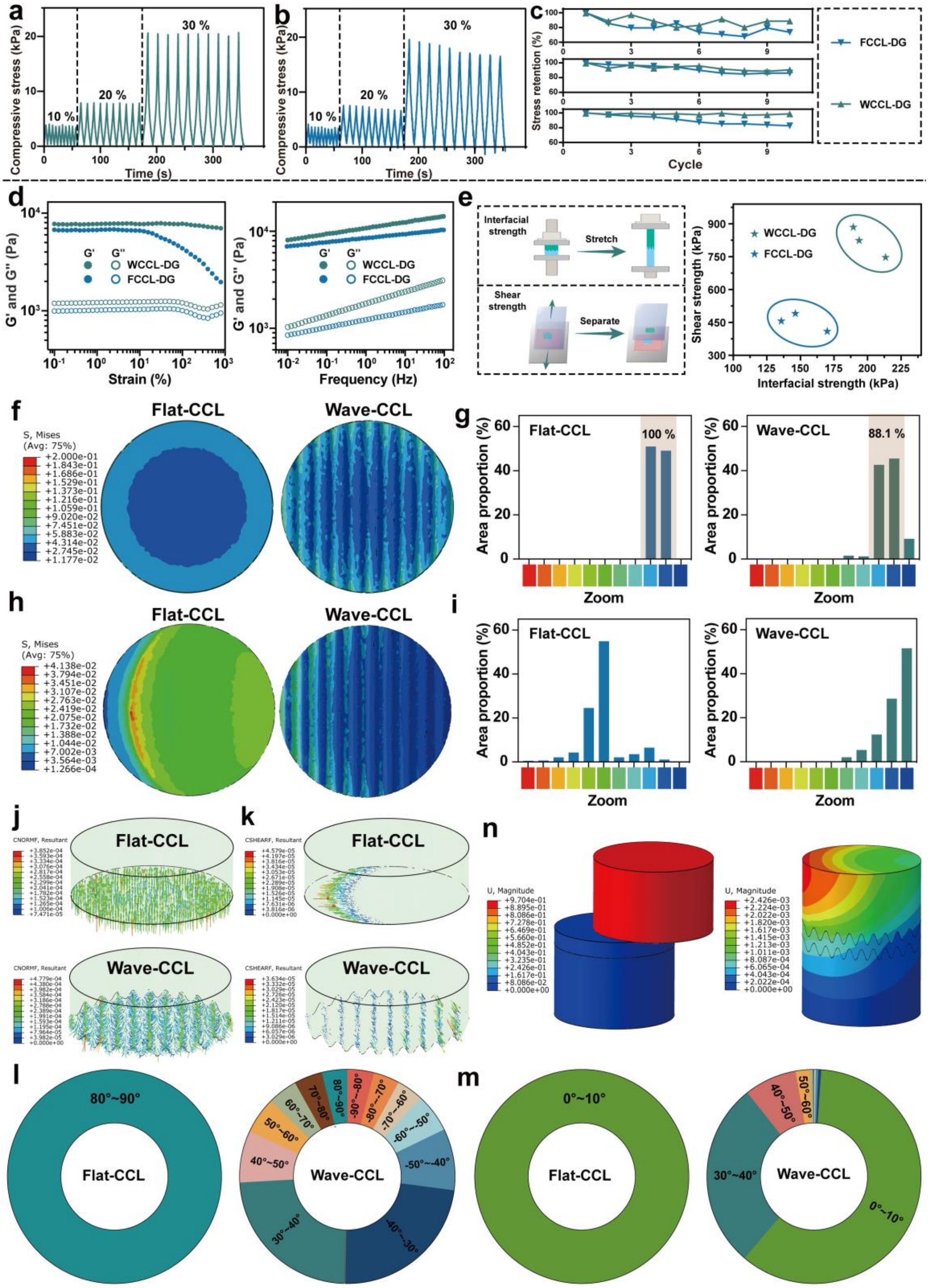


**Fig. 6 The mechanical differences between WCCL-DG and FCCL-DG. a)** The cyclic compression curves of WCCL-DG hydrogel under different strains. **b)** The cyclic compression curves of FCCL-DG hydrogel under different strains. **c)** The stress retention conditions of WCCL-DG hydrogel and FCCL-DG hydrogel during the cyclic compression process. **d)** Rheological properties of FCCL-DG and WCCL-DG hydrogels. **e)** Stretch and shear properties of WCCL-DG and FCCL-DG hydrogels. **f)** Thermograms of stress distribution on the lower surface of the cartilage

layer when two models were subjected to a compressive stress of 0.1 MPs respectively. **g)** Statistical analysis of the proportion of area occupied by different color blocks in **f)**. **h)** Thermograms of stress distribution on the lower surface of the cartilage layer when two models were subjected to a shear stress of 0.1 MPs respectively. **i)** Statistical analysis of the proportion of area occupied by different color blocks in **h)**. **j)** Direction of stress in the lower surface of the cartilage layer when subjected to compressive stress in two models. **k)** Direction of stress in the lower surface of the cartilage layer when subjected to shear stress in two models. **l)** Statistical analysis of stress of different ranges in **j)**. **m)** Statistical analysis of stress of different ranges in **k)**. **n)** Thermal map of overall displacement under shear stress for two models.

6. Finite element analysis should simulate practical application scenarios, such as implantation in osteochondral defects subject to confinement by surrounding tissues.

**Reply 6:** Thanks for your kind suggestions. We are extremely grateful for your valuable suggestions regarding the finite element analysis (FEA) simulation of the actual application scenarios. In our original FEA, we strictly followed the actual clinical application conditions. Specifically, we constructed a rectangular prism to simulate the constraining effect of the surrounding tissues on the osteochondral defect site, and placed a cylinder inside this rectangular prism to represent the osteochondral defect model where the implant was located (Supplementary Fig. 28). However, in the result section, we only displayed the isolated cylinder (defect + implant) instead of the entire rectangular prism model. This might lead to misunderstandings about whether the factor of surrounding tissue constraints was considered. We would like to confirm that all FEA results were derived from the complete model (rectangular prism + cylinder), which fully took into account the constraints of the surrounding tissues. Therefore, the conclusions regarding the mechanical behavior of the implant and the defect repair process remain valid and reliable.

The following is the model we initially developed. After applying different mechanical stimuli, we analyzed the cylindrical structure in the middle. Thanks again for your careful review and constructive suggestions. We have included the complete FEA model in the revised manuscript.

Revision:

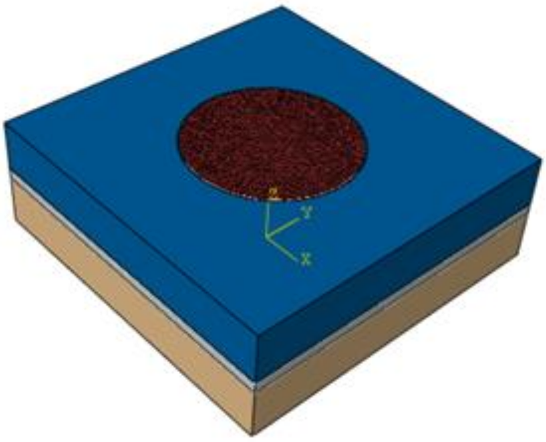


**Supplementary Fig. 28** The presentation and analysis section of the finite element model. After applying different mechanical stimuli, a force analysis was conducted on the middle red cylinder.

7. Regarding the advantages of this study compared to others: degradable hydrogels with modulus closer to cartilage have already been reported. The advantages of this work should be elaborated in detail in the Discussion section.

**Reply 7:** Thanks for your kind suggestions. We have systematically highlighted the core advantages of our system in the revised Discussion section. Our system unites three critical functions of decoupled spatiotemporal control, biomimetic biomechanical interface, and sequential biological processes, thereby surpassing state-of-the-art approaches. Firstly, we achieve toxicity-free intrinsic hypoxia mimicry via $Fe^{3+}$ chelation, avoiding cytotoxic exogenous agents like deferoxamine used in other studies (Nature 2019, 565, 511). Moreover, using spatiotemporal KGN distribution, our system inhibits chondrocytes hypertrophy in cartilage layer to preserve hyaline phenotype and promotes hypertrophy, vascularization, and ossification via ECO process in subchondral bone layer. Of note, these sequential biological processes (cartilage anti-hypertrophy and bone vascularization) work synergistically to achieve heterogeneous osteochondral regeneration. This is something that a single modulus-matched hydrogel cannot achieve due to the lack of precise control. Therefore, based on intrinsic hypoxia-inducible capacity by the scaffold and divisional intervention of chondrocyte metabolism using small KGN, it was particularly simple to pioneer a new spatiotemporal decoupling paradigm for synchronous regulation of cartilage anti-hypertrophy and bone pro-vascularization process. Additionally, the biomimetic wavy CCL effectively alleviates stress concentration and enhances shear resistance, thereby promoting cartilage regeneration and addressing a critical gap in existing scaffolds that always overlook the biomechanical functions of natural CCL. It is the first time to fabricate a biomimetic wavy CCL interface and reveal its biomechanical transmission and cartilage protection function in addressing the challenges of stress concentration and interfacial detachment via the finite element analysis and transcriptome studies, enabling the best therapeutic effect of full-thickness osteochondral defects. These comparisons and elaborations have been added into the Discussion section according to your constructive comments, which will significantly improve the quality of our manuscript.

Revision: "Compared with current state-of-the-art technologies such as cartilage-like modulus degradable hydrogels or gradient scaffolds, our system's core advantage lies in integrating rarely combined functional elements that address key gaps in these methods. As far as we know, most advanced methods are relatively simple in function and lack integrated regenerative capabilities. For example, gradient scaffolds always rely on the fixed biochemical cues but lack targeted control, and many modulus-matched hydrogels only meet mechanical requirements but neglect the reconstruction of wavy CCL with key mechanical properties, not to mention the regulation of differentiation signals. In contrast, our system unites three critical functions of decoupled spatiotemporal control, biomimetic biomechanical interface, and sequential biological processes, thereby surpassing state-of-the-art approaches. The bidirectional soaking strategy not only constructed a biochemical gradient of $Mg^{2+}$ in the upper layer and $Cu^{2+}$ in the lower layer but also generated the adjustable pore size and modulus gradients, which could simultaneously regulate cell

differentiation and structural-mechanical properties of hydrogel scaffolds. Furthermore, the scaffolds possessed inherent iron chelation capabilities to trigger a hypoxic microenvironment by activating HIF-1α. In the cartilage layer, HIF-1α worked in synergy with $Mg^{2+}$ to promote chondrogenic differentiation of BMSCs; while in the subchondral bone layer, HIF-1α worked in synergy with $Cu^{2+}$ to promote vasculogenesis, thereby facilitating the process of ECO and achieving the effect of homologous initiation - heterochronic differentiation. Notably, KGN in the cartilage layer prevented chondrocytes hypertrophy caused by long-term HIF-1α activation, representing a repurposing of KGN beyond its conventional use. Under this circumstance, this decoupling strategy could control HIF-1α expression spatially and temporally, inhibiting hypertrophy in cartilage while promoting ECO in subchondral bone, beyond the existing literatures through integrated innovations that addressed unmet needs in osteochondral repair. Additionally, we innovatively developed a biomimetic wavey CCL, which effectively alleviated stress concentration and enhanced shear resistance, addressing a critical gap in existing scaffolds that overlooked the biomechanical functions of natural CCL. It was the first time to fabricate a biomimetic wavy CCL interface and reveal its biomechanical transmission and cartilage protection function in addressing the challenges of stress concentration and interfacial detachment via the FEA and transcriptome studies, enabling the best therapeutic effect of full-thickness osteochondral defects."

8. The strength and toughness of the hydrogel can be counted, because it seems that strength is a significant advantage, and the modulus is far less than the natural cartilage tissue.

**Reply 8:** Thanks for your kind suggestions. In fact, we have made some attempts to enhance the modulus of the hydrogel scaffold, such as increasing the solid content of PAA and ionic soaking concentration (Adv. Sci. 2023, 10, 2206242). However, high PAA concentration can cause a decrease in the environmental pH value while high ionic soaking concentration also brings about a certain biological toxicity, which can inhibit cell proliferation and damage biocompatibility (Supplementary Fig. 4). Furthermore, the compressive modulus of natural subchondral bone can typically reach the GPa level, which has exceeded the mechanical modulus upper limit of all degradable hydrogels. So, taking into account the biocompatibility, mechanical performance, biological characteristics and feasible accessibility for the hydrogel scaffold and avoidance of the unnecessary excessive variables introduced by other additives (such as inorganic nanoparticles), here in this work, we have decided not to consider significantly increasing the compressive modulus. Instead of overemphasizing complete modulus matching with natural tissues, we concentrated on the regulatory effects of the cartilage hydrogel and the subchondral bone hydrogel on cell fate, including the maintenance of hyaline cartilage phenotype in the cartilage layer and the promotion of vasculogenesis in the subchondral bone layer, supported by detailed cellular experimental data and molecular mechanism analysis (Fig. 4d-f, Fig. 5 and Supplementary Fig. 26). This discovery highlights the necessity of balancing mechanical performance and biological functionality when designing scaffolds, which is also an important consideration in our design process. In addition, we would like to briefly mention that the modulus of our hydrogel is not significantly different from

the previous literatures (Adv. Mater. 2024, 36, 2409400; Bioact. Mater. 2023, 19, 678), which indicates that the current design meets the basic mechanical requirements for osteochondral repair. We once again express our gratitude for your valuable constructive suggestion. We will further refine the relevant content in the revised manuscript to enhance the scientific rigor and completeness of our research.

Finally, we sincerely thanks for your meticulous review of our manuscript and your constructive suggestions, and hope our explanation can get your understanding and recognition. We recognize that this feedback is of great importance in further refining and enhancing our research outcomes.

# A point-by-point response to the reviewers

Dear reviewers:

Thank you very much for your kind advice and valuable comments in helping us improve our m anuscript (Title: "A decoupling strategy toward spatiotemporal regulation and biomechanical transmission of sandwiched scaffold for full-thickness osteochondral regeneration"). We have su bstantially modified the manuscript, according to the questions raised by the reviewers. All the modified words, sentences and paragraphs were labeled with red fonts. A point-to-point response t o highlight how we have addressed each of the comments is listed below.

Reviewer #1 (Remarks to the Author):

The authors have addressed most of the concerns and comments in an acceptale way, though some questions are not supplemented with extra data in this revision, the authors pointed out the limitations in the manuscript. Therefore, due to their attempts on waved interfaces and tissue-specific hydrogel compositions for osteochondral repair, it shows the value for publication.

Reply: Thank you very much for your professional suggestions, which have provided important guidance for the optimization of our work. We are honored to receive your recognition of our work, which will motivate us to keep improving.

Reviewer #2 (Remarks to the Author):

The revisions provided by the authors have improved the manuscript, and we appreciate their response to the reviewers' comments.

However, the manuscript still falls short of the level of completeness and clarity required for publication in this journal. Several important aspects, such as experimental design, data interpretation, and writing presentation, need further attention.

We therefore recommend another round of major revisions, with a focus on the points detailed below. We are confident that these changes will enhance the manuscript's quality and impact.

1. The article does not provide precise geometric parameters of the template (such as wavelength, amplitude), materials, or preparation methods, which may affect the reproducibility of the experiments.

**Reply 1:** Thanks for your kind suggestions. In the revised manuscript, we have added a detailed description of the template's material, geometry, and manufacturing process. Specifically, we have specified that the templates were fabricated from DPI 8400 polyurethane resin using a vacuum casting technique. Furthermore, we have clarified the technical parameters of the preparation process in the revised Supplementary Table 3, such as the vacuum degassing step and the specific mold pre-heating temperature required to ensure dimensional accuracy. We believe these additions adequately address the concerns regarding experimental reproducibility.

Revision:

**Process of template manufacture**

The biomimetic wavy templates were fabricated using a three-component vacuum polyurethane resin (DPI 8400). The geometric profile of the interface was designed via Computer-Aided Design (CAD) to mimic the natural tidemark topography of osteochondral tissue. To prepare the templates, a master model was first created using high-precision 3D printing to produce a negative silicone rubber mold. The DPI 8400 resin components were mixed in a specific weight ratio and subjected to vacuum degassing to ensure the removal of all air bubbles. The mixture was then poured into the silicone mold, which had been pre-heated to 60–70 °C to ensure complete curing and optimal dimensional stability. Following polymerization and demolding, the high-precision polyurethane templates were utilized as the structural base for the subsequent layered polymerization of the hydrogel scaffold, ensuring the accurate replication of the biomimetic wavy interface.

**Supplementary Table 3.** Template parameters.

| Name | Amplitude (mm) | Wavelength (mm) |
|---|---|---|
| M1 (Origin) | 0.15 | 0.3 |
| M2 | 0.15 | 0.15 |
| M3 | 0.15 | 0.45 |
| M4 | 0.2 | 0.3 |
| M5 | 0.1 | 0.3 |

2. Although "control of the soaking depth" is mentioned, no specific control methods (e.g., visual systems, mechanical positioning) are described, nor is the uniformity of ion diffusion during soaking or batch-to-batch variability evaluated.

**Reply 2:** Thanks for your kind suggestions. Regarding the control of the soaking process, we have implemented a mechanical positioning strategy to ensure both precision and reproducibility. Specifically, we used an XYZ Linear Stage System (Zolix Instruments Co., Ltd.) with a closed-loop control system. This system, featuring a high-resolution optical encoder, allows for precise vertical adjustment of the scaffold with an accuracy of ± 0.05 mm. This mechanical control effectively eliminates manual errors and ensures consistent soaking depths across all samples.

Furthermore, the uniformity of ion diffusion was verified via EDS mapping (Fig. 3b and Supplementary Fig. 7). The results demonstrated that $Mg^{2+}$ and $Cu^{2+}$ were homogeneously distributed within their respective target layers without localized enrichment. By combining high-precision mechanical positioning with these post-fabrication validations, we have minimized the batch-to-batch variability.

3. The influence of wave geometric parameters (e.g., curvature, thickness) on stress distribution and cellular behavior is not explored.

**Reply 3:** Thanks for your kind suggestions. We fully agree that the geometric parameters of the wavy interface significantly influence the mechanical microenvironment and subsequent cellular

responses. To address this point, we have developed four additional models (M2-M5) by independently modulating the wavelength and amplitude of the wavy interface based on the original model (M1), as shown in the revised Supplementary Table 3. Specifically, the FEA results under 0.1 MPa compressive stress revealed that a decrease in wavelength led to progressive stress concentration, particularly in the M2. Although wavy scaffolds could maintain a multidirectional stress mode, our results indicated that disproportionate stress distribution in models with excessive amplitude or short wavelength (e.g., M2 and M4) could restrict coordinated cartilage development.

Consistent with the mechanical simulations, biological evaluations using BMSCs-seeded scaffolds under dynamic loading demonstrated that although all wavy geometries promoted higher COL-II secretion than flat controls, the M2 and M4 groups exhibited lower chondrogenic differentiation due to stress concentration and uneven stress distribution. These findings highlight that optimized geometric parameters are critical for ensuring high-quality cartilage regeneration.

Thank you for the inspiration brought by your suggestions and guidance. Here, we have merely explored some simple models (M2-M5). In future work, we will conduct a more detailed and in-depth analysis to further refine the impact of wavey parameters and mechanical properties on cartilage protection, cell activity, and mechanical dispersion.

Revision:

To investigate the influence of geometric parameters on stress distribution and cellular behavior, four additional models (M2-M5) were developed based on the original model (M1) by independently modulating the wavelength and amplitude of the wavy interface (Supplementary Fig. 32a). After applying a compressive stress of 0.1 MPa, the mechanical response of the cartilage layer varies significantly with changes in geometric parameters of the interface. Specifically, as the wavelength shortens, the local stress on the cartilage layer increased, with the M2 showing the most obvious stress concentration phenomenon (Supplementary Fig. 32b,c). Meanwhile, the increase in wave amplitude also contributed to the enhancement of stress intensity to a certain extent. Additionally, quantitative analysis of the stress direction indicated that although the M2 and M4 still maintained a multi-directional stress distribution pattern, the proportion differences between different intervals were too large. This highly unbalanced mechanical stimulation to some extent restricted the coordinated development of cartilage tissue (Supplementary Fig. 32d,e). In contrast, the M3 and M5 exhibited a stress direction distribution highly similar to that of the original M1, providing a more optimized mechanical microenvironment.

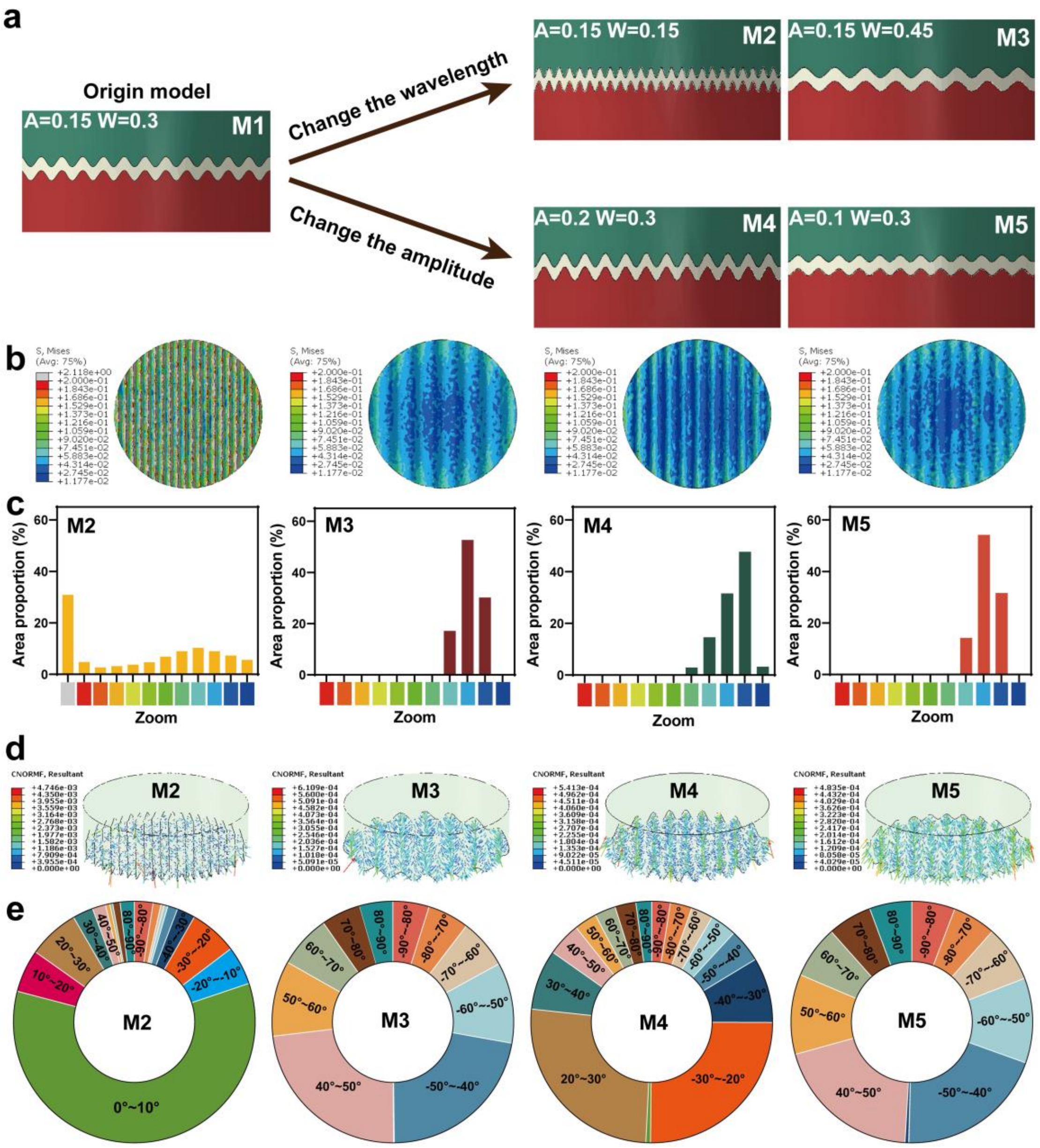


**Supplementary Fig. 32 Stress responses of models with different parameters. a)** Schematic diagram of models' construction with different parameters. W: Wavelength, A: Amplitude. M: Model. **b)** Thermograms of stress distribution on the lower surface of the cartilage layer when these models were subjected to a compressive stress of 0.1 MPs respectively. **c)** Statistical analysis of the proportion of area occupied by different color blocks in **b)**. **d)** Direction of stress in the lower surface of the cartilage layer when subjected to compressive stress in these models. **e)** Statistical analysis of stress of different ranges in **d)**.

In terms of biological evaluation, we fabricated five groups of osteochondral scaffolds using the corresponding biomimetic templates (Supplementary Fig. 33a) and investigated the biological responses of BMSCs under dynamic mechanical stimulation (Supplementary Fig. 33b). The qRT-PCR and IF staining results showed that compared to the flat scaffold, the wavy interface scaffolds significantly promoted the secretion of more COL-II by BMSCs, once again confirming the

superiority of biomimetic wavy structure (Supplementary Fig. 33c,d). However, the chondrogenic differentiation levels of the M2 and M4 groups were overall lower than those of the M1, M3, and M5 groups. This result was highly consistent with the FEA prediction, indicating that the uneven distribution of stress directions weakened the chondrogenic efficacy of the scaffolds.

These results indicate that when designing bionic osteochondral scaffolds, the optimization of the parameters of the wavey structure is of crucial importance. Excessively high amplitudes or overly short wavelengths will lead to stress concentration and imbalance in the distribution of stress, thereby hindering the regeneration of high-quality cartilage."

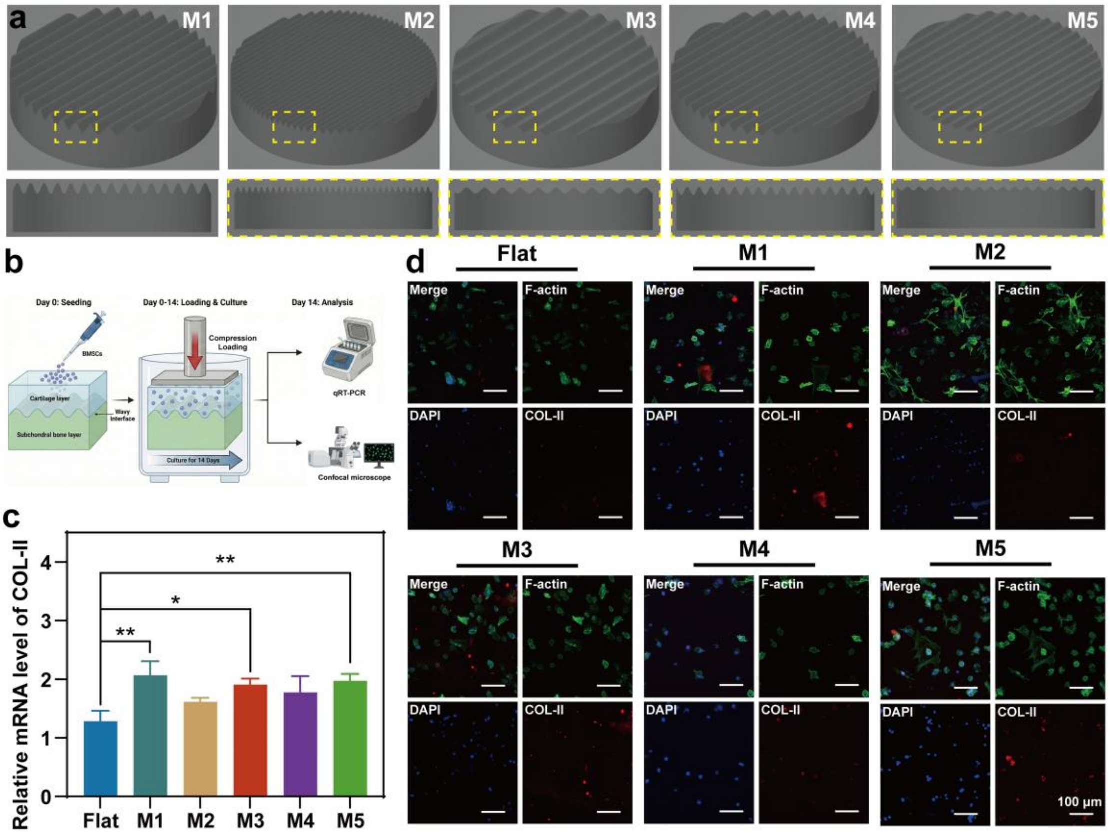


**Supplementary Fig. 33 Chondrogenic differentiation of BMSCs cultivated in scaffolds with different parameters. a)** Drawings for manufacturing wave-shaped templates with different parameters. **b)** Schematic diagram of the cultivation and analysis of BMSCs. **c)** Relative mRNA expression of COL-II of BMSCs cultured in different scaffolds for 14 days. **d)** IF staining of COL-II and F-Actin of BMSCs cultured in different scaffolds for 14 days. Data were presented as means ± SD, n = 3. Statistical significance was determined using the one-way ANOVA with Tukey's post-hoc test. *$p < 0.05$, **$p < 0.01$, ***$p < 0.001$.

4. The scale bars in the upper and lower groups of supplementary Fig. 7 are inconsistent.

**Reply 4:** Thanks for your kind suggestions. We have carefully checked and revised the figure. The scale bars of Supplementary Fig. 7 have now been unified to ensure consistency and accuracy in visual presentation. We greatly appreciate your attention to this detail, which helps improve the quality of our manuscript.

Revision:

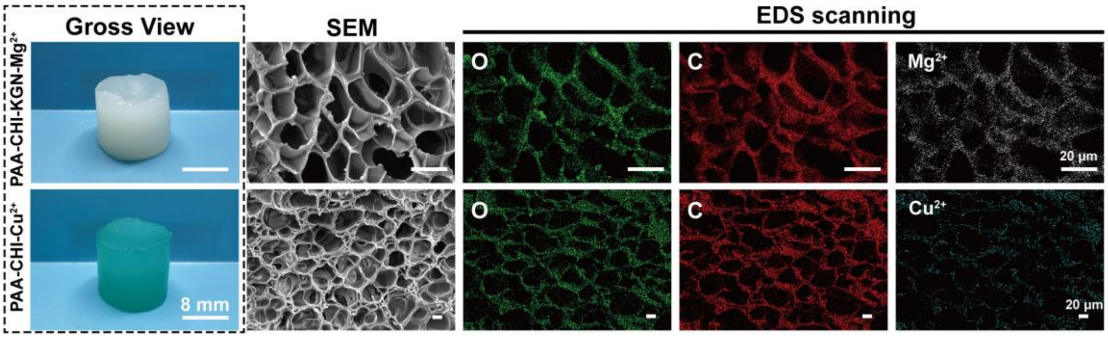


5. How were the hydrogels sterilized?

**Reply 5:** Thanks for your kind suggestions. We adopted a strict aseptic processing procedure for the hydrogel. Firstly, we treated the hydrogel solution with a sterile filter membrane to remove any microbial contaminants; then, we cross-linked the filtered solution into a hydrogel under ultraviolet irradiation in a clean bench. Throughout the entire process, we strictly followed the aseptic operation standards to ensure the sterility of the final hydrogel products.

6. The Methods section lacks the experimental procedure for measuring ion release from PAA-CHI-20 hydrogels after different ionic soaking treatments.

**Reply 6:** Thanks for your kind suggestions. Regarding your comment on the experimental procedure for measuring ion release from PAA-CHI-20 hydrogels after different ionic soaking treatments, we would like to clarify that the relevant experimental steps have already been detailed in the Methods section of our original submission. To facilitate your quick reference and review, we have highlighted this specific content in red within the revised manuscript, which was consistent with the previous literature (Adv. Funct. Mater. 2023, 33, 2304829). We greatly appreciate your attention to detail and hope this clarification addresses your concern effectively.

7. Bacterial specific terms and abbreviations should be italicized (line 292).

**Reply 7:** Thanks for your kind suggestions. We have changed the specific terms and abbreviations related to bacteria in line 292 to be in italics. Additionally, we have conducted a thorough review of the entire manuscript to ensure that all such forms are consistently presented throughout, adhering to the academic writing standards of microbiological terminology.

8. Some figure and caption annotations are unclear (e.g., certain explanations in Fig. 1a are missing and are placed below Fig. 1b, affecting readability).

**Reply 8:** Thanks for your kind suggestions. We have carefully revised the manuscript to address this issue. The missing explanatory notes originally placed below Fig. 1b have been moved to the corresponding position under Fig. 1a, which significantly enhances the readability and logical correspondence between the figure and its annotations. Additionally, we have conducted a comprehensive check of all figures and their captions to ensure that all annotations are accurately positioned and clearly presented in the revised manuscript.

Revision:

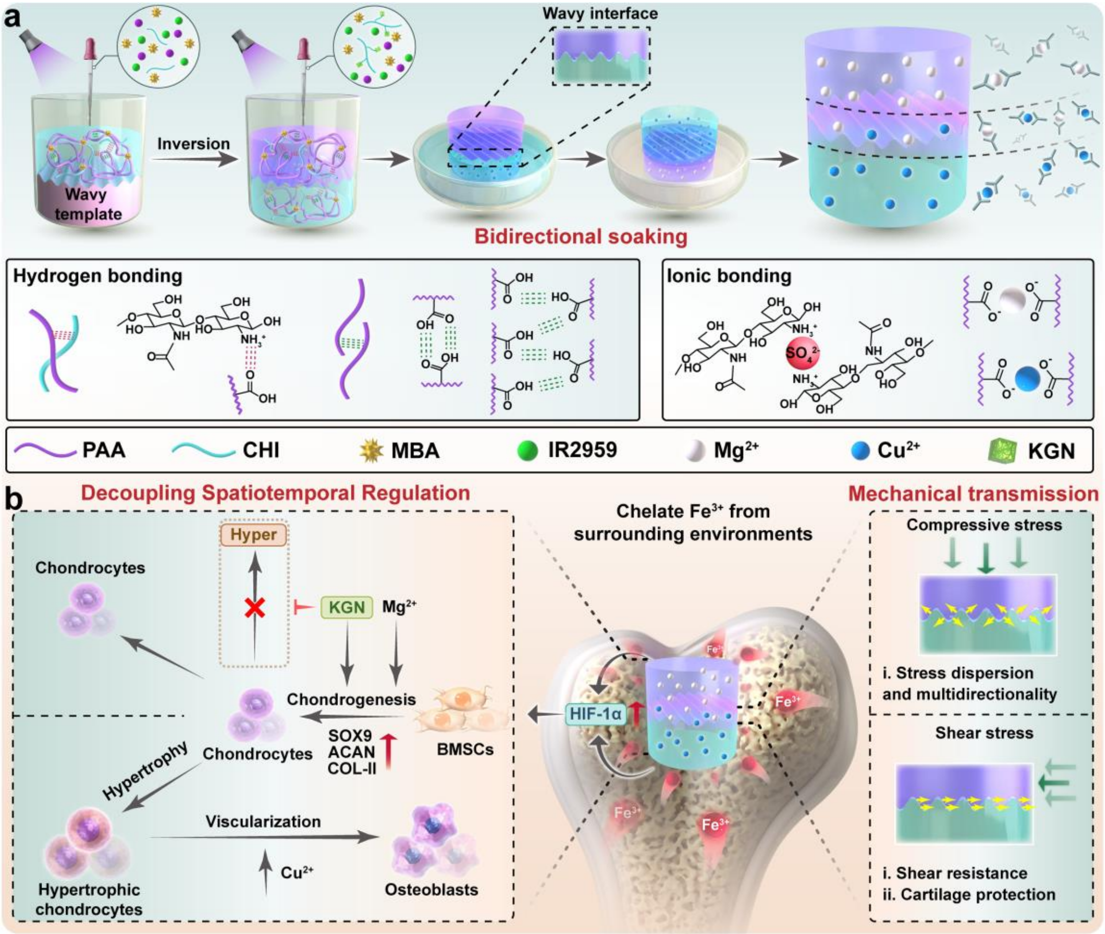


9. The manuscript would benefit from grammatical polishing.

**Reply 9:** Thanks for your kind suggestions. We greatly appreciate your attention to detail, which is crucial for enhancing the clarity, precision, and professionalism of our manuscript. We have conducted a comprehensive review and systematically revised all identified grammatical issues, including correcting noun number agreement, refining verb-preposition collocations, optimizing redundant expressions, and adjusting article usage to ensure fluent and accurate academic writing. These revisions have been integrated to maintain consistency in tone and readability. We believe these changes have significantly improved the overall quality of our work and effectively addressed your concerns.

Reviewer #3 (Remarks to the Author):

I co-reviewed this manuscript with one of the reviewers who provided the listed reports. This is part of the Nature Communications initiative to facilitate training in peer review and to provide appropriate recognition for Early Career Researchers who co-review manuscripts.

Reply: Thank you very much for your professional suggestions, which have provided important guidance for the optimization of our work. We are honored to receive your recognition of our work, which will motivate us to keep improving.

Reviewer #4 (Remarks to the Author):

Thank you for submitting your revised manuscript and for your thorough responses to the reviewers' comments. I have carefully reviewed your detailed point-by-point responses and the corresponding revisions made to the manuscript. Therefore, based on the substantial improvements made to the manuscript, I recommend Acceptance of this manuscript for publication in its current form.

Reply: Thank you very much for your professional suggestions, which have provided important guidance for the optimization of our work. We are honored to receive your recognition of our work, which will motivate us to keep improving.
