# Supplementary material for: Author Correction: A decoupling strategy toward spatiotemporal regulation and biomechanical transmission of sandwiched scaffold for osteochondral regeneration
Source: Nat Commun. 2026 Oct 26;17:11122. doi: 10.1038/s41467-026-79199-w (PMC13710949; doi:10.1038/s41467-026-79199-w)
Supplement: Supplementary file 1 [file 41467_2026_79199_MOESM1_ESM.pdf]

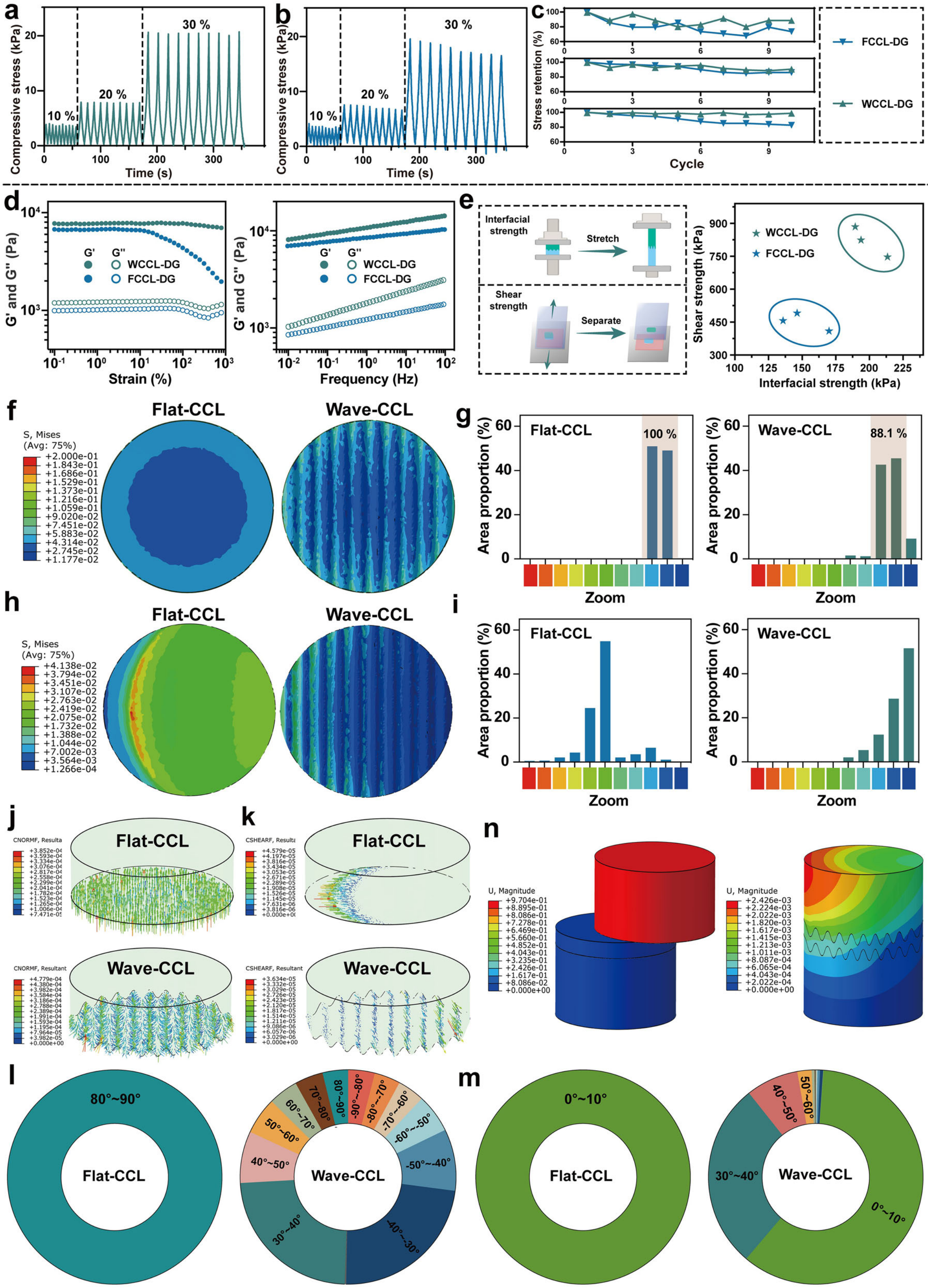

a
b
c
Compressive stress (kPa)
Time (s)
10 %
20 %
30 %
Stress retention (%)
Cycle
FCCL-DG
WCCL-DG
d
G' and G'' (Pa)
Strain (%)
Frequency (Hz)
G'
G''
WCCL-DG
FCCL-DG
e
Interfacial strength
Stretch
Shear strength
Separate
Shear strength (kPa)
Interfacial strength (kPa)
f
Flat-CCL
Wave-CCL
S, Mises
(Avg: 75%)
g
Area proportion (%)
100 %
88.1 %
Zoom
h
i
j
k
n
U, Magnitude
l
m
80°~90°
0°~10°
30°~40°
40°~50°
50°~60°
-40°~-30°
-50°~-40°
